# Cobalt- or rhodium-catalyzed synthesis of 1,2-dihydrophosphete oxides *via* C–H activation and formal phosphoryl migration†

**DOI:** 10.1039/d4sc00649f

**Published:** 2024-03-14

**Authors:** Shengbo Xu, Ruijie Mi, Guangfan Zheng, Xingwei Li

**Affiliations:** a School of Chemistry and Chemical Engineering, Shaanxi Normal University (SNNU) Xi'an 710062 P. R. China lixw@snnu.edu.cn; b Institute of Molecular Science and Engineering, Institute of Frontier and Interdisciplinary Sciences, Shandong University Qingdao 266237 P. R. China; c Department of Chemistry, Northeast Normal University Changchun 130024 P. R. China

## Abstract

A highly stereo- and chemoselective intermolecular coupling of diverse heterocycles with dialkynylphosphine oxides has been realized *via* cobalt/rhodium-catalyzed C–H bond activation. This protocol provides an efficient synthetic entry to functionalized 1,2-dihydrophosphete oxides in excellent yields *via* the merger of C–H bond activation and formal 1,2-migration of the phosphoryl group. Compared with traditional methods of synthesis of 1,2-dihydrophosphetes that predominantly relied on stoichiometric metal reagents, this catalytic system features high efficiency, a relatively short reaction time, atom-economy, and operational simplicity. Photophysical properties of selected 1,2-dihydrophosphete oxides are also disclosed.

## Abstract 2

1,2-Dihydrophosphete oxides have been accessed in excellent yields *via* the merger of metal-catalyzed C–H bond activation and formal 1,2-migration of a phosphoryl group.

## Graphical abstract

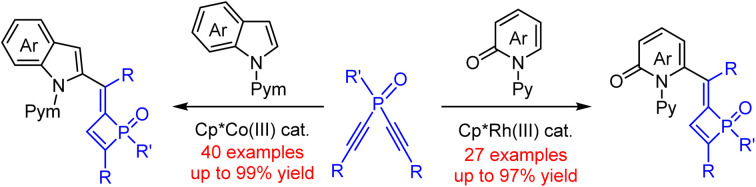

## Introduction

**Fig. 1:**
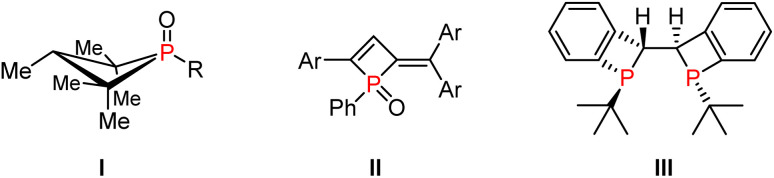
Selected examples of four-membered phosphacycles.

**Scheme 1:**
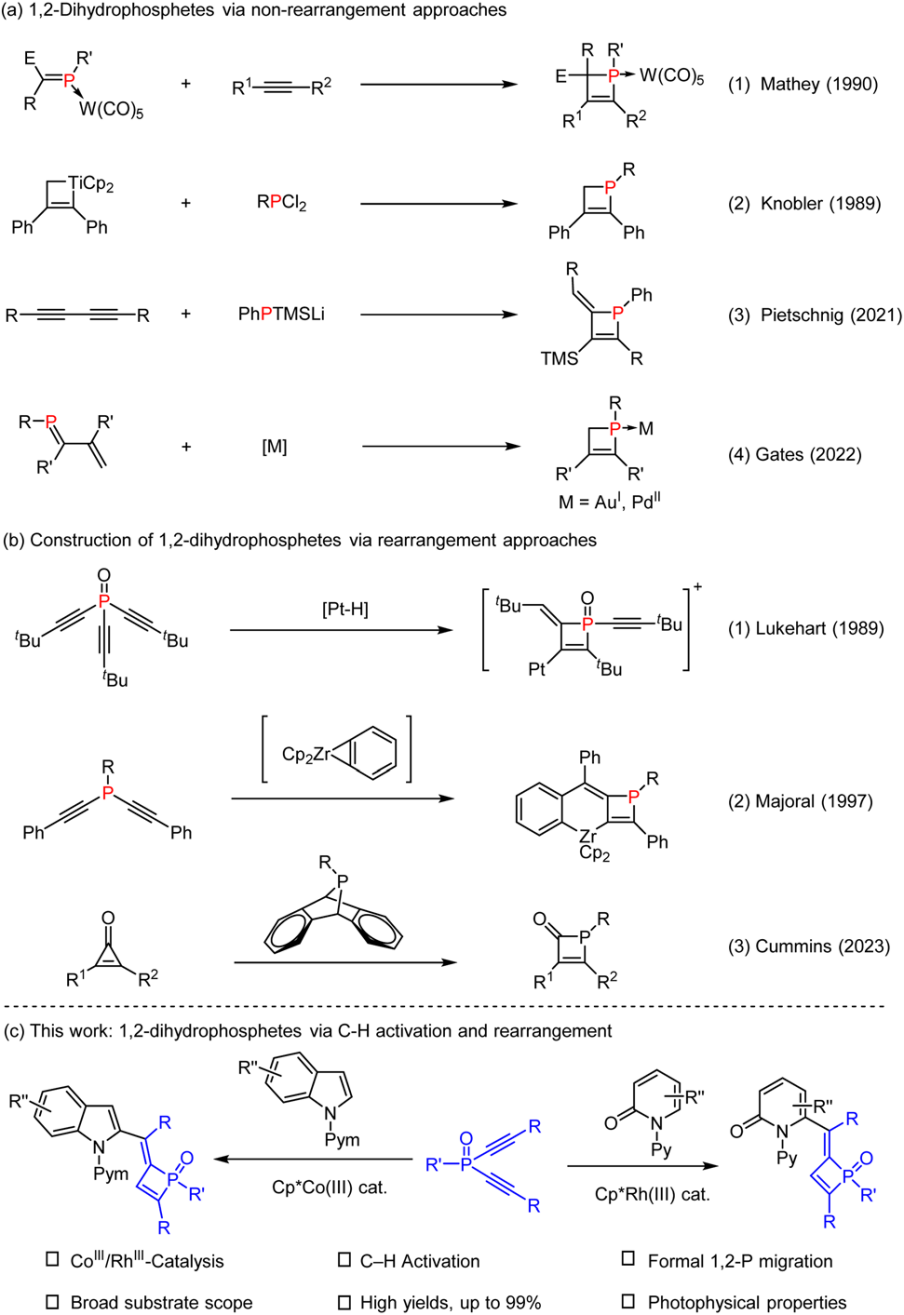
Synthesis of 1,2-dihydrophosphetes.

Organophosphorus molecules are an important class of compounds which are not only widely utilized as ligands or organocatalysts for diverse transformations but also function as drugs and bioactive molecules.^1^ The development of efficient methods for the synthesis of functionalized organophosphorus compounds has attracted continuous attention.^2^ In particular, four-membered phosphorus compounds have found vast applications in the studies of catalysis, medicinal chemistry, and materials science (Fig. 1).^3^ For instance, compound I is known as a new organic catalyst that enables the reductive C–N cross-coupling of functionalized nitroalkanes with arylboronic acids, and compound II exhibits unique photophysical properties, while compound III is employed as a useful chiral bidentate ligand. However, efficient methods to access four-membered phosphacycles, especially 1,2-dihydrophosphete (oxides), only remain sporadic. In 1985, Mathey and co-workers reported synthesis of metal complexes of 2-keto-1,2-dihydrophosphetes *via* CO insertion into a P–C bond of phosphirene-chromium, -molybdenum, and -tungsten pentacarbonyl complexes as a result of ring expansion.^4^ After that, they further disclosed coupling of electron-poor phospha-alkene P–W(CO)_5_ complexes with electron-rich alkynes *via* [2 + 2] cycloadditions (Scheme 1a1).^5^ In 1989, Knobler reported the reaction of diphenyltitanacyclobutene with phenyldichlorophosphine, allowing the first isolation of free 1,2-dihydrophosphetes (Scheme 1a2).^6^ In 2021, Pietschnig and co-workers established a transition-metal free annulation reaction between 1,3-diynes and phosphanides (Scheme 1a3).^7^ Recently, Gates accomplished cyclization of 1-phosphabutadiene and isolated Au- and Pd-stabilized 1,2-dihydrophosphete complexes (Scheme 1a4).^8^

Rearrangement reactions provide straightforward and efficient access to complex organic frameworks *via* structural reorganizations.^9^ In 1989, Lukehart developed stoichiometric reactions of trialkynylphosphine oxide and platinum hydride that involve Pt–H addition and rearrangement reactions (Scheme 1b1).^10^ In 1997, Majoral reported synthesis of metallacycle-supported 1,2-dihydrophosphete *via* coupling of bis(alkynyl)phosphines with zirconocene-benzynes (Scheme 1b2).^11^ Very recently, Cummins realized phosphinidene transfer to cyclopropenones for synthesis of phosphet-2-ones(Scheme 1b3).^12^ Despite these strategies, the majority of systems require the employment of stoichiometric amounts of metal reagents such as zirconium, platinum, titanium, and tungsten complexes. In addition, the reaction scope is also limited due to compatibility issues. Therefore, efficient synthesis of 1,2-dihydrophosphetes from readily available reagents still awaits further development.

We reasoned that 1,2-dihydrophosphetes can be accessed *via* catalytic manipulation of the triple bond in dialkynylphosphine (oxides). Indeed, metal-catalyzed coupling of dialkynylphosphine oxides/sulfides delivered various powerful strategies to create complex phosphacycles.^13^ The C≡C bond in the dialkynylphosphine is expected to readily participate as a special π-bond in C–H activation reactions, and the resulting alkenyl phosphine intermediate may also undergo rearrangement to give complex structures. While significant achievements have been made in C–H bond activation catalyzed by transition metals such as Pd, Rh, Ru, Ir, Mn, and Co,^14^ P-containing substrates are generally less explored either as the arene or the coupling reagent.^15^ Consequently, catalytic construction of four-membered phosphacycles remains a formidable challenge. By taking advantage of ready availability of arenes, our objective was to synthesize 1,2-dihydrophosphetes *via* integration of C–H bond activation and migration of the P atom in the reactive intermediate. Herein, we report the development of cobalt^III^ or rhodium^III^-catalyzed C–H activation of diverse classes of heteroarenes and coupling with dialkynylphosphine oxides, which affords a series of rare 1,2-dihydrophosphete oxides in high yields (Scheme 1c). The mechanism of this reaction has also been briefly explored.

## Results and discussion

We commenced our studies with optimization of reaction parameters of the coupling of 1-(pyrimidin-2-yl)-1*H*-indole 1a and methylbis(phenylethynyl)phosphine oxide 2a (Table 1). The reaction occurred at 100 °C in the presence of a Cp*Co(CO)I_2_/AgSbF_6_ catalyst in DCE for 3 h, and the desired four membered P^V^=O product (3a) was isolated in 95% yield. It was found that Cp*Co(CO)I_2_ showed superiority to other catalysts such as [Cp*RhCl_2_]_2_ and [Cp*Co(MeCN)_3_](BF_4_)_2_ (Table 1, entries 1–3). The screening of solvents showed that TFE was the best choice (Table 1, entries 4–8), and a nearly quantitative yield was realized when the reaction was performed at 100 °C (entry 8). In addition, the reaction efficiency was slightly affected when the reaction temperature was lowered from 100 °C to 60 °C (entries 8–12).

**Table 1:** Optimization studies^a^

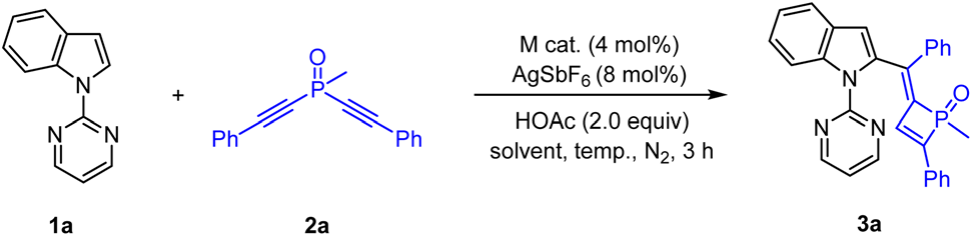				
Entry	Catalyst	Solvent	Temp. (°C)	Yield^b^ (%)
1	[Cp*RhCl_2_]_2_^c^	DCE	100	78
2	[Cp*Co(MeCN)_3_](BF_4_)_2_	DCE	100	93^d^
3	Cp*Co(CO)I_2_	DCE	100	95
4	Cp*Co(CO)I_2_	MeCN	100	43
5	Cp*Co(CO)I_2_	THF	100	Trace
6	Cp*Co(CO)I_2_	EA	100	35
7	Cp*Co(CO)I_2_	Dioxane	100	40
8	Cp*Co(CO)I_2_	TFE	100	98
9	Cp*Co(CO)I_2_	TFE	90	96
10	Cp*Co(CO)I_2_	TFE	80	91
11	Cp*Co(CO)I_2_	TFE	70	90
12	Cp*Co(CO)I_2_	TFE	60	93

a The reactions were carried out with 1a (0.13 mmol), 2a (0.10 mmol), M cat. (4 mol%), AgSbF_6_ (8 mol%) and HOAc (2.0 equiv.) in a solvent (1.0 mL) under N_2_ for 3 h.

b Isolated yields.

c AgSbF_6_ (16 mol%) was used.

d No AgSbF_6_ was used.

**Scheme 2:**
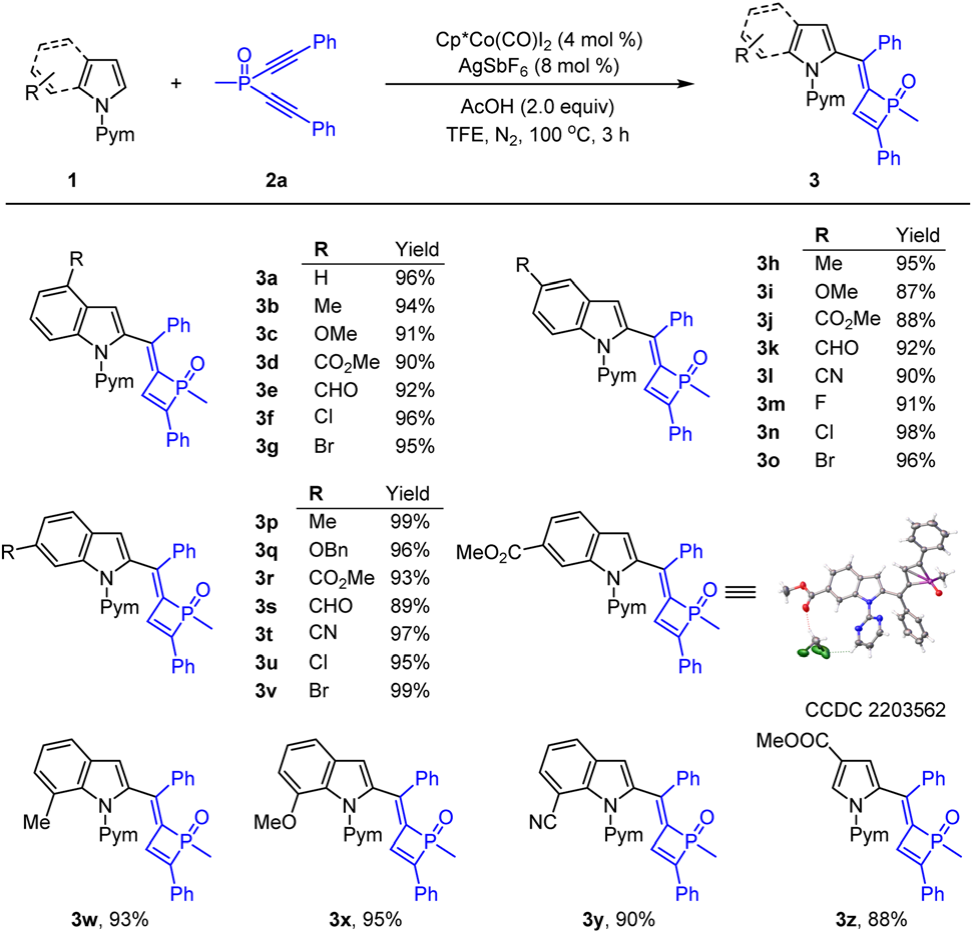
Substrate scope of indoles by cobalt-catalysis. Conditions: indoles 1 (0.26 mmol), dialkynylphosphine oxide 2a (0.20 mmol, 1.0 equiv.), Cp*Co(CO)I_2_ (4.0 mol%), AgSbF_6_ (8 mol%), HOAc (2.0 equiv.) in 2.0 mL of TFE under N_2_ at 100 °C for 3 h. Isolated yields.

With the optimized conditions established, we next evaluated the generality of this transformation (Scheme 2). Indoles with a variety of functionalities at the 4-position such as methyl, methoxy, halogens, ester group and formyl reacted smoothly to give the corresponding products 3a–3g in excellent yields. Besides, other functional groups such as electron-donating (Me, OBn) and -withdrawing (CO_2_Me, CHO and CN) and halogen groups (F, Cl and Br) at the C-5 position of the indoles were all generally tolerated, and the corresponding products (3h–3o) were isolated in high yields. Indoles bearing a 6-substituent underwent rapid coupling with 2a in good to excellent chemical yields (up to 99% yield, 3p–3v). The structure of 3r was confirmed by X-ray crystallographic analysis.^16^ 7-Substituted indoles were also viable, affording the desired coupling products in good efficiency (3w–3y). Moreover, a pyrrole substrate was also amenable to this transformation, delivering the desired product 3z in 88% yield.

**Scheme 3:**
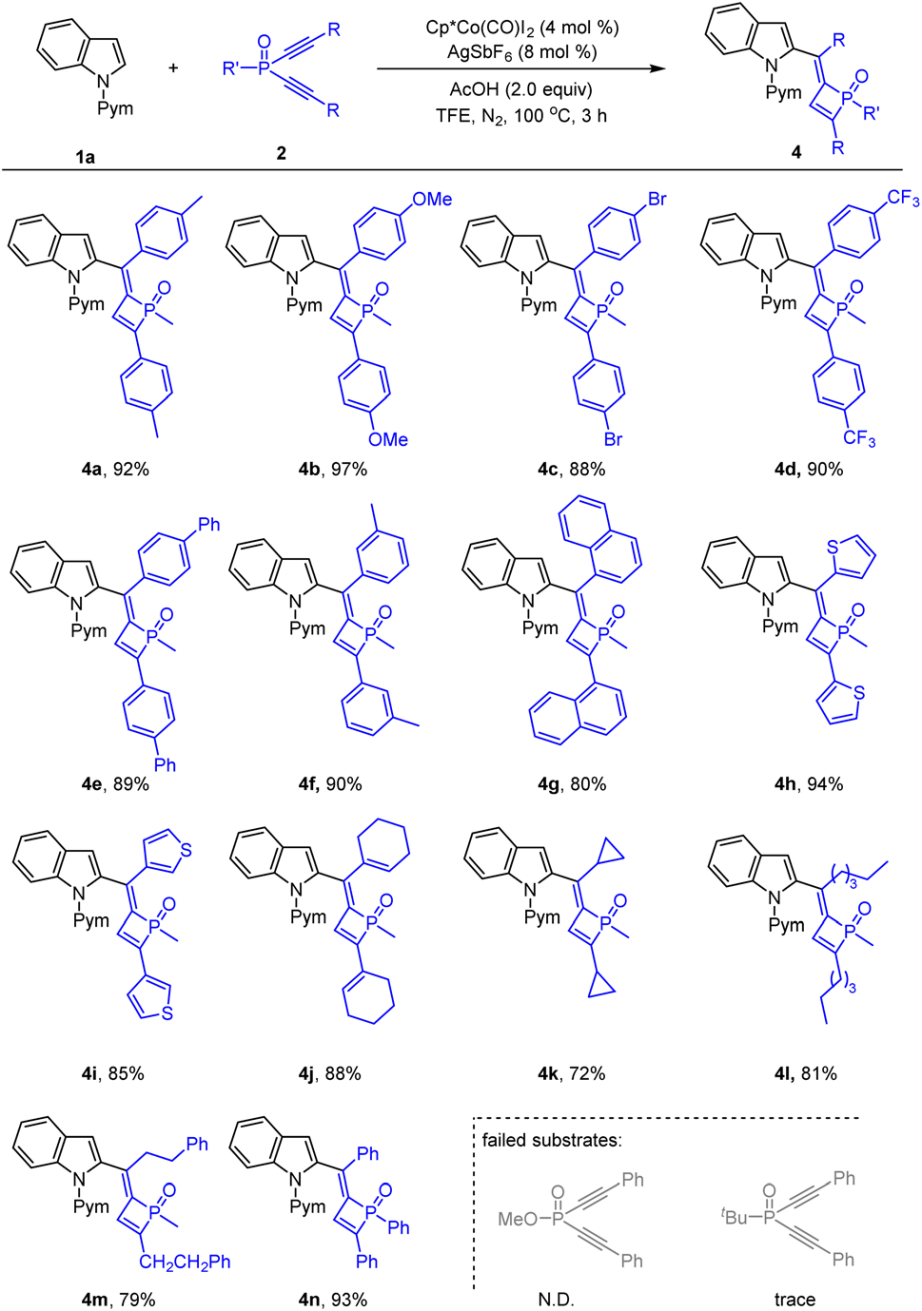
Substrate scope of dialkynylphosphine oxides by cobalt catalysis. Conditions: indoles 1a (0.26 mmol), dialkynylphosphine oxides 2 (0.20 mmol, 1.0 equiv.), Cp*Co(CO)I_2_ (4.0 mol%), AgSbF_6_ (8 mol%), and HOAc (2.0 equiv.) in TFE (2 mL) under N_2_ at 100 °C for 3 h. Isolated yields.

The scope of the dialkynylphosphine oxides was next investigated (Scheme 3). Examination of the *para*- and *meta*-substituents in the benzene ring of the diyne terminus revealed that both electron-withdrawing and -donating groups were tolerated, providing products 4a–4f in 88–97% yields. The reaction worked well when a 1-naphthyl-substituted substrate was used, delivering the product 4g in 80% yield. Besides, dialkynylphosphine oxides containing heteroaryl groups in the alkyne unit were also applicable, and 2- and 3-thienyl groups produced the desired products 4h and 4i in 94% and 85% yield, respectively. Gratifyingly, alkenyl-substituted substrates afforded satisfactory results as in the isolation of product 4j in 88% yield. Cyclic and linear alkyl substituents were all tolerated under the reaction conditions with corresponding 4k–4m being isolated in 72–81% yields. Of note, changing the *P*-methyl group diyne to a *P*-phenyl one led to the desired product 4n in high isolated yield. To our disappointment, replacing the methyl group on the phosphorus center with a methoxyl or *tert*-butyl group failed to deliver any desired product.

**Scheme 4:**
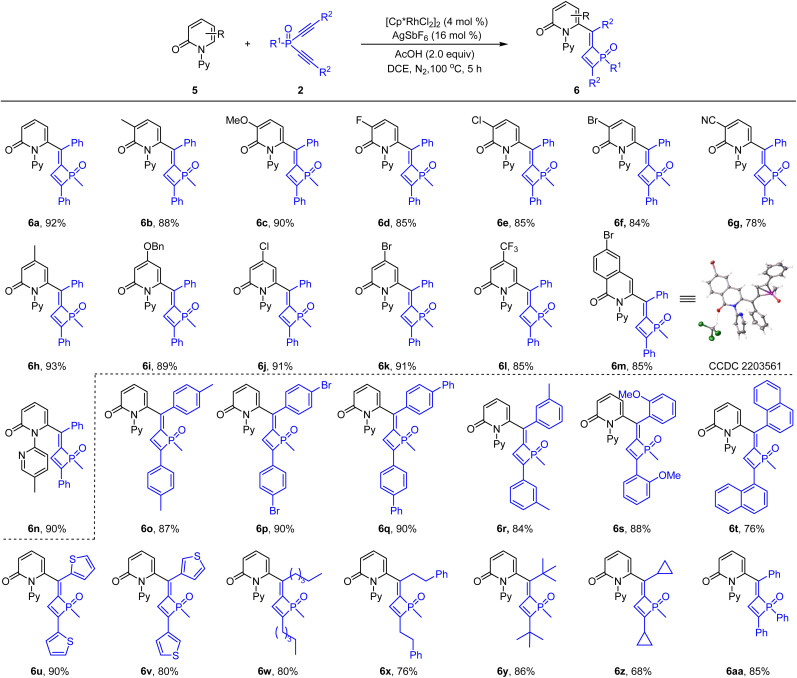
Scope of 2-pyridones and dialkynylphosphine oxides by rhodium catalysis. Conditions: 2-pyridones 5 (0.26 mmol), dialkynylphosphine oxides 2 (0.20 mmol, 1.0 equiv.), [Cp*RhCl_2_]_2_ (4.0 mol%), AgSbF_6_ (16 mol%), HOAc (2.0 equiv.) in 2.0 mL of DCE under N_2_ at 100 °C for 5 h. Isolated yields.

To further expand the scope of arenes, 2-pyridones were then examined. The coupling of 2-pyridone 5a and dialkynylphosphine oxide 2a only gave poor efficiency when using the same Cp*Co(Co)I_2_ catalyst. To our delight, when catalyzed by [Cp*RhCl_2_]_2_/AgSbF_6_ in DCE, the desired product was isolated in 92% yield (see the ESI† for details). The scope of the 2-pyridone substrate was then explored using the dialkynylphosphine oxide 2a as a coupling partner (Scheme 4). Thus, 2-pyridones bearing a series of substituents such as methyl, methoxyl, benzyloxyl, halogen, cyano and trifluoromethyl at the C3- or C4-positions were all compatible in this reaction, delivering the corresponding products in consistently high yields (6b–6l, 78–93% yield). 6-Bromo-isoquinolinone was tolerated under the reaction conditions and the coupling afforded the corresponding product 6m in 85% yield, which was confirmed by X-ray crystallography studies.^17^ 2-Pyridone bearing a 5-methyl-*N*-pyridinyl group reacted smoothly to give product 6n in 90% yield. Regarding the dialkynylphosphine oxides, our results indicated that a series of phenyl, naphthyl, heteroaryl, and alkyl groups in the alkyne were all tolerated, furnishing the corresponding 1,2-dihydrophosphete oxides in good to high yields (6o–6z, 68–90%). The tolerance of bulky ^*t*^Bu groups in 6y may provide mechanistic insights (*vide infra*).

**Fig. 2:**
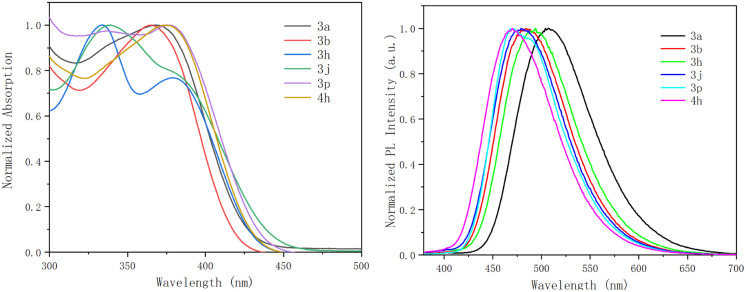
Normalized absorption (left) and emission (right) spectra of 3a, 3b, 3h, 3j, 3p, and 4h in DCM (1 × 10^−5^ M).

The photophysical properties of six products were briefly investigated (Fig. 2 and Table 2). Compounds 3a–4h displayed an intense absorption band in the UV/Vis region centered at 370 nm, attributed to the π–π* transitions of the extended π-conjugated system. Besides, these derivatives exhibited blue emissions. The fluorescent emission maxima appeared in the range of 470–504 nm (Table 2). These results may indicate their potential for applications in photoelectronics.

**Table 2:** Photophysical properties of selected products (1 × 10^−5^ M in DCM)

Compound	*λ*_abs_ a (nm)	*λ*_em_ b (nm)	*Φ*_F_ c
3a	368	504	0.0383
3b	365	484	0.0478
3h	334, 379	494	0.0282
3j	339	479	0.0305
3p	346, 375	470	0.0430
4h	374	470	0.0288

a Absorption maxima.

b Fluorescent emission maxima.

c Absolute quantum yields (determined with an integrating sphere system).

**Scheme 5:**
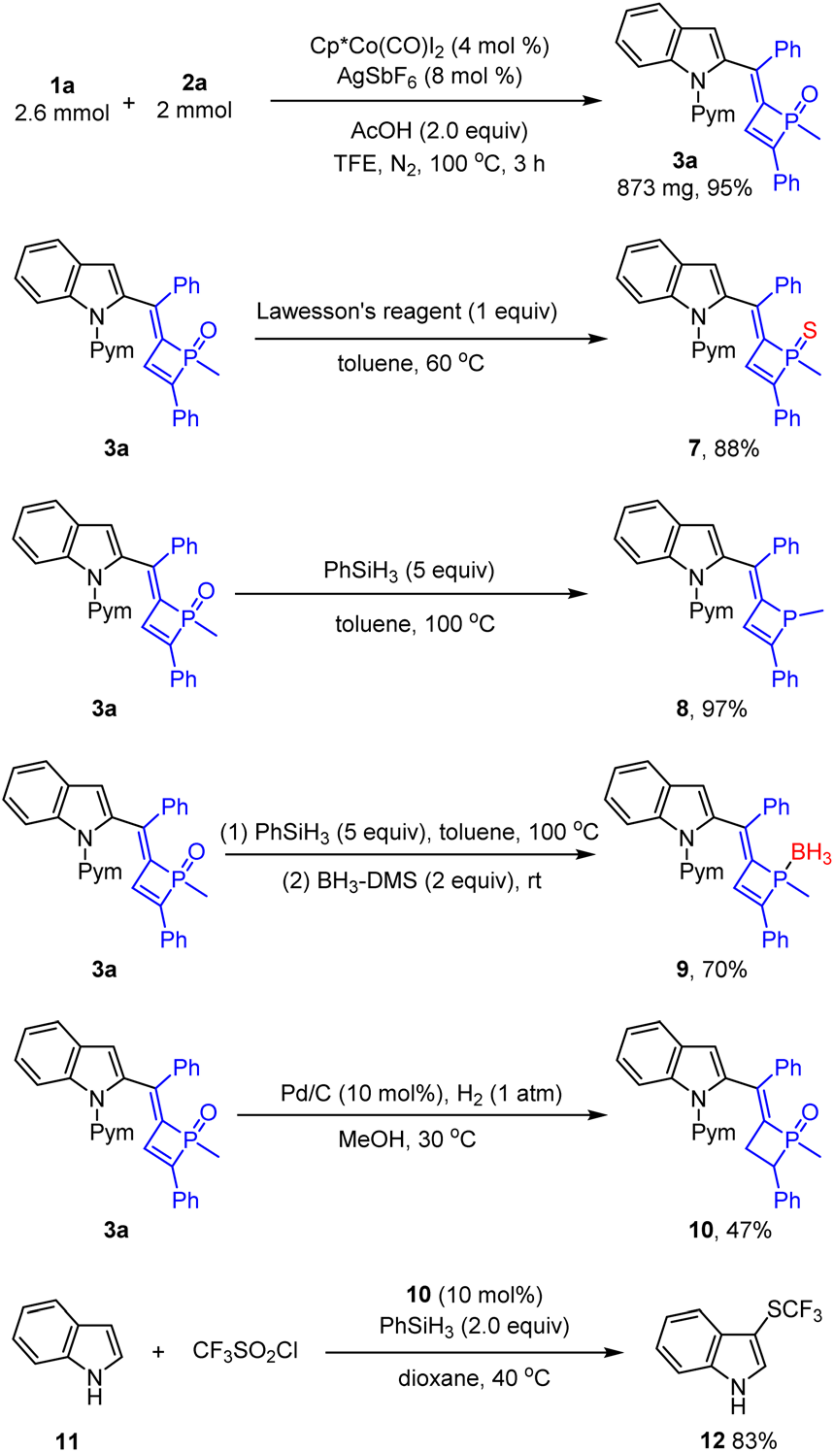
Synthetic applications of a product.

Synthetic applications of a representative product have been demonstrated (Scheme 5). The reaction of 1a and dialkynylphosphine oxide 2a was scaled up to a mmol scale, from which product 3a was isolated in 95% yield. Treatment of 3a with Lawesson's reagent afforded the phosphine sulfide 7 in high yield. The phosphine oxide 3a was reduced by PhSiH_3_ to give phosphine 8 in excellent yield. Reduction of the phosphine oxide by PhSiH_3_ followed by protection by borane gave the adduct 9 in 70% yield. Tetrahydrophosphete oxide 10 was formed in 47% yield under a palladium/H_2_ reductive system. Meanwhile, the tetrahydrophosphete oxide 10 as an organic phosphine catalyst could catalyze deoxygenation of CF_3_SO_2_Cl and the reaction with indole to afford C3-trifluoromethylsulfenylation indole 12 in 83% yield.^3*j*^

**Scheme 6:**
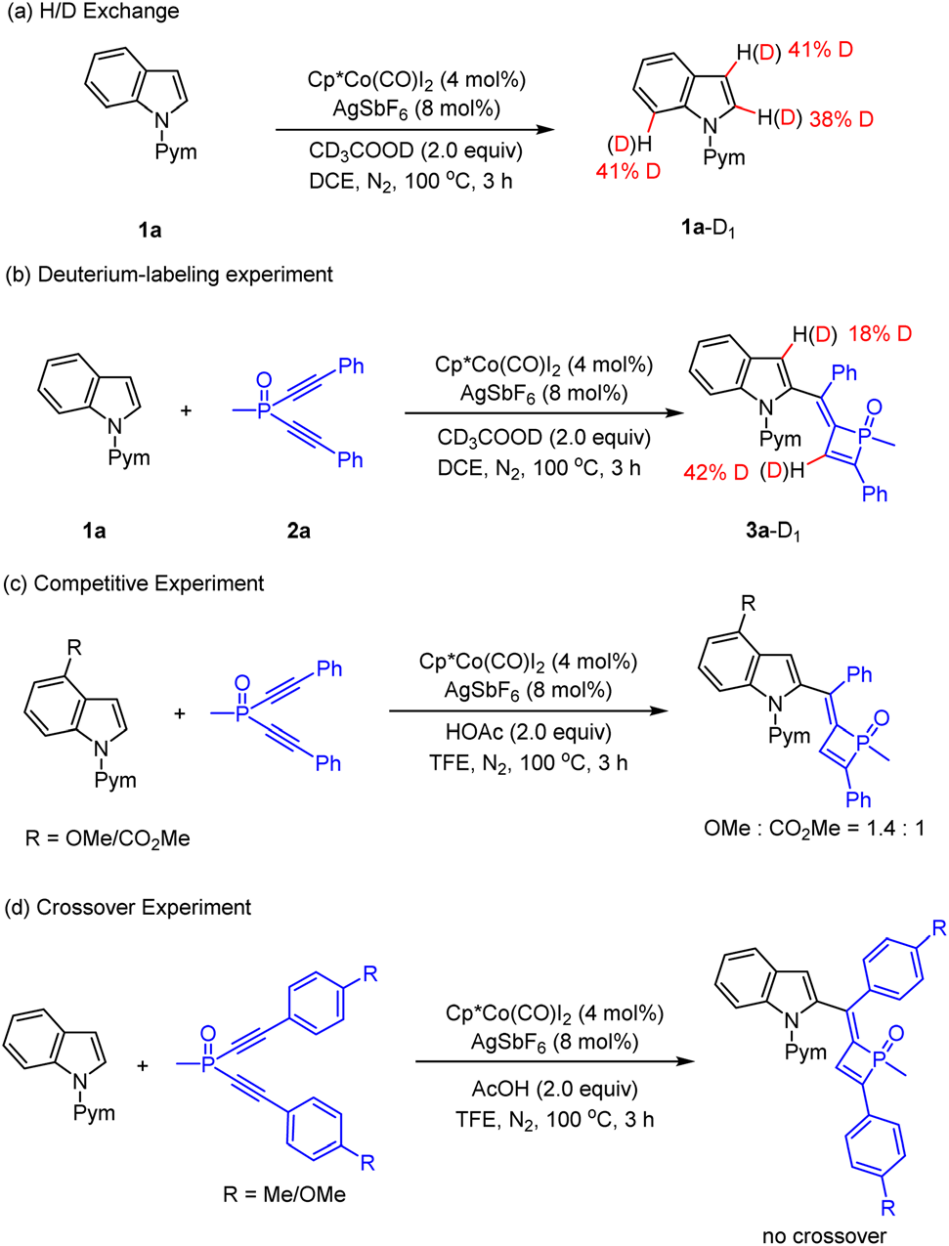
Mechanistic studies.

**Scheme 7:**
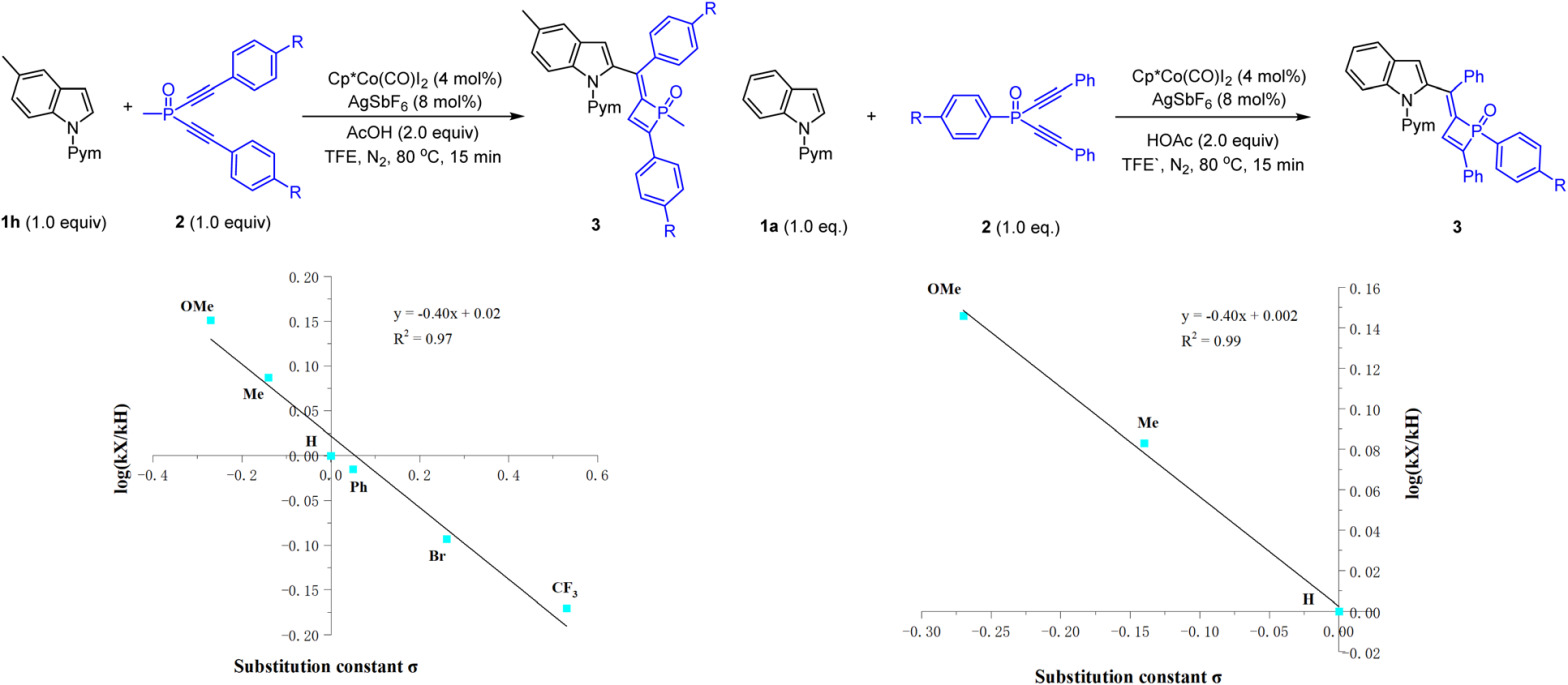
Hammett studies for the cobalt-catalyzed systems.

Preliminary mechanistic studies were conducted to gain insight into the reaction mechanism (Schemes 6 and 7). First, an H/D exchange experiment was conducted using CD_3_COOD as a deuterium source, and H/D scrambling was observed at the C2, C3, and C7 of indole 1a (Scheme 6a), supporting the reversibility of C–H activation at these positions under the reaction conditions. A deuterium labeling experiment was then performed using 1a and 2a in the presence of CD_3_COOD (Scheme 6b), and analysis of the product by ^1^H NMR spectroscopy revealed that the 3-position of the indole and olefinic C–H of dihydrophosphete ring was substantially deuterated. The significant deuteration at the olefinic C–H position likely suggests protonolysis of a Co–C bond in the catalytic cycle. Parallel competitive reactions of two electronically distinguishable indoles 1b (R = OMe) and 1d (R = CO_2_Me) have been conducted, and the more electron-rich indole tends to react with slightly higher reactivity (Scheme 6c). Next, a crossover experiment using a mixture of 2a (R = Me) and 2b (R = OMe) was then performed, and HRMS analysis of the product mixture indicated that no crossover product was present, revealing an intramolecular P=O migration process (Scheme 6d). Moreover, Hammett studies were also performed for a series of indoles with various substituents at the C5-position (see the ESI† for details) and for a series of dialkynylphosphine oxides bearing different para substituents (Scheme 7). A linear correlation was observed for each series through the Hammett plot. A negative *ρ* value (−0.40 for the dialkynylphosphine oxide series) and (−1.18 for the indole series (see the ESI† for details)) was observed for each plot, and this outcome suggests positive charge accumulation in the transition state, which is stabilized by an electron-donating substituent.

**Scheme 8:**
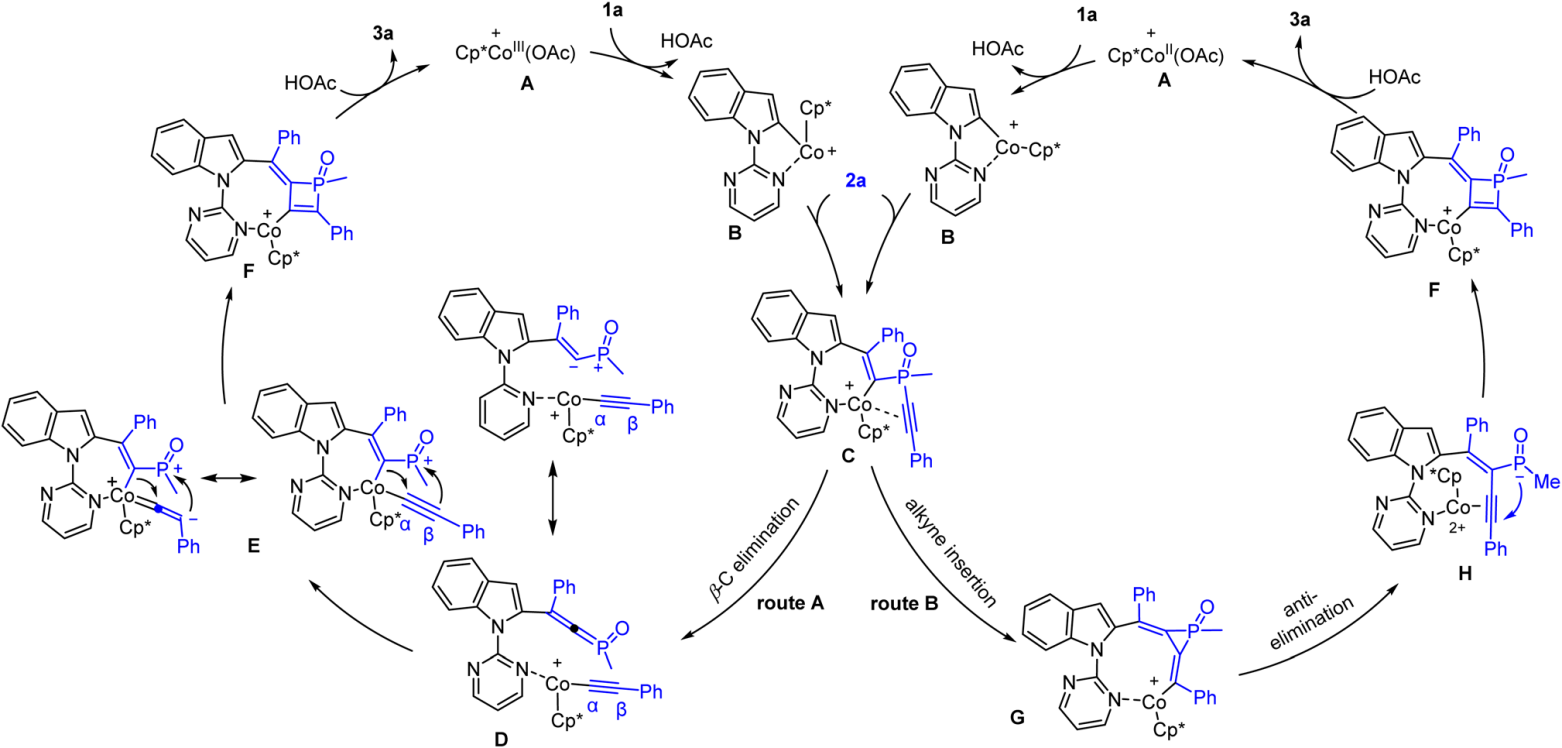
Proposed possible reaction pathways.

On the basis of these results and related literature reports of C–H activation-coupling with alkynes, a plausible mechanism for the Co(iii)-catalyzed system is proposed in Scheme 8. Starting form a Co(iii) caboxylate species, C–H activation occurs at the C(2) position to give the intermediate B*via* cyclometalation. Subsequently, the resulting Co–C(2) bond undergoes migratory insertion into an alkyne unit of the incoming diyne 2a, forming an alkenylcobalt intermediate C. At this stage, two pathways are possible. In route A (Scheme 8, left), β-C(alkynyl) elimination is proposed to give a cobalt alkynyl intermediate with a pendant phosphonium ylide (D). Coordination of the carbanion leads to an alkenyl intermediate E, and the subsequent α insertion of the Co–alkenyl bond into the vinylidene-like C(α) triggers a nucleophilic addition of the C(β) to the phosphonium center. The resulting intermediate F undergoes protonolysis to release the final product and completes the catalytic cycle. Alternatively in route B (Scheme 8, right), the intermediate C is proposed to undergo the 2nd migratory insertion into the alkyne to generate a three-membered phosphacycle (G). Anti-elimination^18^ of the phosphinate anion gives an enyne intermediate H, where the alkyne unit is activated toward 4-*endo*-dig cyclization to form the same intermediate F. While it is challenging to unequivocally distinguish between these two pathways by experimental methods, we tend to prefer the route A based on the kinetic studies. Hammett plots (Scheme 7) of both series of Co-catalyzed reactions supported the intermediacy of key cationic species that is stabilized by EDGs. Accordingly, the route A is more likely. On the other hand, the isolation of product 6y in good yields under the Rh-catalyzed conditions starting from the diyne with *tert*-butyl termini also argues against the route B. This is because the formation of the corresponding rhodium congener of intermediate G will cause strong steric repulsions between the metal center and proximal ^*t*^Bu group.

## Conclusions

In summary, a series of 1,2-dihydrophosphete oxides were efficiently synthesized through a cobalt/rhodium-catalyzed C–H alkenylation/P=O migration sequence *via* the coupling of indoles/2-pyridone with dialkynylphosphine oxides. A large array of functional groups were tolerated in this catalytic system. The photophysical properties of selected products indicate the potentiality of 1,2-dihydrophosphetes as electroluminescent materials. Mechanistic studies have been performed and Hammett studies suggest build-up of positive charges in the catalytic cycle, and the *P*-alkynyl group likely undergoes beta-elimination and migratory insertion. Asymmetric reactions of the related chemistry of diynes are underway in our laboratory.

## Data availability

Further details of the experimental procedure, ^1^H and ^13^C NMR, and X-ray crystallographic data for products 3r and 6m are available in the ESI.†

## Author contributions

X. L. conceived the idea and initiated the project. G. Z. performed the initial studies and analyzed the data. S. X. and R. M. performed the experiments. X. L. and S. X. wrote the manuscript.

## Conflicts of interest

The authors declare no competing financial interests.

## Supplementary Material

SC-015-D4SC00649F-s001

SC-015-D4SC00649F-s002
