# Supplementary material for: Cobalt- or rhodium-catalyzed synthesis of 1,2-dihydrophosphete oxides via C–H activation and formal phosphoryl migration
Source: Chem Sci. 2024 Mar 14;15(16):6012–21. doi: 10.1039/d4sc00649f (PMC11040647; doi:10.1039/d4sc00649f)
Supplement: SC-015-D4SC00649F-s001 [file SC-015-D4SC00649F-s001.pdf]

Electronic Supplementary Material (ESI) for Chemical Science.
This journal is © The Royal Society of Chemistry 2024

Supplemental Information

Cobalt or Rhodium-Catalyzed Synthesis of 1,2-Dihydrophosphete Oxides via C–H Activation and Formal Phosphoryl Migration

Shengbo Xu,[†] Ruijie Mi,[‡] Guangfan Zheng,[§] Xingwei Li*,[†,‡]

† School of Chemistry and Chemical Engineering, Shaanxi Normal University (SNNU), Xi'an 710062, P. R. China

‡ Institute of Molecular Science and Engineering, Institute of Frontier and Interdisciplinary Sciences, Shandong University, Qingdao 266237, P. R. China

§ Department of Chemistry, Northeast Normal University, Changchun 130024, P. R. China.

E-mail: lixw@snnu.edu.cn

Table of Contents

1 General Information...S3

2 Experimental Procedure..S4

3 Synthetic applications..S6

4 Photophysical properties S9

5 Mechanistic Studies...S10

6 X-Ray crystallographic data S18

7 Spectroscopic data..S21

8 NMR spectra...S57

9 References S165

1 General Information:

All chemicals were obtained from commercial sources and were used as received unless otherwise noted. All air- and moisture-sensitive manipulations were carried out with standard Schlenk techniques under nitrogen or in a glove box under argon. The ^{1}H NMR spectra were recorded on 600 MHz NMR spectrometer. The ^{13}C NMR spectra were recorded at or 150 MHz. The ^{31}P NMR spectra were recorded at 243 MHz. The ^{11}B NMR spectra were recorded at 193 MHz. Chemical shifts were expressed in parts per million (δ) downfield from the internal standard tetramethylsilane (TMS), and were reported as s (singlet), d (doublet), t (triplet), dd (doublets of doublet), dt (doublets of triplet), and m (multiplet). The residual solvent signals were used as references and the chemical shifts were converted to the TMS scale ($CDCl_3$: δ H = 7.26 ppm, δ C = 77.16 ppm). The coupling constants J were given in Hz. High resolution mass spectra (HRMS) were obtained via ESI mode by using a MicroTOF mass spectrometer. Absorption data were recorded on a SHIMADZU UV-3600 spectrophotometer. Fluorescence and quantum yield data were recorded on a PerkinElmer LS45 fluorescence spectrometer. Column chromatography was performed on silica gel (200-300 mesh) with freshly distilled ethyl acetate and petroleum ether or dichloromethane and methanol.

The *N*-pyrimidylindoles[1], 2-pyridones[2] and dialkynylphosphine oxides[3] were synthesized according to reported procedures.

2 Experimental Procedure

General procedure for the synthesis of 3 and 4

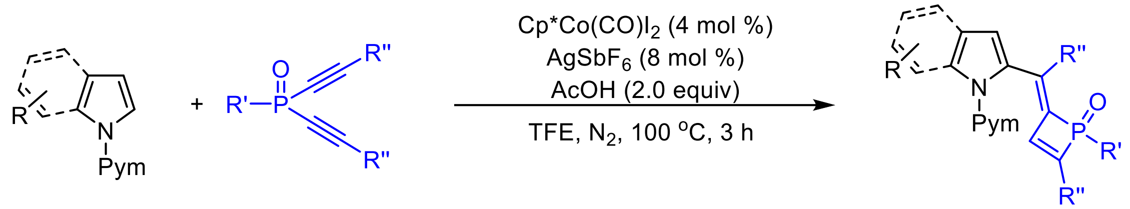

1 **2** **3 or 4**

A screw-cap vial (8 mL) was charged with **1** (0.26 mmol, 1.3 equiv), **2** (0.2 mmol, 1.0 equiv), Cp*Co(CO)I_2 (3.8 mg, 4 mol%), $AgSbF_6$ (5.5 mg, 8 mol%), HOAc (24 mg, 0.4 mmol, 2.0 equiv), in TFE (2 mL) was stirred in a vial at 100 °C for 3 h under N_2. After cooling to room temperature, the reaction mixture was evaporated under vacuum and the residue was purified by flash chromatography on silica gel (eluent: petroleum ether/ethyl acetate = 5/1, v/v) to give the corresponding **3** or **4**.

2 mmol scale synthesis of 3a

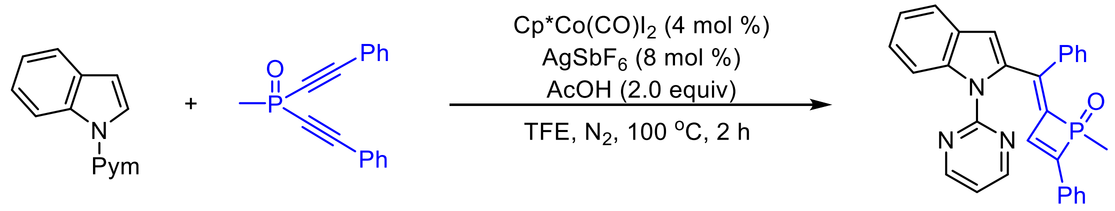

1a 2.6 mmol **2a** 2 mmol **3a** 873.1 mg 95%

To a sealable tube (50 mL) was charged with *N*-pyrimidylindole **1a** (507 mg, 2.6 mmol, 1.3 equiv), dialkynylphosphine oxide **2a** (529 mg, 0.2 mmol, 1.0 equiv), Cp*Co(CO)I_2 (38 mg, 4 mol%), $AgSbF_6$ (55 mg, 8 mol%), HOAc (240 mg, 4.0 mmol, 2.0 equiv), in TFE (20 mL) was stirred at 100 °C for 3 h under N_2. After cooling to room temperature, the resultant mixture was evaporated under reduced pressure and the residue was purified by flash column chromatography on silica gel (eluent: petroleum ether/ethyl acetate = 5/1, v/v) to give pure product **3a** 873.1 mg (95%).

Table 1. Optimization of the reaction condition for the synthesis of **6**[a]

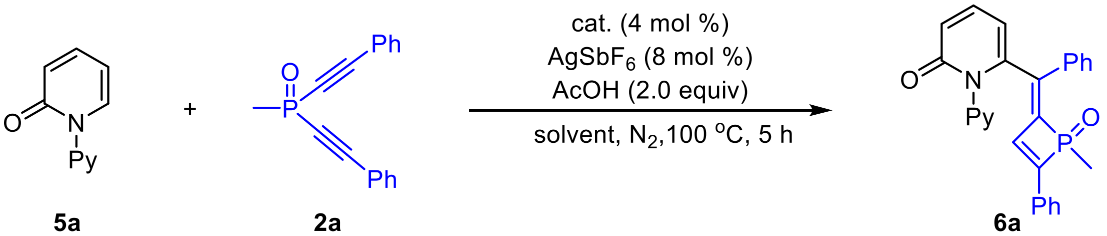

entry	cat.	solvent	yield (%)[b]
1	Cp*Co(CO)I_2	TFE	36
2	[Cp*RhCl_2]$_2$	TFE	84[c]
3	[Cp*RhCl_2]$_2$	DCE	92[c]

[a]The reactions were carried out with **5a** (0.13 mmol), **2a** (0.10 mmol), Cat. (4 mol %), $AgSbF_6$ (8 mol%) and HOAc (0.2 mmol, 2.0 equiv) in a solvent (1.0 mL) under N_2 for 5 h. [b]Isolated yields. [c]$AgSbF_6$ (16 mol%) was used.

General procedure for the synthesis of 6

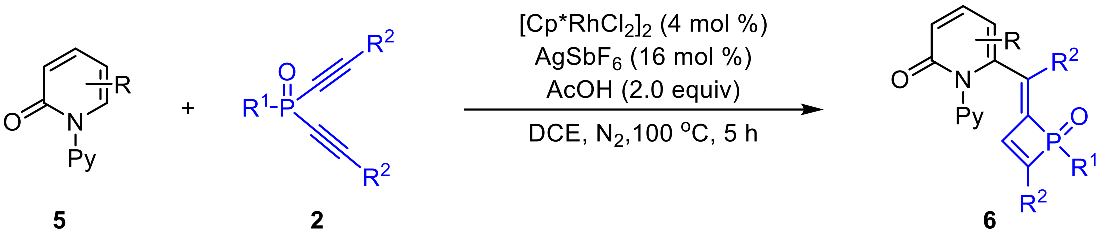

A screw-cap vial (8 mL) was charged with **5** (0.26 mmol, 1.3 equiv), **2** (0.2 mmol, 1.0 equiv), [Cp*RhCl_2]$_2$ (2.5 mg, 4 mol%), $AgSbF_6$ (11 mg, 16 mol%), HOAc (24 mg, 0.4 mmol, 2.0 equiv), in DCE (2 mL) was stirred in a vial at 100 °C for 5 h under N_2. After cooling to room temperature, the reaction mixture was evaporated under vacuum and the residue was purified by flash chromatography on silica gel (eluent: dichloromethane/methanol = 50/1, v/v) to give the corresponding **6**.

3 Synthetic applications

General procedure for the synthesis of 7

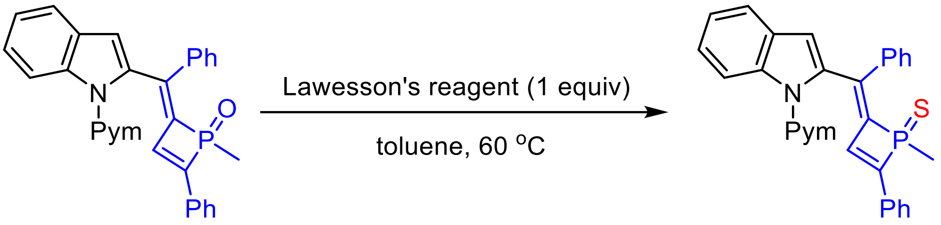

3a **7** 88%

To a solution of **3a** (45.9 mg, 0.1 mmol, 1.0 equiv) in toulene (1.0 mL) was added Lawesson’s reagent (40.4 mg, 0.1 mmol, 1.0 equiv). The solution was stirred at 60 °C for 1 h. After cooling to room temperature, the reaction was concentrated and purified by column chromatography (eluent: petroleum ether/ethyl acetate = 10/1, v/v) to afford the product **7** (41.9 mg, 88%).

General procedure for the synthesis of 8

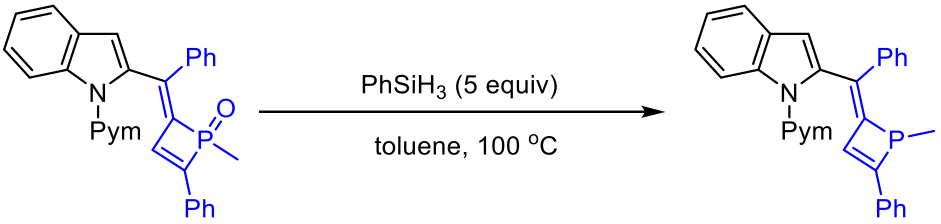

3a **8** 97%

Under N_2 atmosphere, to a solution of **3a** (45.9 mg, 0.1 mmol, 1.0 equiv) in toulene (1.0 mL) was added $PhSiH_3$ (54.1 mg, 0.5 mmol, 5.0 equiv). The solution was stirred at 100 °C for 10 h. After cooling to room temperature, the reaction was concentrated and purified by column chromatography (eluent: petroleum ether/ethyl acetate = 100/1, v/v) to afford the product **8** (43.0 mg, 97%).

General procedure for the synthesis of 9

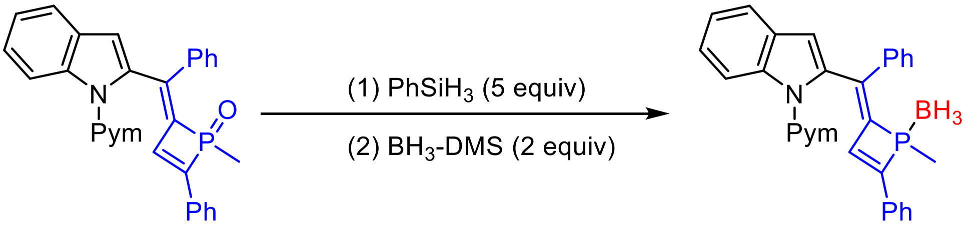

3a **9** 70%

Under N_2 atmosphere, to a solution of **3a** (45.9 mg, 0.1 mmol, 1.0 equiv) in toulene

(1.0 mL) was added $PhSiH_3$ (54.1 mg, 0.5 mmol, 1.0 equiv). The solution was stirred at 100 °C for 10 h. After cooling to room temperature, BH_3-DMS (20.0 μL, 2.0 equiv, 10.0 M solution in DMS) was added into the mixture and the resulting solution was stirred at room temperature for 2 h. Upon completion, the reaction was concentrated and purified by column chromatography (eluent: petroleum ether/ethyl acetate = 100/1, v/v) to afford the product **9** (32.0 mg, 70%).

General procedure for the synthesis of 10

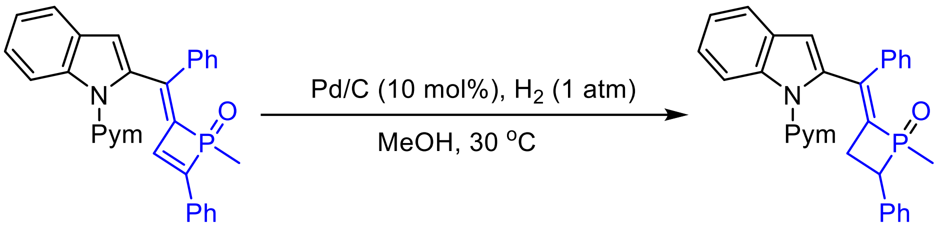

3a **10**, 47%

Pd/C (16.0 mg, palladium on activated carbon, 10% Pd basis, 0.1 equiv) was added to a solution of **3a** (230.8 mg, 0.5 mmol, 1.0 equiv) in MeOH (5.0 mL). The reaction mixture was stirred under H_2 atmosphere (1 atm) at 30 °C for 36 h. After the reaction was complete (monitored by TLC), the crude reaction mixture was filtered through celite and washed with EtOAc. The solvent was removed under reduced pressure. Then the residue was purified by silica gel column chromatography (eluent: dichloromethane/methanol = 100/1, v/v) to afford the desired product **10** (108.5 mg, 47% yield).

General procedure for the synthesis of 12

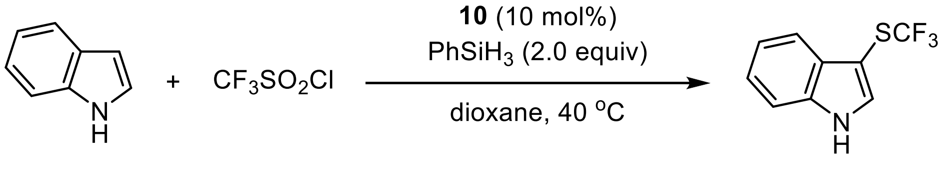

11 **12**

A screw-cap vial (4 mL) was charged with **11** (23.4 mg, 0.20 mmol, 1.0 equiv), CF_3SO_2Cl (60.7 mg, 0.36 mmol, 1.8 equiv), **10** (9.2 mg, 10 mol%), $PhSiH_3$ (43.2 mg, 0.4 mmol, 2.0 equiv), in dioxane (0.5 mL) was stirred in a vial at 40 °C for 10 h under N_2. Upon completion, the reaction mixture was evaporated under vacuum and the residue was purified by flash chromatography on silica gel (eluent: petroleum

ether/ethyl acetate = 15/1, v/v) to give the corresponding **12**.

4 Photophysical properties

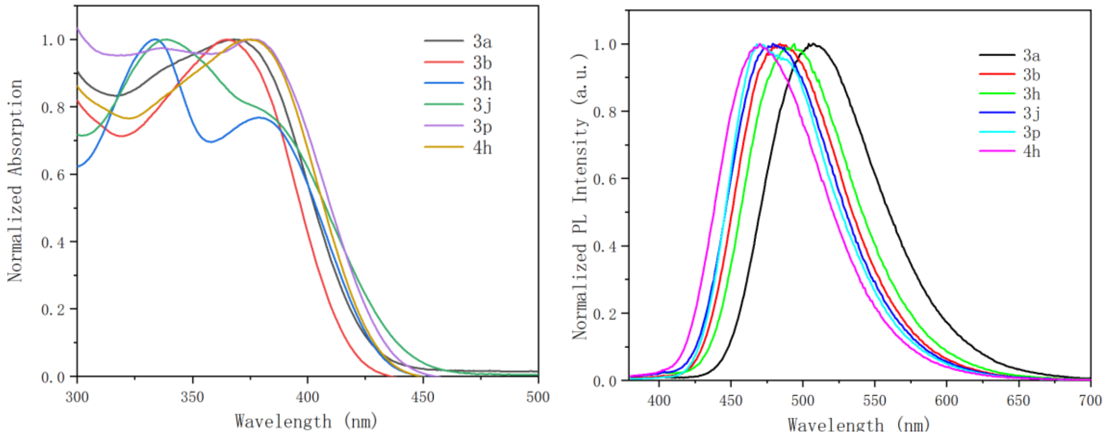

Figure 1. Normalized Absorption (left) and Emission Spectra (right) of **3a**, **3b**, **3h**, **3j**, **3p** and **4h** in DCM (1×10^{-5}M).

Table 2. Photophysical Properties of Selected Products (1×10^{-5} M in DCM).

compound	λ_{abs}^{a}(nm)	λ_{em}^{b}(nm)	Φ_F^{c}
**3a**	368	504	0.0383
**3b**	365	484	0.0478
**3h**	334, 379	494	0.0282
**3j**	339	479	0.0305
**3p**	346, 375	470	0.0430
**4h**	374	470	0.0288

[a]Absorption maxima. [b]Emission maxima. [c]Absolute quantum yields was determined with an integrating sphere system.

5 Mechanistic Studies

(a) H/D Exchange experiment

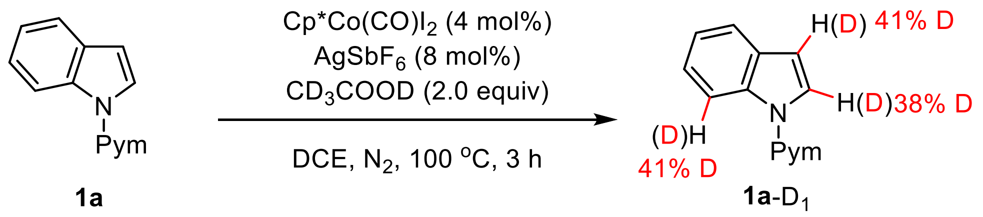

A screw-cap vial (4 mL) was charged with *N*-pyrimidylindole **1a** (0.1 mmol, 1.0 equiv), Cp*Co(CO)I_2 (1.9 mg, 4 mol%), $AgSbF_6$ (2.7 mg, 8 mol%), CD_3COOD (12.2 mg, 0.2 mmol, 2.0 equiv), in DCE (1 mL) was stirred in a vial at 100 °C for 3 h under N_2. After cooling to room temperature, the reaction mixture was evaporated under vacuum and the residue was purified by flash chromatography on silica gel (eluent: petroleum ether/ethyl acetate = 30/1, v/v) to afford the **1a**-D_1.

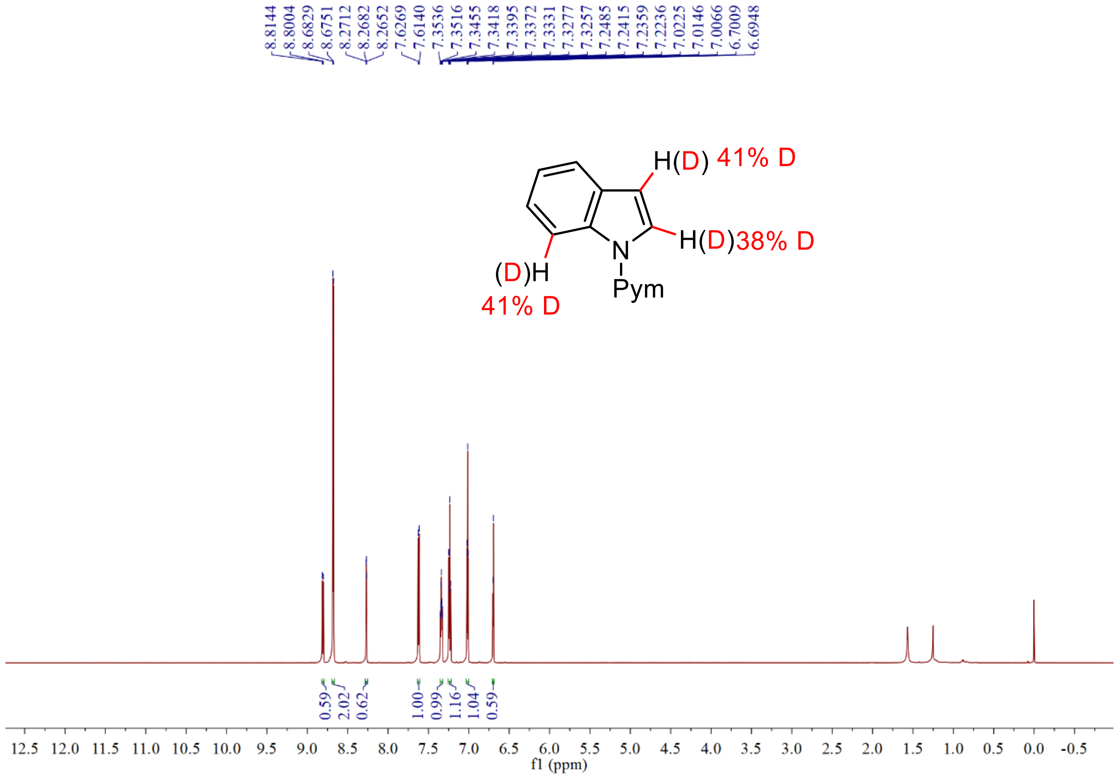

(b) Deuterium-labeling experiment

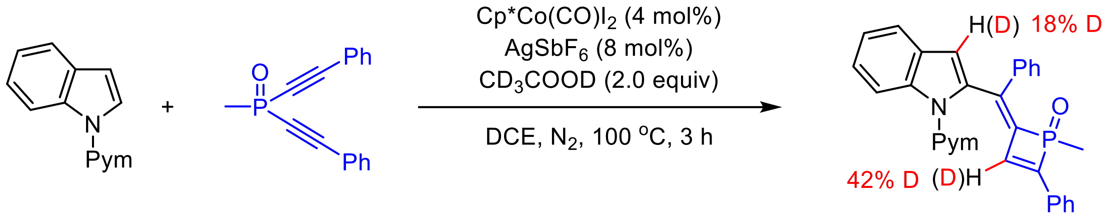

1a **2a** **3a**-D_1

A screw-cap vial (4 mL) was charged with *N*-pyrimidylindole **1a** (0.1 mmol, 1.0 equiv), dialkynylphosphine oxide **2a** (0.1 mmol, 1.0 equiv), Cp*Co(CO)I_2 (1.9 mg, 4 mol%), $AgSbF_6$ (2.7 mg, 8 mol%), CD_3COOD (12.2 mg, 0.2 mmol, 2.0 equiv), in DCE (1 mL) was stirred in a vial at 100 ºC for 3 h under N_2. After cooling to room temperature, the reaction mixture was evaporated under vacuum and the residue was purified by flash chromatography on silica gel (eluent: petroleum ether/ethyl acetate = 5/1, v/v) to afford the **3a**-D_1.

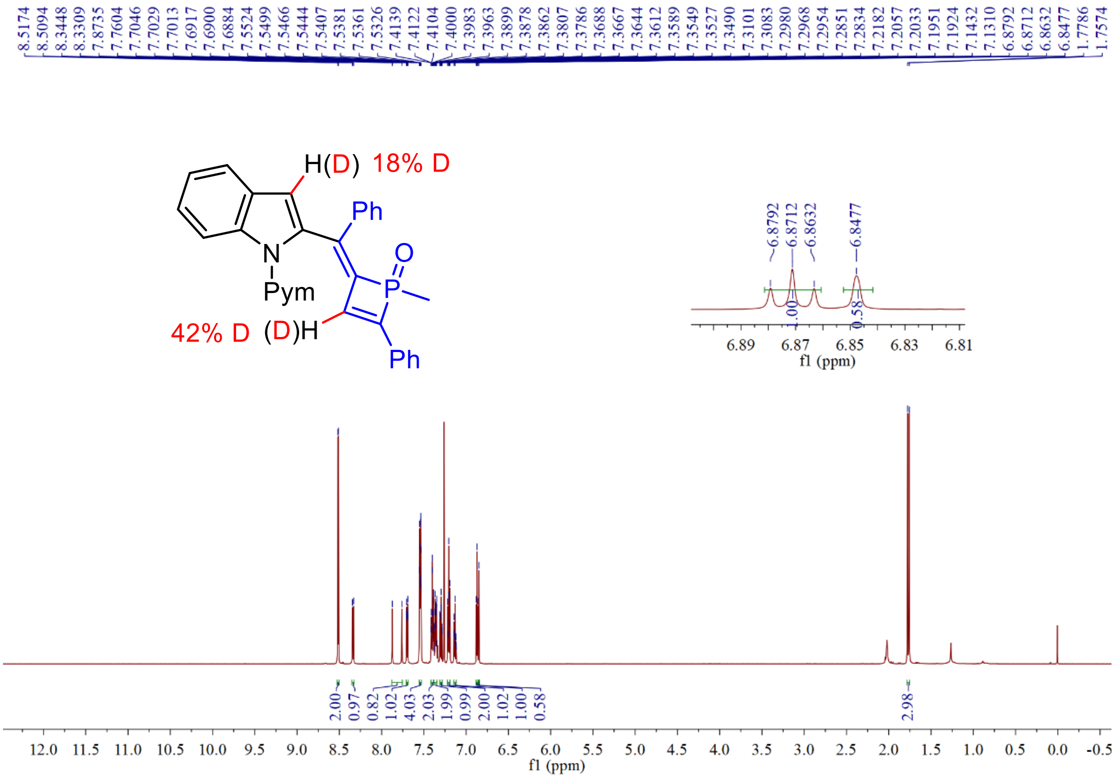

(c) Competition Reaction

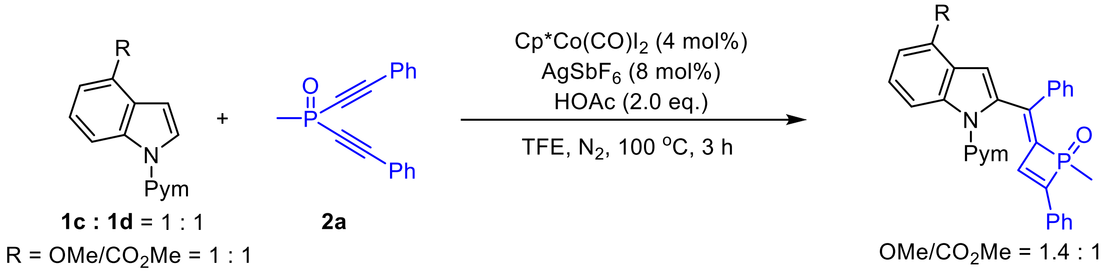

A screw-cap vial (4 mL) was charged with *N*-pyrimidylindole **1c** (0.1 mmol, 1.0 equiv), **1d** (0.1 mmol, 1.0 equiv), dialkynylphosphine oxide **2a** (0.1 mmol, 1.0 equiv), Cp*Co(CO)I_2 (1.9 mg, 4 mol%), $AgSbF_6$ (2.7 mg, 8 mol%), HOAc (12 mg, 0.2 mmol, 2.0 equiv), in TFE (2 mL) was stirred in a vial at 100 ºC for 3 h under N_2. After cooling to room temperature, the reaction mixture was evaporated under vacuum and the residue was purified by flash chromatography on silica gel (eluent: petroleum ether/ethyl acetate = 5/1, v/v) to afford the mixture products of **3c** and **3d**.

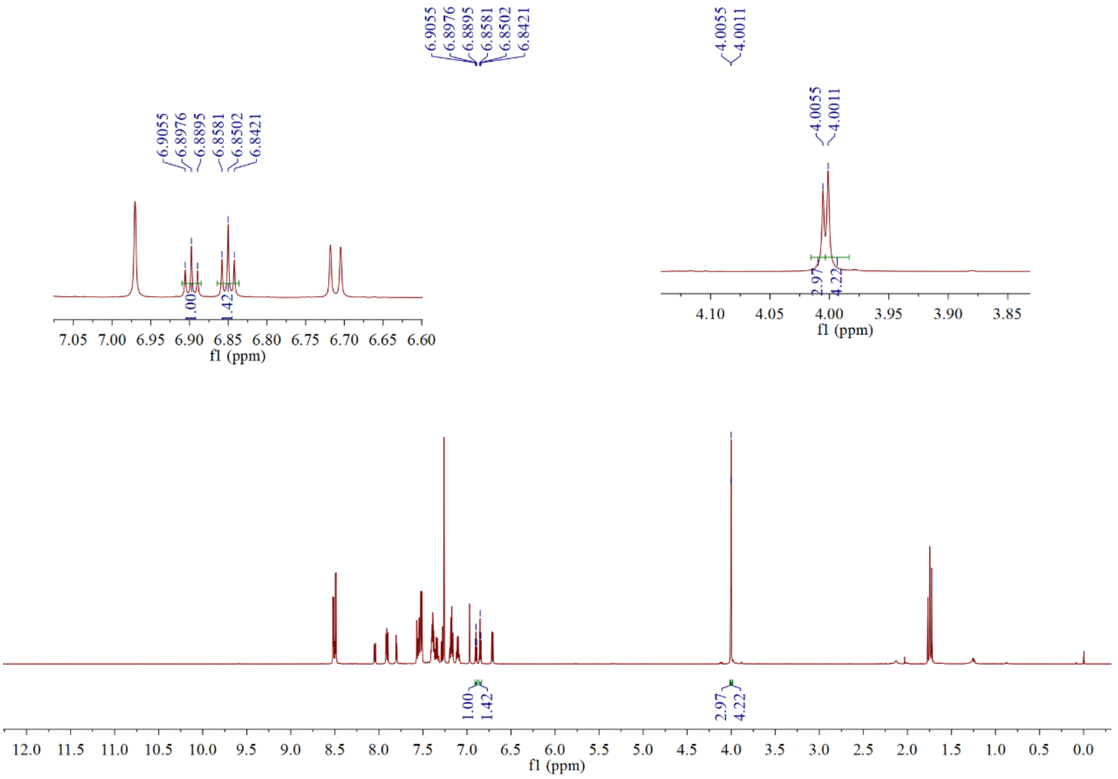

(d) Crossover experiment

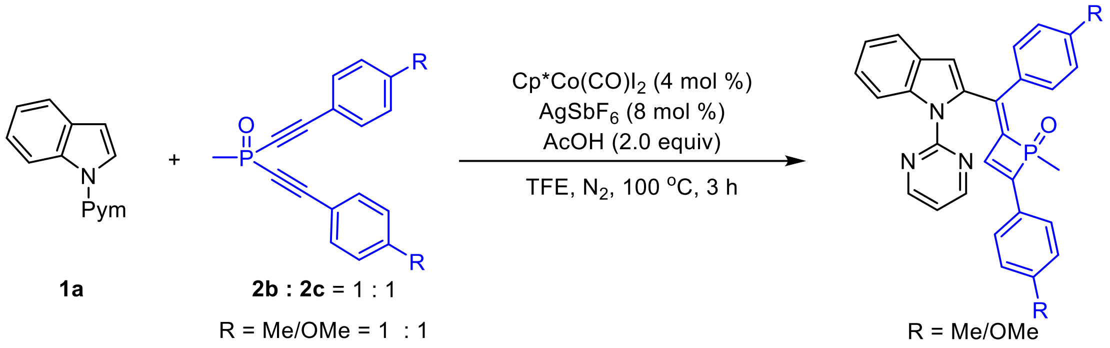

A screw-cap vial (8 mL) was charged with *N*-pyrimidylindole **1a** (0.1 mmol, 1.0 equiv), dialkynylphosphine oxides **2b** (0.1 mmol, 1.0 equiv) and **2c** (0.1 mmol, 1.0 equiv), Cp*Co(CO)I$_2$ (1.9 mg, 4 mol%), AgSbF$_6$ (2.7 mg, 8 mol%), HOAc (12 mg, 0.2 mmol, 2.0 equiv), in TFE (3 mL) was stirred in a vial at 100 °C for 3 h under N$_2$. The reaction mixture was evaporated under vacuum and the residue was purified by flash chromatography on silica gel (eluent: petroleum ether/ethyl acetate = 5/1, v/v) to afford the mixture products of **4a** and **4b**.

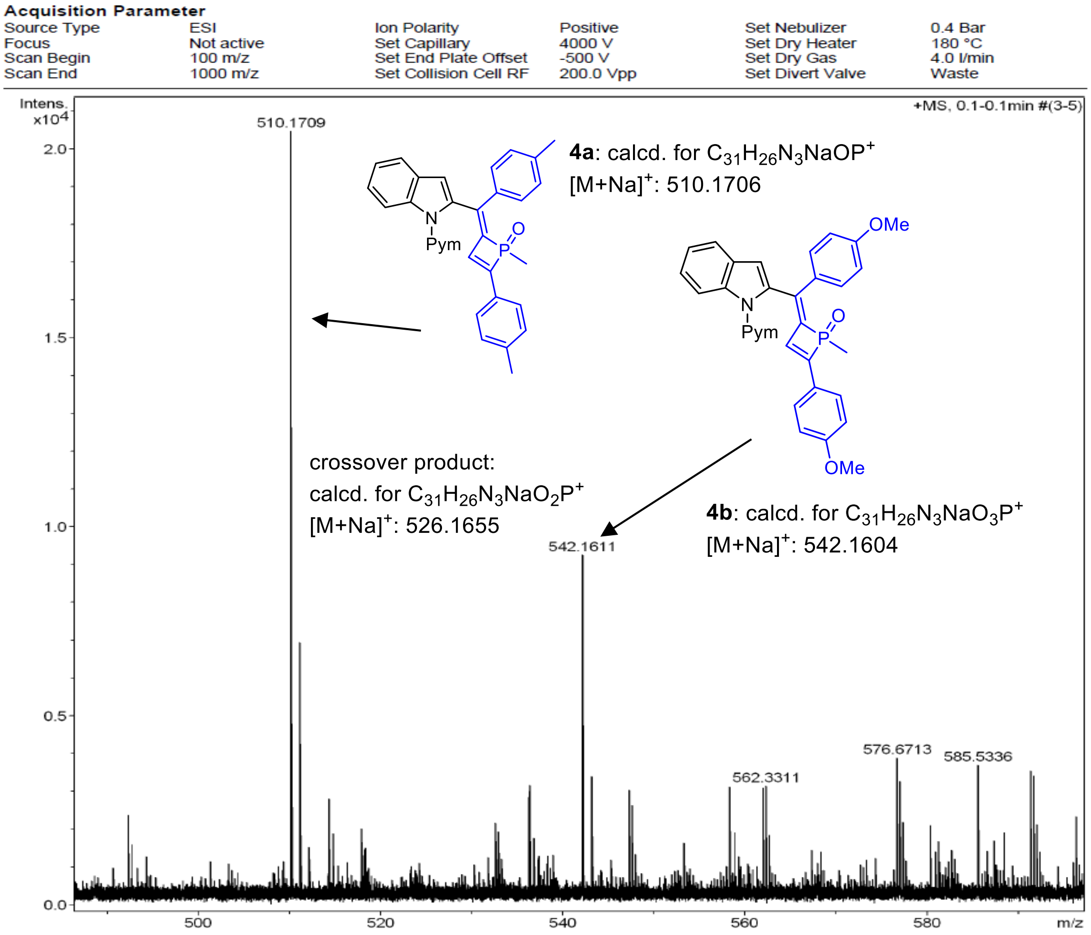

(e) Hammett plots of dialkynylphosphine oxides

A screw-cap vial (4 mL) was charged with *N*-pyrimidylindole **1h** (0.1 mmol, 1.0 equiv), dialkynylphosphine oxide **2** (0.1 mmol, 1.0 equiv), Cp*Co(CO)I_2 (1.9 mg, 4 mol%), $AgSbF_6$ (2.7 mg, 8 mol%), HOAc (12 mg, 0.2 mmol, 2.0 equiv), in TFE (1 mL) was stirred in a vial at 80 °C for 15 min under N_2. After rapid cooling with ice water, the reaction mixture was evaporated under vacuum, the initial reaction rates were determined by ^{1}H NMR with 1,3,5-trimethoxybenzene as an internal standard.

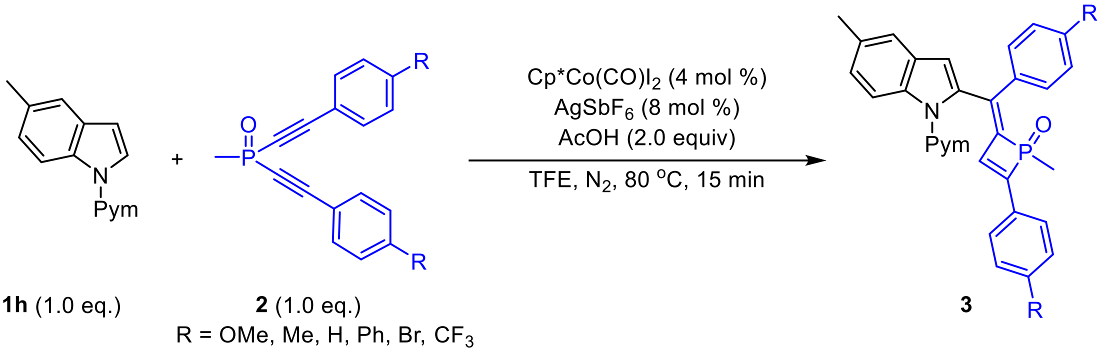

Table 3. Hammett plots of dialkynylphosphine oxides

R	σ	Initial Reaction Rate (M/min)
OMe	-0.27	2.93×10^{-3}
Me	-0.14	2.53×10^{-3}
H	0	2.07×10^{-3}
Ph	0.05	2.00×10^{-3}
Br	0.26	1.67×10^{-3}
CF_3	0.53	1.40×10^{-3}

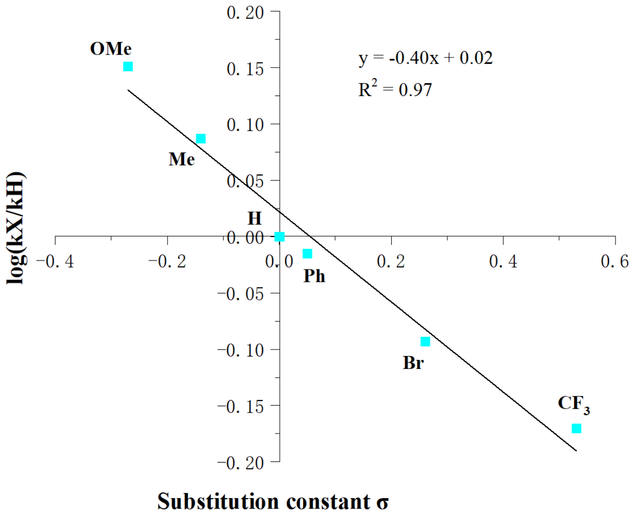

A screw-cap vial (4 mL) was charged with *N*-pyrimidylindole **1a** (0.1 mmol, 1.0 equiv), dialkynylphosphine oxide **2** (0.1 mmol, 1.0 equiv), Cp*Co(CO)I_2 (1.9 mg, 4 mol%), $AgSbF_6$ (2.7 mg, 8 mol%), HOAc (12 mg, 0.2 mmol, 2.0 equiv), in TFE (1 mL) was stirred in a vial at 80 °C for 15 min under N_2. After rapid cooling with ice water, the reaction mixture was evaporated under vacuum, the initial reaction rates were determined by ^{1}H NMR with 1,3,5-trimethoxybenzene as an internal standard.

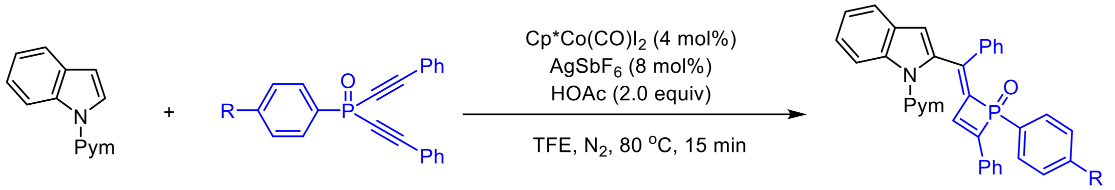

1a (1.0 eq.)

2 (1.0 eq.)
R = H, OMe, Me

3

Table 4. Hammett plots of dialkynylphosphine oxides

R	σ	Initial Reaction Rate (M/min)
OMe	-0.27	1.40 × 10^{-3}
Me	-0.14	1.21 × 10^{-3}
H	0	1.0 × 10^{-3}

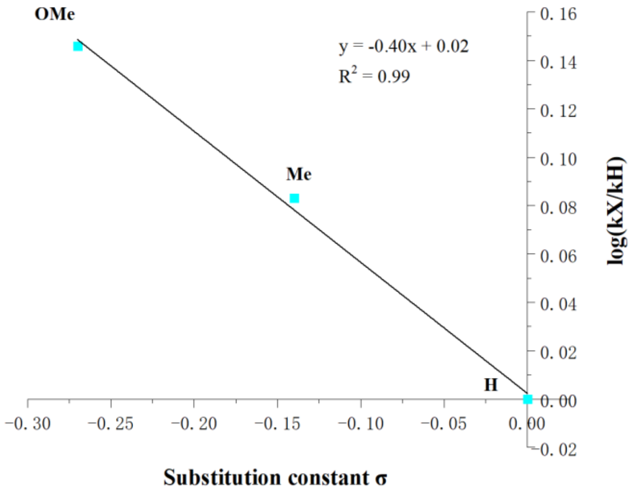

(f) Hammett plots of indoles

A screw-cap vial (4 mL) was charged with *N*-pyrimidylindole **1** (0.1 mmol, 1.0 equiv), dialkynylphosphine oxide **2b** (0.1 mmol, 1.0 equiv), Cp*Co(CO)I_2 (1.9 mg, 4 mol%), $AgSbF_6$ (2.7 mg, 8 mol%), HOAc (12 mg, 0.2 mmol, 2.0 equiv), in TFE (1 mL) was stirred in a vial at 80 °C for 15 min under N_2. After rapid cooling with ice water, the reaction mixture was evaporated under vacuum, the initial reaction rates were

determined by ^{1}H NMR with 1,3,5-trimethoxybenzene as an internal standard.

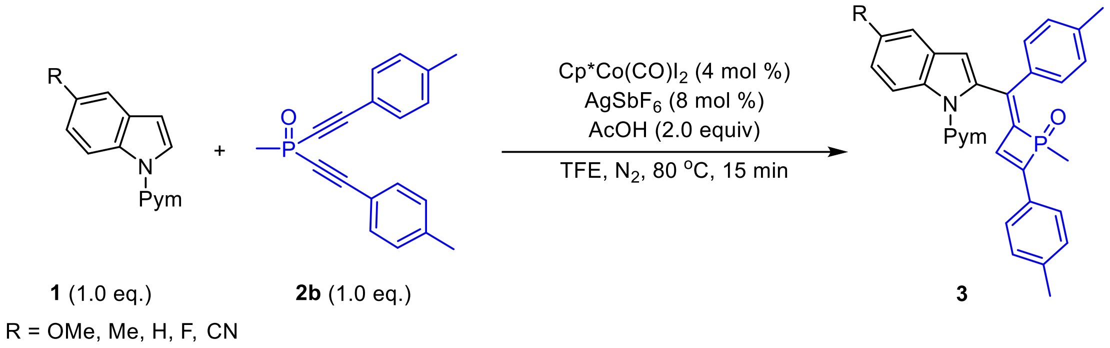

Table 5. Hammett plots of dialkynylphosphine oxides

R	σ_p	Initial Reaction Rate (M/min)
OMe	-0.27	2.4×10^{-3}
Me	-0.14	1.64×10^{-3}
H	0	1.35×10^{-3}
F	0.05	1.28×10^{-3}
CN	0.66	1.87×10^{-4}

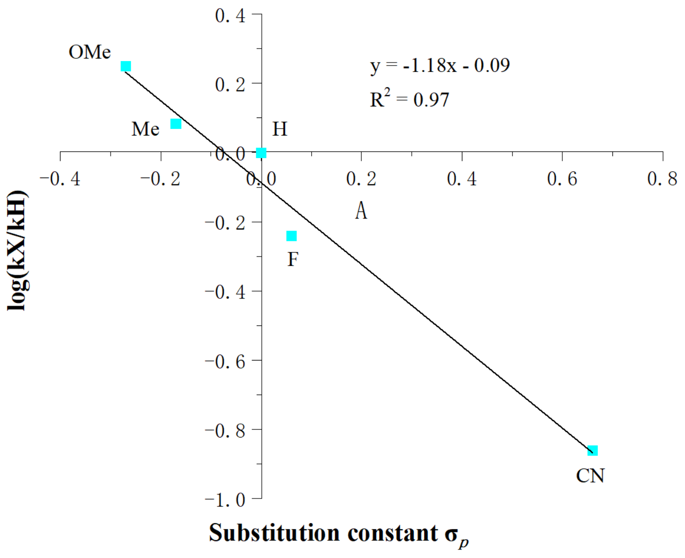

R	σ_m	Initial Reaction Rate (M/min)
OMe	0.12	2.4×10^{-3}
Me	-0.07	1.64×10^{-3}
H	0	1.35×10^{-3}
F	0.34	1.28×10^{-3}
CN	0.56	1.87×10^{-4}

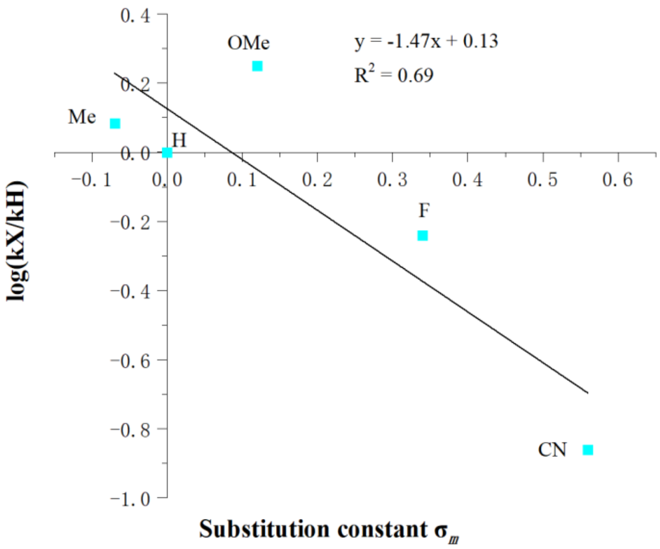

y = -1.47x + 0.13
R^2 = 0.69
OMe
Me
H
F
CN
log(kX/kH)
Substitution constant σ_m

6 X-Ray crystallographic data

(1) X-ray crystal structures of 3r (CCDC 2203562) and 6m (CCDC 2203561)

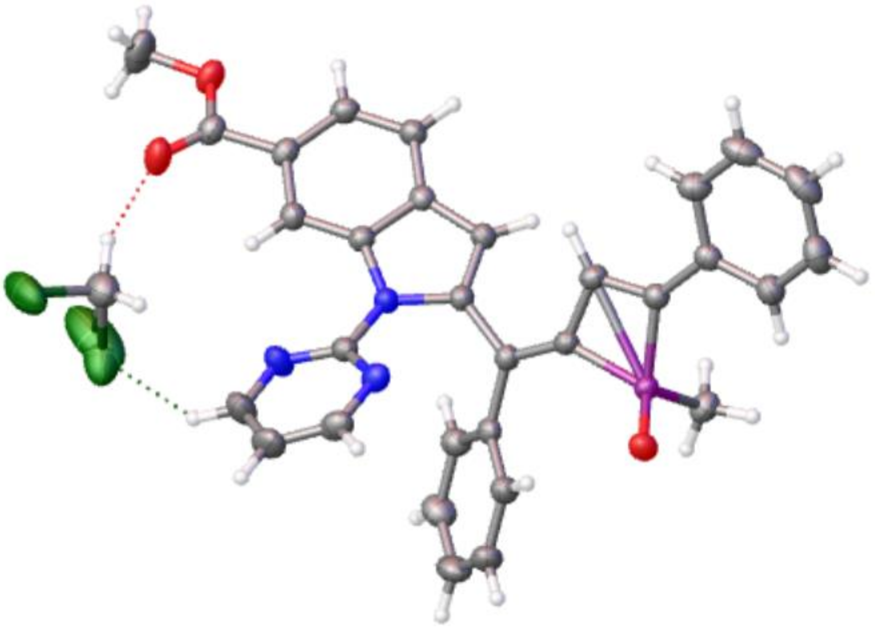

Table 6. Crystal data and structure refinement for **3r**.

Identification code	**3r**	
Empirical formula	C32 H26 Cl2 N3 O3 P	
Formula weight	602.43	
Temperature	193(2) K	
Wavelength	0.71073 Å	
Crystal system	Triclinic	
Space group	P -1	
Unit cell dimensions	a = 9.3078(2) Å	a= 83.5890(10)°.
	b = 12.9452(3) Å	b= 79.8970(10)°.
	c = 13.3909(3) Å	g = 69.5020(10)°.
Volume	1485.69(6) $Å^3$	
Z	2	
Density (calculated)	1.347 Mg/m^3	
Absorption coefficient	0.310 mm^{-1}	
F(000)	624	

Crystal size	0.150 x 0.120 x 0.080 mm^3
Theta range for data collection	2.347 to 25.500°.
Index ranges	-11<=h<=11, -15<=k<=15, -15<=l<=16
Reflections collected	21981
Independent reflections	5519 [R(int) = 0.0612]
Completeness to theta = 25.242°	99.7 %
Absorption correction	Semi-empirical from equivalents
Max. and min. transmission	0.7456 and 0.4940
Refinement method	Full-matrix least-squares on F^2
Data / restraints / parameters	5519 / 0 / 394
Goodness-of-fit on F^2	1.046
Final R indices [I>2sigma(I)]	R1 = 0.0459, wR2 = 0.1132
R indices (all data)	R1 = 0.0553, wR2 = 0.1222
Extinction coefficient	0.021(3)
Largest diff. peak and hole	0.472 and -0.532 e.Å^{-3}

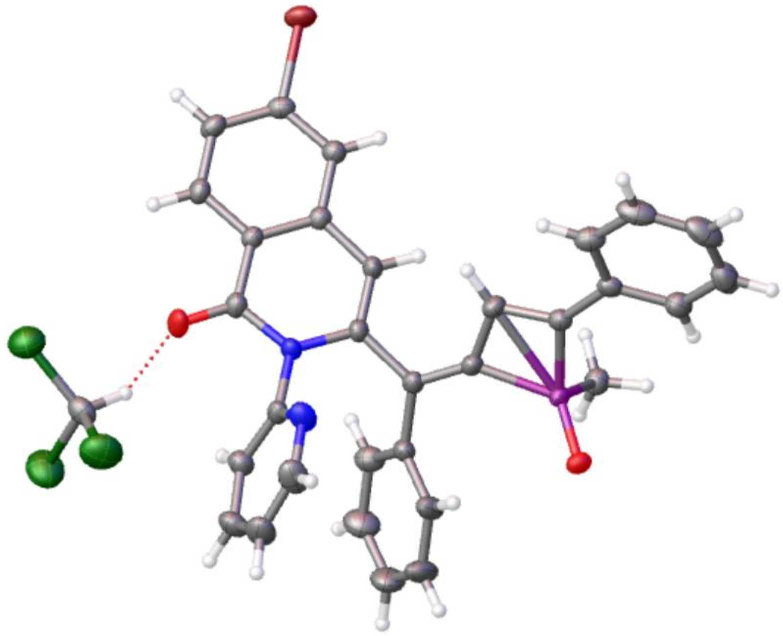

Table 7. Crystal data and structure refinement for **6m**.

Identification code	**6m**
Empirical formula	C32 H23 Br Cl3 N2 O2 P
Formula weight	684.75

Temperature	193(2) K
Wavelength	0.71073 Å
Crystal system	Triclinic
Space group	P -1
Unit cell dimensions	a = 9.3848(2) Å a= 79.7470(10)°.
	b = 11.5366(3) Å b= 83.5210(10)°.
	c = 14.8861(4) Å g = 72.5000(10)°.
Volume	1509.54(7) Å^3
Z	2
Density (calculated)	1.507 Mg/m^3
Absorption coefficient	1.713 mm^{-1}
F(000)	692
Crystal size	0.160 x 0.140 x 0.100 mm^3
Theta range for data collection	2.498 to 25.999°.
Index ranges	-11<=h<=11, -14<=k<=14, -17<=l<=18
Reflections collected	22100
Independent reflections	5895 [R(int) = 0.0331]
Completeness to theta = 25.242°	99.3 %
Absorption correction	Semi-empirical from equivalents
Max. and min. transmission	0.7456 and 0.5557
Refinement method	Full-matrix least-squares on F^2
Data / restraints / parameters	5895 / 0 / 376
Goodness-of-fit on F^2	1.022
Final R indices [I>2sigma(I)]	R1 = 0.0292, wR2 = 0.0758
R indices (all data)	R1 = 0.0351, wR2 = 0.0798
Extinction coefficient	0.0103(14)
Largest diff. peak and hole	0.394 and -0.309 e.Å^{-3}

7 Spectroscopic data

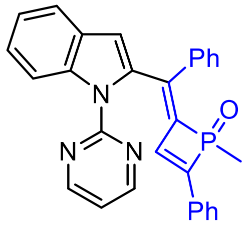

(*E*)-1-methyl-4-phenyl-2-(phenyl(1-(pyrimidin-2-yl)-1*H*-indol-2-yl)methylene)-2*H*-phosphete 1-oxide (3a)

The title compound was isolated as a yellow solid (eluent: petroleum ether/ethyl acetate = 5/1, 88.2 mg, 96%). **^{1}H NMR (600 MHz, $CDCl_3$)** δ 8.51 (d, *J* = 4.8 Hz, 2H), 8.34 (d, *J* = 8.4 Hz, 1H), 7.82 (d, *J* = 67.8 Hz, 1H), 7.70 (d, *J* = 7.9 Hz, 1H), 7.55 – 7.53 (m, 4H), 7.40 (t, *J* = 7.7 Hz, 2H), 7.38 – 7.34 (m, 2H), 7.31 – 7.28 (m, 1H), 7.22 – 7.19 (m, 2H), 7.15 – 7.11 (m, 1H), 6.87 (t, *J* = 4.8 Hz, 1H), 6.85 (s, 1H), 1.77 (d, *J* = 12.7 Hz, 3H); **^{13}C NMR (150 MHz, $CDCl_3$)** δ 157.9, 157.1, 155.8 (d, *J* = 78.0 Hz), 143.1 (d, *J* = 76.5 Hz), 141.9 (d, *J* = 13.5 Hz), 138.9 (d, *J* = 7.5 Hz), 137.1, 136.3 (d, *J* = 19.5 Hz), 133.2, 131.4 (d, *J* = 6.0 Hz), 129.8, 129.1, 128.8, 128.5, 128.1, 127.8, 128.0 (d, *J* = 9.0 Hz), 124.4, 122.4, 120.8, 117.1, 114.0, 111.6, 16.3 (d, *J* = 55.5 Hz); **^{31}P NMR (243 MHz, $CDCl_3$)** δ 42.4; **HRMS (ESI) :** calcd. for $C_{29}H_{22}N_3NaOP^+$ $[M+Na]^+$: 482.1393; found : 482.1393.

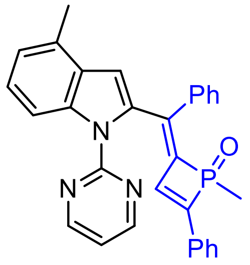

(*E*)-1-methyl-2-((4-methyl-1-(pyrimidin-2-yl)-1*H*-indol-2-yl)(phenyl)methylene)-4-phenyl-2*H*-phosphete 1-oxide (3b)

The title compound was isolated as a yellow solid (eluent: petroleum ether/ethyl acetate = 5/1, 89.0 mg, 94%). **^{1}H NMR (600 MHz, $CDCl_3$)** δ 8.52 (d, *J* = 4.8 Hz, 2H), 8.16 (d, *J* = 8.3 Hz, 1H), 7.81 (d, *J* = 67.9 Hz, 1H), 7.56 – 7.53 (m, 4H), 7.42 – 7.39 (m, 2H), 7.37 – 7.34 (m, 1H), 7.28 (d, *J* = 7.5 Hz, 1H), 7.23 – 7.20 (m, 2H), 7.16 – 7.13 (m, 1H), 7.10 (dt, *J* = 7.2, 0.9 Hz, 1H), 6.88 (t, *J* = 4.8 Hz, 1H), 6.86 (s, 1H), 2.63 (s, 3H), 1.77 (d, *J* = 12.7 Hz, 3H); **^{13}C NMR (150 MHz, $CDCl_3$)** δ 157.9, 157.2, 155.7 (d, *J* = 78.0 Hz), 142.9 (d, *J* = 75.0 Hz), 141.9 (d, *J* = 12.0 Hz), 139.0 (d, *J* = 7.5 Hz), 136.9, 135.8 (d, *J* = 19.5 Hz), 133.4, 131.4 (d, *J* = 6.0 Hz), 130.3, 129.8, 129.1, 128.5, 128.4, 128.1, 127.9, 127.0 (d, *J* = 9.0 Hz), 124.5, 122.7, 117.1, 111.5, 110.0, 18.7, 16.3 (d, *J* = 54.0 Hz); **^{31}P NMR (243 MHz, $CDCl_3$)** δ 42.4; **HRMS (ESI) :** calcd. for $C_{30}H_{24}N_3NaOP^+$ $[M+Na]^+$: 496.1549; found : 496.1546.

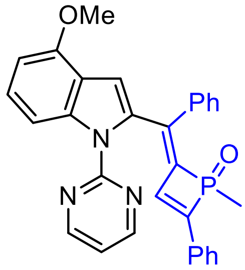

(E)-2-((4-methoxy-1-(pyrimidin-2-yl)-1H-indol-2-yl)(phenyl)methylene)-1-methyl-4-phenyl-2H-phosphete 1-oxide (3c)

The title compound was isolated as a yellow solid (eluent: petroleum ether/ethyl acetate = 5/1, 89.1 mg, 91%). **^{1}H NMR (600 MHz, CDCl$_3$)** δ 8.50 (d, *J* = 4.8 Hz, 2H), 7.93 (s, 0.5H), 7.90 (d, *J* = 8.3 Hz, 1H), 7.82 (s, 0.5H), 7.56 – 7.53 (m, 2H), 7.53 – 7.50 (m, 2H), 7.40 (t, *J* = 7.6 Hz, 2H), 7.36 – 7.33 (m, 1H), 7.29 (t, *J* = 8.2 Hz, 1H), 7.20 – 7.16 (m, 2H), 7.12 – 7.09 (m, 1H), 6.97 (s, 1H), 6.86 (t, *J* = 4.8 Hz, 1H), 6.72 (d, *J* = 7.9 Hz, 1H), 4.01 (s, 3H), 1.74 (d, *J* = 12.7 Hz, 3H); **^{13}C NMR (150 MHz, CDCl$_3$)** δ 157.9, 157.2, 155.6 (d, *J* = 78.0 Hz), 153.0, 143.0 (d, *J* = 75.0 Hz), 142.0 (d, *J* = 12.0 Hz), 139.0 (d, *J* = 7.5 Hz), 138.4, 134.8 (d, *J* = 19.5 Hz), 133.2, 131.5 (d, *J* = 6.0 Hz), 129.7, 129.1, 128.4, 128.0, 127.8, 127.0 (d, *J* = 9.0 Hz), 125.3, 119.3, 117.1, 108.7, 107.1, 102.3, 55.5, 16.3 (d, *J* = 55.5 Hz); **^{31}P NMR (243 MHz, CDCl$_3$)** δ 42.4; **HRMS (ESI) :** calcd. for $C_{30}H_{24}N_3NaO_2P^+$ $[M+Na]^+$: 512.1498; found : 512.1495.

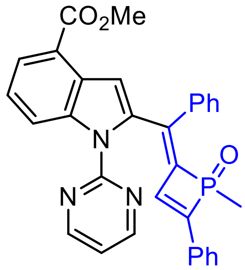

Methyl (*E*)-2-((1-methyl-1-oxido-4-phenyl-2*H*-phosphet-2-ylidene)(phenyl)methyl)-1-(pyrimidin-2-yl)-1*H*-indole-4-carboxylate (3d)

The title compound was isolated as a yellow solid (eluent: petroleum ether/ethyl acetate = 5/1, 93.2 mg, 90%). **^{1}H NMR (600 MHz, CDCl$_3$)** δ 8.53 (d, *J* = 4.8 Hz, 2H), 8.51 (d, *J* = 8.3 Hz, 1H), 8.05 (d, *J* = 7.5 Hz, 1H), 7.86 (d, *J* = 67.8 Hz, 1H), 7.56 (d, *J* = 6.6 Hz, 3H), 7.51 (d, *J* = 7.6 Hz, 2H), 7.42 – 7.35 (m, 4H), 7.19 (t, *J* = 7.5 Hz, 2H), 7.11 (t, *J* = 7.3 Hz, 1H), 6.91 (t, *J* = 4.8 Hz, 1H), 4.01 (s, 3H), 1.76 (d, *J* = 12.6 Hz, 3H); **^{13}C NMR (150 MHz, CDCl$_3$)** δ 167.6, 158.0, 156.8, 141.7 (d, *J* = 12.0 Hz), 138.7 (d, *J* = 7.5 Hz), 138.2 (d, *J* = 19.5 Hz), 137.70, 132.52, 131.3 (d, *J* = 6.0 Hz), 129.91, 129.11, 128.58, 128.46, 128.13, 127.78, 127.1 (d, *J* = 9.0 Hz), 125.36, 123.53, 121.59, 118.62, 117.51, 111.87, 51.95, 16.2 (d, *J* = 55.5 Hz); **^{31}P NMR (243 MHz, CDCl$_3$)** δ 42.6; **HRMS (ESI) :** calcd. for $C_{31}H_{24}N_3NaO_3P^+$

[M+Na]$^+$: 540.1447; found : 540.1451.

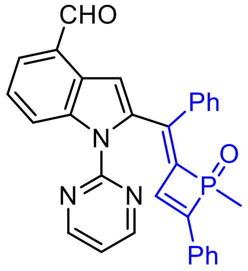

(*E*)-2-((1-methyl-1-oxido-4-phenyl-2*H*-phosphet-2-ylidene)(phenyl)methyl)-1-(pyrimidin-2-yl)-1*H*-indole-4-carbaldehyde (3e)

The title compound was isolated as a yellow solid (eluent: petroleum ether/ethyl acetate = 5/1, 89.7 mg, 92%). **^{1}H NMR (600 MHz, CDCl$_3$)** δ 10.29 (s, 1H), 8.55 (dd, *J* = 8.4, 0.9 Hz, 1H), 8.53 (d, *J* = 4.8 Hz, 2H), 7.87 (d, *J* = 67.7 Hz, 1H), 7.78 (dd, *J* = 7.4, 1.0 Hz, 1H), 7.71 (s, 1H), 7.57 – 7.55 (m, 2H), 7.51 – 7.46 (m, 3H), 7.42 – 7.38 (m, 2H), 7.37 – 7.34 (m, 1H), 7.19 – 7.15 (m, 2H), 7.11 – 7.08 (m, 1H), 6.92 (t, *J* = 4.8 Hz, 1H), 1.75 (d, *J* = 12.7 Hz, 3H); **^{13}C NMR (150 MHz, CDCl$_3$)** δ 193.0, 158.1, 156.8 (d, *J* = 78.0 Hz) , 156.7, 144.4 (d, *J* = 75.0 Hz), 141.6 (d, *J* = 12.0 Hz), 139.6 (d, *J* = 21.0 Hz), 138.6 (d, *J* = 7.5 Hz), 137.7, 132.2, 131.3 (d, *J* = 7.5 Hz), 130.0, 129.5, 129.1, 128.5, 128.3, 128.2, 127.8, 127.2 (d, *J* = 7.5 Hz), 126.6, 123.7, 120.1, 117.7, 110.9, 16.1 (d, *J* = 55.5 Hz); **^{31}P NMR (243 MHz, CDCl$_3$)** δ 42.7; **HRMS (ESI) :** calcd. for $C_{30}H_{23}N_3O_2P^+$ [M+H]$^+$: 488.1522; found : 488.1526.

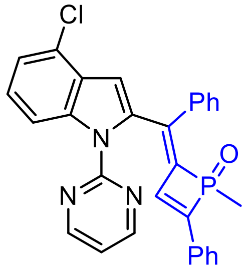

(*E*)-2-((4-chloro-1-(pyrimidin-2-yl)-1*H*-indol-2-yl)(phenyl)methylene)-1-methyl-4-phenyl-2*H*-phosphete 1-oxide (3f)

The title compound was isolated as a yellow solid (eluent: petroleum ether/ethyl acetate = 5/1, 94.8 mg, 96%). **^{1}H NMR (600 MHz, CDCl$_3$)** δ 8.52 (d, *J* = 4.8 Hz, 2H), 8.20 (dt, *J* = 7.8, 1.1 Hz, 1H), 7.84 (d, *J* = 67.7 Hz, 1H), 7.57 – 7.55 (m, 2H), 7.53 – 7.50 (m, 2H), 7.43 – 7.39 (m, 2H), 7.38 – 7.35 (m, 1H), 7.30 – 7.25 (m, 2H), 7.21 –7.18 (m, 2H), 7.14 – 7.11 (m, 1H), 6.96 (s, 1H), 6.91 (t, *J* = 4.8 Hz, 1H), 1.77 (d, *J* = 12.7 Hz, 3H); **^{13}C NMR (150 MHz, CDCl$_3$)** δ 158.0, 156.8, 156.5 (d, *J* = 78.0 Hz), 143.8 (d, *J* = 75.0 Hz), 141.6 (d, *J* = 12.0 Hz), 138.7 (d, *J* = 7.5 Hz), 137.7, 136.9 (d, *J* = 15.0 Hz), 132.4, 131.3 (d, *J* = 5.0 Hz), 130.0, 129.2, 128.5, 128.2, 127.8, 127.6, 127.1 (d, *J* = 7.5 Hz), 125.9, 124.9, 122.1, 117.5, 112.6, 109.3, 16.2 (d, *J* = 55.5 Hz); **^{31}P NMR (243 MHz,**

$CDCl_3$) δ 42.5; **HRMS (ESI) :** calcd. for $C_{29}H_{22}ClN_3OP^+$ $[M+H]^+$: 494.1184; found : 494.1189.

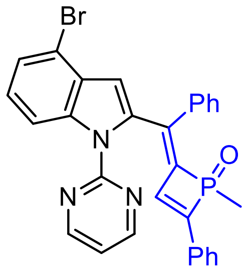

(*E*)-2-((4-bromo-1-(pyrimidin-2-yl)-1*H*-indol-2-yl)(phenyl)methylene)-1-methyl-4-phenyl-2*H*-phosphete 1-oxide (3g)

The title compound was isolated as a yellow solid (eluent: petroleum ether/ethyl acetate = 5/1, 102.3 mg, 95%). **^{1}H NMR (600 MHz, $CDCl_3$)** δ 8.52 (d, *J* = 4.8 Hz, 2H), 8.25 (d, *J* = 8.3 Hz, 1H), 7.83 (d, *J* = 67.6 Hz, 1H), 7.59 – 7.55 (m, 2H), 7.54 – 7.51 (m, 2H), 7.46 (d, *J* = 7.7 Hz, 1H), 7.42 (t, *J* = 7.6 Hz, 2H), 7.38 – 7.35 (m, 1H), 7.21 (q, *J* = 7.9 Hz, 3H), 7.14 – 7.11 (m, 1H), 6.91 (t, *J* = 4.8 Hz, 1H), 6.90 (s, 1H), 1.77 (d, *J* = 12.7 Hz, 3H); **^{13}C NMR (150 MHz, $CDCl_3$)** δ 158.0, 156.9, 156.5 (d, *J* = 78.0 Hz), 143.9 (d, *J* = 75.0 Hz), 141.6 (d, *J* = 12.0 Hz), 138.6 (d, *J* = 9.0 Hz), 137.3, 137.0 (d, *J* = 21.0 Hz), 132.37, 131.3 (d, *J* = 6.0 Hz), 129.96, 129.40, 129.15, 128.49, 128.20, 127.79, 127.1 (d, *J* = 9.0 Hz), 125.24, 125.16, 117.54, 114.55, 113.14, 110.92, 16.2 (d, *J* = 55.5 Hz); **^{31}P NMR (243 MHz, $CDCl_3$)** δ 42.5; **HRMS (ESI) :** calcd. for $C_{29}H_{21}BrN_3NaOP^+$ $[M+Na]^+$: 560.0498; found : 560.0503.

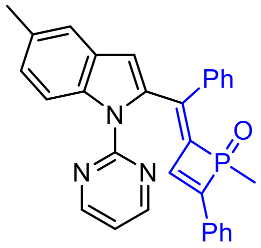

(*E*)-1-methyl-2-((5-methyl-1-(pyrimidin-2-yl)-1*H*-indol-2-yl)(phenyl)methylene)-4-phenyl-2*H*-phosphete 1-oxide (3h)

The title compound was isolated as a yellow solid (eluent: petroleum ether/ethyl acetate = 5/1, 90.0 mg, 95%). **^{1}H NMR (600 MHz, $CDCl_3$)** δ 8.49 (d, *J* = 4.8 Hz, 2H), 8.24 (d, *J* = 8.5 Hz, 1H), 7.80 (d, *J* = 67.8 Hz, 1H), 7.55 – 7.52(m, 4H), 7.47 (s, 1H), 7.39 (t, *J* = 7.6 Hz, 2H), 7.36 – 7.33 (m, 1H), 7.21 – 7.17 (m, 3H), 7.14 – 7.11 (m, 1H), 6.84 (t, *J* = 4.8 Hz, 1H), 6.76 (s, 1H), 2.50 (s, 3H), 1.76 (d, *J* = 12.7 Hz, 3H); **^{13}C NMR (150 MHz, $CDCl_3$)** δ 157.8, 157.1, 155.7 (d, *J* = 78.0 Hz), 142.8 (d, *J* = 76.5 Hz), 141.9 (d, *J* = 12.0 Hz), 139.0 (d, *J* = 9.0 Hz), 136.2 (d, *J* = 19.5 Hz), 135.4, 133.4, 131.9, 131.5 (d, *J* = 6.0 Hz), 129.7, 129.1, 129.0, 128.4, 128.1, 127.8, 127.0 (d, *J* = 9.0 Hz), 125.9, 120.5, 116.8, 113.9, 111.4, 21.4, 16.3 (d, *J* = 55.5 Hz); **^{31}P NMR (243 MHz, $CDCl_3$)** δ 42.4; **HRMS**

(ESI) : calcd. for $C_{30}H_{24}N_3NaOP^+$ $[M+Na]^+$: 496.1549; found : 496.1549.

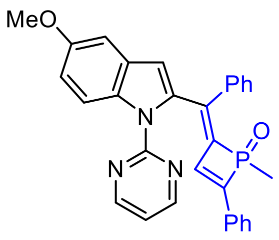

(*E*)-2-((5-methoxy-1-(pyrimidin-2-yl)-1*H*-indol-2-yl)(phenyl)methylene)-1-methyl-4-phenyl-2*H*-phosphete 1-oxide (3i) The title compound was isolated as a yellow solid (eluent: petroleum ether/ethyl acetate = 5/1, 85.2 mg, 87%). **^{1}H NMR (600 MHz, $CDCl_3$)** δ 8.48 (d, *J* = 4.8 Hz, 2H), 8.28 (d, *J* = 9.1 Hz, 1H), 7.80 (d, *J* = 67.8 Hz, 1H), 7.56 – 7.52 (m, 4H), 7.40 (t, *J* = 7.6 Hz, 2H), 7.36 – 7.33 (m, 1H), 7.22 – 7.19 (m, 2H), 7.15 – 7.11 (m, 2H), 7.00 (dd, *J* = 9.1, 2.6 Hz, 1H), 6.85 (t, *J* = 4.8 Hz, 1H), 6.77 (s, 1H), 3.90 (s, 3H), 1.77 (d, *J* = 12.7 Hz, 3H); **^{13}C NMR (150 MHz, $CDCl_3$)** δ 157.8, 157.0, 155.8, 155.7 (d, *J* = 76.5 Hz), 142.8 (d, *J* = 75.0 Hz), 141.8 (d, *J* = 12.0 Hz), 139.0 (d, *J* = 9.0 Hz), 136.7 (d, *J* = 21.0 Hz), 133.3, 132.1, 131.4 (d, *J* = 6.0 Hz), 129.8, 129.5, 129.1, 128.4, 128.1, 127.8, 127.0 (d, *J* = 9.0 Hz), 116.9, 115.2, 113.8, 111.4, 102.6, 55.8, 16.3 (d, *J* = 55.5 Hz); **^{31}P NMR (243 MHz, $CDCl_3$)** δ 42.5; **HRMS (ESI) :** calcd. for $C_{30}H_{24}N_3NaO_2P^+$ $[M+Na]^+$: 512.1498; found : 512.1497.

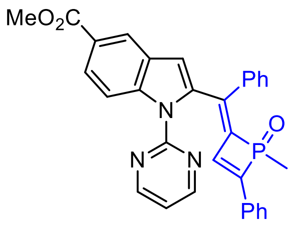

methyl (*E*)-2-((1-methyl-1-oxido-4-phenyl-2*H*-phosphet-2-ylidene)(phenyl)methyl)-1-(pyrimidin-2-yl)-1*H*-indole-5-carboxylate (3j)

The title compound was isolated as a yellow solid (eluent: petroleum ether/ethyl acetate = 5/1, 91.1 mg, 88%). **^{1}H NMR (600 MHz, $CDCl_3$)** 8.54 (d, *J* = 4.8 Hz, 2H), 8.45 – 8.42 (m, 1H), 8.30 (d, *J* = 8.8 Hz, 1H), δ 8.04 (dd, *J* = 8.8, 1.7 Hz, 1H), 7.80 (d, *J* = 67.6 Hz, 1H), 7.54 (d, *J* = 8.0 Hz, 2H), 7.51 (d, *J* = 7.3 Hz, 2H), 7.40 (t, *J* = 7.5 Hz, 2H), 7.36 (t, *J* = 7.3 Hz, 1H), 7.20 (t, *J* = 7.6 Hz, 2H), 7.12 (t, *J* = 7.4 Hz, 1H), 6.94 – 6.91 (m, 2H), 3.97 (s, 3H), 1.76 (d, *J* = 12.7 Hz, 3H); **^{13}C NMR (150 MHz, $CDCl_3$)** δ 167.6, 158.0, 156.8, 156.6 (d, *J* = 76.5 Hz), 143.8 (d, *J* = 75.0 Hz), 141.4 (d, *J* = 12.0 Hz), 139.6, 138.6 (d, *J* = 9.0 Hz), 137.7 (d, *J* = 21.0 Hz), 132.3, 131.3 (d, *J* = 6.0 Hz), 130.0, 129.2, 128.51, 128.4, 128.2, 127.8, 127.0 (d, *J* = 9.0 Hz), 125.5, 124.4,

123.3, 117.6, 113.6, 111.7, 52.0, 16.3 (d, J = 55.5 Hz); **^{31}P NMR (243 MHz, $CDCl_3$)** δ 42.3; **HRMS (ESI) :** calcd. for $C_{31}H_{24}N_3NaO_3P^+$ [M+Na]$^+$: 540.1447; found : 540.1443.

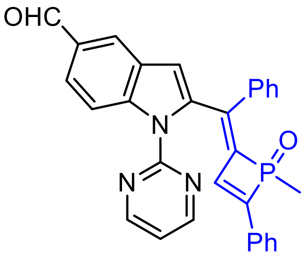

(*E*)-2-((1-methyl-1-oxido-4-phenyl-2*H*-phosphet-2-ylidene)(phenyl)methyl)-1-(pyrimidin-2-yl)-1*H*-indole-5-carbaldehyde (3k)

The title compound was isolated as a yellow solid (eluent: petroleum ether/ethyl acetate = 5/1, 89.7 mg, 92%). **^{1}H NMR (600 MHz, $CDCl_3$)** δ 10.10 (s, 1H), 8.55 (d, J = 4.8 Hz, 2H), 8.36 (d, J = 8.6 Hz, 1H), 8.24 – 8.21 (m, 1H), 7.89 (dd, J = 8.7, 1.6 Hz, 1H), 7.80 (d, J = 67.6 Hz, 1H), 7.57 – 7.53 (m, 2H), 7.52 – 7.49 (m, 2H), 7.43 – 7.39 (m, 2H), 7.38 – 7.35 (m, 1H), 7.22 – 7.18 (m, 2H), 7.14 – 7.11 (m, 1H), 6.97 (s, 1H), 6.96 (t, J = 4.8 Hz, 1H), 1.77 (d, J = 12.8 Hz, 3H); **^{13}C NMR (150 MHz, $CDCl_3$)** δ 192.1, 158.1, 156.8 (d, J = 76.5 Hz) , 156.6, 144.2 (d, J = 75.0 Hz), 141.3 (d, J = 12.0 Hz), 140.4, 138.4 (d, J = 7.5 Hz), 138.3 (d, J = 21.0 Hz), 131.9, 131,6, 131.2 (d, J = 6.0 Hz), 130.1, 129.2, 128.7, 128.6, 128.3, 127.7, 127.1 (d, J = 7.5 Hz), 124.9, 124.7, 117.9, 114.4, 111.7, 16.3 (d, J = 55.5 Hz); **^{31}P NMR (243 MHz, $CDCl_3$)** δ 42.4; **HRMS (ESI) :** calcd. for $C_{30}H_{22}N_3NaO_2P^+$ [M+Na]$^+$: 510.1342; found : 510.1341.

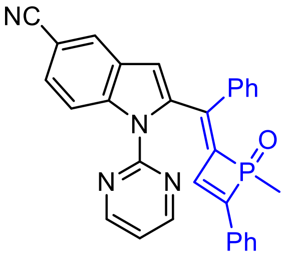

(*E*)-2-((1-methyl-1-oxido-4-phenyl-2*H*-phosphet-2-ylidene)(phenyl)methyl)-1-(pyrimidin-2-yl)-1*H*-indole-5-carbonitrile (3l)

The title compound was isolated as a yellow solid (eluent: petroleum ether/ethyl acetate = 5/1, 87.2 mg, 90%). **^{1}H NMR (600 MHz, $CDCl_3$)** δ 8.56 (d, J = 4.8 Hz, 2H), 8.36 (d, J = 8.6 Hz, 1H), 8.04 (s, 1H), 7.78 (d, J = 67.4 Hz, 1H), 7.58 (dd, J = 8.7, 1.7 Hz, 1H), 7.56 (d, J = 7.9 Hz, 2H), 7.49 (d, J = 7.3 Hz, 2H), 7.42 (t, J = 7.4 Hz, 2H), 7.40 – 7.36 (m, 1H), 7.22 – 7.19 (m, 2H), 7.16 – 7.12 (m, 1H), 6.97 (t, J = 4.8 Hz, 1H), 6.91 (s, 1H), 1.78 (d, J = 12.7 Hz, 3H); **^{13}C NMR (150 MHz, $CDCl_3$)** δ 158.1, 157.1 (d, J = 78.0 Hz) ,156.5, 144.4 (d, J

= 75.0 Hz), 141.1 (d, *J* = 13.5 Hz), 138.8, 138.6, 138.3 (d, *J* = 7.5 Hz), 131.6, 131.1 (d, *J* = 6.0 Hz), 130.2, 129.2, 128.6, 128.5, 128.4, 127.7, 127.1, 127.1, 125.8, 120.1, 118.0, 114.9, 110.6, 105.6, 16.3 (d, *J* = 55.5 Hz); **^{31}P NMR (243 MHz, CDCl$_3$)** δ 42.4; **HRMS (ESI) :** calcd. for $C_{30}H_{21}N_4NaOP^+$ [M+Na]$^+$: 507.1345; found : 507.1341.

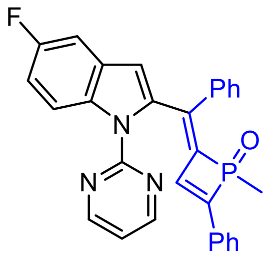

(*E*)-2-((5-fluoro-1-(pyrimidin-2-yl)-1*H*-indol-2-yl)(phenyl)methylene)-1-methyl-4-phenyl-2*H*-phosphete 1-oxide (3m)

The title compound was isolated as a yellow solid (eluent: petroleum ether/ethyl acetate = 5/1, 86.9 mg, 91%). **^{1}H NMR (600 MHz, CDCl$_3$)** δ 8.51 (d, *J* = 4.8 Hz, 2H), 8.30 (dd, *J* = 9.1, 4.5 Hz, 1H), 7.79 (d, *J* = 67.7 Hz, 1H), 7.56 – 7.51 (m, 4H), 7.40 (t, *J* = 7.6 Hz, 2H), 7.37 – 7.34 (m, 1H), 7.33 (dd, *J* = 8.8, 2.6 Hz, 1H), 7.21 (t, *J* = 7.7 Hz, 2H), 7.13 (t, *J* = 7.3 Hz, 1H), 7.09 (td, *J* = 9.1, 2.6 Hz, 1H), 6.89 (t, *J* = 4.8 Hz, 1H), 6.80 (s, 1H), 1.76 (d, *J* = 12.7 Hz, 3H); **^{13}C NMR (150 MHz, CDCl$_3$)** δ 159.1 (d, *J* = 238.5 Hz), 157.9, 156.9, 156.2 (d, *J* = 78.0 Hz), 143.4 (d, *J* = 76.5 Hz), 141.6 (d, *J* = 12.0 Hz), 138.8 (d, *J* = 7.5 Hz), 137.8 (d, *J* = 21.0 Hz), 133.5, 132.8, 131.3 (d, *J* = 6.0 Hz), 129.9, 129.4 (d, *J* = 10.5 Hz), 129.2, 128.5, 128.2, 127.7, 127.0 (d, *J* = 9.0 Hz), 117.2, 115.2 (d, *J* = 9.0 Hz), 112.3 (d, *J* = 25.5 Hz), 111.1 (d, *J* = 4.5 Hz), 105.8 (d, *J* = 24.0 Hz), 16.3 (d, *J* = 55.5 Hz); **^{31}P NMR (243 MHz, CDCl$_3$)** δ 42.5; **HRMS (ESI) :** calcd. for $C_{29}H_{21}FN_3NaOP^+$ [M+Na]$^+$: 500.1298; found : 500.1300.

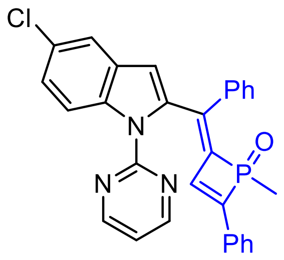

(*E*)-2-((5-chloro-1-(pyrimidin-2-yl)-1*H*-indol-2-yl)(phenyl)methylene)-1-methyl-4-phenyl-2*H*-phosphete 1-oxide (3n)

The title compound was isolated as a yellow solid (eluent: petroleum ether/ethyl acetate = 5/1, 96.8 mg, 98%). **^{1}H NMR (600 MHz, CDCl$_3$)** δ 8.51 (d, *J* = 4.8 Hz, 2H), 8.27 (d, *J* = 8.9 Hz, 1H), 7.78 (d, *J* = 67.7 Hz, 1H), 7.65 (d, *J* = 2.1 Hz, 1H), 7.56 – 7.53 (m, 2H), 7.53 – 7.50 (m, 2H), 7.41 (t, *J* = 7.6 Hz, 2H), 7.38 – 7.35 (m, 1H), 7.30 (dd, *J* = 8.9, 2.1 Hz, 1H), 7.20 (t, *J* = 7.7 Hz, 2H), 7.15 – 7.12 (m, 1H), 6.90 (t, *J* = 4.8 Hz, 1H), 6.78 (s, 1H), 1.77 (d, *J* = 12.7 Hz, 3H); **^{13}C NMR (150 MHz, CDCl$_3$)** δ 157.9, 156.8, 156.4 (d,

J = 78.0 Hz), 143.5 (d, J = 75.0 Hz), 141.5 (d, J = 12.0 Hz), 138.7 (d, J = 9.0 Hz), 137.5 (d, J = 19.5 Hz), 135.4, 132.6, 131.3 (d, J = 6.0 Hz), 130.0, 129.8, 129.2, 128.5, 128.2, 127.9, 127.7, 127.0 (d, J = 9.0 Hz), 124.5, 120.1, 117.4, 115.3, 110.6, 16.3 (d, J = 55.5 Hz); **^{31}P NMR (243 MHz, CDCl3)** δ 42.5; **HRMS (ESI) :** calcd. for $C_{29}H_{21}ClN_3NaOP^+$ $[M+Na]^+$: 516.1003; found : 516.1005.

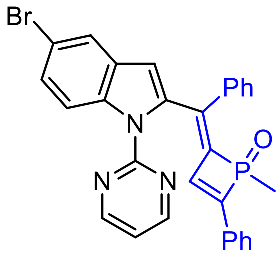

(*E*)-2-((5-bromo-1-(pyrimidin-2-yl)-1*H*-indol-2-yl)(phenyl) methylene)-1-methyl-4-phenyl-2*H*-phosphete 1-oxide (3o)

The title compound was isolated as a yellow solid (eluent: petroleum ether/ethyl acetate = 5/1, 103.4 mg, 96%). **^{1}H NMR (600 MHz, CDCl3)** δ 8.51 (d, J = 4.8 Hz, 2H), 8.22 (d, J = 8.8 Hz, 1H), 7.83 (s, 0.5H), 7.81 (d, J = 2.0 Hz, 1H), 7.72 (s, 0.5H), 7.56 – 7.53 (m, 2H), 7.52 – 7.49 (m, 2H), 7.43 (dd, J = 8.9, 2.0 Hz, 1H), 7.40 (t, J = 7.6 Hz, 2H), 7.37 – 7.34 (m, 1H), 7.22 – 7.19 (m, 2H), 7.14 – 7.11 (m, 1H), 6.90 (t, J = 4.8 Hz, 1H), 6.78 (s, 1H), 1.76 (d, J = 12.7 Hz, 3H); **^{13}C NMR (150 MHz, CDCl3)** δ 157.9, 156.8, 156.4 (d, J = 78.0 Hz), 143.6 (d, J = 75.0 Hz), 141.5 (d, J = 12.0 Hz), 138.7 (d, J = 7.5 Hz), 137.4 (d, J = 21.0 Hz), 135.7, 132.5, 131.3 (d, J = 6.0 Hz), 130.4, 130.0, 129.2, 128.5, 128.2, 127.7, 127.1, 127.0 (d, J = 9.0 Hz), 123.2, 117.4, 115.7, 115.6, 110.5, 16.3 (d, J = 55.5 Hz); **^{31}P NMR (243 MHz, CDCl3)** δ 42.4; **HRMS (ESI) :** calcd. for $C_{29}H_{21}BrN_3NaOP^+$ $[M+Na]^+$: 560.0498; found : 560.0497.

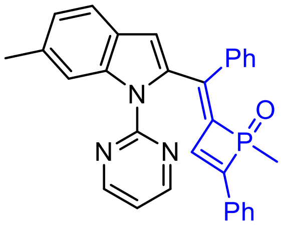

(*E*)-1-methyl-2-((6-methyl-1-(pyrimidin-2-yl)-1*H*-indol-2-yl)(phenyl)methylene)-4-phenyl-2*H*-phosphete 1-oxide (3p)

The title compound was isolated as a yellow solid (eluent: petroleum ether/ethyl acetate = 5/1, 93.8 mg, 99%). **^{1}H NMR (600 MHz, CDCl3)** δ 8.52 (d, J = 4.8 Hz, 2H), 8.12 (s, 1H), 7.84 (d, J = 67.9 Hz, 1H), 7.57 (d, J = 8.0 Hz, 1H), 7.55 – 7.52 (m, 4H), 7.40 (t, J = 7.5 Hz, 2H), 7.36 – 7.33 (m, 1H), 7.19 (t, J = 7.7 Hz, 2H), 7.14 – 7.11 (m, 2H), 6.86 (t, J = 4.8 Hz, 1H), 6.80 (s, 1H), 2.53 (s, 3H), 1.76 (d, J = 12.7 Hz, 3H); **^{13}C NMR (150 MHz, CDCl3)** δ 157.9, 157.1, 155.6 (d, J = 76.5 Hz), 142,8 (d, J = 75.0 Hz), 141.9 (d, J = 12.0 Hz), 139.0 (d,

J = 9.0 Hz), 137.5, 135.7 (d, J = 21.0 Hz), 134.6, 133.3, 131.5 (d, J = 6.0 Hz), 129.7, 129.1, 128.4, 128.1, 127.9, 127.0 (d, J = 9.0 Hz), 126.5, 124.1, 120.45, 117.0, 113.8, 111.6, 22.2, 16.3 (d, J = 55.5 Hz); **^{31}P NMR (243 MHz, CDCl$_3$)** δ 42.5; **HRMS (ESI) :** calcd. for $C_{30}H_{24}N_3NaOP^+$ [M+Na]$^+$: 496.1549; found : 496.1547.

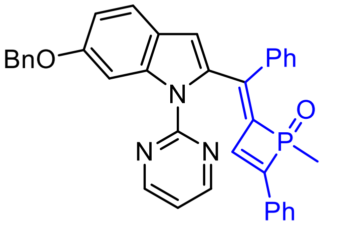

(*E*)-2-((6-(benzyloxy)-1-(pyrimidin-2-yl)-1*H*-indol-2-yl)(phenyl)methylene)-1-methyl-4-phenyl-2*H*-phosphete 1-oxide (3q)

The title compound was isolated as a yellow solid (eluent: petroleum ether/ethyl acetate = 5/1, 108.6 mg, 96%). **^{1}H NMR (600 MHz, CDCl$_3$)** δ 8.50 (d, J = 4.8 Hz, 2H), 8.04 (d, J = 2.2 Hz, 1H), 7.84 (d, J = 68.1 Hz, 1H), 7.58 – 7.53 (m, 5H), 7.49 (d, J = 7.4 Hz, 2H), 7.42 – 7.38 (m, 4H), 7.36 – 7.31 (m, 2H), 7.21 (t, J = 7.7 Hz, 2H), 7.13 (t, J = 7.3 Hz, 1H), 7.03 (dd, J = 8.6, 2.2 Hz, 1H), 6.86 (t, J = 4.8 Hz, 1H), 6.78 (s, 1H), 5.16 (dd, J = 28.3, 11.5 Hz, 2H), 1.76 (d, J = 12.7 Hz, 3H); **^{13}C NMR (150 MHz, CDCl$_3$)** δ 157.8, 157.3, 157.2, 155.6 (d, J = 78.0 Hz), 142.0 (d, J = 12.0 Hz), 139.1 (d, J = 7.5 Hz), 138.1, 137.3, 135.4, 135.3, 133.4, 131.5 (d, J = 6.0 Hz), 129.7, 129.1, 128.6, 128.4, 128.1, 128.0, 127.9, 127.7, 127.0 (d, J = 9.0 Hz), 123.1, 121.3, 116.9, 112.5, 111.7, 99.6, 70.7, 16.3 (d, J = 55.5 Hz); **^{31}P NMR (243 MHz, CDCl$_3$)** δ 42.5; **HRMS (ESI) :** calcd. for $C_{36}H_{28}N_3NaO_2P^+$ [M+Na]$^+$: 588.1811; found : 588.1813.

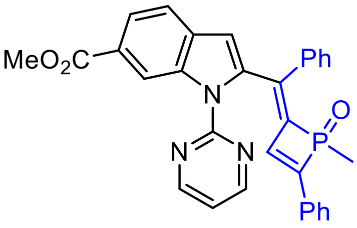

methyl (*E*)-2-((1-methyl-1-oxido-4-phenyl-2*H*-phosphet-2-ylidene)(phenyl)methyl)-1-(pyrimidin-2-yl)-1*H*-indole-6-carboxylate (3r)

The title compound was isolated as a yellow solid (eluent: petroleum ether/ethyl acetate = 5/1, 96.3 mg, 93%). **^{1}H NMR (600 MHz, CDCl$_3$)** δ 8.97 (s, 1H), 8.54 (d, J = 4.8 Hz, 2H), 7.97 (dd, J = 8.3, 1.5 Hz, 1H), 7.82 (s, 0.5H), 7.71 (s, 1H), 7.69 (s, 0.5H), 7.53 – 7.50 (m, 4H), 7.37 (t, J = 7.4 Hz, 2H), 7.33 (t, J = 7.2 Hz, 1H), 7.19 (t, J = 7.6 Hz, 2H), 7.12 (t, J = 7.3 Hz, 1H), 6.91 (t, J = 4.8 Hz, 1H), 6.86 (s, 1H), 3.92 (s,

3H), 1.76 (d, J = 12.7 Hz, 3H); **^{13}C NMR (150 MHz, CDCl$_3$)** δ 167.8, 158.1, 156.7, 156.7 (d, J = 76.5 Hz) 156.4, 143.9 (d, J = 75.0 Hz), 141.4 (d, J = 12.0 Hz), 139.4 (d, J = 19.5 Hz), 138.5 (d, J = 9.0 Hz), 136.5, 132.3, 131.2 (d, J = 6.0 Hz), 130.0, 129.2, 128.5, 128.3, 127.8, 127.1 (d, J = 9.0 Hz), 125.9, 123.5, 120.4, 117.6, 116.0, 111.0, 52.1, 16.3 (d, J = 55.5 Hz); **^{31}P NMR (243 MHz, CDCl$_3$)** δ 42.5; **HRMS (ESI) :** calcd. for $C_{31}H_{24}N_3NaO_3P^+$ [M+Na]$^+$: 540.1447; found : 540.1449.

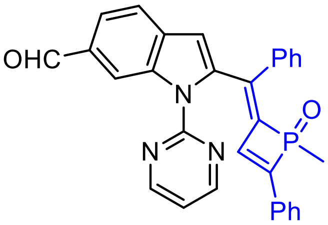

(*E*)-2-((1-methyl-1-oxido-4-phenyl-2*H*-phosphet-2-ylidene)(phenyl)methyl)-1-(pyrimidin-2-yl)-1*H*-indole-6-carbaldehyde (3s)

The title compound was isolated as a yellow solid (eluent: petroleum ether/ethyl acetate = 5/1, 86.8 mg, 89%). **^{1}H NMR (600 MHz, CDCl$_3$)** δ 10.10 (s, 1H), 8.82 (s, 1H), 8.57 (d, J = 4.7 Hz, 2H), 7.85 – 7.82 (m, 1.5H), 7.79 (d, J = 8.1 Hz, 1H), 7.71 (s, 0.5H), 7.56 – 7.51 (m, 4H), 7.40 (t, J = 7.2 Hz, 2H), 7.38 – 7.35 (m, 1H), 7.21 (t, J = 7.6 Hz, 2H), 7.16 – 7.13 (m, 1H), 6.96 (t, J = 4.8 Hz, 1H), 6.91 (s, 1H), 1.77 (d, J = 12.7 Hz, 3H); **^{13}C NMR (150 MHz, CDCl$_3$)** δ 192.4, 158.2, 157.1 (d, J = 76.5 Hz), 156.7, 144.3 (d, J = 75.0 Hz), 141.2 (d, J = 12.0 Hz), 140.5 (d, J = 19.5 Hz), 138.4 (d, J = 7.5 Hz), 136.7, 133.6, 133.0, 132.0, 131.2 (d, J = 7.5 Hz), 130.1, 129.2, 128.6, 128.3, 127.8, 127.1 (d, J = 9.0 Hz), 123.0, 121.2, 117.8, 117.5, 111.1, 16.3 (d, J = 55.5 Hz); **^{31}P NMR (243 MHz, CDCl$_3$)** δ 42.4; **HRMS (ESI) :** calcd. for $C_{30}H_{22}N_3NaO_2P^+$ [M+Na]$^+$: 510.1342; found : 510.1342.

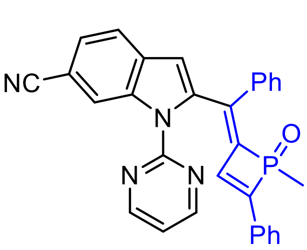

(*E*)-2-((1-methyl-1-oxido-4-phenyl-2*H*-phosphet-2-ylidene)(phenyl)methyl)-1-(pyrimidin-2-yl)-1*H*-indole-6-carbonitrile (3t)

The title compound was isolated as a yellow solid (eluent: petroleum ether/ethyl acetate = 5/1, 94.0 mg, 97%). **^{1}H NMR (600 MHz, CDCl$_3$)** δ 8.72 (s, 1H), 8.55 (d, J = 4.8 Hz, 2H), 7.79 (s, 0.5H), 7.75 (d, J = 8.1 Hz, 1H), 7.68 (s, 0.5H), 7.55 – 7.49 (m, 5H), 7.40 (t, J = 7.5 Hz, 2H), 7.38 – 7.35 (m, 1H), 7.21 (t, J = 7.6 Hz, 2H), 7.14 (t, J = 7.3 Hz, 1H), 6.97 (t, J = 4.8 Hz, 1H), 6.89 (s, 1H), 1.77 (d, J

= 12.7 Hz, 3H); **^{13}C NMR (150 MHz, CDCl$_3$)** δ 158.1, 157.2 (d, *J* = 76.5 Hz), 156.5, 144.3 (d, *J* = 75.0 Hz), 141.1 (d, *J* = 12.0 Hz), 140.0 (d, *J* = 21.0 Hz), 138.3 (d, *J* = 7.5 Hz), 135.9, 131.9, 131.8, 131.1 (d, *J* = 6.0 Hz), 130.2, 129.2, 128.6, 128.4, 127.7, 127.1 (d, *J* = 9.0 Hz), 125.3, 121.5, 120.3, 119.2, 117.9, 111.0, 106.8, 16.3 (d, *J* = 55.5 Hz); **^{31}P NMR (243 MHz, CDCl$_3$)** δ 42.3; **HRMS (ESI) :** calcd. for $C_{30}H_{21}N_4NaOP^+$ $[M+Na]^+$: 507.1345; found : 507.1343.

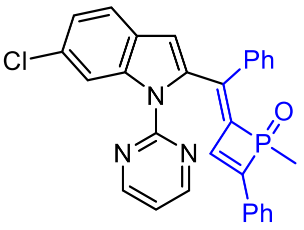

(*E*)-2-((6-chloro-1-(pyrimidin-2-yl)-1*H*-indol-2-yl)(phenyl)methylene)-1-methyl-4-phenyl-2*H*-phosphete 1-oxide (3u)

The title compound was isolated as a yellow solid (eluent: petroleum ether/ethyl acetate = 5/1, 93.8 mg, 95%). **^{1}H NMR (600 MHz, CDCl$_3$)** δ 8.52 (d, *J* = 4.8 Hz, 2H), 8.38 (s, 1H), 7.78 (d, *J* = 67.7 Hz, 1H), 7.59 (d, *J* = 8.4 Hz, 1H), 7.55 – 7.51 (m, 4H), 7.39 (t, *J* = 7.6 Hz, 2H), 7.36 – 7.33 (m, 1H), 7.27 – 7.25 (m, 1H), 7.20 (t, *J* = 7.6 Hz, 2H), 7.13 (t, *J* = 7.3 Hz, 1H), 6.90 (t, *J* = 4.8 Hz, 1H), 6.81 (s, 1H), 1.76 (d, *J* = 12.7 Hz, 3H); **^{13}C NMR (150 MHz, CDCl$_3$)** δ 158.0, 156.8, 156.2 (d, *J* = 76.5 Hz), 143.3 (d, *J* = 76.5 Hz), 141.6 (d, *J* = 13.5 Hz), 138.7 (d, *J* = 9.0 Hz), 137.4, 137.0 (d, *J* = 19.5 Hz), 132.7, 131.3 (d, *J* = 6.0 Hz), 130.3, 129.9, 129.1, 128.5, 128.2, 127.8, 127.2, 127.0 (d, *J* = 9.0 Hz), 123.1, 121.5, 117.4, 114.3, 111.1, 16.2 (d, *J* = 55.5 Hz); **^{31}P NMR (243 MHz, CDCl$_3$)** δ 42.4; **HRMS (ESI) :** calcd. for $C_{29}H_{22}ClN_3OP^+$ $[M+H]^+$: 494.1184; found : 494.1185.

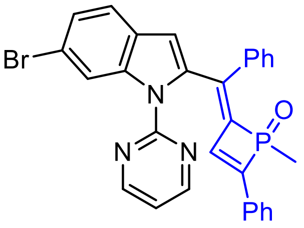

(*E*)-2-((6-bromo-1-(pyrimidin-2-yl)-1*H*-indol-2-yl)(phenyl)methylene)-1-methyl-4-phenyl-2*H*-phosphete 1-oxide (3v)

The title compound was isolated as a yellow solid (eluent: petroleum ether/ethyl acetate = 5/1, 106.6 mg, 99%). **^{1}H NMR (600 MHz, CDCl$_3$)** δ 8.57 – 8.50 (m, 3H), 7.78 (d, *J* = 67.7 Hz, 1H), 7.54 (d, *J* = 7.9 Hz, 3H), 7.53 – 7.49 (m, 2H), 7.42 –7.38 (m, 3H), 7.37 – 7.34 (m, 1H), 7.20 (t, *J* = 7.6 Hz, 2H), 7.13 (t, *J* = 7.3 Hz, 1H), 6.91 (t, *J* = 4.8 Hz, 1H), 6.81 (s, 1H), 1.76 (d, *J* = 12.7 Hz, 3H); **^{13}C**

NMR (150 MHz, CDCl₃) δ 158.0, 156.8, 156.3 (d, *J* = 76.5 Hz), 143.4 (d, *J* = 75.0 Hz), 141.5 (d, *J* = 12.0 Hz), 138.7 (d, *J* = 9.0 Hz), 137.7, 136.9 (d, *J* = 19.5 Hz), 132.6, 131.3 (d, *J* = 6.0 Hz), 129.9, 129.2, 128.5, 128.2, 127.8, 127.6, 127.0 (d, *J* = 9.0 Hz), 125.7, 121.9, 118.1, 117.4, 117.2, 111.2, 16.3 (d, *J* = 55.5 Hz); **^{31}P NMR (243 MHz, $CDCl_3$)** δ 42.3; **HRMS (ESI) :** calcd. for $C_{29}H_{21}BrN_3NaOP^+$ $[M+Na]^+$: 560.0498; found : 560.0503.

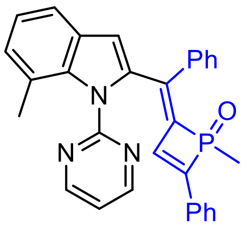

(*E*)-1-methyl-2-((7-methyl-1-(pyrimidin-2-yl)-1*H*-indol-2-yl)(phenyl)methylene)-4-phenyl-2*H*-phosphete 1-oxide (3w)

The title compound was isolated as a yellow solid (eluent: petroleum ether/ethyl acetate = 5/1, 88.1 mg, 93%). **^{1}H NMR (600 MHz, $CDCl_3$)** δ 8.50 (d, *J* = 4.8 Hz, 2H), 7.77 (d, *J* = 67.7 Hz, 1H), 7.58 (d, *J* = 7.9 Hz, 1H), 7.53 (d, *J* = 7.5 Hz, 2H), 7.42 – 7.37 (m, 4H), 7.35 (t, *J* = 7.2 Hz, 1H), 7.18 – 7.14 (m, 3H), 7.11 (t, *J* = 7.2 Hz, 1H), 7.06 (d, *J* = 7.3 Hz, 1H), 6.94 (t, *J* = 4.8 Hz, 1H), 6.80 (s, 1H), 2.02 (s, 3H), 1.70 (d, *J* = 12.7 Hz, 3H); **^{13}C NMR (150 MHz, $CDCl_3$)** δ 158.1, 157.7, 156.5 (d, *J* = 78.0 Hz), 145.5 (d, *J* = 73.5 Hz), 142.1 (d, *J* = 12.0 Hz), 138.1 (d, *J* = 9.0 Hz), 137.1 (d, *J* = 21.0 Hz), 136.4, 131.6, 131.3 (d, *J* = 6.0 Hz), 129.9, 129.1, 128.9, 128.3, 128.2, 128.0, 127.1 (d, *J* = 9.0 Hz), 126.7, 122.3, 121.7, 118.8, 118.8, 108.8, 20.3, 16.3 (d, *J* = 55.5 Hz); **^{31}P NMR (243 MHz, $CDCl_3$)** δ 41.9; **HRMS (ESI) :** calcd. for $C_{30}H_{24}N_3NaOP^+$ $[M+Na]^+$: 496.1549; found : 496.1547.

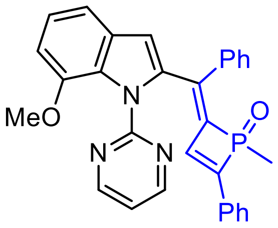

(*E*)-2-((7-methoxy-1-(pyrimidin-2-yl)-1*H*-indol-2-yl)(phenyl)methylene)-1-methyl-4-phenyl-2*H*-phosphete 1-oxide (3x)

The title compound was isolated as a yellow solid (eluent: petroleum ether/ethyl acetate = 5/1, 93.0 mg, 95%). **^{1}H NMR (600 MHz, $CDCl_3$)** δ 8.48 (d, *J* = 4.8 Hz, 2H), 7.77 (d, *J* = 67.8 Hz, 1H), 7.53 (d, *J* = 7.7 Hz, 2H), 7.43 (d, *J* = 7.3 Hz, 2H), 7.39 (t, *J* = 7.4 Hz, 2H), 7.34 (t, *J* = 7.2 Hz, 1H), 7.32 (d, *J* = 7.9 Hz, 1H), 7.18 – 7.14 (m, 3H), 7.12 (t, *J* = 7.2 Hz, 1H), 6.93 (t, *J* = 4.8 Hz, 1H), 6.77 – 6.73 (m, 2H), 3.64 (s, 3H), 1.67 (d, *J* = 12.7 Hz, 3H); **^{13}C NMR (150**

MHz, $CDCl_3$) δ 158.2, 157.3, 157.0, 156.4 (d, *J* = 78.0 Hz), 147.2, 145.5 (d, *J* = 73.5 Hz), 142.1 (d, *J* = 13.5 Hz), 138.1 (d, *J* = 7.5 Hz), 137.1 (d, *J* = 19.5 Hz), 131.3 (d, *J* = 6.0 Hz), 131.3, 130.2, 129.9, 129.1, 128.3, 128.2, 128.1, 127.1 (d, *J* = 12.0 Hz), 122.0, 118.6, 113.6, 108.4, 105.3, 55.6, 16.3 (d, *J* = 55.5 Hz); **^{31}P NMR (243 MHz, $CDCl_3$)** δ 42.0; **HRMS (ESI) :** calcd. for $C_{30}H_{24}N_3NaO_2P^+$ $[M+Na]^+$: 512.1498; found : 512.1499.

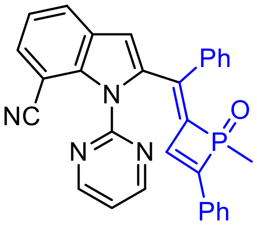

(*E*)-2-((1-methyl-1-oxido-4-phenyl-2*H*-phosphet-2-ylidene)(phenyl)methyl)-1-(pyrimidin-2-yl)-1*H*-indole-7-carbonitrile (3y)

The title compound was isolated as a yellow solid (eluent: petroleum ether/ethyl acetate = 5/1, 87.2 mg, 90%). **^1H NMR (600 MHz, $CDCl_3$)** δ 8.60 (d, *J* = 4.9 Hz, 2H), 7.95 (dd, *J* = 7.9, 1.2 Hz, 1H), 7.72 (d, *J* = 67.4 Hz, 1H), 7.64 (d, *J* = 7.5 Hz, 1H), 7.56 – 7.53 (m, 2H), 7.43 – 7.39 (m, 4H), 7.39 – 7.35 (m, 1H), 7.31 (t, *J* = 7.7 Hz, 1H), 7.21 – 7.17 (m, 2H), 7.15 – 7.12 (m, 1H), 7.05 (t, *J* = 4.9 Hz, 1H), 6.89 (s, 1H), 1.73 (d, *J* = 12.7 Hz, 3H); **^{13}C NMR (150 MHz, $CDCl_3$)** δ 158.4, 157.6 (d, *J* = 78 Hz), 155.9, 146.3 (d, *J* = 73.5 Hz), 141.3 (d, *J* = 12.0 Hz), 138.4 (d, *J* = 21.0 Hz), 137.6 (d, *J* = 9.0 Hz), 135.3, 131.1 (d, *J* = 6.0 Hz), 130.5, 130.2, 130.1, 129.6, 129.2, 128.5, 128.5, 127.9, 127.2 (d, *J* = 7.5 Hz), 126.1, 121.4, 119.8, 117.0, 108.5, 96.4, 16.3 (d, *J* = 55.5 Hz); **^{31}P NMR (243 MHz, $CDCl_3$)** δ 42.0; **HRMS (ESI) :** calcd. for $C_{30}H_{21}N_4NaOP^+$ $[M+Na]^+$: 507.1345; found : 507.1346.

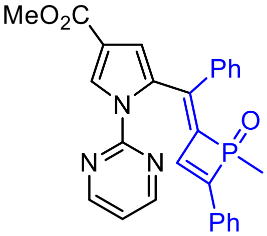

methyl (*E*)-5-((1-methyl-1-oxido-4-phenyl-2*H*-phosphet-2-ylidene)(phenyl)methyl)-1-(pyrimidin-2-yl)-1*H*-pyrrole-3-carboxylate (3z)

The title compound was isolated as a yellow solid (eluent: petroleum ether/ethyl acetate = 5/1, 82.3 mg, 88%). **^1H NMR (600 MHz, $CDCl_3$)** δ 8.47 – 8.37 (m, 3H), 7.75 (d, *J* = 67.7 Hz, 1H), 7.57 – 7.55 (m, 2H), 7.51 – 7.48 (m, 2H), 7.41 (t, *J* = 7.6 Hz, 2H), 7.38 – 7.35 (m, 1H), 7.23 – 7.19 (m, 2H), 7.14 – 7.10 (m, 1H), 6.94 (t, *J* =

4.8 Hz, 1H), 6.87 (d, J = 1.9 Hz, 1H), 3.90 (s, 3H), 1.75 (d, J = 12.7 Hz, 3H); **^{13}C NMR (150 MHz, $CDCl_3$)** δ 164.7, 158.1, 155.8 (d, J = 78.0 Hz), 155.6, 143.1 (d, J = 76.5 Hz), 141.6 (d, J = 12.0 Hz), 139.4 (d, J = 7.5 Hz), 132.3, 131.3 (d, J = 6.0 Hz), 130.5 (d, J = 21.0 Hz), 129.8, 129.1, 128.5, 128.0, 127.5, 127.4, 127.0 (d, J = 7.5 Hz), 118.4, 117.5, 116.7, 51.5, 16.1 (d, J = 55.5 Hz); **^{31}P NMR (243 MHz, $CDCl_3$)** δ 42.3; **HRMS (ESI) :** calcd. for $C_{27}H_{22}N_3NaO_3P^+$ $[M+Na]^+$: 490.1291; found : 490.1291.

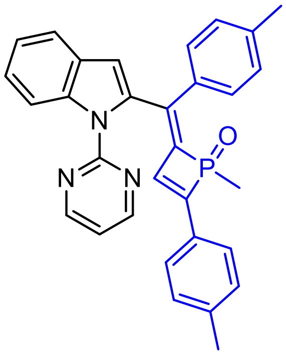

(*E*)-1-methyl-2-((1-(pyrimidin-2-yl)-1*H*-indol-2-yl)(*p*-tolyl)methylene)-4-(*p*-tolyl)-2*H*-phosphete 1-oxide (4a)

The title compound was isolated as a yellow solid (eluent: petroleum ether/ethyl acetate = 5/1, 89.7 mg, 92%). **^{1}H NMR (600 MHz, $CDCl_3$)** δ 8.52 (d, J = 4.8 Hz, 2H), 8.34 (d, J = 8.3 Hz, 1H), 7.76 (s, 0.5H), 7.68 (d, J = 7.7 Hz, 1H), 7.64 (s, 0.5H), 7.46 – 7.43 (m, 2H), 7.42 (d, J = 7.7 Hz, 2H), 7.37 – 7.34 (m, 1H), 7.30 – 7.27 (m, 1H), 7.19 (d, J = 7.9 Hz, 2H), 7.01 (d, J = 8.0 Hz, 2H), 6.87 (t, J = 4.8 Hz, 1H), 6.82 (s, 1H), 2.36 (s, 3H), 2.24 (s, 3H), 1.75 (d, J = 12.7 Hz, 3H); **^{13}C NMR (150 MHz, $CDCl_3$)** δ 157.9, 157.2, 155.4 (d, J = 78.0 Hz), 142.5 (d, J = 75.0 Hz), 140.8 (d, J = 13.5 Hz), 140.1, 137.9, 137.1, 136.6 (d, J = 21.0 Hz), 136.2 (d, J = 9.0 Hz), 132.5, 129.8, 129.2, 128.8, 128.8 (d, J = 7.5 Hz), 127.8, 126.9 (d, J = 9.0 Hz), 124.3, 122.4, 120.8, 117.1, 114.0, 111.3, 21.6, 21.2, 16.4 (d, J = 55.5 Hz); **^{31}P NMR (243 MHz, $CDCl_3$)** δ 42.3; **HRMS (ESI) :** calcd. for $C_{31}H_{26}N_3NaOP^+$ $[M+Na]^+$: 510.1706; found : 510.1704.

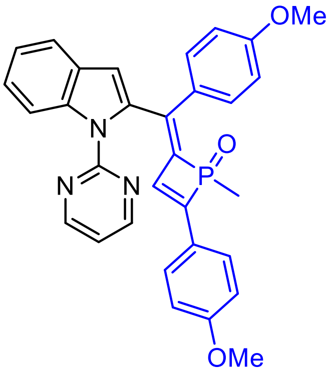

(*E*)-4-(4-methoxyphenyl)-2-((4-methoxyphenyl)(1-(pyrimidin-2-yl)-1*H*-indol-2-yl)methylene)-1-methyl-2*H*-phosphete 1-oxide (4b)

The title compound was isolated as a yellow solid (eluent: petroleum ether/ethyl acetate = 5/1, 100.8 mg, 97%). **^{1}H NMR (600 MHz, $CDCl_3$)** δ 8.56 – 8.50 (m, 2H), 8.30 (d,

J = 8.0 Hz, 1H), 7.69 – 7.56 (m, 2H), 7.51 – 7.45 (m, 4H), 7.37 – 7.33 (m, 1H), 7.30 – 7.27 (m, 1H), 6.93 – 6.88 (m, 3H), 6.81 (s, 1H), 6.75 – 6.71 (m, 2H), 3.82 (s, 3H), 3.73 (s, 3H), 1.74 (d, J = 12.6 Hz, 3H); **^{13}C NMR (150 MHz, CDCl$_3$)** δ 160.8, 159.3, 157.9, 157.2, 154.4 (d, J = 76.5 Hz), 141.8 (d, J = 76.5 Hz), 139.4 (d, J = 12.0 Hz), 137.1, 136.7 (d, J = 21.0 Hz), 131.8 (d, J = 9.0 Hz), 131.3, 129.2, 128.8, 128.5 (d, J = 9.0 Hz), 124.4 (d, J = 7.5 Hz), 124.2, 122.3, 120.7, 117.1, 114.6, 113.9, 113.8, 111.1, 55.4, 55.2, 16.3 (d, J = 55.5 Hz); **^{31}P NMR (243 MHz, CDCl$_3$)** δ 42.4; **HRMS (ESI) :** calcd. for $C_{31}H_{26}N_3NaO_3P^+$ [M+Na]$^+$: 542.1604; found : 542.1609.

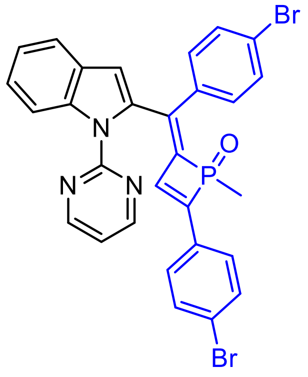

(*E*)-4-(4-bromophenyl)-2-((4-bromophenyl)(1-(pyrimidin-2-yl)-1*H*-indol-2-yl)methylene)-1-methyl-2*H*-phosphete 1-oxide (4c)

The title compound was isolated as a yellow solid (eluent: petroleum ether/ethyl acetate = 5/1, 108.6 mg, 88%). **^{1}H NMR (600 MHz, CDCl$_3$)** δ 8.52 (d, J = 4.8 Hz, 2H), 8.37 (d, J = 8.4 Hz, 1H), 7.77 (d, J = 67.1 Hz, 1H), 7.68 (d, J = 7.8 Hz, 1H), 7.51 (d, J = 8.1 Hz, 2H), 7.43 (d, J = 8.3 Hz, 2H), 7.38 – 7.35 (m, 3H), 7.33 (d, J = 8.4 Hz, 2H), 7.29 (t, J = 7.5 Hz, 1H), 6.91 (t, J = 4.8 Hz, 1H), 6.82 (s, 1H), 1.75 (d, J = 12.7 Hz, 3H); **^{13}C NMR (150 MHz, CDCl$_3$)** δ 157.9, 157.0, 155.0 (d, J = 78.0 Hz), 143.2 (d, J = 75.0 Hz), 142.2 (d, J = 12.0 Hz), 138.0 (d, J = 9.0 Hz), 137.2, 135.5 (d, J = 19.5 Hz), 132.7, 132.4, 131.7, 130.1 (d, J = 7.5 Hz), 129.4, 128.7, 128.3 (d, J = 9.0 Hz), 124.7, 124.2, 122.6, 122.5, 120.9, 117.2, 114.3, 111.9, 16.3 (d, J = 55.5 Hz); **^{31}P NMR (243 MHz, CDCl$_3$)** δ 41.9; **HRMS (ESI) :** calcd. for $C_{29}H_{20}Br_2N_3NaOP^+$ [M+Na]$^+$: 637.6903; found : 637.6904.

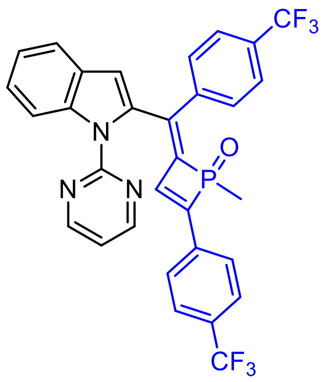

(*E*)-1-methyl-2-((1-(pyrimidin-2-yl)-1*H*-indol-2-yl)(4-(trifluoromethyl)phenyl)methylene)-4-(4-(trifluoromethyl)phenyl)-2*H*-phosphete 1-oxide (4d)

The title compound was isolated as a yellow solid (eluent:

petroleum ether/ethyl acetate = 5/1, 107.2 mg, 90%). **^{1}H NMR (600 MHz, $CDCl_3$)** δ 8.52 (d, *J* = 4.8 Hz, 2H), 8.41 (d, *J* = 8.4 Hz, 1H), 7.92 (d, *J* = 66.5 Hz, 1H), 7.72 – 7.68 (m, 3H), 7.66 (dd, *J* = 13.4, 8.7 Hz, 4H), 7.48 (d, *J* = 8.3 Hz, 2H), 7.40 (t, *J* = 7.8 Hz, 1H), 7.32 (t, *J* = 7.5 Hz, 1H), 6.92 (t, *J* = 4.8 Hz, 1H), 6.88 (s, 1H), 17.8 (d, *J* = 12.7 Hz, 3H). **^{13}C NMR (150 MHz, $CDCl_3$)** δ 157.93, 156.96, 155.4 (d, *J* = 78.0 Hz), 144.1 (d, *J* = 75.0 Hz), 143.8 (d, *J* = 12.0 Hz), 142.3 (d, *J* = 9.0 Hz), 137.3, 135.1 (d, *J* = 21.0 Hz), 134.4 (d, *J* = 6.0 Hz), 133.6, 131.5 (d, *J* = 31.5 Hz), 130.0 (d, *J* = 31.5 Hz), 128.6, 128.2, 127.2 (d, *J* = 9.0 Hz), 126.2 (q, *J* = 3.0 Hz), 125.5 (q, *J* = 3.0 Hz), 124.9, 124.7 (d, *J* = 24.0 Hz), 122.9 (d, *J* = 25.5 Hz), 122.8, 121.0, 117.2, 114.5, 112.3, 16.3 (d, *J* = 55.5 Hz); **^{31}P NMR (243 MHz, $CDCl_3$)** δ 41.9; **HRMS (ESI) :** calcd. for $C_{31}H_{20}F_6N_3NaOP^+$ [M+Na]$^+$: 618.1140; found : 618.1150.

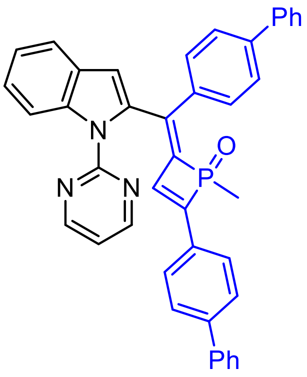

(*E*)-4-([1,1'-biphenyl]-4-yl)-2-([1,1'-biphenyl]-4-yl(1-(pyrimidin-2-yl)-1*H*-indol-2-yl)methylene)-1-methyl-2*H*-phosphete 1-oxide (4e)

The title compound was isolated as a yellow solid (eluent: petroleum ether/ethyl acetate = 5/1, 108.9 mg, 89%). **^{1}H NMR (600 MHz, $CDCl_3$)** δ 8.53 (d, *J* = 4.7 Hz, 2H), 8.36 (d, *J* = 8.4 Hz, 1H), 7.83 (d, *J* = 67.8 Hz, 1H), 7.71 (d, *J* = 7.8 Hz, 1H), 7.64 (d, *J* = 8.2 Hz, 4H), 7.61 (d, *J* = 9.0 Hz, 4H), 7.53 (d, *J* = 7.7 Hz, 2H), 7.48 (d, *J* = 8.1 Hz, 2H), 7.44 (t, *J* = 7.6 Hz, 2H), 7.40 (t, *J* = 7.8 Hz, 3H), 7.37 – 7.34 (m, 1H), 7.31 (t, *J* = 7.4 Hz, 2H), 6.88 (s, 1H), 6.86 (t, *J* = 4.8 Hz, 1H), 1.82 (d, *J* = 12.7 Hz, 3H); **^{13}C NMR (150 MHz, $CDCl_3$)** δ 157.9, 157.2, 155.4 (d, *J* = 78 Hz), 143.2 (d, *J* = 75.0 Hz) 142.5, 141.7 (d, *J* = 12.0 Hz), 140.5, 140.3, 140.1, 138.0 (d, *J* = 9.0 Hz), 137.2, 136.3 (d, *J* = 19.5 Hz), 132.7, 130.4 (d, *J* = 6.0 Hz), 128.9, 128.8, 128.8, 128.3, 127.9, 127.8, 127.5, 127.5, 127.5, 127.0, 126.8, 124.5, 122.5, 120.8, 117.1, 114.1, 111.6, 16.5 (d, *J* = 54.0 Hz); **^{31}P NMR (243 MHz, $CDCl_3$)** δ 42.4; **HRMS (ESI) :** calcd. for $C_{41}H_{30}N_3NaOP^+$ [M+Na]$^+$: 634.2019; found : 634.2027.

(*E*)-1-methyl-2-((1-(pyrimidin-2-yl)-1*H*-indol-2-yl)(*m*-tolyl)methylene)-4-(*m*-tolyl)

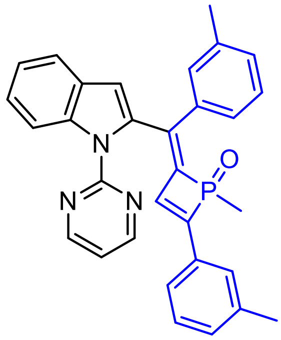

-2H-phosphete 1-oxide (4f)

The title compound was isolated as a yellow solid (eluent: petroleum ether/ethyl acetate = 5/1, 87.8 mg, 90%). **^{1}H NMR (600 MHz, CDCl$_3$)** δ 8.50 (d, *J* = 4.8 Hz, 2H), 8.32 (d, *J* = 8.3 Hz, 1H), 7.74 (d, *J* = 64.0 Hz, 1H), 7.67 (d, *J* = 3.7 Hz, 1H), 7.43 (d, *J* = 7.9 Hz, 1H), 7.35 – 7.30 (m, 4H), 7.29 – 7.23 (m, 2H), 7.14 – 7.09 (m, 2H), 6.94 (d, *J* = 7.5 Hz, 1H), 6.84 (t, *J* = 4.8 Hz, 1H), 6.82 (s, 1H), 2.33 (s, 3H), 2.21 (s, 3H), 1.75 (d, *J* = 12.7 Hz, 3H); **^{13}C NMR (150 MHz, CDCl$_3$)** δ 157.9, 157.2, 156.0 (d, *J* = 78.0 Hz), 143.1 (d, *J* = 75.0 Hz), 141.6 (d, *J* = 12.0 Hz), 139.0 (d, *J* = 9.0 Hz), 138.9, 137.9, 137.2, 136.5 (d, *J* = 19.5 Hz), 133.0, 131.4 (d, *J* = 6.0 Hz), 130.7, 129.0, 129.0, 128.8, 128.5, 128.4, 127.6 (d, *J* = 9.0 Hz), 125.1, 124.3, 124.1 (d, *J* = 9.0 Hz), 122.4, 120.8, 117.2, 114.0, 111.4, 21.3, 16.5 (d, *J* = 55.5 Hz); **^{31}P NMR (243 MHz, CDCl$_3$)** δ 42.3; **HRMS (ESI) :** calcd. for $C_{31}H_{26}N_3NaOP^+$ $[M+Na]^+$: 510.1706; found : 510.1708.

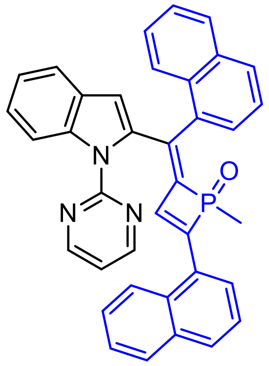

(*E*)-1-methyl-4-(naphthalen-1-yl)-2-(naphthalen-1-yl(1-(pyrimidin-2-yl)-1*H*-indol-2-yl)methylene)-2*H*-phosphete 1-oxide (4g)

The title compound was isolated as a yellow solid (eluent: petroleum ether/ethyl acetate = 5/1, 89.5 mg, 80%). **^{1}H NMR (600 MHz, CDCl$_3$)** δ 8.65 (d, *J* = 4.8 Hz, 2H), 8.43 (d, *J* = 8.5 Hz, 1H), 8.31 (d, *J* = 8.5 Hz, 1H), 8.17 (d, *J* = 8.4 Hz, 1H), 7.91 – 7.78 (m, 6H), 7.63 (dt, *J* = 7.2, 1.4 Hz, 1H), 7.59 – 7.56 (m, 2H), 7.52 (t, *J* = 7.2 Hz, 1H), 7.48 (t, *J* = 7.6 Hz, 1H), 7.45 (t, *J* = 7.7 Hz, 1H), 7.41 (t, *J* = 7.1 Hz, 1H), 7.37 – 7.34 (m, 1H), 7.33 – 7.30 (m, 1H), 7.23 (t, *J* = 7.4 Hz, 1H), 6.94 (t, *J* = 4.8 Hz, 1H), 6.78 (s, 1H), 1.50 (d, *J* = 12.7 Hz, 3H); **^{13}C NMR (150 MHz, CDCl$_3$)** δ 158.2, 158.0, 157.3 (d, *J* = 73.5 Hz), 144.4, 143.1 (d, *J* = 12.0 Hz), 138.7 (d, *J* = 19.5 Hz), 138.5, 136.0 (d, *J* = 10.5 Hz), 134.0 (d, *J* = 13.5 Hz), 131.8, 130.9, 130.9, 130.6, 130.4, 129.4, 129.3, 128.9, 128.8, 128.1, 127.4, 127.0 (d, *J* = 9.0 Hz), 126.8, 126.4, 126.2, 125.8, 125.42, 125.3, 125.1, 124.7, 122.6, 121.2, 117.5, 113.2, 16.5 (d, *J* = 55.5 Hz); **^{31}P NMR (243 MHz, CDCl$_3$)**

δ 45.3; **HRMS (ESI) :** calcd. for $C_{37}H_{26}N_3NaOP^+$ $[M+Na]^+$: 582.1706; found : 582.1711.

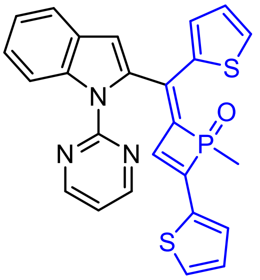

(*Z*)-1-methyl-2-((1-(pyrimidin-2-yl)-1*H*-indol-2-yl)(thiophen-2-yl)methylene)-4-(thiophen-2-yl)-2*H*-phosphete 1-oxide (4h)

The title compound was isolated as a yellow solid (eluent: petroleum ether/ethyl acetate = 5/1, 88.6 mg, 94%). **^{1}H NMR (600 MHz, $CDCl_3$)** δ 8.58 (d, *J* = 4.8 Hz, 2H), 8.37 (d, *J* = 8.3 Hz, 1H), 7.69 (d, *J* = 7.9 Hz, 1H), 7.40 – 7.30 (m, 4H), 7.28 (d, *J* = 6.3 Hz, 1H), 7.17 (d, *J* = 5.1 Hz, 1H), 7.14 (d, *J* = 3.7 Hz, 1H), 7.05 (t, *J* = 4.4 Hz, 1H), 6.94 (t, *J* = 4.8 Hz, 1H), 6.88 (s, 1H), 6.85 (t, *J* = 2.7 Hz, 1H), 1.96 (d, *J* = 12.8 Hz, 3H); **^{13}C NMR (150 MHz, $CDCl_3$)** δ 158.0, 157.3, 148.4 (d, *J* = 76.5 Hz), 142.8 (d, *J* = 9.0 Hz), 141.6 (d, *J* = 75.0 Hz), 139.1 (d, *J* = 10.5 Hz), 137.1, 135.6 (d, *J* = 19.5 Hz), 134.4 (d, *J* = 9.0 Hz), 128.7, 128.7, 128.5 (d, *J* = 6.0 Hz), 128.4, 127.8, 127.5, 126.6, 125.5, 124.5, 122.5, 121.0, 117.3, 114.1, 111.1, 16.7 (d, *J* = 57.0 Hz); **^{31}P NMR (243 MHz, $CDCl_3$)** δ 40.7; **HRMS (ESI) :** calcd. for $C_{25}H_{18}N_3NaOPS_2^+$ $[M+Na]^+$: 494.0521; found : 494.0522.

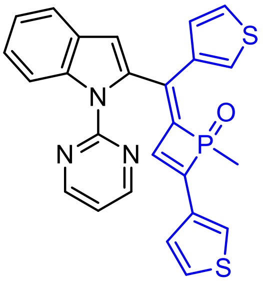

(*E*)-1-methyl-2-((1-(pyrimidin-2-yl)-1*H*-indol-2-yl)(thiophen-3-yl)methylene)-4-(thiophen-3-yl)-2*H*-phosphete 1-oxide (4i)

The title compound was isolated as a yellow solid (eluent: petroleum ether/ethyl acetate = 5/1, 80.2 mg, 85%). **^{1}H NMR (600 MHz, $CDCl_3$)** δ 8.56 (d, *J* = 4.8 Hz, 2H), 8.32 (d, *J* = 8.3 Hz, 1H), 7.68 (d, *J* = 7.8 Hz, 1H), 7.59 – 7.46 (m, 2H), 7.43 (d, *J* = 4.7 Hz, 1H), 7.39 – 7.34 (m, 3H), 7.28 (t, *J* = 7.5 Hz, 1H), 7.20 (d, *J* = 5.1 Hz, 1H), 7.14 (dd, *J* = 5.1, 3.0 Hz, 1H), 6.93 (t, *J* = 4.8 Hz, 1H), 6.82 (s, 1H), 1.80 (d, *J* = 12.7 Hz, 3H); **^{13}C NMR (150 MHz, $CDCl_3$)** δ 157.9, 157.3, 149.7 (d, *J* = 76.5 Hz), 141.8 (d, *J* = 75.0 Hz), 140.5 (d, *J* = 9.0 Hz), 140.3 (d, *J* = 12.0 Hz), 137.1, 136.3 (d, *J* = 19.5 Hz), 133.0 (d, *J* = 6.0 Hz), 128.7, 127.3, 127.1, 127.1, 125.9, 125.4, 125.4 (d, *J* = 16.5 Hz), 124.4, 124.3, 122.4, 120.8,

117.2, 113.9, 110.7, 16.2 (d, J = 55.5 Hz); **^{31}P NMR (243 MHz, $CDCl_3$)** δ 42.4; **HRMS (ESI) :** calcd. for $C_{25}H_{18}N_3NaOPS_2^+$ [M+Na]$^+$: 494.0521; found : 494.0520.

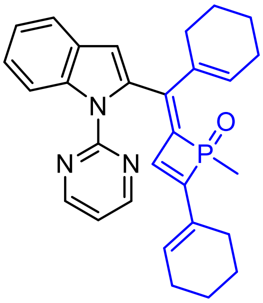

(*E*)-4-(cyclohex-1-en-1-yl)-2-(cyclohex-1-en-1-yl(1-(pyrimidin-2-yl)-1*H*-indol-2-yl)methylene)-1-methyl-2*H*-phosphete 1-oxide (4j)

The title compound was isolated as a yellow solid (eluent: petroleum ether/ethyl acetate = 5/1, 82.3 mg, 88%). **^{1}H NMR (600 MHz, $CDCl_3$)** δ 8.68 (d, J = 4.8 Hz, 2H), 8.23 (dq, J = 8.4, 0.9 Hz, 1H), 7.61 (d, J = 7.6 Hz, 1H), 7.31 – 7.27 (m, 1H), 7.24 – 7.21 (m, 1H), 7.09 – 6.97 (m, 2H), 6.64 (s, 1H), 6.27 – 6.22 (m, 1H), 5.51 (t, J = 4.3 Hz, 1H), 2.63 – 2.57 (m, 1H), 2.26 – 2.19 (m, 3H), 2.09 – 2.01 (m, 2H), 1.86 (d, J = 12.5 Hz, 3H), 1.83 – 1.77 (m, 2H), 1.66 – 1.58 (m, 4H), 1.52 – 1.48 (m, 2H), 1.45 – 1.41 (m, 1H), 1.36 – 1.29 (m, 1H); **^{13}C NMR (150 MHz, $CDCl_3$)** δ 157.9, 157.6, 156.5 (d, J = 78.0 Hz) , 141.0 (d, J = 73.5 Hz), 139.1 (d, J = 13.5 Hz), 136.7 (d, J = 21.0 Hz), 136.7 (d, J = 7.5 Hz) 136.6, 133.8 (d, J = 7.5 Hz), 133.6, 131.2 (d, J = 3.0 Hz), 130.6 (d, J = 1.5.0 Hz), 128.8, 123.7, 122.1, 120.5, 117.2, 113.7, 109.6, 27.6, 26.1, 25.9, 24.8 (d, J = 7.5 Hz), 22.9, 22.0, 21.9, 21.7, 17.4 (d, J = 55.5 Hz); **^{31}P NMR (243 MHz, $CDCl_3$)** δ 41.0; **HRMS (ESI) :** calcd. for $C_{29}H_{30}N_3NaOP^+$ [M+Na]$^+$: 490.2019; found : 490.2028.

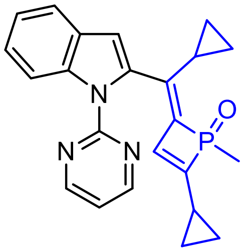

(*E*)-4-cyclopropyl-2-(cyclopropyl(1-(pyrimidin-2-yl)-1*H*-indol-2-yl)methylene)-1-methyl-2*H*-phosphete 1-oxide (4k)

The title compound was isolated as a yellow oil (eluent: petroleum ether/ethyl acetate = 5/1, 55.8 mg, 72%). **^{1}H NMR (600 MHz, $CDCl_3$)** δ 8.76 (d, J = 4.8 Hz, 2H), 8.26 (d, J = 8.3 Hz, 1H), 7.58 (d, J = 7.7 Hz, 1H), 7.29 (t, J = 7.8 Hz, 1H), 7.22 (t, J = 7.4 Hz, 1H), 7.14 (t, J = 4.8 Hz, 1H), 6.86 (d, J = 69.7 Hz, 1H), 6.55 (s, 1H), 1.80 – 1.76 (m, 4H), 1.63 – 1.55 (m, 1H), 0.96 – 0.93(m, 2H), 0.82 – 0.79 (m, 1H), 0.77 – 0.73 (m, 1H), 0.70 – 0.63 (m, 3H), 0.56 – 0.52 (m, 1H); **^{13}C NMR (150 MHz, $CDCl_3$)** δ 162.6 (d, J = 73.5 Hz), 158.1, 157.8, 141.7 (d, J = 76.5 Hz), 141.1 (d, J = 15.0 Hz), 136.9, 136.2

(d, J = 21.0 Hz), 130.5, 128.9, 123.8, 122.2, 120.5, 117.3, 113.6, 109.0, 16.7 (d, J = 54.0 Hz), 16.4 (d, J = 12.0 Hz), 10.4 (d, J = 6.0 Hz), 9.1 (d, J = 3.0 Hz), 9.0 (d, J = 3.0 Hz), 6.9, 6.3; **^{31}P NMR (243 MHz, CDCl$_3$)** δ 42.9; **HRMS (ESI) :** calcd. for $C_{23}H_{22}N_3NaOP^+$ $[M+Na]^+$: 410.1393; found : 410.1395.

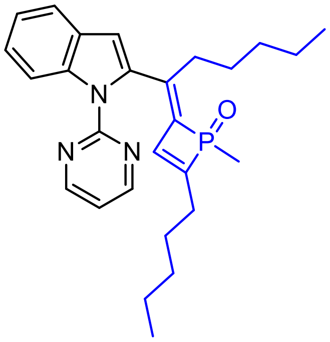

(*E*)-1-methyl-4-pentyl-2-(1-(1-(pyrimidin-2-yl)-1*H*-indol-2-yl)hexylidene)-2*H*-phosphete 1-oxide (4l)

The title compound was isolated as a yellow oil (eluent: petroleum ether/ethyl acetate = 5/1, 72.5 mg, 81%). **^{1}H NMR (600 MHz, CDCl$_3$)** 8.76 (d, J = 4.8 Hz, 2H), 8.27 (d, J = 8.3 Hz, 1H), 7.60 (d, J = 7.7 Hz, 1H), 7.31 – 7.27 (m, 1H), 7.25 – 7.22 (m, 1H), 7.13 (t, J = 4.8 Hz, 1H), 6.93 (d, J = 71.9 Hz, 1H), 6.65 (s, 1H), 2.50 – 2.41 (m, 2H), 2.36 – 2.30 (m, 2H), 1.76 (d, J = 12.5 Hz, 3H), 1.58 – 1.53 (m, 1H), 1.51 – 1.43 (m, 3H), 1.31 – 1.21 (m, 8H), 0.88 (t, J = 7.0 Hz, 3H), 0.81 (t, J = 7.1 Hz, 3H); **^{13}C NMR (150 MHz, CDCl$_3$)** δ 161.0 (d, J = 72.0 Hz), 158.2, 157.9, 143.9 (d, J = 16.5 Hz), 141.2 (d, J = 76.5 Hz), 137.6, 137.5 (d, J = 21.0 Hz), 130.6, 129.1, 123.9, 122.3, 120.6, 117.3, 113.6, 109.0, 36.4 (d, J = 10.5 Hz), 31.8, 31.5, 28.4 (d, J = 1.5 Hz), 28.3 (d, J = 6.0 Hz), 27.2 (d, J = 7.5 Hz), 22.4, 22.3, 16.4 (d, J = 52.5 Hz), 14.0, 13.9; **^{31}P NMR (243 MHz, CDCl$_3$)** δ 43.9; **HRMS (ESI) :** calcd. for $C_{27}H_{34}N_3NaOP^+$ $[M+Na]^+$: 470.2332; found : 470.2330.

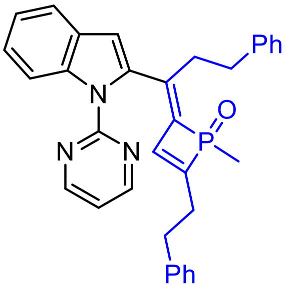

(*E*)-1-methyl-4-phenethyl-2-(3-phenyl-1-(1-(pyrimidin-2-yl)-1*H*-indol-2-yl)propylidene)-2*H*-phosphete 1-oxide (4m)

The title compound was isolated as a yellow oil (eluent: petroleum ether/ethyl acetate = 5/1, 81.5 mg, 79%). **^{1}H NMR (600 MHz, CDCl$_3$)** δ 8.73 (d, J = 4.8 Hz, 2H), 8.33 (dd, J = 8.3, 1.0 Hz, 1H), 7.63 (dt, J = 7.7, 1.0 Hz, 1H), 7.34 – 7.31 (m, 1H), 7.29 – 7.25 (m, 3H), 7.23 – 7.18 (m, 3H), 7.16 – 7.12 (m, 5H), 7.10 (t, J = 4.8 Hz, 1H), 7.01 (dt, J = 70.8, 1.5 Hz, 1H), 6.66 (s, 1H), 2.90 – 2.85 (m, 1H), 2.81 – 7.77 (m, 2H), 2.75 – 2.66 (m, 5H), 1.48 (d, J = 12.5 Hz, 3H); **^{13}C NMR (150 MHz, CDCl$_3$)** δ 160.3 (d, J = 70.5

Hz), 158.3, 157.9, 144.6 (d, J = 15.0 Hz), 142.0, 141.6, 140.5, 137.6, 137.1 (d, J =22.5 Hz), 130.0, 129.0, 128.6, 128.6, 128.3, 128.3, 126.4, 126.0, 124.1, 122.5, 120.7, 117.4, 113.9, 109.7, 38.8 (d, J = 10.5 Hz), 35.0 (d, J = 1.5 Hz), 33.6 (d, J = 6.0 Hz), 29.9 (d, J = 6.0 Hz), 15.8 (d, J = 54.0 Hz); **^{31}P NMR (243 MHz, CDCl$_3$)** δ 44.4; **HRMS (ESI) :** calcd. for $C_{33}H_{30}N_3NaOP^+$ [M+Na]$^+$: 538.2019; found : 538.2017.

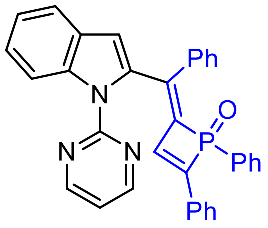

(*E*)-1,4-diphenyl-2-(phenyl(1-(pyrimidin-2-yl)-1*H*-indol-2-yl)methylene)-2*H*-phosphete 1-oxide (4n)

The title compound was isolated as a yellow solid (eluent: petroleum ether/ethyl acetate = 5/1, 97.0 mg, 93%). **^{1}H NMR (600 MHz, CDCl$_3$)** δ 8.45 (d, J = 4.8 Hz, 2H), 8.36 (dd, J = 8.3, 1.0 Hz, 1H), 8.10 (d, J = 70.0 Hz, 1H), 7.90 – 7.84 (m, 2H), 7.72 (d, J = 7.7 Hz, 1H), 7.52 – 7.49 (m, 1H), 7.48 – 7.45 (m, 2H), 7.45 – 7.41 (m, 2H), 7.39 – 7.36 (m, 1H), 7.35 – 7.28 (m, 6H), 7.07 – 7.01 (m, 3H), 6.92 (s, 1H), 6.85 (t, J = 4.8 Hz, 1H); **^{13}C NMR (150 MHz, CDCl$_3$)** δ 157.9, 157.1, 155.4 (d, J = 79.5 Hz), 145.0 (d, J = 12.0 Hz), 142.8 (d, J = 76.5 Hz), 138.5 (d, J = 9.0 Hz), 137.1, 136.5 (d, J = 21.0 Hz), 133.0, 132.4 (d, J = 3.0 Hz), 131.3 (d, J = 10.5 Hz), 131.1 (d, J = 6.0 Hz), 130.6, 129.8, 129.0, 128.9, 128.9, 128.2, 128.0, 127.9, 127.2 (d, J = 9.0 Hz), 124.4, 122.5, 120.9, 117.0, 114.1, 111.3; **^{31}P NMR (243 MHz, CDCl$_3$)** δ 34.9; **HRMS (ESI) :** calcd. for $C_{34}H_{24}N_3NaOP^+$ [M+Na]$^+$: 544.1549; found : 544.1552.

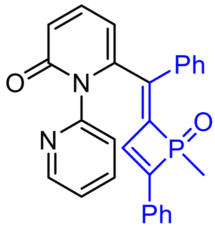

(*E*)-6-((1-methyl-1-oxido-4-phenyl-2*H*-phosphet-2-ylidene)(phenyl)methyl)-2*H*-[1,2'-bipyridin]-2-one (6a)

The title compound was isolated as a yellow solid (eluent: dichloromethane/methanol = 50/1, 80.3 mg, 92%). **^{1}H NMR (600 MHz, CDCl$_3$)** δ 8.35 (d, J = 3.9 Hz, 1H), 7.72 (d, J = 65.4 Hz, 1H), 7.57 (d, J = 7.7 Hz, 2H), 7.52 (dd, J = 9.4, 6.7 Hz, 1H), 7.46 – 7.35 (m, 4H), 7.22 (s, 5H), 7.08 (s, 1H), 7.05 – 6.75 (m, 1H), 6.73 (dd, J = 9.4, 1.2 Hz, 1H), 6.34 (d, J = 6.7 Hz, 1H), 1.59 (d, J = 12.6 Hz, 3H); **^{13}C NMR (150 MHz, CDCl$_3$)** δ 163.1, 158.5 (d, J = 76.5 Hz), 150.9, 148.9, 145.6, 140.6, 139.6, 137.01, 136.6 (d, J = 7.5 Hz), 130.8 (d, J = 6.0 Hz), 130.5, 129.2, 128.7, 127.4, 127.3, 127.2, 124.0, 123.5, 121.6, 109.0, 16.2; **^{31}P NMR (243

MHz, CDCl3) δ 42.2; **HRMS (ESI) :** calcd. for $C_{27}H_{21}N_2NaO_2P^+$ $[M+Na]^+$: 459.1233; found : 459.1237.

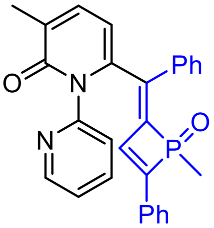

(*E*)-3-methyl-6-((1-methyl-1-oxido-4-phenyl-2*H*-phosphet-2-ylidene)(phenyl)methyl)-2*H*-[1,2'-bipyridin]-2-one (6b)

The title compound was isolated as a yellow solid (eluent: dichloromethane/methanol = 50/1, 79.3 mg, 88%). **^{1}H NMR (600 MHz, CDCl3)** δ 8.35 (d, *J* = 4.0 Hz, 1H), 7.71 (d, *J* = 69.2 Hz, 1H), 7.58 – 7.55 (m, 2H), 7.46 – 7.37 (m, 5H), 7.22 (s, 5H), 7.08 (s, 1H), 6.81 (s, 1H), 6.27 (d, *J* = 6.8 Hz, 1H), 2.25 (s, 3H), 1.58 (d, *J* = 12.8 Hz, 3H); **^{13}C NMR (150 MHz, CDCl3)** δ 163.55, 158.0 (d, *J* = 78.0 Hz), 148.8, 140.9, 136.9 (d, *J* = 7.5 Hz), 130.9 (d, *J* = 6.0 Hz), 130.4, 129.2, 128.7, 127.4, 127.3, 127.3, 124.0, 123.3, 108.8, 17.2; **^{31}P NMR (243 MHz, CDCl3)** δ 42.0; **HRMS (ESI) :** calcd. for $C_{28}H_{23}N_2NaO_2P^+$ $[M+Na]^+$: 473.1389; found : 473.1382.

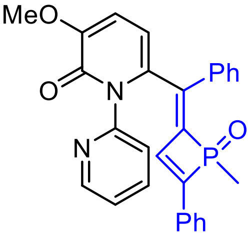

(*E*)-3-methoxy-6-((1-methyl-1-oxido-4-phenyl-2*H*-phosphet-2-ylidene)(phenyl)methyl)-2*H*-[1,2'-bipyridin]-2-one (6c)

The title compound was isolated as a yellow solid (eluent: dichloromethane/methanol = 50/1, 84.0 mg, 90%). **^{1}H NMR (600 MHz, CDCl3)** δ 8.34 (d, *J* = 3.8 Hz, 1H), 7.73 (d, *J* = 64.4 Hz, 1H), 7.57 – 7.55 (m, 2H), 7.45 – 7.35 (m, 4H), 7.18 (s, 5H), 7.06 (s, 1H), 6.96 –6.74 (m, 2H), 6.29 (d, *J* = 7.5 Hz, 1H), 3.92 (s, 3H), 1.60 (d, *J* = 12.7 Hz, 3H); **^{13}C NMR (150 MHz, CDCl3)** δ 158.6, 157.8 (d, *J* = 76.5 Hz), 150.9, 150.3, 148.7, 140.9, 137.2 (d, *J* = 9.0 Hz), 136.9, 131.0 (d, *J* = 6.0 Hz), 130.3, 129.2, 128.6, 128.6, 127.3, 127.3, 127.2, 124.2, 123.4, 111.9, 108.4, 56.1, 16.2; **^{31}P NMR (243 MHz, CDCl3)** δ 41.9; **HRMS (ESI) :** calcd. for $C_{28}H_{23}N_2NaO_3P^+$ $[M+Na]^+$: 489.1339; found : 489.1334.

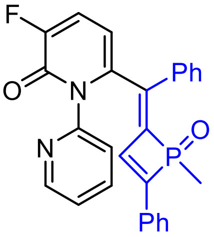

(*E*)-3-fluoro-6-((1-methyl-1-oxido-4-phenyl-2*H*-phosphet-2-ylidene)(phenyl)methyl)-2*H*-[1,2'-bipyridin]-2-one (6d)

The title compound was isolated as a yellow solid (eluent: dichloromethane/methanol = 50/1, 77.3 mg, 85%). **^{1}H NMR (600 MHz, CDCl$_3$)** δ 8.34 (d, *J* = 3.9 Hz, 1H), 7.70 (d, *J* = 67.6 Hz, 1H), 7.55 (d, *J* = 7.5 Hz, 2H), 7.45 – 7.37 (m, 4H), 7.31 – 7.27 (m, 2H), 7.19 (s, 5H), 7.08 (s, 1H), 6.84 (s, 1H), 6.28 (dd, *J* = 7.5, 4.3 Hz, 1H), 1.59 (d, *J* = 12.7 Hz, 3H); **^{13}C NMR (150 MHz, CDCl$_3$)** δ 158.8 (d, *J* = 78.0 Hz), 156.9 (d, *J* = 25.5 Hz), 153.1, 151.5, 150.1, 148.9, 141.0, 140.5 (d, *J* = 12.0 Hz), 137.2, 136.6 (d, *J* = 7.5 Hz), 130.8 (d, *J* = 6.0 Hz), 130.6, 129.2, 128.8, 128.8, 127.4, 127.4, 127.2, 124.0, 123.8, 119.9, 16.1; **^{31}P NMR (243 MHz, CDCl$_3$)** δ 42.1; **HRMS (ESI) :** calcd. for $C_{27}H_{20}FN_2NaO_2P^+$ $[M+Na]^+$: 477.1139; found : 477.1140.

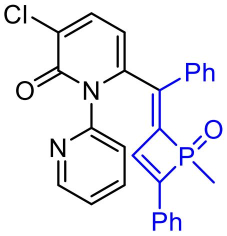

(*E*)-3-chloro-6-((1-methyl-1-oxido-4-phenyl-2*H*-phosphet-2-ylidene)(phenyl)methyl)-2*H*-[1,2'-bipyridin]-2-one (6e)

The title compound was isolated as a yellow solid (eluent: dichloromethane/methanol = 50/1, 80.1 mg, 85%). **^{1}H NMR (600 MHz, CDCl$_3$)** δ 8.31 (s, 1H), 7.79 – 7.62 (m, 2H), 7.54 (d, *J* = 7.0 Hz, 2H), 7.45 – 7.34 (m, 4H), 7.29 (s, 1H), 7.20 (s, 4H), 7.05 (s, 1H), 6.98 – 6.68 (m, 1H), 6.32 (d, *J* = 7.4 Hz, 1H), 1.58 (d, *J* = 12.7 Hz, 3H); **^{13}C NMR (150 MHz, CDCl$_3$)** δ 159.4, 159.0 (d, *J* = 76.5 Hz), 150.5, 148.8, 144.3, 140.5 (d, *J* = 12.0 Hz), 137.5, 137.2, 136.5 (d, *J* = 7.5 Hz), 130.7 (d, *J* = 6.0 Hz), 130.6, 129.2, 128.9, 128.8, 127.5, 127.4, 127.2, 126.8, 123.9, 123.8, 108.4, 16.2; **^{31}P NMR (243 MHz, CDCl$_3$)** δ 42.3; **HRMS (ESI) :** calcd. for $C_{27}H_{20}ClN_2NaO_2P^+$ $[M+Na]^+$: 493.0843; found : 493.0836.

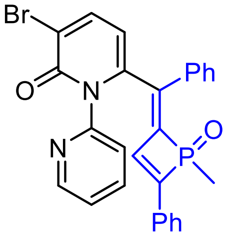

(*E*)-3-bromo-6-((1-methyl-1-oxido-4-phenyl-2*H*-phosphet-2-ylidene)(phenyl)methyl)-2*H*-[1,2'-bipyridin]-2-one (6f)

The title compound was isolated as a yellow solid (eluent: dichloromethane/methanol = 50/1, 86.4 mg, 84%). **^{1}H NMR (600 MHz, CDCl$_3$)** δ 8.34 (d, *J* = 3.9 Hz, 1H), 7.94 (d, *J* = 7.4 Hz, 1H), 7.70 (d, *J* = 68.6 Hz, 1H), 7.57 – 7.55 (m, 2H), 7.45 – 7.41 (m, 3H), 7.40 – 7.32 (m, 1H), 7.21 (s, 5H), 7.09 (s, 1H), 7.02 – 6.67 (s, 1H), 6.26 (d, *J* = 7.3 Hz, 1H), 1.59 (d, *J* = 12.8 Hz, 3H); **^{13}C NMR (150 MHz, CDCl$_3$)** δ 159.4, 159.0 (d, *J* = 78.0 Hz), 150.8, 148.8, 141.5,

140.4, 137.1, 136.4 (d, *J* = 17.5 Hz), 136.4, 130.8 (d, *J* = 6.0 Hz), 130.7, 129.3, 128.9, 128.8, 127.4, 127.4, 127.2, 123.9, 123.8, 117.2, 109.0, 16.3. **^{31}P NMR (243 MHz, CDCl$_3$)** δ 42.3; **HRMS (ESI) :** calcd. for $C_{27}H_{20}BrN_2NaO_2P^+$ [M+Na]$^+$: 537.0338; found : 537.0333.

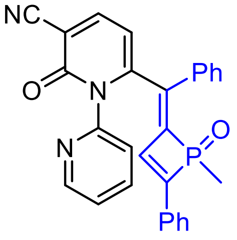

(*E*)-6-((1-methyl-1-oxido-4-phenyl-2*H*-phosphet-2-ylidene)(phenyl)methyl)-2-oxo-2*H*-[1,2'-bipyridine]-3-carbonitrile (6g)

The title compound was isolated as a yellow solid (eluent: dichloromethane/methanol = 50/1, 72.0 mg, 78%). **^{1}H NMR (600 MHz, CDCl$_3$)** δ 8.33 (d, *J* = 3.8 Hz, 1H), 8.02 (d, *J* = 7.3 Hz, 1H), 7.69 (d, *J* = 64.7 Hz, 1H), 7.58 – 7.54 (m, 2H), 7.47 – 7.37 (m, 4H), 7.25 – 7.13 (m, 5H), 7.11 (s, 1H), 6.83 (s, 1H), 6.46 (d, *J* = 7.3 Hz, 1H), 1.59 (d, *J* = 12.8 Hz, 3H); **^{13}C NMR (150 MHz, CDCl$_3$)** δ 160.4 (d, *J* = 76.5 Hz), 160.2, 151.7, 149.7, 149.7, 149.0, 147.3, 139.9 (d, *J* = 12.0 Hz), 137.4, 135.8 (d, *J* = 9.0 Hz), 131.0, 130.5 (d, *J* = 6.0 Hz), 129.3, 129.2, 129.0, 127.6 (d, *J* = 9.0 Hz), 127.2, 124.2, 123.8, 115.3, 108.6, 106.0, 16.0; **^{31}P NMR (243 MHz, CDCl$_3$)** δ 42.6; **HRMS (ESI) :** calcd. for $C_{28}H_{20}N_3NaO_2P^+$ [M+Na]$^+$: 484.1185; found : 484.1186.

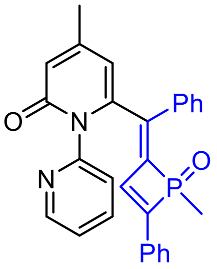

(*E*)-4-methyl-6-((1-methyl-1-oxido-4-phenyl-2*H*-phosphet-2-ylidene)(phenyl)methyl)-2*H*-[1,2'-bipyridin]-2-one (6h)

The title compound was isolated as a yellow solid (eluent: dichloromethane/methanol = 50/1, 83.8 mg, 93%). **^{1}H NMR (600 MHz, CDCl$_3$)** δ 8.35 (d, *J* = 4.0 Hz, 1H), 7.75 (d, *J* = 61.1 Hz, 1H), 7.58 (d, *J* = 7.5 Hz, 2H), 7.45 – 7.39 (m, 3H), 7.22 (s, 6H), 7.07 (s, 1H), 6.75 (s, 1H), 6.55 (s, 1H), 6.18 (s, 1H), 2.32 (s, 3H), 1.60 (s, 3H); **^{13}C NMR (150 MHz, CDCl$_3$)** δ 163.2, 158.5 (d, *J* = 78.0 Hz), 151.0, 148.8, 140.7, 137.0, 136.7 (d, *J* = 9.0 Hz), 130.9 (d, *J* = 6.0 Hz), 130.5, 129.2, 128.7, 127.4, 127.3, 124.1, 123.4, 119.8, 111,6, 21.6, 16.2; **^{31}P NMR (243 MHz, CDCl$_3$)** δ 42.2; **HRMS (ESI) :** calcd. for $C_{28}H_{23}N_2NaO_2P^+$ [M+Na]$^+$: 473.1389; found : 473.1387.

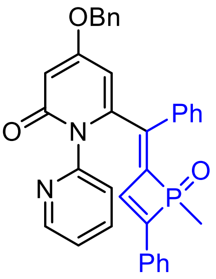

(*E*)-4-(benzyloxy)-6-((1-methyl-1-oxido-4-phenyl-2*H*-phosphet-2-ylidene)(phenyl)methyl)-2*H*-[1,2'-bipyridin]-2-one (6i)

The title compound was isolated as a yellow solid (eluent: dichloromethane/methanol = 50/1, 96.6 mg, 89%). **^{1}H NMR (600 MHz, CDCl$_3$)** δ 8.32 (d, *J* = 3.8 Hz, 1H), 7.76 (d, *J* = 68.8 Hz, 1H), 7.57 (d, *J* = 7.6 Hz, 2H), 7.49 – 7.35 (m, 9H), 7.21 (s, 5H), 7.05 (s, 1H), 6.74 (s, 1H), 6.16 (s, 2H), 5.11 (s, 2H), 1.57 (d, *J* = 10.8 Hz, 3H); **^{13}C NMR (150 MHz, CDCl$_3$)** δ 164.7, 158.5 (d, *J* = 78.0 Hz), 148.8, 140.6, 136.9, 136.4 (d, *J* = 9.0 Hz), 135.0, 130.8 (d, *J* = 6.0 Hz), 130.6, 129.2, 128.8, 128.8, 128.7, 127.9, 127.4 (d, *J* = 9.0 Hz), 127.3, 124.4, 123.4, 104.7, 98.2, 70.6, 16.0; **^{31}P NMR (243 MHz, CDCl$_3$)** δ 42.4; **HRMS (ESI) :** calcd. for $C_{34}H_{27}N_2NaO_3P^+$ $[M+Na]^+$: 565.1652; found : 565.1649.

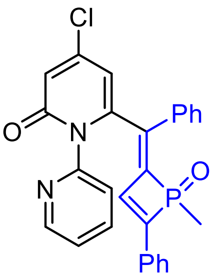

(*E*)-4-chloro-6-((1-methyl-1-oxido-4-phenyl-2*H*-phosphet-2-ylidene)(phenyl)methyl)-2*H*-[1,2'-bipyridin]-2-one (6j)

The title compound was isolated as a yellow solid (eluent: dichloromethane/methanol = 50/1, 85.7 mg, 91%). **^{1}H NMR (600 MHz, CDCl$_3$)** δ 8.35 (d, *J* = 3.9 Hz, 1H), 7.75 (d, *J* = 66.4 Hz, 1H), 7.59 (d, *J* = 7.1 Hz, 2H), 7.46 – 7.40 (m, 3H), 7.22 (s, 6H), 7.09 (s, 1H), 7.00 – 6.63 (m, 2H), 6.37 (d, *J* = 2.2 Hz, 1H), 1.59 (s, 3H); **^{13}C NMR (150 MHz, CDCl$_3$)** δ 162.1, 159.4 (d, *J* = 76.5 Hz), 150.0, 149.0, 146.8, 146.0, 140.1, 137.1, 136.1 (d, *J* = 9.0 Hz), 130.8, 130.7 (d, *J* = 6.0 Hz), 129.3, 9.0, 128.9, 127.5 (d, *J* = 9.0 Hz), 127.2, 124.0, 123.7, 119.7, 110.5, 16.2; **^{31}P NMR (243 MHz, CDCl$_3$)** δ 42.3; **HRMS (ESI) :** calcd. for $C_{27}H_{20}ClN_2NaO_2P^+$ $[M+Na]^+$: 493.0843; found : 493.0844.

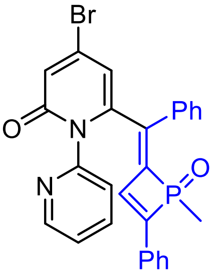

(*E*)-4-bromo-6-((1-methyl-1-oxido-4-phenyl-2*H*-phosphet-2-ylidene)(phenyl)methyl)-2*H*-[1,2'-bipyridin]-2-one (6k)

The title compound was isolated as a yellow solid (eluent: dichloromethane/methanol = 50/1, 93.8 mg, 91%). **^{1}H NMR (600 MHz, CDCl$_3$)** δ 8.35 (s, 1H), 7.75 (d, *J* = 65.2 Hz, 1H), 7.59 (s, 2H), 7.46 – 7.37 (m, 4H), 7.22 (s, 5H), 7.09 (s, 1H), 7.00 (s, 1H), 6.72 (s, 1H), 6.50 (s,

1H), 1.60 (s, 3H); **^{13}C NMR (150 MHz, CDCl$_3$)** δ 161.8, 159.4 (d, *J* = 76.5 Hz), 150.1, 149.0, 145.7, 140.1, 137.1, 136.2 (d, *J* = 7.5 Hz), 130.8, 130.7 (d, *J* = 6.0 Hz), 129.3, 129.0, 128.9, 127.5 (d, *J* = 9.0 Hz), 127.2, 123.9, 123.7, 123.3, 112.9, 16.1; **^{31}P NMR (243 MHz, CDCl$_3$)** δ 42.3; **HRMS (ESI) :** calcd. for $C_{27}H_{20}BrN_2NaO_2P^+$ $[M+Na]^+$: 537.0338; found : 537.0333.

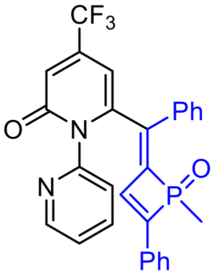

(*E*)-6-((1-methyl-1-oxido-4-phenyl-2*H*-phosphet-2-ylidene)(phenyl)methyl)-4-(trifluoromethyl)-2*H*-[1,2'-bipyridin]-2-one (6l)

The title compound was isolated as a yellow solid (eluent: dichloromethane/methanol = 50/1, 85.8 mg, 85%). **^{1}H NMR (600 MHz, CDCl$_3$)** δ 8.37 (d, *J* = 3.9 Hz, 1H), 7.70 (d, *J* = 63.9 Hz, 1H), 7.60 – 7.58 (m, 2H), 7.46 – 7.42 (m, 3H), 7.23 (s, 6H), 7.12 (s, 1H), 7.02 (s, 1H), 6.74 (s, 1H), 6.46 (s, 1H), 1.62 (s, 3H); **^{13}C NMR (150 MHz, CDCl$_3$)** δ 162.1, 159.8 (d, *J* = 78.0 Hz), 150.0, 149.1, 147.7, 141.2, 139.8, 137.2, 136.1 (d, *J* = 9.0 Hz), 130.9, 130.6 (d, *J* = 6.0 Hz), 129.3, 129.0, 128.9, 127.5 (d, *J* = 9.0 Hz), 123.9, 123.8, 122,1 (q, *J* = 271.5 Hz), 119.1, 103.7, 16,1; **^{31}P NMR (243 MHz, CDCl$_3$)** δ 42.3; **HRMS (ESI) :** calcd. for $C_{28}H_{20}F_3N_2NaO_2P^+$ $[M+Na]^+$: 527.1107; found : 527.1103.

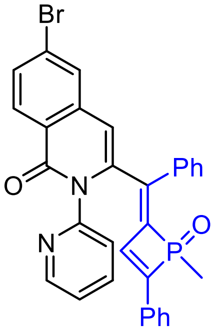

(*E*)-6-bromo-3-((1-methyl-1-oxido-4-phenyl-2*H*-phosphet-2-ylidene)(phenyl)methyl)-2-(pyridin-2-yl)isoquinolin-1(2*H*)-one (6m)

The title compound was isolated as a yellow solid (eluent: dichloromethane/methanol = 50/1, 96.1 mg, 85%). **^{1}H NMR (600 MHz, CDCl$_3$)** δ 8.36 (dd, *J* = 4.9, 1.9 Hz, 1H), 8.30 (d, *J* = 8.6 Hz, 1H), 7.97 – 7.71 (m, 2H), 7.64 (dd, *J* = 8.5, 1.9 Hz, 1H), 7.57 (d, *J* = 7.5 Hz, 2H), 7.48 – 7.37 (m, 4H), 7.29 –7.16 (m, 5H), 7.11 – 6.73 (m, 2H), 6.60 (s, 1H), 1.64 (d, *J* = 12.6 Hz, 3H); **^{13}C NMR (150 MHz, CDCl$_3$)** δ 162.5, 158.4 (d, *J* = 76.5 Hz), 148.9, 140.8, 137.8, 136.9, 136.7 (d, *J* = 7.5 Hz), 130.9 (d, *J* = 7.5 Hz), 130.8, 130.5, 130.2, 129.2, 128.8, 128.7, 128.4, 128.3, 128.0, 127.4, 127.3, 127.3, 127.3, 124.7, 124.4, 123.3, 108.3, 16.0; **^{31}P NMR (243 MHz, CDCl$_3$)** δ 42.2; **HRMS (ESI) :** calcd. for

$C_{31}H_{22}BrN_2NaO_2P^+$ [M+Na]$^+$: 587.0494; found : 587.0490.

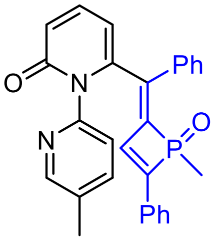

(*E*)-5'-methyl-6-((1-methyl-1-oxido-4-phenyl-2*H*-phosphet-2-ylidene)(phenyl)methyl)-2*H*-[1,2'-bipyridin]-2-one (6n)

The title compound was isolated as a yellow solid (eluent: dichloromethane/methanol = 50/1, 81.1 mg, 90%). **^{1}H NMR (600 MHz, $CDCl_3$)** δ 8.16 (s, 1H), 7.71 (d, *J* = 64.7 Hz, 1H), 7.58 – 7.56 (m, 2H), 7.51 (dd, *J* = 9.4, 6.7 Hz, 1H), 7.44 – 7.39 (m, 3H), 7.22 (s, 7H), 6.86 – 6.58 (m, 2H), 6.32 (d, *J* = 6.5 Hz, 1H), 2.20 (s, 3H), 1.59 (d, *J* = 11.4 Hz, 3H); **^{13}C NMR (150 MHz, $CDCl_3$)** δ 163.31, 158.4 (d, *J* = 78.0 Hz), 149.0, 145.7, 140.7, 139.5, 137.6, 136.7 (d, *J* = 7.5 Hz), 133.4, 130.9 (d, *J* = 6.0 Hz), 130.5, 129.2, 128.7, 128.6, 127.4, 127.3, 127.3, 123.2, 121.6, 108.9, 17.9, 16.1 (d, *J* = 67.5 Hz); **^{31}P NMR (243 MHz, $CDCl_3$)** δ 42.3; **HRMS (ESI) :** calcd. for $C_{28}H_{23}N_2NaO_2P^+$ [M+Na]$^+$: 473.1389; found : 473.1381.

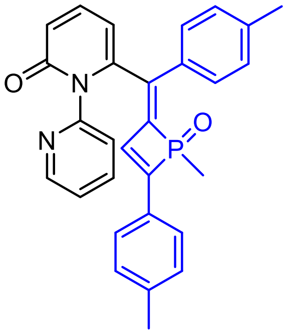

(*E*)-6-((1-methyl-1-oxido-4-(*p*-tolyl)-2*H*-phosphet-2-ylidene)(*p*-tolyl)methyl)-2*H*-[1,2'-bipyridin]-2-one (6o)

The title compound was isolated as a yellow solid (eluent: dichloromethane/methanol = 50/1, 80.8 mg, 87%). **^{1}H NMR (600 MHz, $CDCl_3$)** δ 8.32 (d, *J* = 3.8 Hz, 1H), 7.77 – 7.50 (m, 1H), 7.48 (dd, *J* = 9.4, 6.7 Hz, 1H), 7.43 (d, *J* = 7.7 Hz, 2H), 7.39 – 7.29 (m, 1H), 7.20 (d, *J* = 7.8 Hz, 2H), 7.03 (d, *J* = 38.2 Hz, 5H), 6.96 – 6.72 (m, 1H), 6.70 (dd, *J* = 9.4, 1.2 Hz, 1H), 6.28 (d, *J* = 6.6 Hz, 1H), 2.36 (s, 3H), 2.27 (s, 3H), 1.53 (s, 3H); **^{13}C NMR (150 MHz, $CDCl_3$)** δ 163.2, 157.9 (d, *J* = 78.0 Hz), 151.0, 148.8, 145.7, 141.0, 139.7, 138.9, 137.1, 133.9 (d, *J* = 9.0 Hz), 129.9, 129.4, 128.2 (d, *J* = 6.0 Hz), 127.3 (d, *J* = 9.0 Hz), 127.1, 123.9, 123.5, 121.4, 108.9, 21.6, 21.3, 16.2; **^{31}P NMR (243 MHz, $CDCl_3$)** δ 42.2; **HRMS (ESI) :** calcd. for $C_{29}H_{25}N_2NaO_2P^+$ [M+Na]$^+$: 487.1546; found : 487.1550.

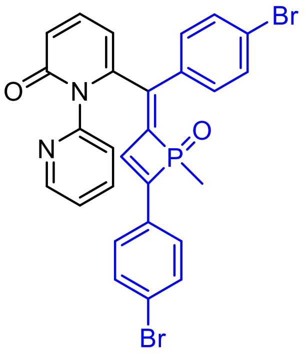

(*E*)-6-((4-bromophenyl)(4-(4-bromophenyl)-1-methyl-1-oxido-2*H*-phosphet-2-ylidene)methyl)-2*H*-[1,2'-bipyridin]-2-one (6p)

The title compound was isolated as a yellow solid (eluent: dichloromethane/methanol = 50/1, 107.0 mg, 90%). **^{1}H NMR (600 MHz, $CDCl_3$)** δ 8.36 (d, *J* = 4.0 Hz, 1H), 7.73 (d, *J* = 65.5 Hz, 1H), 7.57 (d, *J* = 8.2 Hz, 2H), 7.51 (dd, *J* = 9.4, 6.7 Hz, 1H), 7.48 – 7.29 (m, 5H), 7.12 (s, 3H), 6.83 (s, 1H), 6.74 (dd, *J* = 9.4, 1.2 Hz, 1H), 6.30 (dd, *J* = 6.7, 1.2 Hz, 1H), 1.59 (s, 3H); **^{13}C NMR (150 MHz, $CDCl_3$)** δ 163.0, 157.6 (d, *J* = 76.5 Hz), 150.9, 148.9, 144.7, 141.0, 139.4, 137.2, 135.5 (d, *J* = 9.0 Hz), 132.6, 132.0, 129.5 (d, *J* = 6.0 Hz), 128.8, 128.7 (d, *J* = 7.5 Hz), 125.1, 124.0, 123.7, 123.1, 122.0, 109.1, 16.3; **^{31}P NMR (243 MHz, $CDCl_3$)** δ 41.7; **HRMS (ESI) :** calcd. for $C_{27}H_{19}Br_2N_2NaO_2P^+$ $[M+Na]^+$: 614.9443; found : 614.9447.

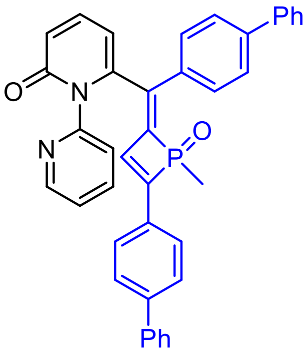

(*E*)-6-([1,1'-biphenyl]-4-yl(4-([1,1'-biphenyl]-4-yl)-1-methyl-1-oxido-2*H*-phosphet-2-ylidene)methyl)-2*H*-[1,2'-bipyridin]-2-one (6q)

The title compound was isolated as a yellow solid (eluent: dichloromethane/methanol = 50/1, 106.0 mg, 90%). **^{1}H NMR (600 MHz, $CDCl_3$)** δ 8.37 (d, *J* = 3.9 Hz, 1H), 7.92 – 7.69 (m, 1H), 7.66 (q, *J* = 8.4 Hz, 4H), 7.63 – 7.61 (m, 2H), 7.57 (d, *J* = 7.6 Hz, 2H), 7.53 (dd, *J* = 9.4, 6.7 Hz, 1H)., 7.51 – 7.41 (m, 7H), 7.40 – 7.31 (m, 4H), 7.16 – 6.81 (m, 2H), 6.76 (dd, *J* = 9.4, 1.2 Hz, 1H), 6.36 (dd, *J* = 6.7, 1.2 Hz, 1H), 1.65 (s, 3H); **^{13}C NMR (150 MHz, $CDCl_3$)** δ 163.2, 157.9 (d, *J* = 76.5 Hz), 149.0, 143.3, 141.4, 140.5, 139.9 (d, *J* = 7.5 Hz), 137.2, 135.7 (d, *J* = 7.5 Hz), 129.8 (d, *J* = 6.0 Hz), 129.0, 128.9, 128.1, 127.9, 127.9, 127.9, 127.8, 127.3, 127.1, 126.9, 124.0, 123.6, 121.7, 108.8, 16.3; **^{31}P NMR (243 MHz, $CDCl_3$)** δ 42.1; **HRMS (ESI) :** calcd. for $C_{39}H_{29}N_2NaO_2P^+$ $[M+Na]^+$: 611.1859; found : 611.1865.

(*E*)-6-((1-methyl-1-oxido-4-(*m*-tolyl)-2H-phosphet-2-ylidene)(*m*-tolyl)methyl)-2*H*-

[1,2'-bipyridin]-2-one (6r)

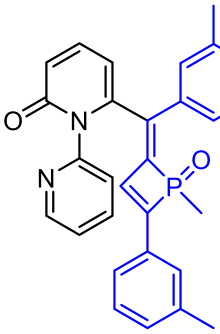

The title compound was isolated as a yellow solid (eluent: dichloromethane/methanol = 50/1, 78.0 mg, 84%). **^{1}H NMR (600 MHz, CDCl$_3$)** δ 8.35 (d, J = 4.2 Hz, 1H), 7.66 (d, J = 67.4 Hz, 1H), 7.50 (dd, J = 9.4, 6.7 Hz, 1H), 7.45 – 7.32 (m, 3H), 7.29 (t, J = 7.5 Hz, 1H), 7.19 (d, J = 7.6 Hz, 1H), 7.07 (s, 2H), 7.03 – 6.73 (m, 4H), 6.71 (d, J = 9.3 Hz, 1H), 6.31 (d, J = 6.7 Hz, 1H), 2.36 (s, 3H), 2.24 (s, 3H), 1.57 (d, J = 12.7 Hz, 3H); **^{13}C NMR (150 MHz, CDCl$_3$)** δ 163.2, 158.6 (d, J = 76.5 Hz), 151.0, 148.9, 145.7, 140,4, 139.6, 139.0, 138.3, 137.0, 136.7 (d, J = 7.5 Hz), 131.4, 130.8 (d, J = 6.0 Hz), 129.5, 129.1, 128.6, 127.9, 127.9, 127.8, 124.5 (d, J = 9.0 Hz), 124.4, 124.0, 123.3, 121.5, 109.0, 21.3, 16.3; **^{31}P NMR (243 MHz, CDCl$_3$)** δ 42.1; **HRMS (ESI) :** calcd. for $C_{29}H_{25}N_2NaO_2P^+$ [M+Na]$^+$: 487.1546; found : 487.1551.

(*E*)-6-((2-methoxyphenyl)(4-(2-methoxyphenyl)-1-methyl-1-oxido-2*H*-phosphet-2-ylidene)methyl)-2*H*-[1,2'-bipyridin]-2-one (6s)

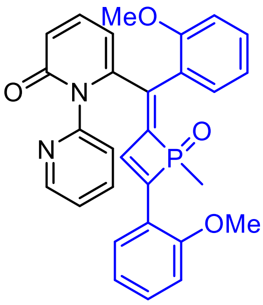

The title compound was isolated as a yellow solid (eluent: dichloromethane/methanol = 50/1, 87.4 mg, 88%). **^{1}H NMR (600 MHz, CDCl$_3$)** δ 8.33 (d, J = 4.8 Hz, 1H), 7.89 (d, J = 68.5 Hz, 1H), 7.54 (d, J = 7.7 Hz, 1H), 7.47 (dd, J = 9.3, 6.9 Hz, 1H), 7.36 – 7.29 (m, 2H), 7.12 – 7.08 (m, 1H), 7.03 (dd, J = 7.4, 4.9 Hz, 1H), 6.98 (t, J = 7.6 Hz, 2H), 6.93 (d, J = 8.4 Hz, 1H), 6.81 (d, J = 8.1 Hz, 1H), 6.66 – 6.60 (m, 3H), 6.37 (d, J = 6.8 Hz, 1H), 3.91 (s, 3H), 3.60 (s, 3H), 1.56 (d, J = 13.0 Hz, 3H); **^{13}C NMR (150 MHz, CDCl$_3$)** δ 163.3, 157.6 (d, J = 4.5 Hz), 156.7, 154.9, 154.3, 151.2, 150.0, 149.5, 148.7, 147.8 (d, J = 18.0 Hz), 141.7 (d, J = 12.0 Hz), 139.7, 136.7, 131.8, 130.7, 129.9, 129.1, 128.2 (d, J = 9.0 Hz), 126.0 (d, J = 9.0 Hz), 124.1, 123.2, 121.1, 121.0, 120.5 (d, J = 4.5 Hz), 120.1, 111.5, 110.7, 109.2, 55.7, 55.6, 16.4 (d, J = 58.5 Hz); **^{31}P NMR (243 MHz, CDCl$_3$)** δ 43.2; **HRMS (ESI) :** calcd. for $C_{29}H_{25}N_2NaO_4P^+$ [M+Na]$^+$: 519.1444; found : 519.1450.

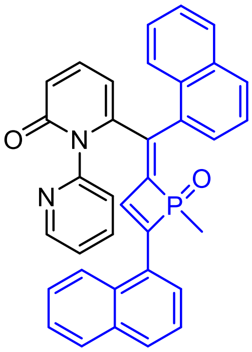

(*E*)-6-((1-methyl-4-(naphthalen-1-yl)-1-oxido-2*H*-phosphet-2-ylidene)(naphthalen-1-yl)methyl)-2*H*-[1,2'-bipyridin]-2-one (6t)

The title compound was isolated as a yellow solid (eluent: dichloromethane/methanol = 50/1, 81.6 mg, 76%). **^{1}H NMR (600 MHz, CDCl$_3$)** δ 8.54 (d, *J* = 8.7 Hz, 1H), 8.43 (s, 1H), 8.26 (d, *J* = 68.0 Hz, 1H), 7.90 (s, 2H), 7.81 (s, 2H), 7.78 (d, *J* = 7.4 Hz, 1H), 7.68 – 7.62 (m, 2H), 7.61 – 7.54 (m, 2H), 7.51 (t, *J* = 7.5 Hz, 1H), 7.43 (t, *J* = 7.6 Hz, 1H), 7.35 (t, *J* = 7.7 Hz, 1H), 7.09 (t, *J* = 7.7 Hz, 1H), 7.05 – 6.91 (m, 3H), 6.79 (s, 1H), 6.69 (d, *J* = 9.3 Hz, 1H), 6.03 (s, 1H), 1.57 (s, 3H); **^{13}C NMR (150 MHz, CDCl$_3$)** δ 163.2, 159.6 (d, *J* = 73.5 Hz) 151.0, 149.7 (d, *J* = 76.5 Hz), 149.0, 147.5 (d, *J* = 19.5 Hz), 143.1 (d, *J* = 12.0 Hz), 140.2, 139.6, 136.5, 134.0, 133.5, 131.9, 131.2, 131.0 (d, *J* = 6.0 Hz), 130.6, 129.3, 129.1 (d, *J* = 4.5 Hz), 129.0, 128.6, 127.8, 127.5 (d, *J* = 10.5 Hz), 126.6 (d, *J* = 3.0 Hz), 126.0, 125.5, 125.4, 125.1, 124.4, 124.3, 123.3, 123.2, 121.6, 110.3, 16.5 (d, *J* = 58.5 Hz), **^{31}P NMR (243 MHz, CDCl$_3$)** δ 44.4; **HRMS (ESI) :** calcd. for $C_{35}H_{25}N_2NaO_2P^+$ $[M+Na]^+$: 559.1546; found : 559.1553.

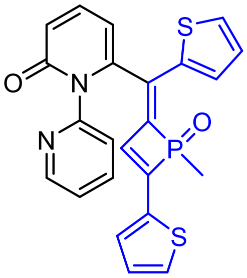

(*Z*)-6-((1-methyl-1-oxido-4-(thiophen-2-yl)-2*H*-phosphet-2-ylidene)(thiophen-2-yl)methyl)-2*H*-[1,2'-bipyridin]-2-one (6u)

The title compound was isolated as a yellow solid (eluent: dichloromethane/methanol = 50/1, 80.7 mg, 90%). **^{1}H NMR (600 MHz, CDCl$_3$)** δ 8.38 (s, 1H), 7.59 – 7.49 (m, 3H), 7.35 (d, *J* = 3.6 Hz, 1H), 7.24 (d, *J* = 5.1 Hz, 2H), 7.17 (t, *J* = 6.2 Hz, 1H), 7.10 (t, *J* = 4.4 Hz, 1H), 7.09 – 6,89 (m, 3H), 6.74 (d, *J* = 9.4 Hz, 1H), 6.34 (d, *J* = 48.0 Hz, 1H), 1.83 – 1.60 (m, 3H); **^{13}C NMR (150 MHz, CDCl$_3$)** δ 163.0, 151.3, 150.8 (d, *J* = 75.0 Hz), 148.7, 144.8, 143.9 (d, *J* = 73.5 Hz),140.5 (d, *J* = 9.0 Hz), 140.0, 139.4, 138.0, 137.4, 133.9 (d, *J* = 9.0 Hz), 129.9, 129.5 (d, *J* = 6.0 Hz), 128.5 128.0, 127.7, 127.4, 123.7, 121.8, 108.2, 16.6 (d, *J* = 60.0 Hz); **^{31}P NMR (243 MHz, CDCl$_3$)** δ 39.7; **HRMS (ESI) :** calcd. for $C_{23}H_{17}N_2NaO_2PS_2^+$ $[M+Na]^+$: 471.0361; found : 471.0365.

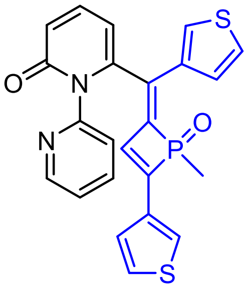

(*E*)-6-((1-methyl-1-oxido-4-(thiophen-3-yl)-2*H*-phosphet-2-ylidene)(thiophen-3-yl)methyl)-2*H*-[1,2'-bipyridin]-2-one (6v)

The title compound was isolated as a yellow solid (eluent: dichloromethane/methanol = 50/1, 71.6 mg, 80%). **^{1}H NMR (600 MHz, CDCl3)** δ 8.38 – 8.34 (m, 1H), 7.63 – 7.60 (m, 1H), 7.55 – 7.46 (m, 2H), 7.41 – 7.39 (m, 1H), 7.36 – 7.20 (m, 4H), 7.13 (s, 2H), 7.07 – 6.85 (m, 1H), 6.72 (dd, *J* = 9.4, 1.3 Hz, 1H), 6.29 (dd, *J* = 6.7, 1.3 Hz, 1H), 1.66 – 1.40 (m, 3H); **^{13}C NMR (150 MHz, CDCl3)** δ 163.1, 152.5, 151.9, 148.9, 145.5, 139.7, 139.0, 138.4, 137.2, 132.6 (d, *J* = 7.5 Hz), 127.4, 126.7, 126.6, 126.5, 125.5 (d, *J* = 9.0 Hz), 124.8, 123.6, 123.5, 121.6, 108.0, 16.0 (d, *J* = 55.5 Hz); **^{31}P NMR (243 MHz, CDCl3)** δ 41.7; **HRMS (ESI) :** calcd. for $C_{23}H_{17}N_2NaO_2PS_2^+$ [M+Na]$^+$: 471.0361; found : 471.0363.

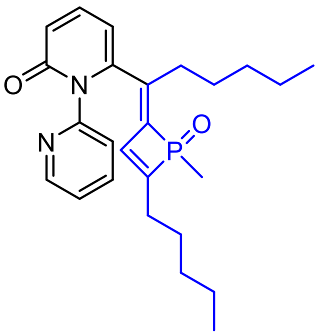

(*E*)-6-(1-(1-methyl-1-oxido-4-pentyl-2*H*-phosphet-2-ylidene)hexyl)-2*H*-[1,2'-bipyridin]-2-one (6w)

The title compound was isolated as a yellow oil (eluent: dichloromethane/methanol = 50/1, 71.6 mg, 80%). **^{1}H NMR (600 MHz, CDCl3)** δ 8.52 (s, 1H), 7.82 (td, *J* = 7.7, 1.9 Hz, 1H), 7.40 (t, *J* = 7.6 Hz, 1H), 7.34 (dd, *J* = 7.5, 4.7 Hz, 2H), 7.01 (d, *J* = 70.4 Hz, 1H), 6.61 (dd, *J* = 9.3, 1.2 Hz, 1H), 6.11 (s, 1H), 2.44 – 2.39 (m, 2H), 2.02 (s, 1H), 1.67 (s, 1H), 1.60 – 1.53 (m, 2H), 1.46 – 1.16 (m, 13H), 0.93 – 0.89 (m, 3H), 0.83 (t, *J* = 7.1 Hz, 3H); **^{13}C NMR (150 MHz, CDCl3)** δ 163.9 (d, *J* = 70.5 Hz), 163.0, 151.5, 149.0, 146.0, 145.5 (d, *J* = 73.5 Hz), 143.4, 139.9, 137.7, 130.4, 123.9, 123.7, 120.5, 107.2, 34.7 (d, *J* = 10.5 Hz), 31,5, 31.4, 28.6 (d, *J* = 6.0 Hz), 27.6, 27.1 (d, *J* = 6.0 Hz), 22.3, 15.8 (d, *J* = 55.5 Hz), 13.9, 13.9; **^{31}P NMR (243 MHz, CDCl3)** δ 43.3; **HRMS (ESI) :** calcd. for $C_{25}H_{33}N_2NaO_2P^+$ [M+Na]$^+$: 447.2172; found : 447.2172.

(*E*)-6-(1-(1-methyl-1-oxido-4-phenethyl-2*H*-phosphet-2-ylidene)-3-phenylpropyl)-

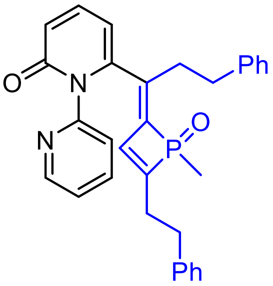

2*H*-[1,2'-bipyridin]-2-one (6x)

The title compound was isolated as a yellow oil (eluent: dichloromethane/methanol = 50/1, 74.9 mg, 76%). **^{1}H NMR (600 MHz, $CDCl_3$)** δ 8.59 – 8.41 (m, 1H), 7.75 (s, 1H), 7.36 (dd, *J* = 9.3, 6.7 Hz, 1H), 7.31 (t, *J* = 7.5 Hz, 3H), 7.25 – 7.19 (m, 6H), 7.15 (t, *J* = 7.4 Hz, 1H), 7.11 – 6.92 (m, 3H), 6.61 (d, *J* = 9.3 Hz, 1H), 5.99 (d, *J* = 6.8 Hz, 1H), 2.91 – 2.63 (m, 6H), 2.30 (s, 1H), 1.93 (s, 1H), 1.28 (d, *J* = 11.9 Hz, 3H); **^{13}C NMR (150 MHz, $CDCl_3$)** δ 163.0, 151.4, 149.1, 145.7, 144.2, 140.5, 140.1, 139.9, 137.8, 130.1, 128.6, 128.5, 128.5, 128.4, 128.3, 126.5, 126.2, 123.9 (d, *J* = 30.0 Hz), 120.8, 107.3, 36.6 (d, *J* = 10.5 Hz), 33.4 (d, *J* = 6.0 Hz), 29.9 (d, *J* = 6.0 Hz), 15.6 (d, *J* = 54.0 Hz); **^{31}P NMR (243 MHz, $CDCl_3$)** δ 43.6; **HRMS (ESI) :** calcd. for $C_{31}H_{29}N_2NaO_2P^+$ $[M+Na]^+$: 515.1859; found : 515.1857.

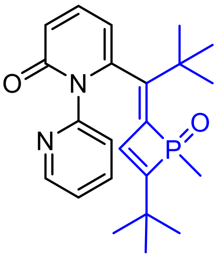

(*E*)-6-(1-(4-(*tert*-butyl)-1-methyl-1-oxido-2*H*-phosphet-2-ylidene)-2,2-dimethylpropyl)-2*H*-[1,2'-bipyridin]-2-one (6y)

The title compound was isolated as a yellow oil (eluent: dichloromethane/methanol = 50/1, 68.2 mg, 86%). **^{1}H NMR (600 MHz, $CDCl_3$)** δ 8.50 – 8.46 (m, 1H), 7.74 (td, *J* = 7.7, 1.9 Hz, 1H), 7.38 (dd, *J* = 9.3, 6.8 Hz, 1H), 7.31 – 7.28 (m, 1H), 7.26 (d, *J* = 8.2 Hz, 1H), 6.75 (d, *J* = 68.7 Hz, 1H), 6.61 (dd, *J* = 9.3, 1.2 Hz, 1H), 5.98 (dd, *J* = 6.8, 1.2 Hz, 1H), 1.74 (d, *J* = 12.3 Hz, 3H), 1.20 (s, 9H), 1.05 (s, 9H); **^{13}C NMR (150 MHz, $CDCl_3$)** δ 169.7 (d, *J* = 72.0 Hz), 163.5, 151.4, 149.2, 147.0 (d, *J* = 19.5 Hz), 145.1 (d, *J* = 69.0 Hz), 142.0 (d, *J* = 16.5 Hz), 140.7, 139.5, 137.5, 124.4, 123.8, 120.2, 106.5, 36.6 (d, *J* = 4.5 Hz), 34.3 (d, *J* = 6.0 Hz), 30.7, 29.0 (d, *J* = 6.0 Hz), 19.1 (d, *J* = 55.5 Hz); **^{31}P NMR (243 MHz, $CDCl_3$)** δ 40.4; **HRMS (ESI) :** calcd. for $C_{23}H_{29}N_2NaO_2P^+$ $[M+Na]^+$: 419.1859; found : 419.1865.

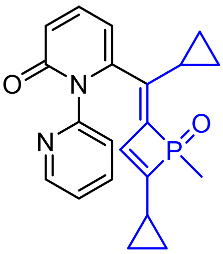

(*E*)-6-(cyclopropyl(4-cyclopropyl-1-methyl-1-oxido-2*H*-phosphet-2-ylidene)methyl)-2*H*-[1,2'-bipyridin]-2-one (6z)

The title compound was isolated as a yellow oil (eluent: dichloromethane/methanol = 50/1, 49.6 mg, 68%). **^{1}H NMR (600 MHz, CDCl$_3$)** δ 8.59 – 8.49 (m, 1H), 7.82 (td, *J* = 7.7, 1.9 Hz, 1H), 7.41 – 7.32 (m, 3H), 7.01 – 6.81 (m, 1H), 6.61 (d, *J* = 9.2 Hz, 1H), 6.07 – 5.90 (m, 1H), 1.81 – 1.59 (m, 2H), 1.36 (d, *J* = 12.6 Hz, 2H), 1.14 – 1.00 (m, 3H), 0.92 – 0.88 (m, 1H), 0.80 – 0.54 (m, 5H); **^{13}C NMR (150 MHz, CDCl$_3$)** δ 165.2 (d, *J* = 75.0 Hz), 163.1, 151.8, 148.9, 145,9, 140.7, 139.9, 137.6, 130.3, 124.0, 123.8, 120.5, 107.0, 16.2 (d, *J* = 54.0 Hz), 15.6 (d, *J* = 12.0 Hz), 10.8 (d, *J* = 6.0 Hz), 9.5 (d, *J* = 4.5 Hz), 9.3, 7.1 (d, *J* = 16.5 Hz); **^{31}P NMR (243 MHz, CDCl$_3$)** δ 42.7; **HRMS (ESI) :** calcd. for $C_{21}H_{21}N_2NaO_2P^+$ $[M+Na]^+$: 387.1233; found : 387.1236.

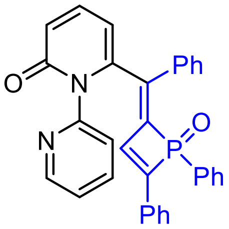

(*E*)-6-((1-oxido-1,4-diphenyl-2*H*-phosphet-2-ylidene)(phenyl)methyl)-2*H*-[1,2'-bipyridin]-2-one (6aa)

The title compound was isolated as a yellow solid (eluent: dichloromethane/methanol = 50/1, 84.7 mg, 85%). **^{1}H NMR (600 MHz, CDCl$_3$)** δ 8.30 (s, 1H), 8.06 (d, *J* = 69.2 Hz, 1H), 7.75 (s, 2H), 7.56 – 7.45 (m, 5H), 7.35 – 7.30 (m, 4H), 7.27 – 7.21 (m, 1H), 7.12 – 6.92 (m, 5H), 6.78 – 6.68 (m, 3H), 6.52 – 6.36 (m, 1H); **^{13}C NMR (150 MHz, CDCl$_3$)** δ 163.2, 151.0, 148.7,145.8, 143.9 (d, *J* = 12.0 Hz), 139.8, 136.9, 136.4 (d, *J* = 9.0 Hz), 132.7, 131.3, 130.6 (d, *J* = 6.0 Hz), 130.5, 129.2, 129.1, 129.0 (d, *J* = 12.0 Hz), 128.6, 128.5, 128.4, 127.6 (d, *J* = 9.0 Hz), 127.2, 124.3, 123.3, 121.5, 113.8, 109.1; **^{31}P NMR (243 MHz, CDCl$_3$)** δ 33.6; **HRMS (ESI) :** calcd. for $C_{32}H_{23}N_2NaO_2P^+$ $[M+Na]^+$: 521.1389; found : 521.1386.

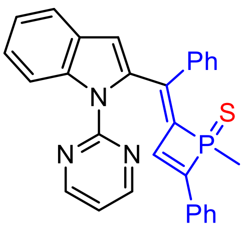

(*E*)-1-methyl-4-phenyl-2-(phenyl(1-(pyrimidin-2-yl)-1*H*-indol-2-yl)methylene)-2*H*-phosphete 1-sulfide (7)

The title compound was isolated as a yellow solid (eluent: petroleum ether/ethyl acetate = 10/1, 41.9 mg, 88%). **^{1}H NMR (600 MHz, CDCl$_3$)** δ 8.55 (d, *J* = 4.8 Hz, 2H), 8.34 (d, *J* = 8.3 Hz, 1H), 7.70 (d, *J* = 7.8 Hz, 1H), 7.66 (s, 0.5 H), 7.62 – 7.59 (m, 4H), 7.55 (s, 0.5 H), 7.41 – 7.34 (m, 4H), 7.31 (td, *J* = 7.5, 1.0 Hz, 1H), 7.24 – 7.21 (m, 2H), 7.19 – 7.16

(m, 1H), 6.90 (t, *J* = 4.8 Hz, 1H), 6.87 (s, 1H), 2.14 (d, *J* = 12.7 Hz, 3H); **^{13}C NMR (150 MHz, CDCl$_3$)** δ 157.94, 157.14, 152.34 (d, *J* = 67.5 Hz), 140.65 (d, *J* = 13.5 Hz), 140.32 (d, *J* = 66.0 Hz), 138.47 (d, *J* = 7.5 Hz), 137.21, 136.29 (d, *J* = 19.5 Hz), 133.01, 131.08 (d, *J* = 6.0 Hz), 129.82, 129.09, 128.80, 128.76, 128.34, 128.17, 126.64 (d, *J* = 9.0 Hz), 124.45, 122.47, 120.89, 117.15, 114.01, 111.67, 23.26 (d, *J* = 40.5 Hz); **^{31}P NMR (243 MHz, CDCl$_3$)** δ 57.5; **HRMS (ESI) :** calcd. for $C_{29}H_{22}N_3NaSP^+$ [M+Na]$^+$: 498.1164; found : 498.1171.

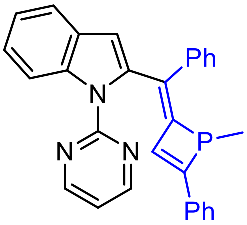

(*E*)-2-((1-methyl-4-phenylphosphet-2(1*H*)-ylidene)(phenyl)methyl)-1-(pyrimidin-2-yl)-1*H*-indole (8)

The title compound was isolated as a yellow solid (eluent: petroleum ether/ethyl acetate = 100/1, 43.0 mg, 97%). **^{1}H NMR (600 MHz, CDCl$_3$)** δ 8.54 (d, *J* = 4.8 Hz, 2H), 8.25 (d, *J* = 8.2 Hz, 1H), 7.69 (d, *J* = 7.8 Hz, 1H), 7.44 – 7,42 (m, 2H), 7.41 – 7.39 (m, 2H), 7.36 (t, *J* = 7.6 Hz, 2H), 7.34 – 7.32 (m, 1H), 7.29 (dd, *J* = 7.4, 5.6 Hz, 2H), 7.27 – 7.25 (m, 1H), 7.16 (t, *J* = 7.6 Hz, 2H), 7.10 (t, *J* = 7.3 Hz, 1H), 6.88 – 6.86 (m, 2H), 1.27 (d, *J* = 2.1 Hz, 3H); **^{13}C NMR (150 MHz, CDCl$_3$)** δ 157.83, 157.47, 156.45, 139.76, 138.09 (d, *J* = 4.5 Hz), 137.43 (d, *J* = 6.0 Hz), 137.12, 134.65 (d, *J* = 4.5 Hz), 133.93 (d, *J* = 10.5 Hz), 129.08, 128.67, 128.47, 128.17, 128.09 (d, *J* = 6.0 Hz), 127.91, 127.00, 125.64 (d, *J* = 6.0 Hz), 123.75, 122.13, 120.57, 116.93, 113.52, 111.01, 13.8 (d, *J* = 30.0 Hz); **^{31}P NMR (243 MHz, CDCl$_3$)** δ 9.2; **HRMS (ESI) :** calcd. for $C_{29}H_{22}N_3NaP^+$ [M+Na]$^+$: 466.1444; found : 466.1446.

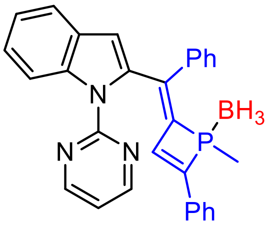

(*E*)-2-((1-methyl-4-phenylphosphet-2(1*H*)-ylidene)(phenyl)methyl)-1-(pyrimidin-2-yl)-1*H*-indole borane (9)

The title compound was isolated as a yellow liquid (eluent: petroleum ether/EtOAc = 100/1, 32.0 mg, 70%). **^{1}H NMR (600 MHz, CDCl$_3$)** δ 8.56 (d, *J* = 4.8 Hz, 2H), 8.33 (d, *J* = 8.3 Hz, 1H), 7.71 (d, *J* = 7.7 Hz, 1H), 7.50 (d, *J* = 7.1 Hz, 2H), 7.46 – 7.43 (m, 2H), 7.42 – 7.36 (m, 4H), 7.36

– 7.33 (m, 1H), 7.32 – 7.30 (m, 1H), 7.23 – 7.20 (m, 2H), 7.19 – 7.16 (m, 1H), 6.93 – 6.89 (m, 2H), 1.59 (d, *J* = 10.4 Hz, 3H), 1.40 – 1.03 (m, 3H); **^{13}C NMR (150 MHz, CDCl$_3$)** δ 157.92, 157.19, 147.97 (d, *J* = 49.5 Hz), 140.23 (d, *J* = 4.5 Hz), 138.47 (d, *J* = 6.0 Hz), 137.36, 136.18 (d, *J* = 15.0 Hz), 133.63 (d, *J* = 3.0 Hz), 132.78 (d, *J* = 49.5 Hz), 132.07 (d, *J* = 9.0 Hz), 129.59, 129.01, 128.80, 128.47, 128.39, 128.22, 126.54 (d, *J* = 7.5 Hz), 124.48, 122.46, 120.88, 117.16, 113.92, 111.84, 12.48 (d, *J* = 19.5 Hz); **^{31}P NMR (243 MHz, CDCl$_3$)** δ 57.3; **^{11}B NMR (193 MHz, CDCl$_3$)** δ -35.6; **HRMS (ESI) :** calcd. for $C_{29}H_{25}BN_3NaOP^+$ [M+Na]$^+$: 480.1771; found : 480.1772.

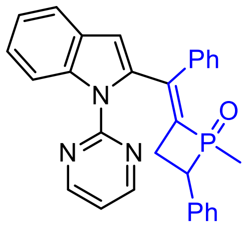

(*E*)-1-methyl-2-phenyl-4-(phenyl(1-(pyrimidin-2-yl)-1*H*-indol-2-yl)methylene)phosphetane 1-oxide (10)

The title compound was isolated as a white solid (eluent: dichloromethane/methanol = 100/1, 108.5 mg, 47%). **^{1}H NMR (600 MHz, CDCl$_3$)** δ 8.57 (d, *J* = 4.8 Hz, 2H), 8.33 (dd, *J* = 8.4, 0.9 Hz, 1H), 7.69 (dt, *J* = 7.7, 1.1 Hz, 1H), 7.60 – 7.55 (m, 2H), 7.38 – 7.33 (m, 3H), 7.30 – 7.27 (m, 1H), 7.27 – 7.22 (m, 3H), 7.20 – 7.16 (m, 2H), 7.15 – 7.12 (m, 1H), 6.95 (t, J = 4.8 Hz, 1H), 6.87 (s, 1H), 4.29 (dt, *J* = 23.5, 10.3 Hz, 1H), 3.34 – 3.23 (m, 1H), 2.94 – 2.88 (m, 1H), 1.24 (d, *J* = 12.1 Hz, 3H); **^{13}C NMR (150 MHz, CDCl$_3$)** δ 157.94, 157.25, 144.17, 138.37 (d, *J* = 70.5 Hz), 138.37 (d, *J* = 9.0 Hz), 136.91, 136.40 (d, *J* = 19.5 Hz), 136.23 (d, *J* = 7.5 Hz), 128.87 (d, *J* = 1.5 Hz), 128.77, 128.50, 128.41, 128.05, 126.93 (d, *J* = 7.5 Hz), 126.81 (d, *J* = 3.0 Hz), 124.43, 122.42, 120.84, 117.05, 114.06, 110.65, 49.79 (d, *J* = 58.5 Hz), 27.62 (d, *J* = 4.5 Hz), 12.72 (d, *J* = 52.5 Hz); **^{31}P NMR (243 MHz, CDCl$_3$)** δ 47.6; **HRMS (ESI) :** calcd. for $C_{29}H_{24}N_3NaOP^+$ [M+Na]$^+$: 484.1549; found : 484.1542.

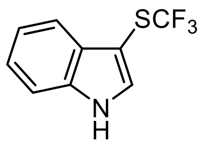

3-((trifluoromethyl)thio)-1*H*-indole (12)[4]

The title compound was isolated as a white solid (eluent: petroleum ether/EtOAc = 15/1, 36.1 mg, 83%). **^{1}H NMR (600 MHz, CDCl$_3$)** **^{1}H NMR (600 MHz, CDCl$_3$)** δ 8.47 (s, 1H), 7.86 – 7.80 (m, 1H), 7.53 (d, *J* = 2.8 Hz,

1H), 7.45 – 7.40 (m, 1H), 7.34 – 7.28 (m, 2H); **^{13}C NMR (150 MHz, $CDCl_3$)** δ 136.03, 132.78, 129.47, 129.45 (q, *J* = 307.5 Hz), 123.46, 121.66, 119.36, 111.68, 95.62.

8 NMR spectra

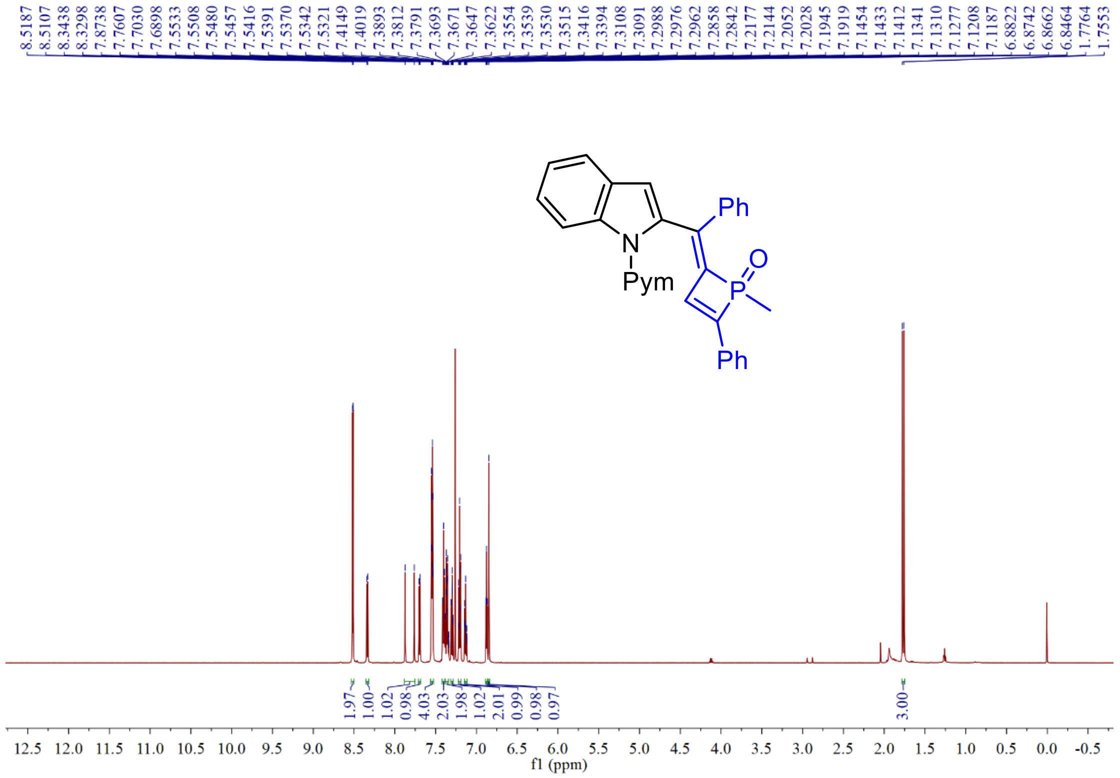

^{1}H NMR spectrum of compound **3a**

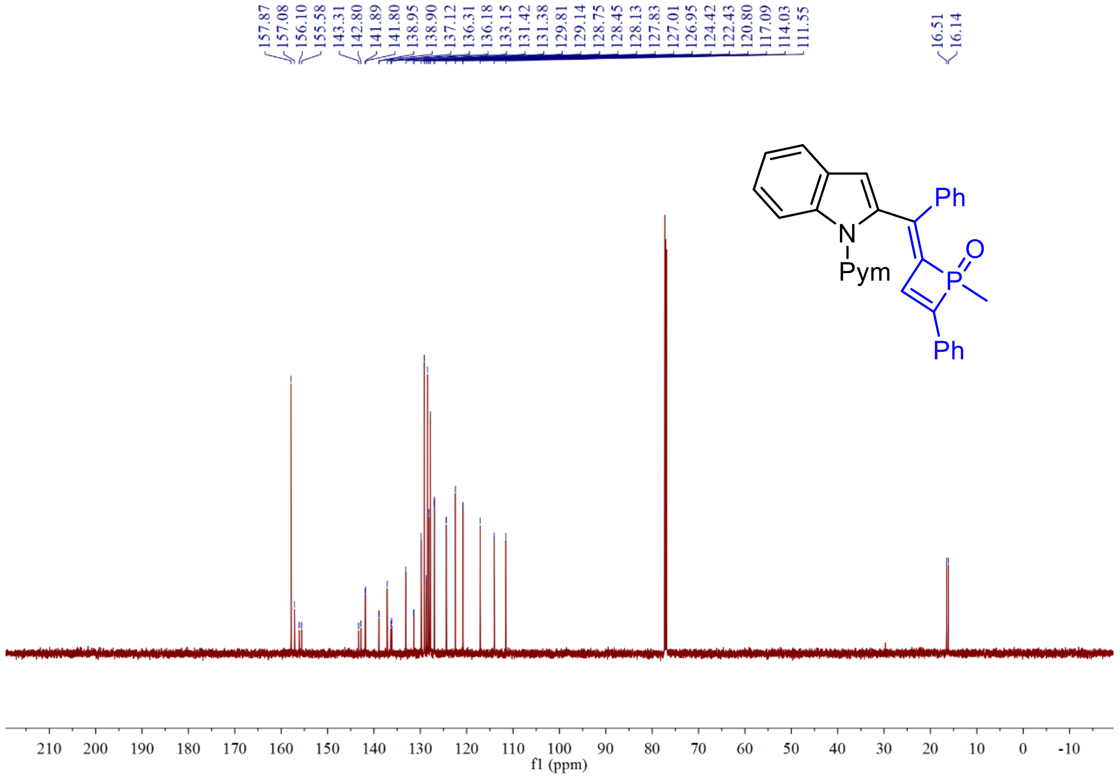

^{13}C NMR spectrum of compound **3a**

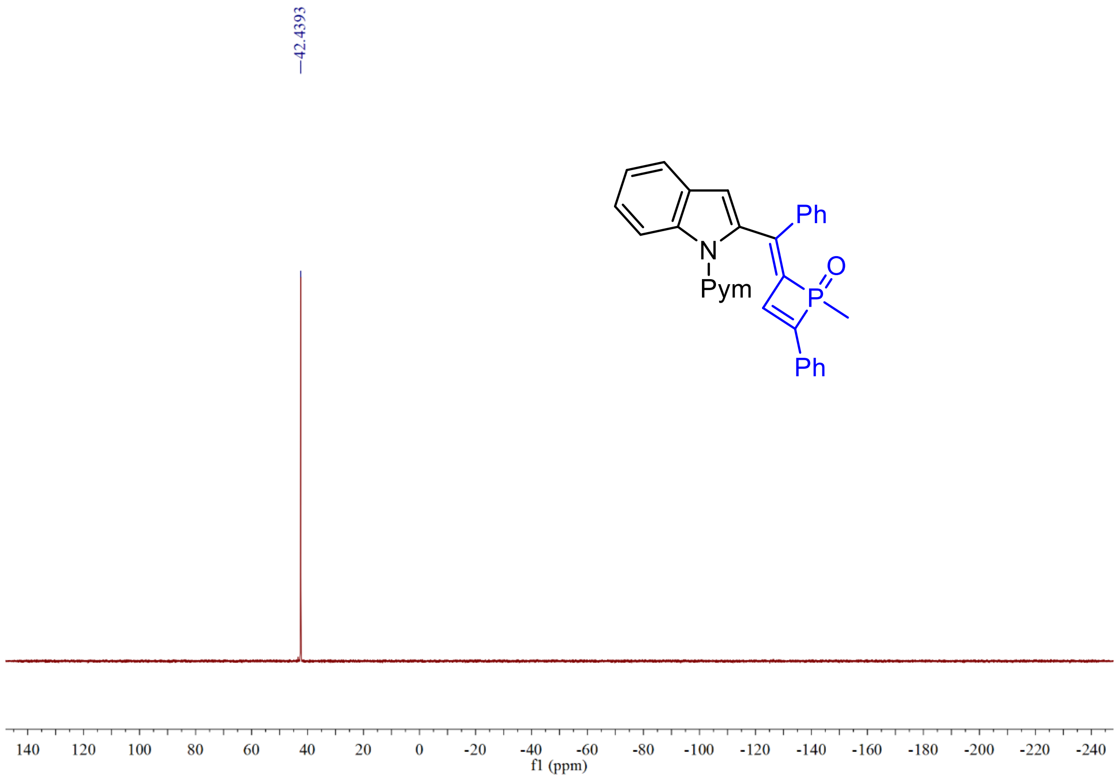

^{31}P NMR spectrum of compound **3a**

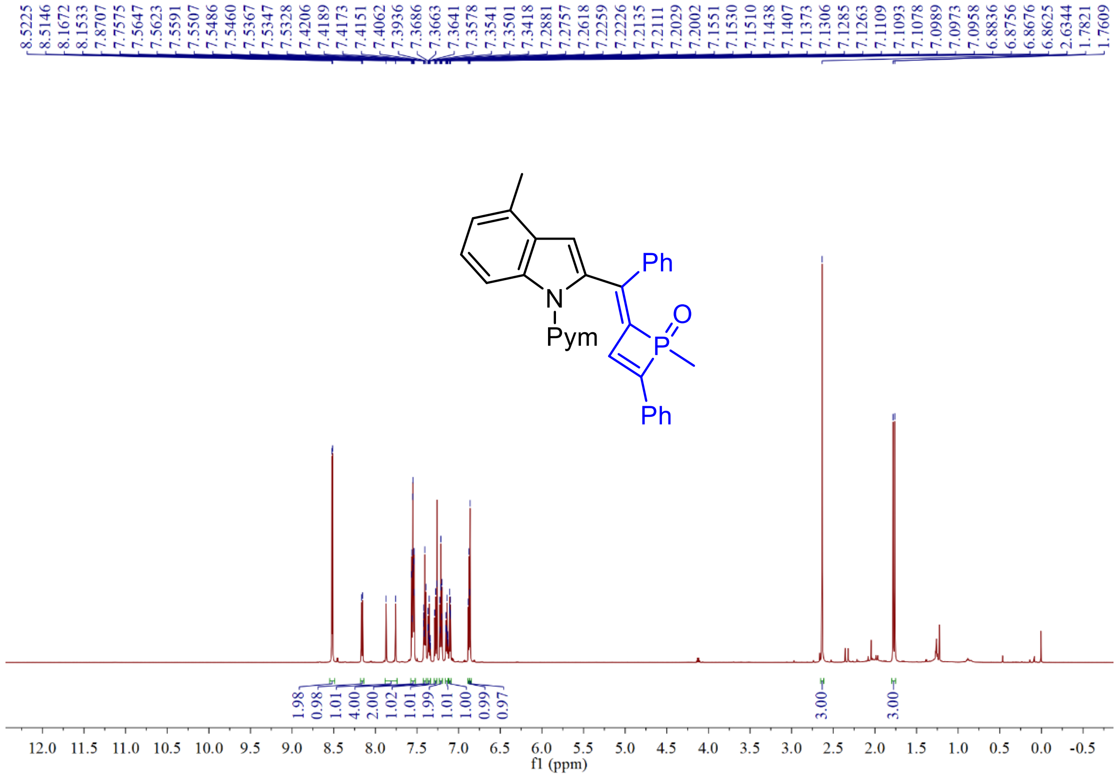

^{1}H NMR spectrum of compound **3b**

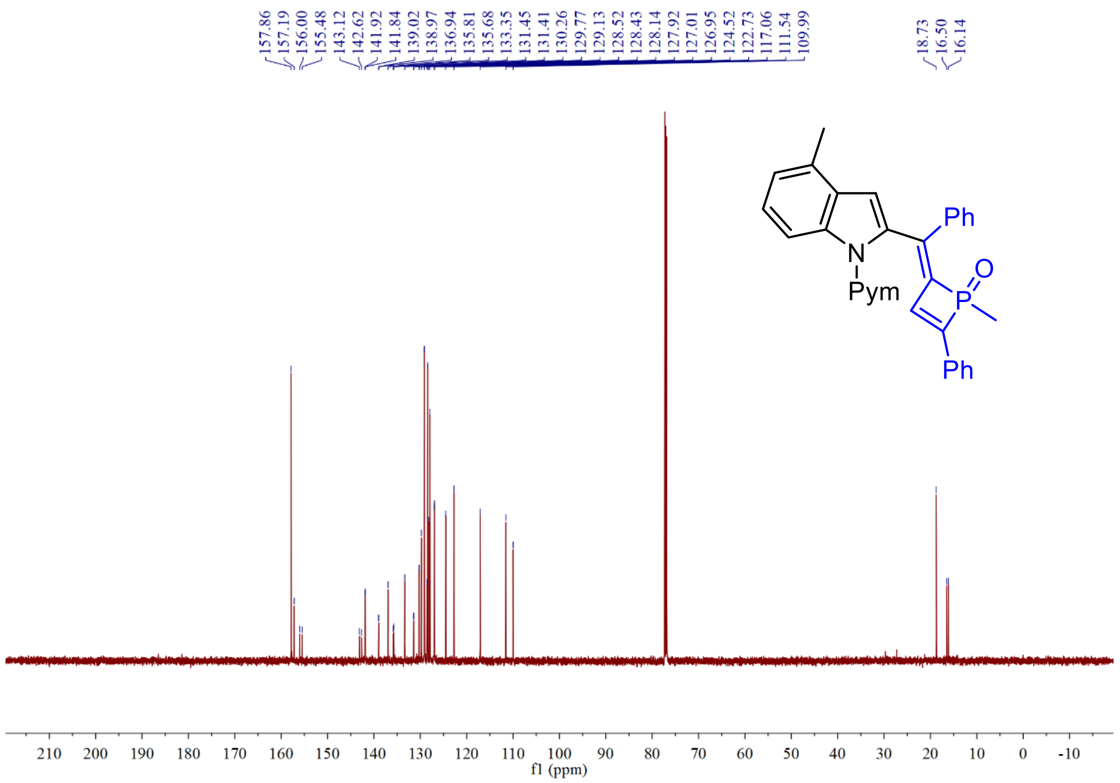

^{13}C NMR spectrum of compound **3b**

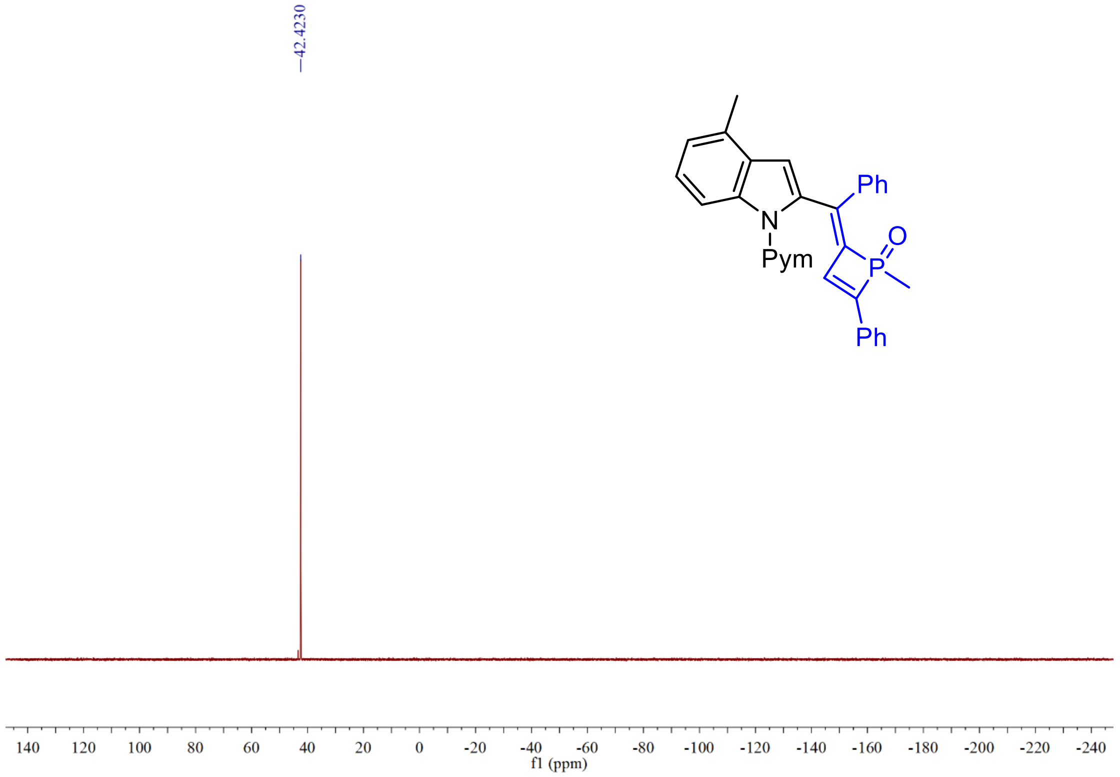

^{31}P NMR spectrum of compound **3b**

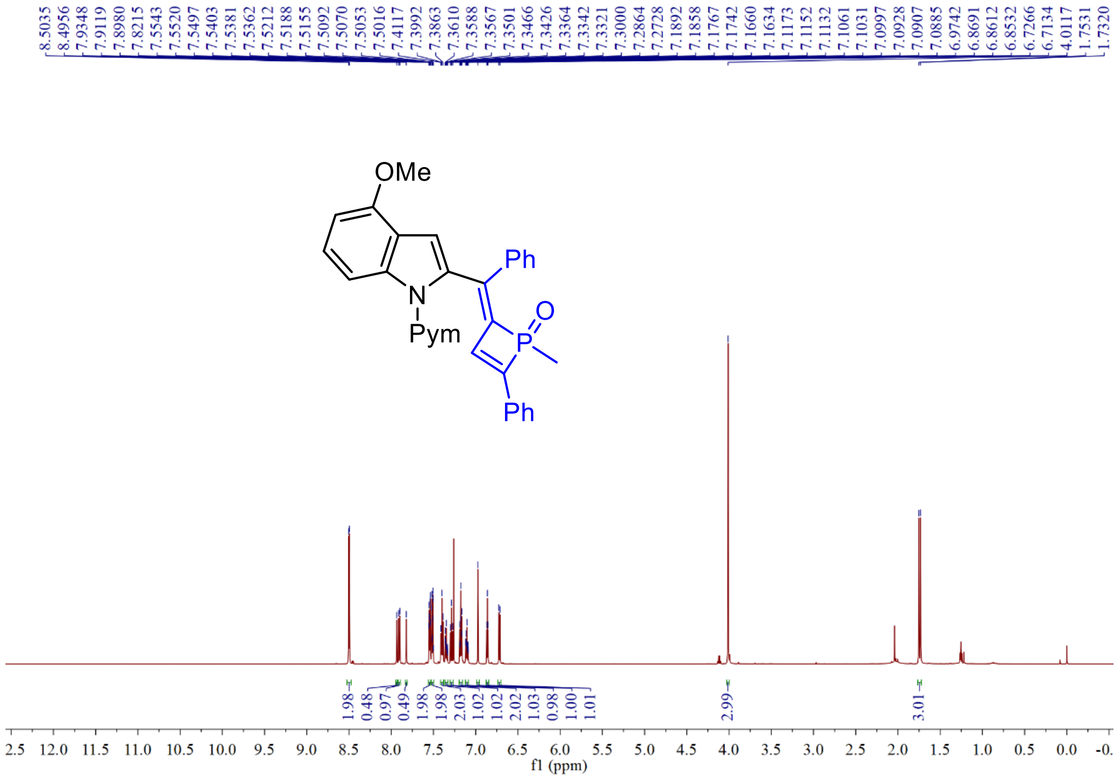

^{1}H NMR spectrum of compound **3c**

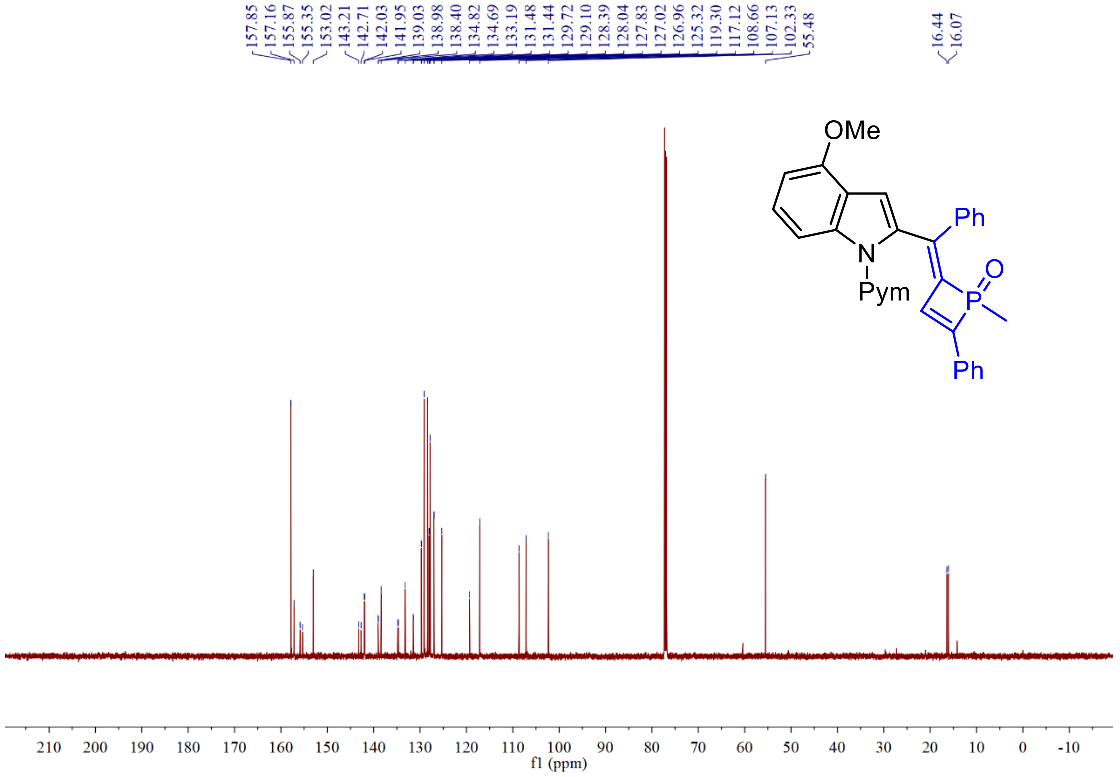

^{13}C NMR spectrum of compound **3c**

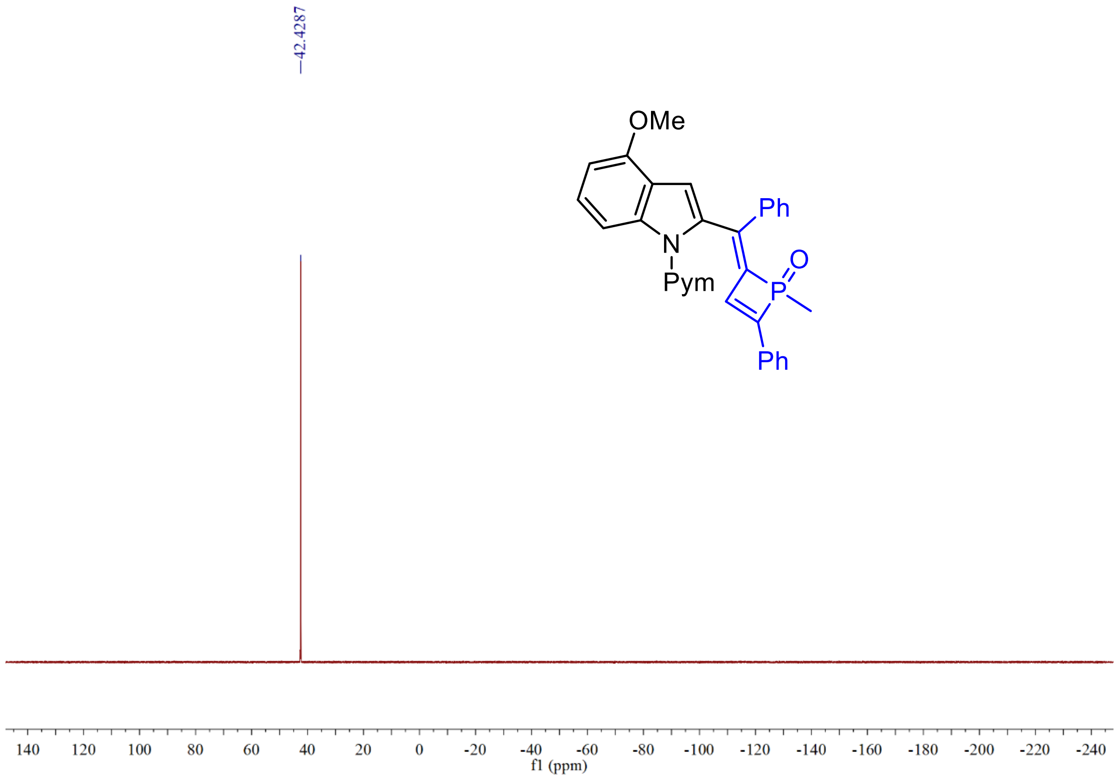

^{31}P NMR spectrum of compound **3c**

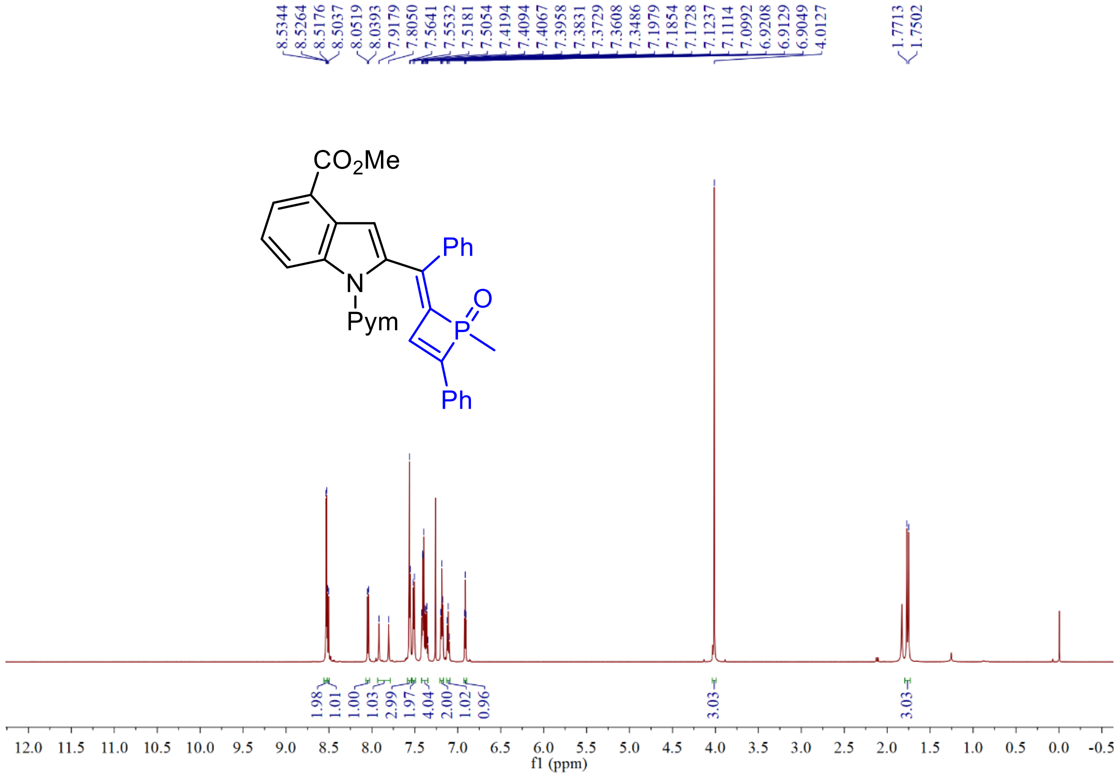

^{1}H NMR spectrum of compound **3d**

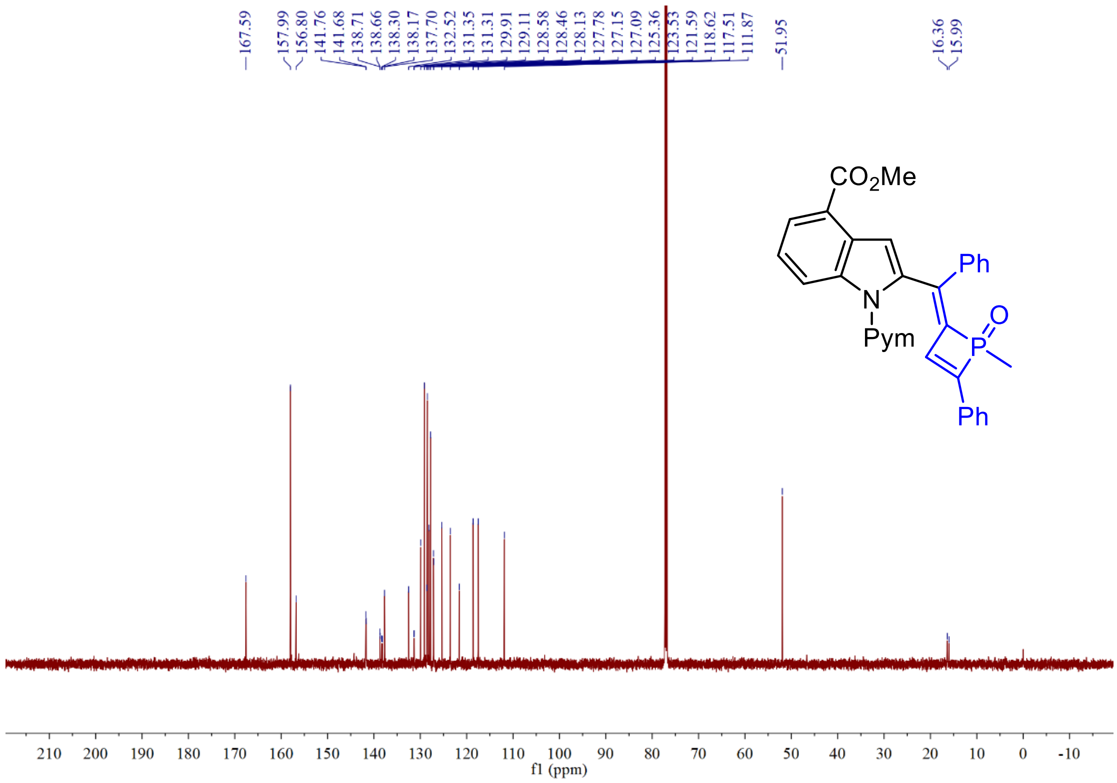

^{13}C NMR spectrum of compound **3d**

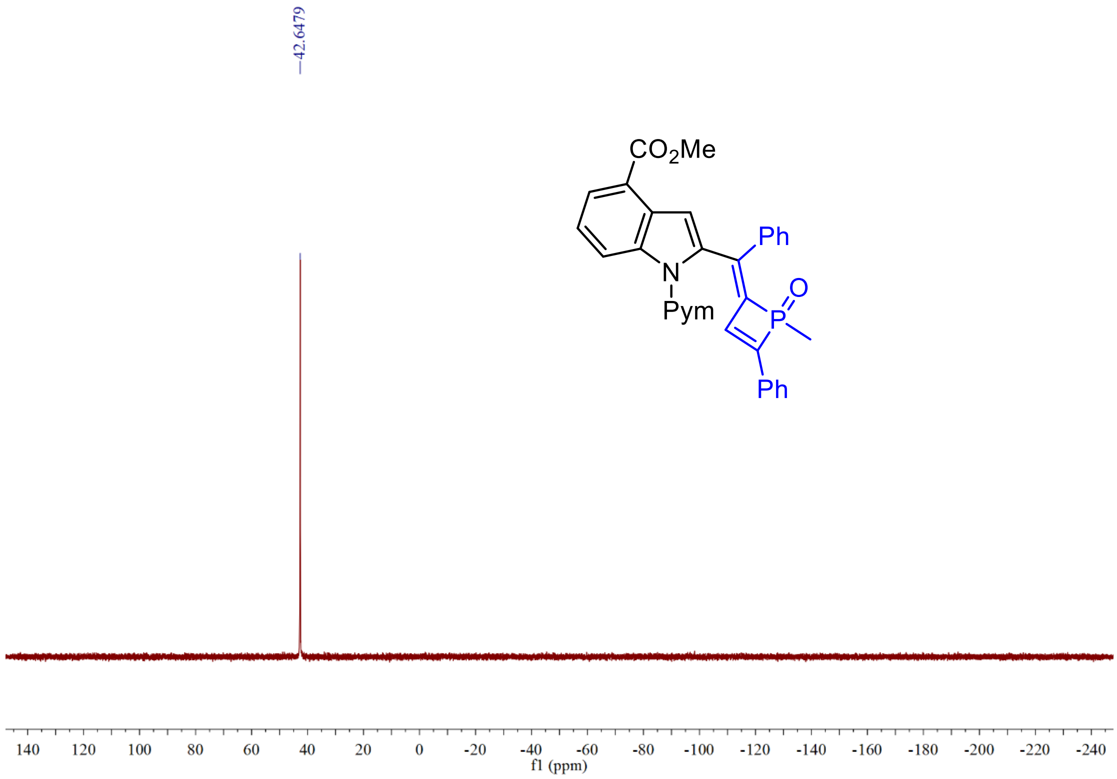

^{31}P NMR spectrum of compound **3d**

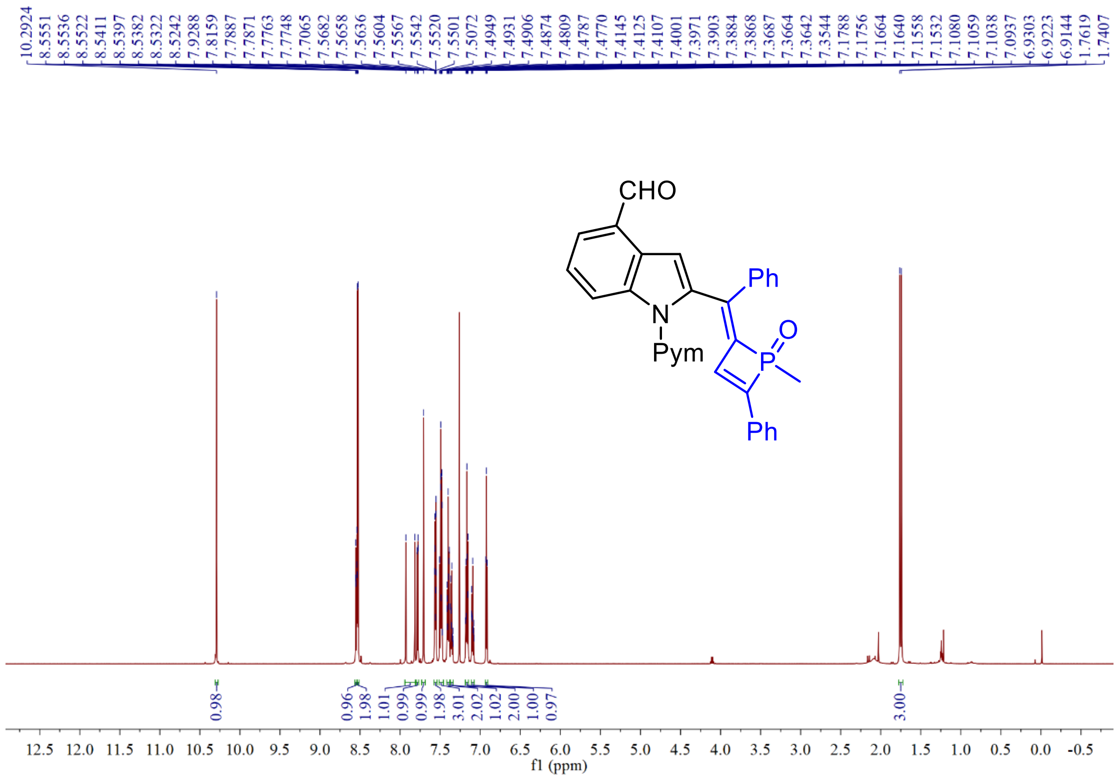

^{1}H NMR spectrum of compound **3e**

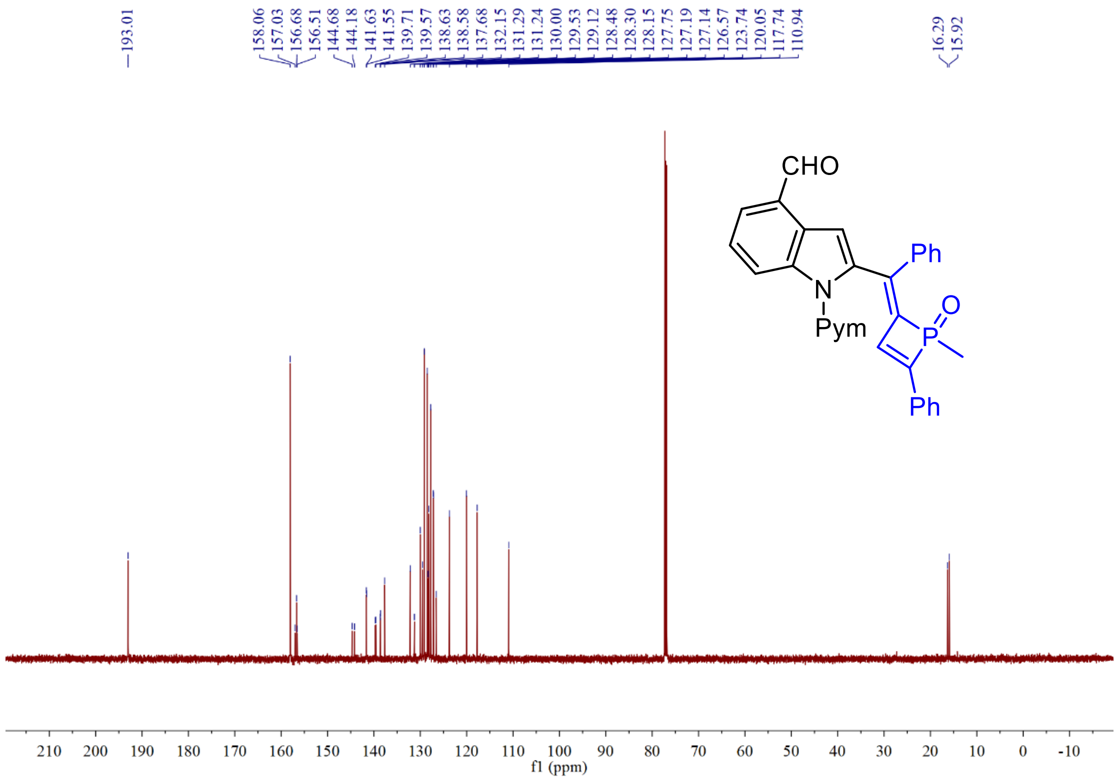

^{13}C NMR spectrum of compound **3e**

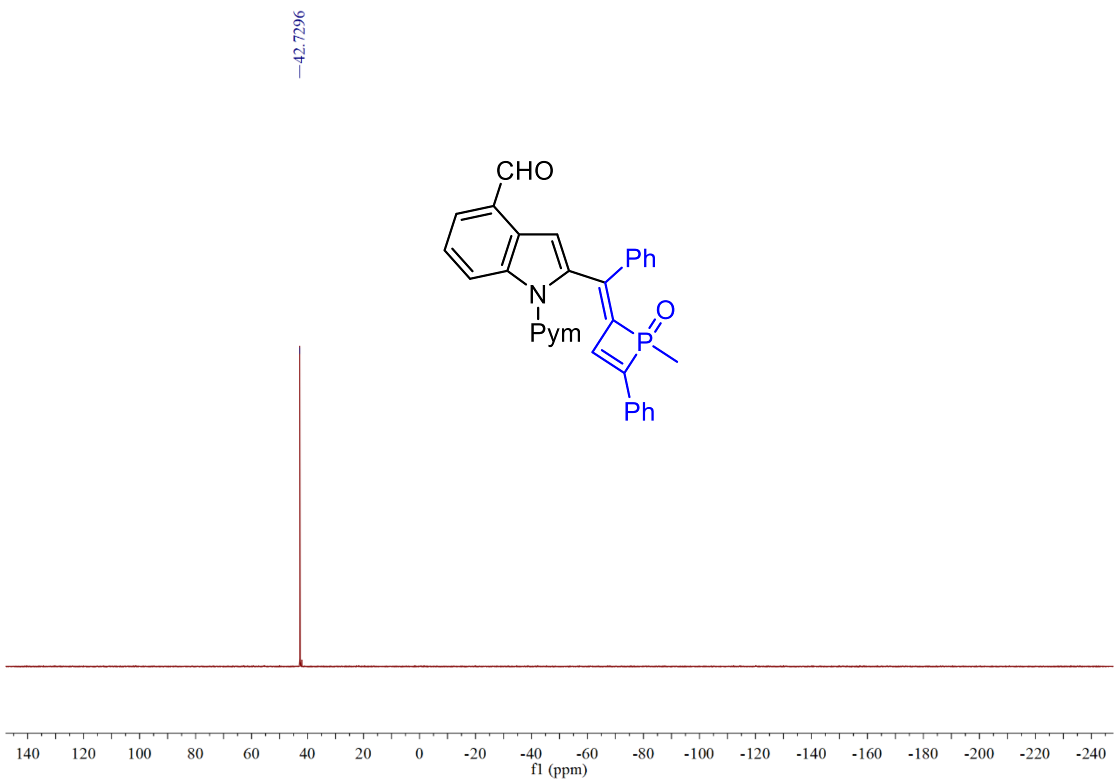

^{31}P NMR spectrum of compound **3e**

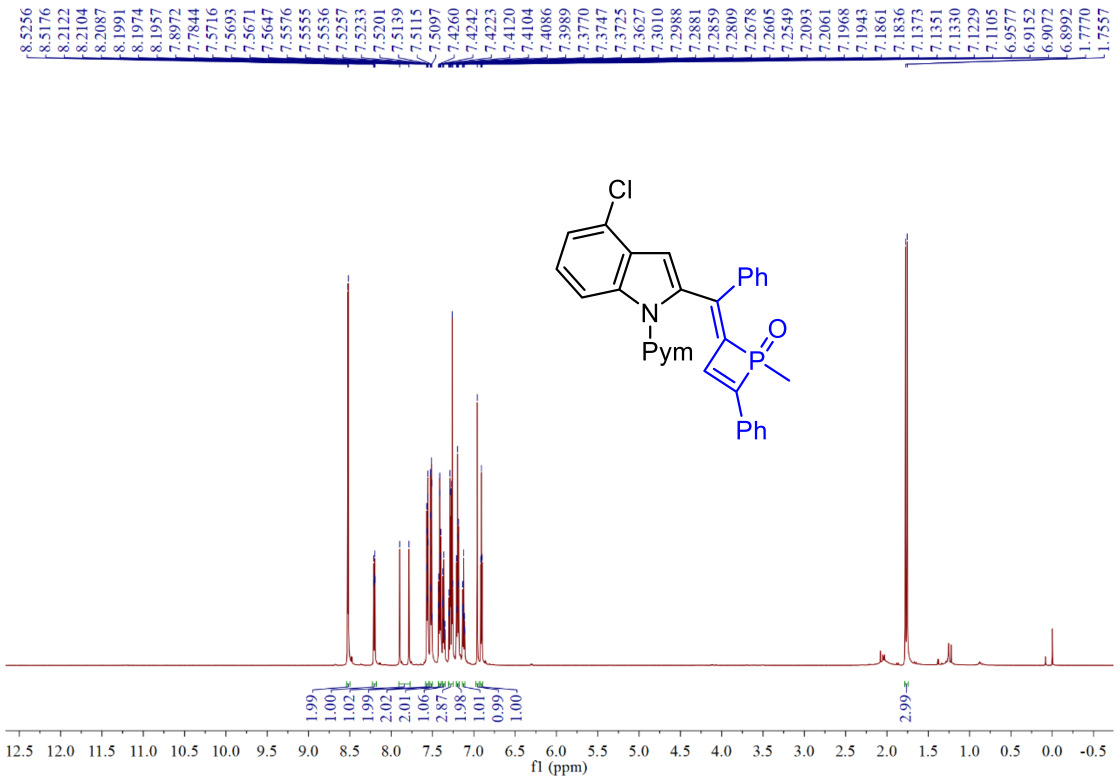

^{1}H NMR spectrum of compound **3f**

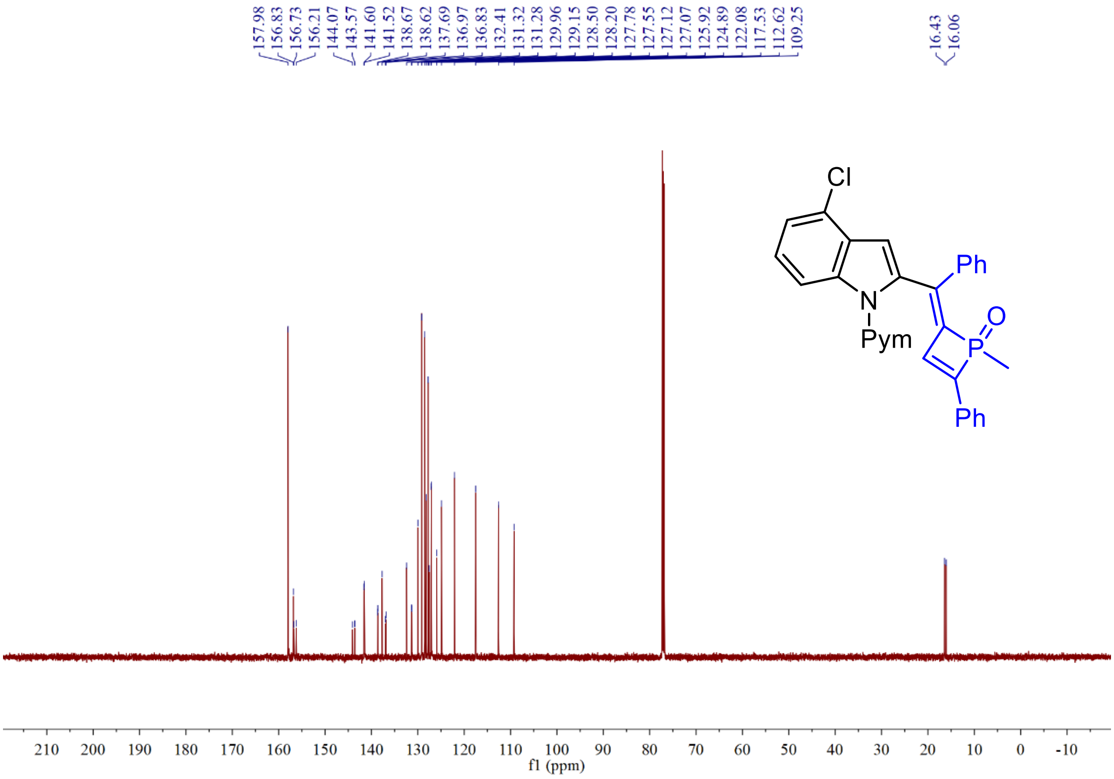

^{13}C NMR spectrum of compound **3f**

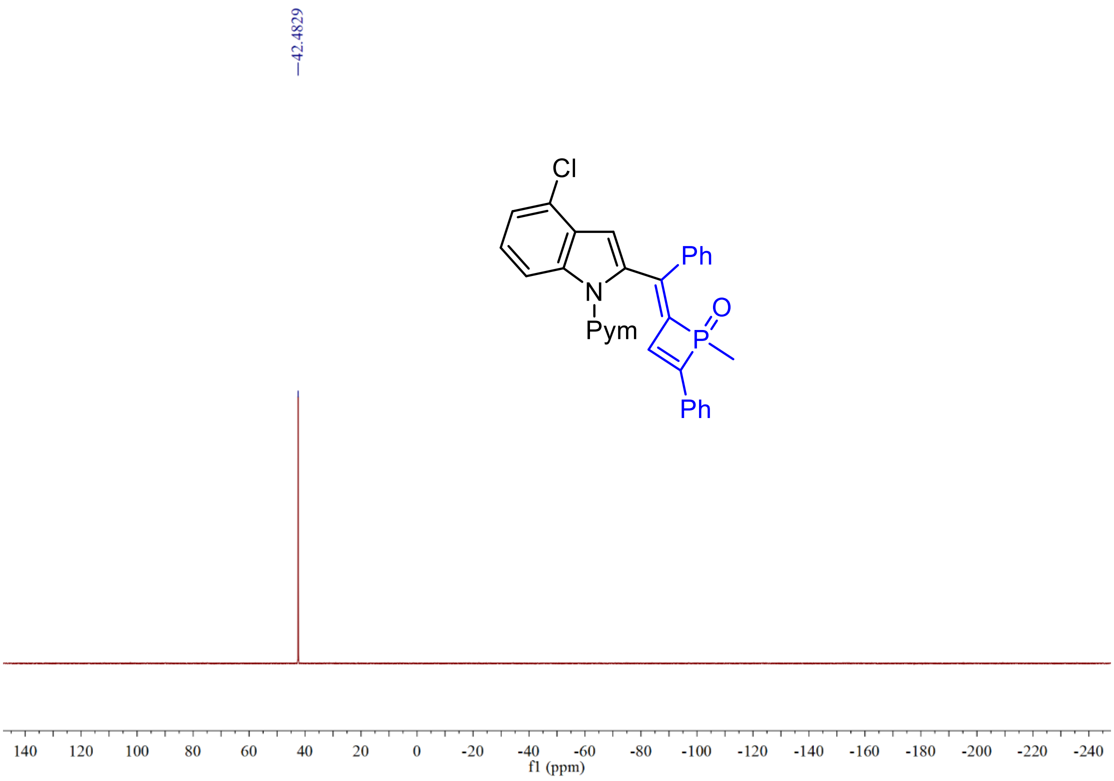

^{31}P NMR spectrum of compound **3f**

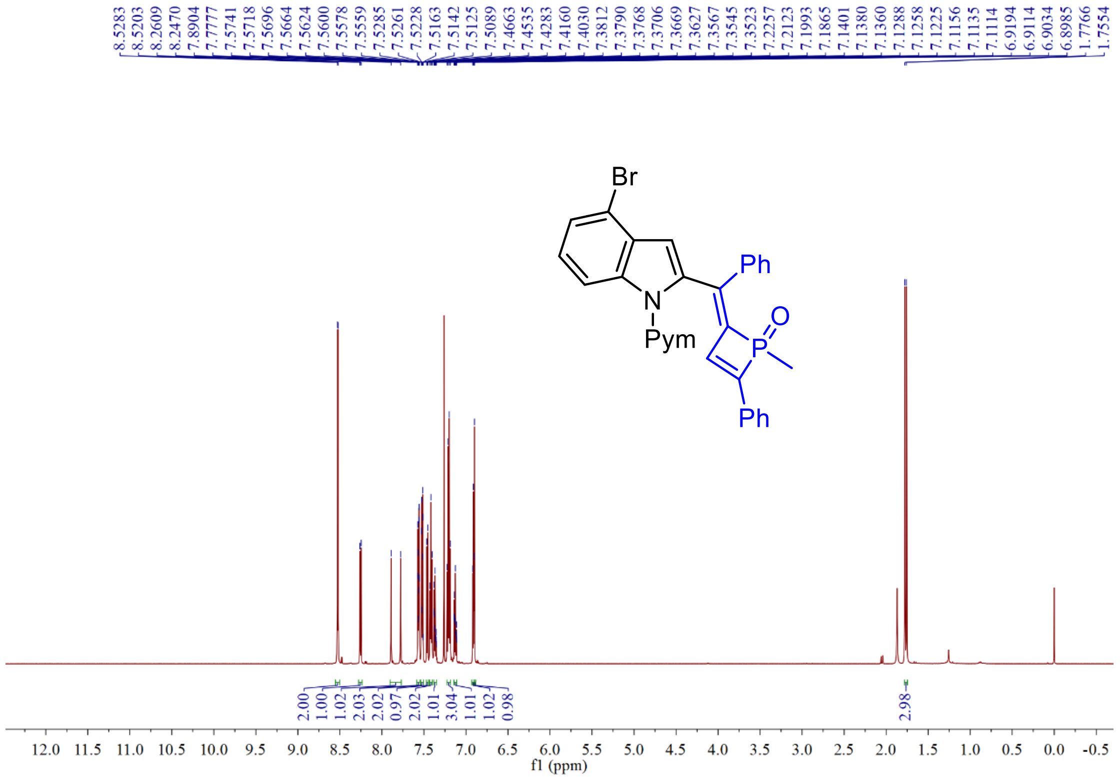

^{1}H NMR spectrum of compound **3g**

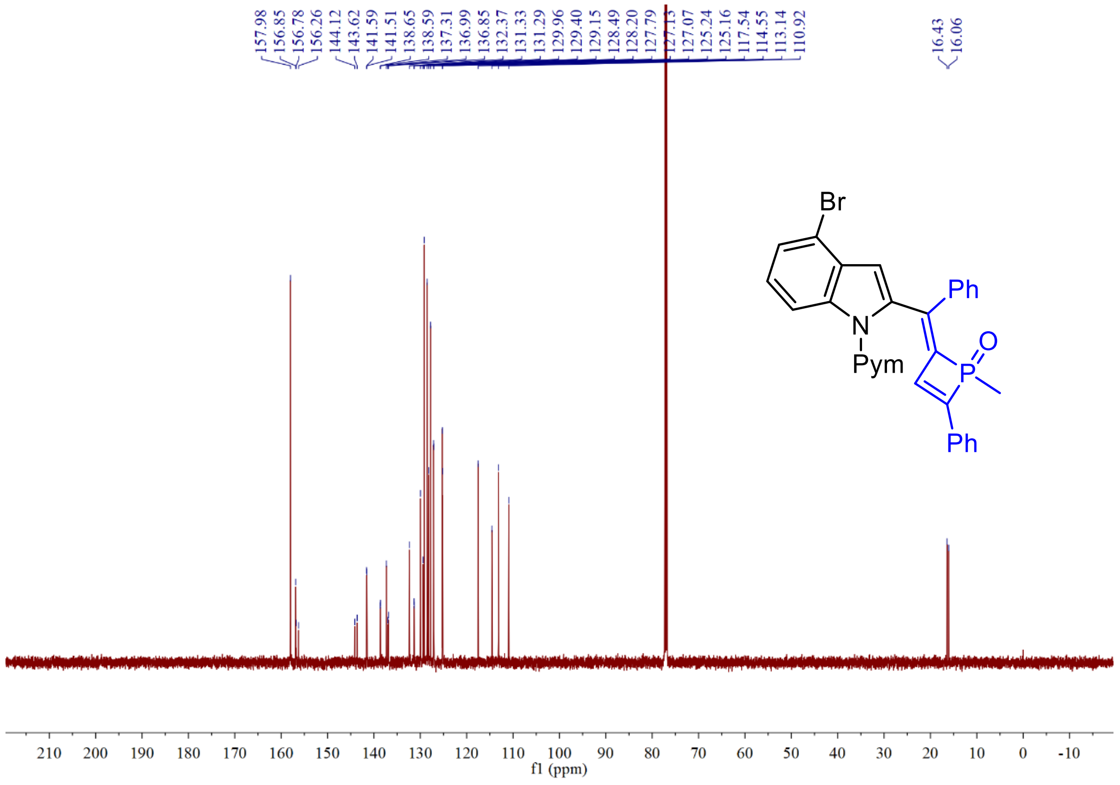

^{13}C NMR spectrum of compound **3g**

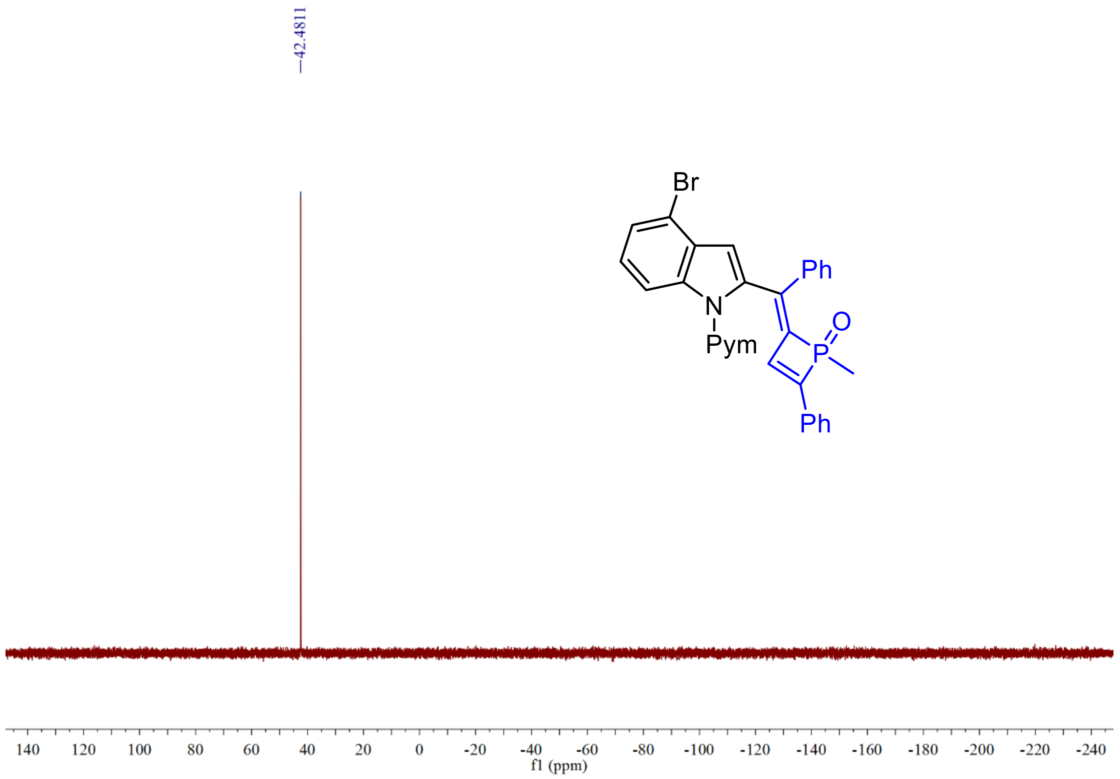

^{13}C NMR spectrum of compound **3g**

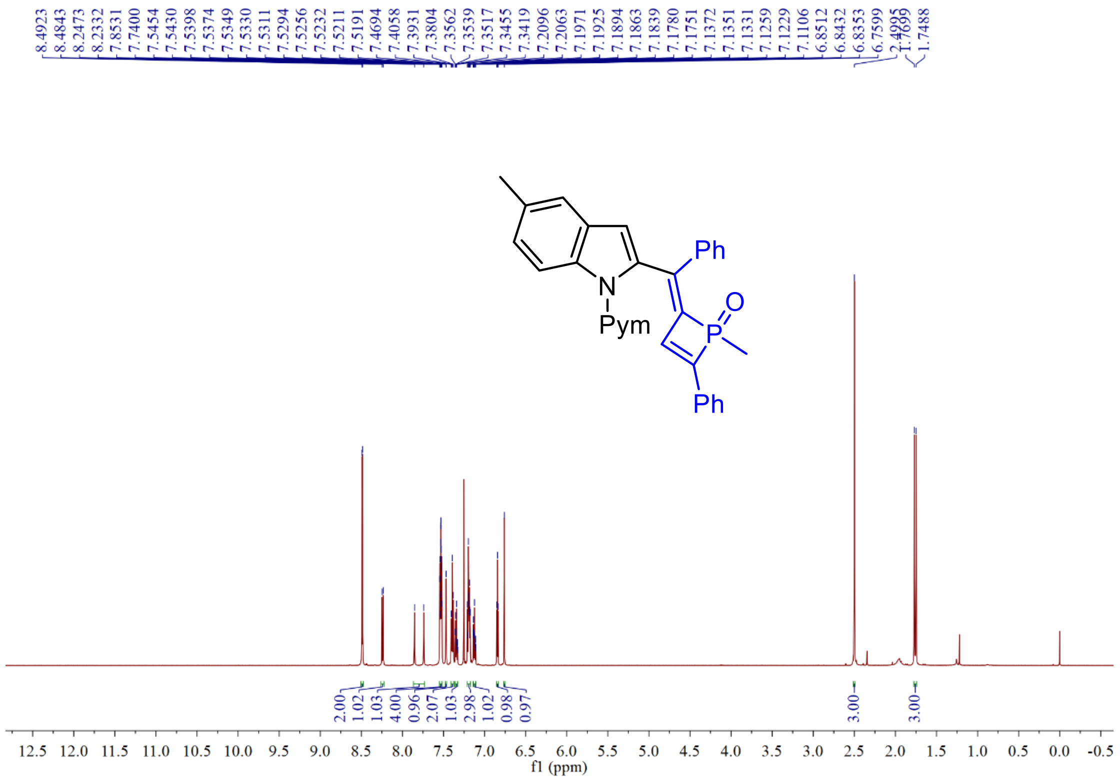

^{1}H NMR spectrum of compound **3h**

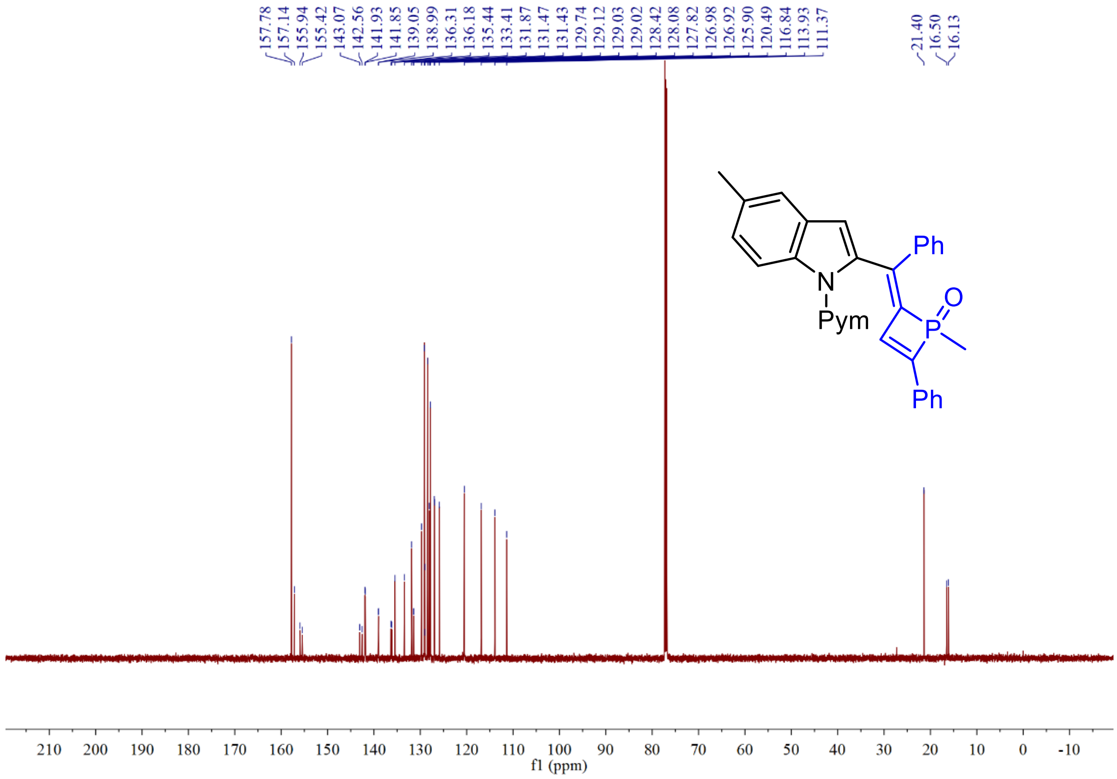

^{13}C NMR spectrum of compound **3h**

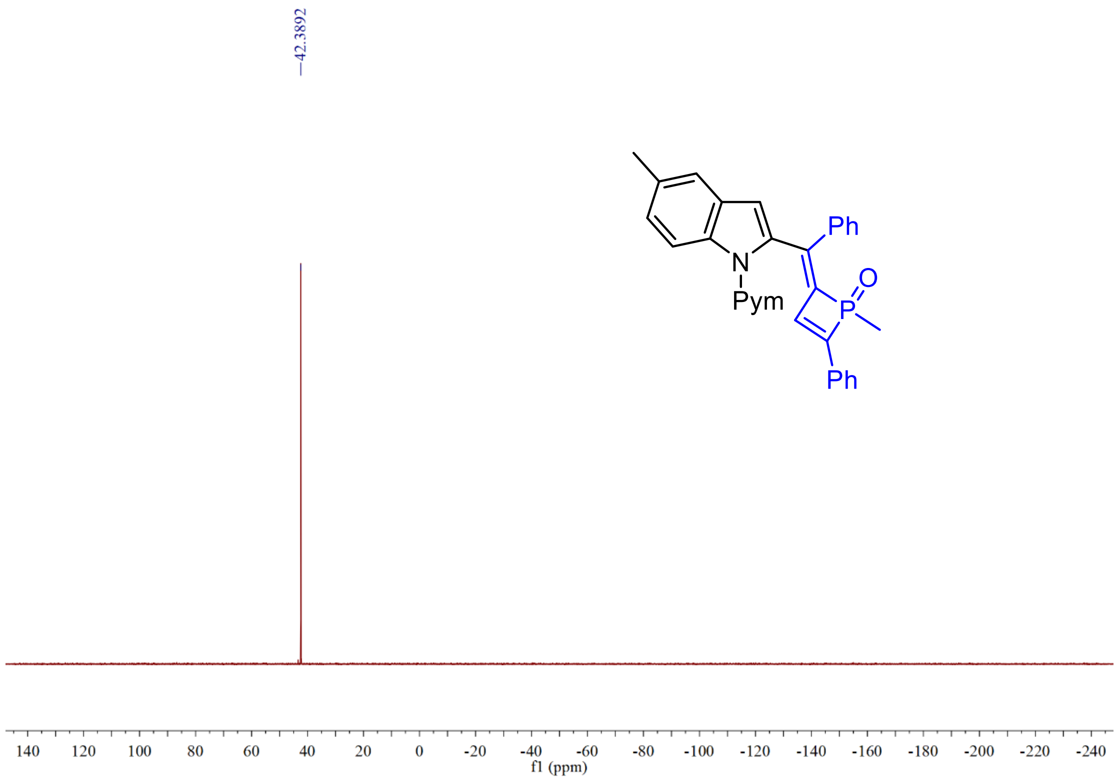

^{31}P NMR spectrum of compound **3h**

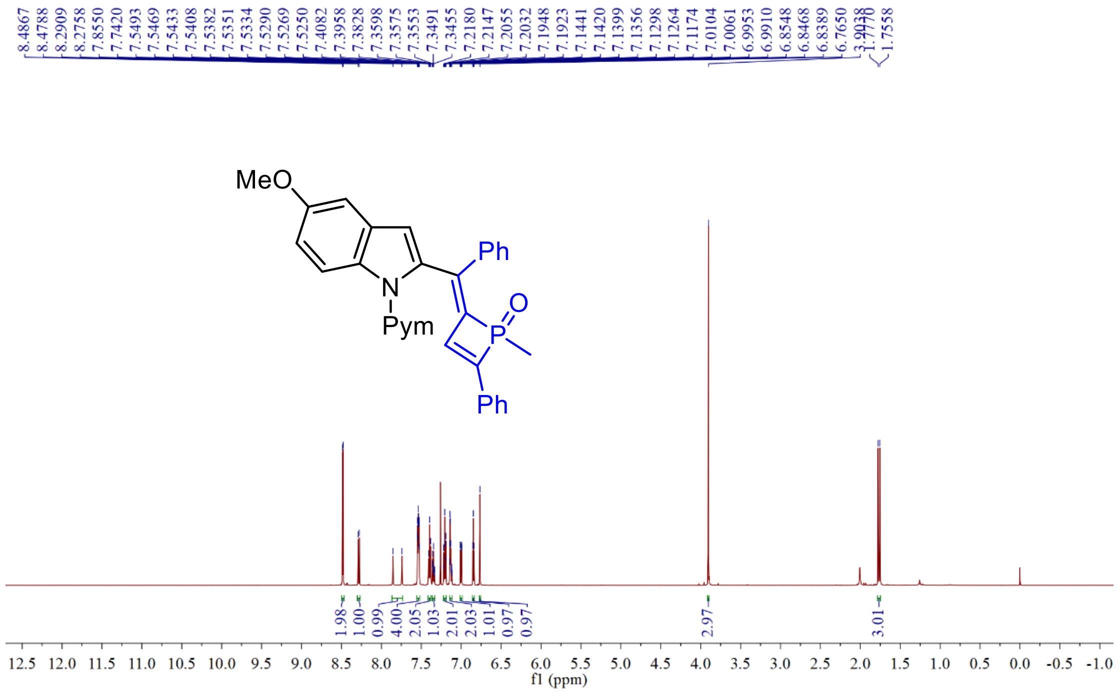

^{1}H NMR spectrum of compound **3i**

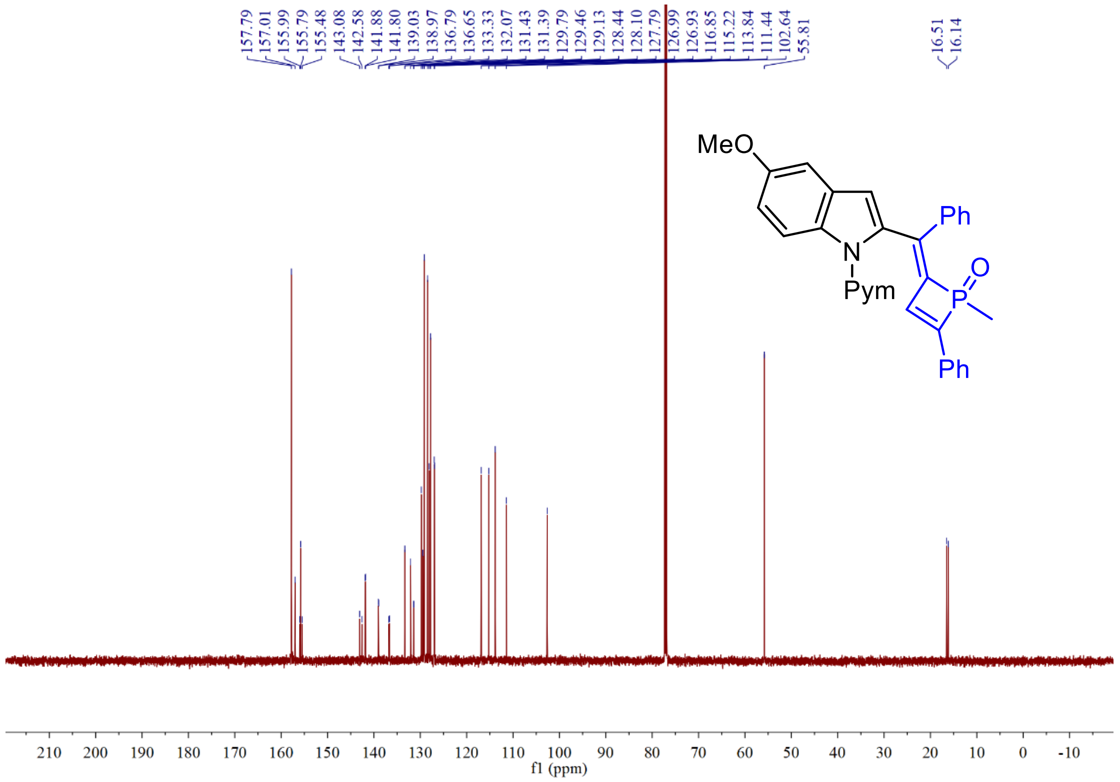

^{13}C NMR spectrum of compound **3i**

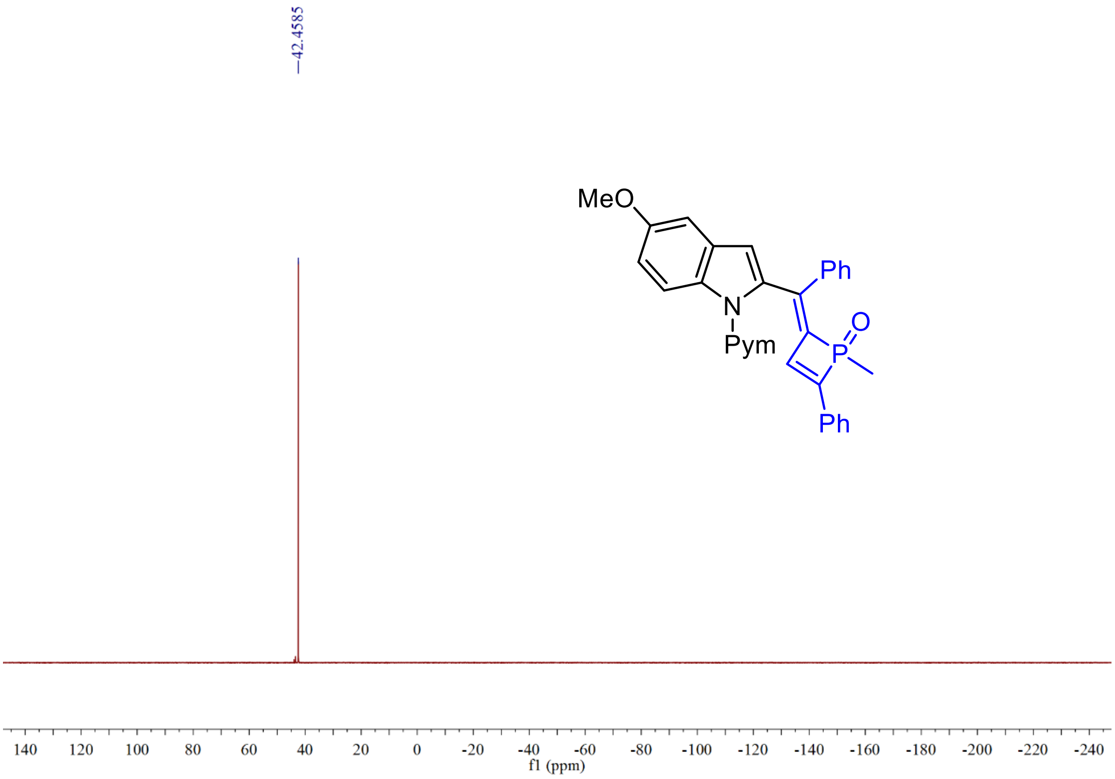

^{31}P NMR spectrum of compound **3i**

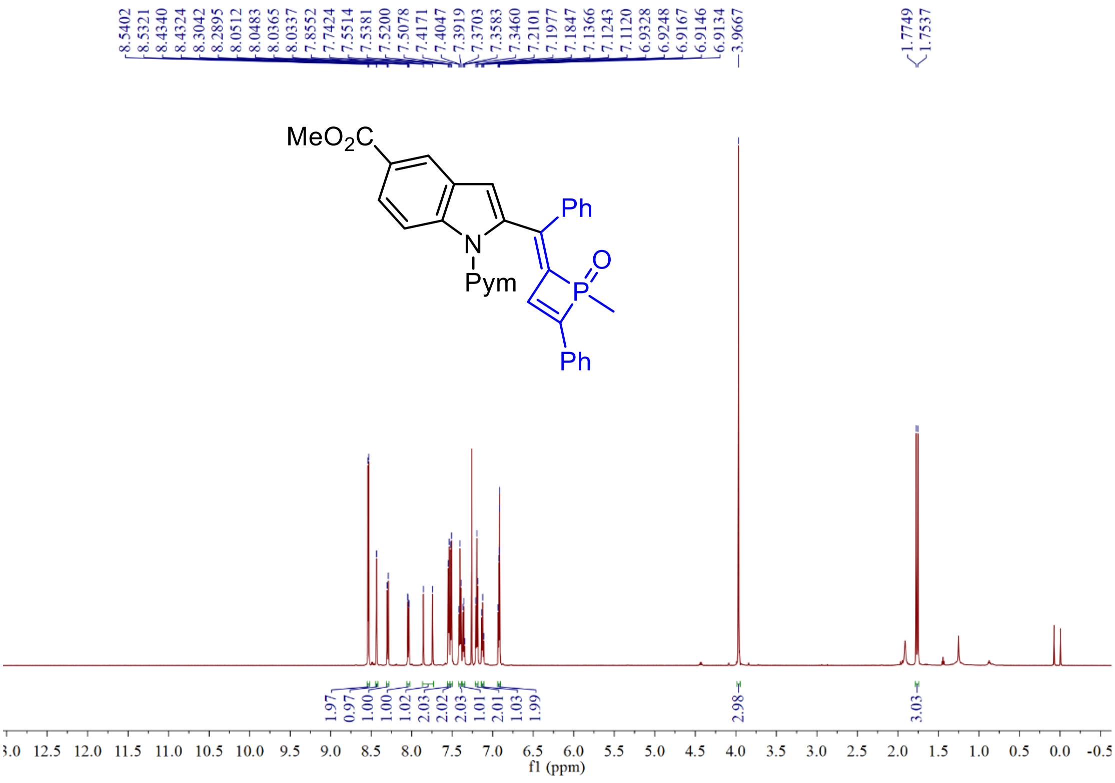

^{1}H NMR spectrum of compound **3j**

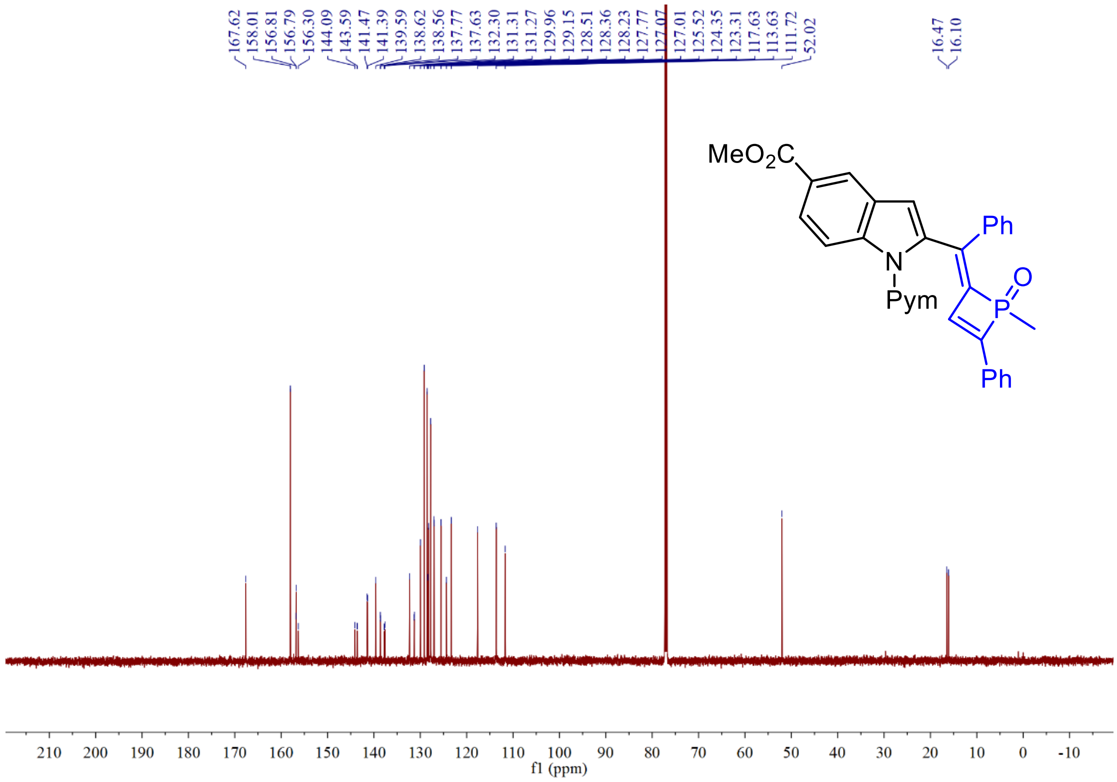

^{13}C NMR spectrum of compound **3j**

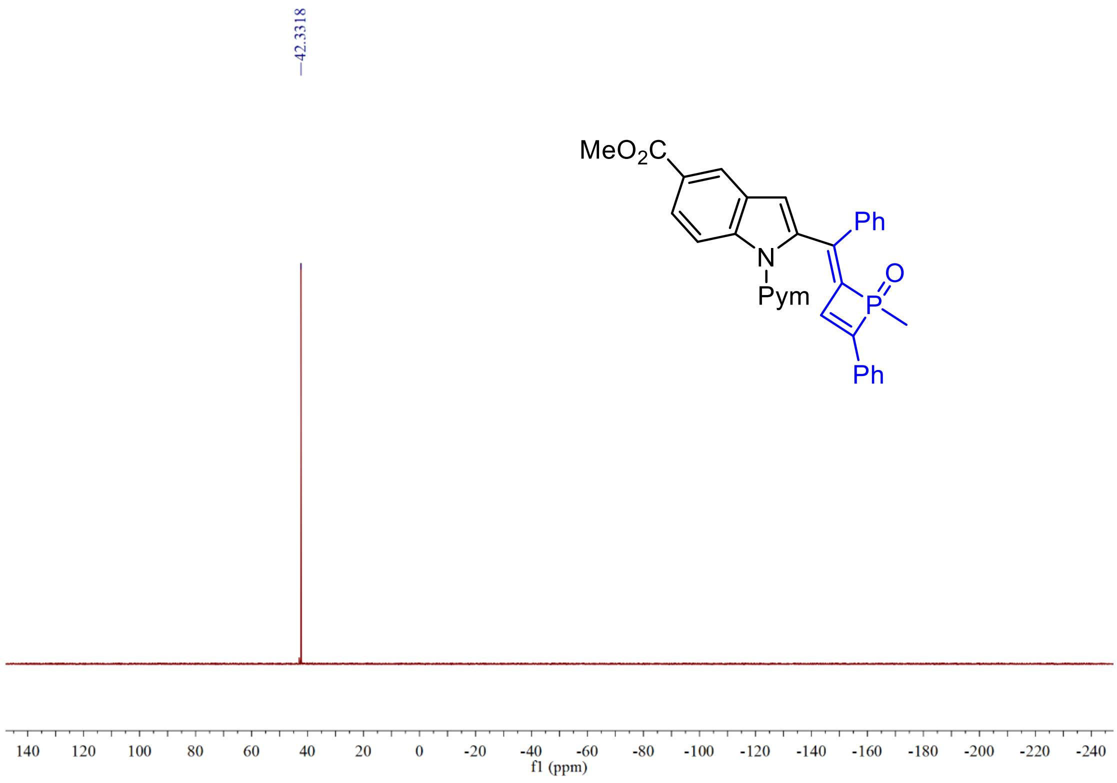

^{31}P NMR spectrum of compound **3j**

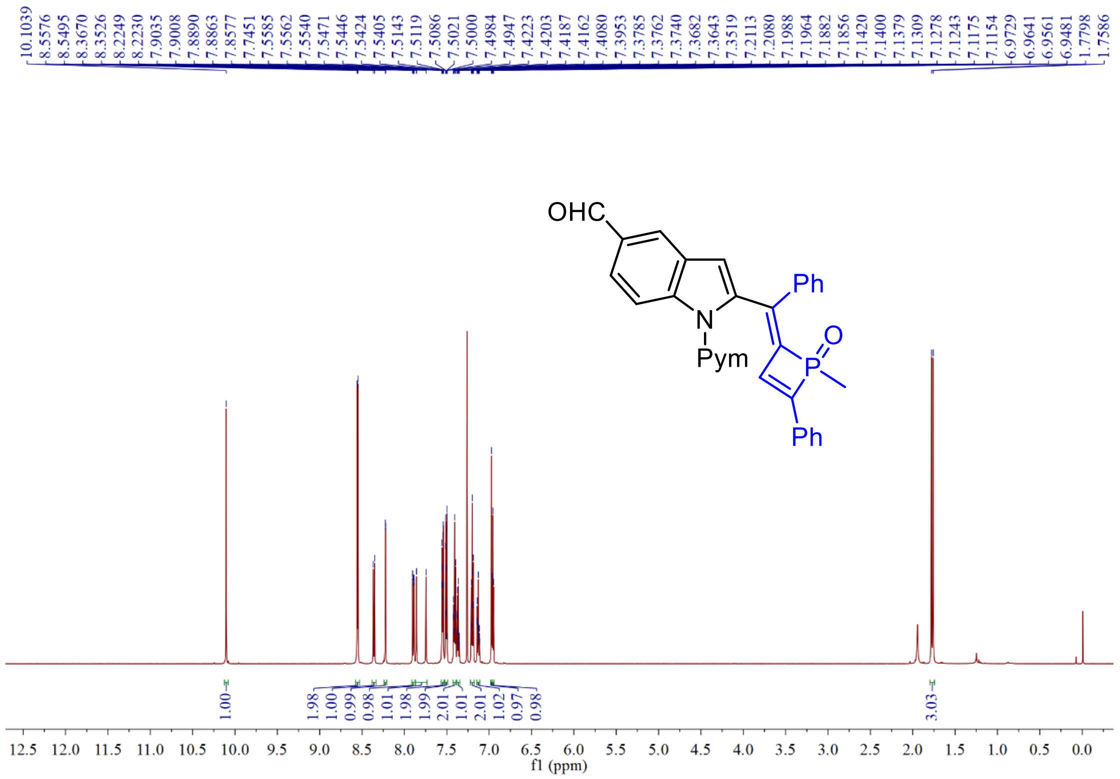

^{1}H NMR spectrum of compound **3k**

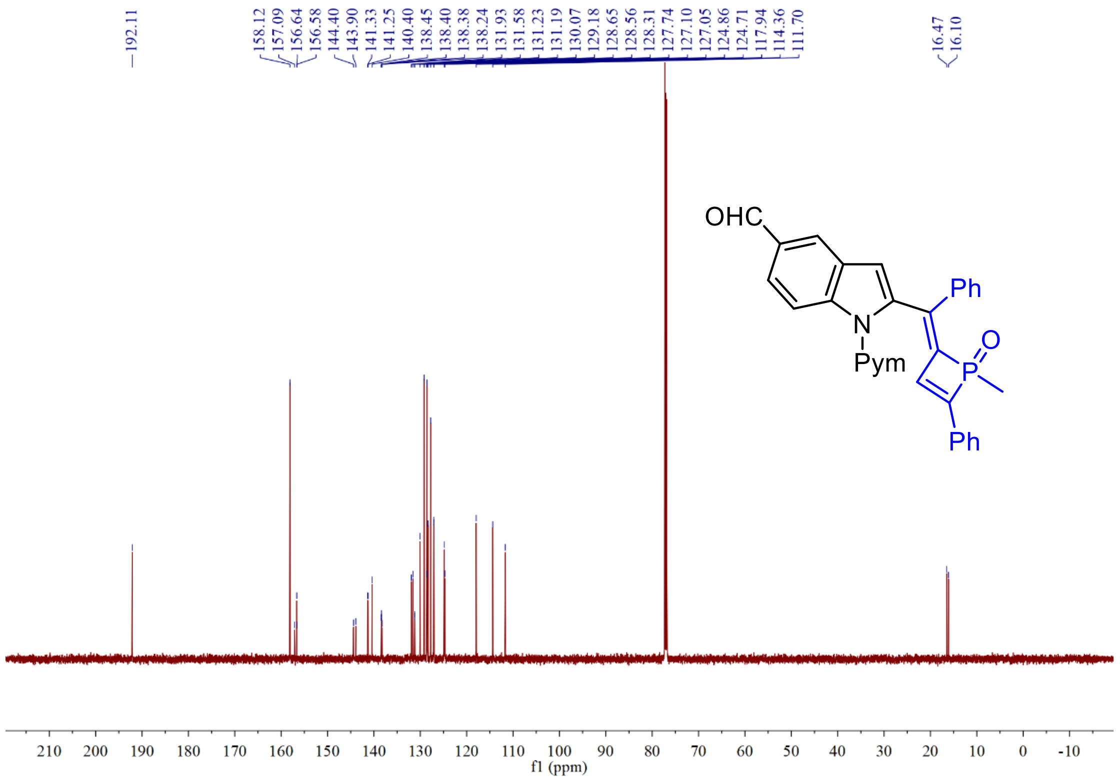

^{13}C NMR spectrum of compound **3k**

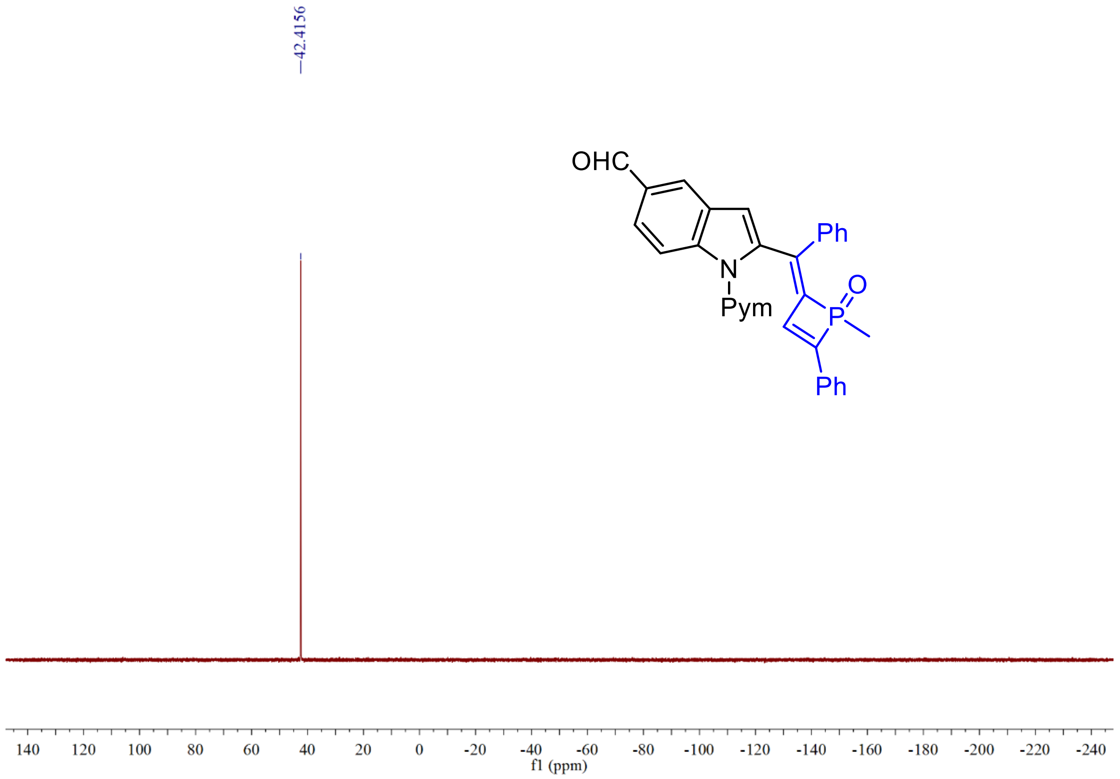

^{31}P NMR spectrum of compound **3k**

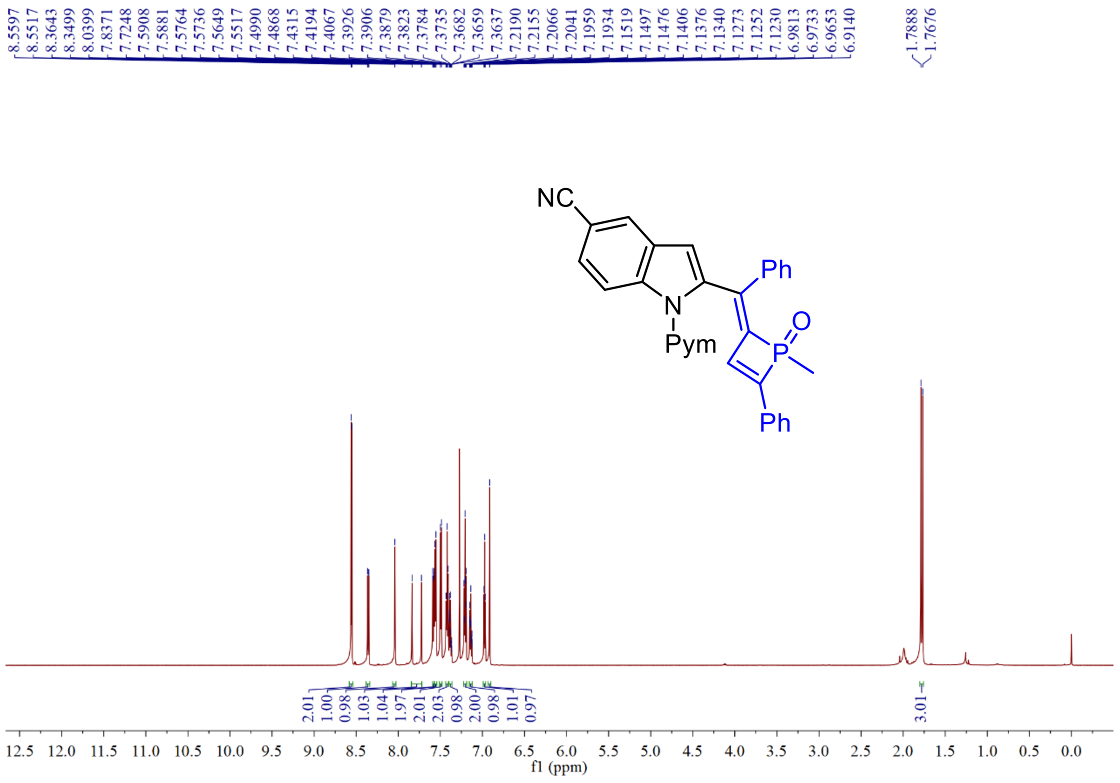

^{1}H NMR spectrum of compound **3l**

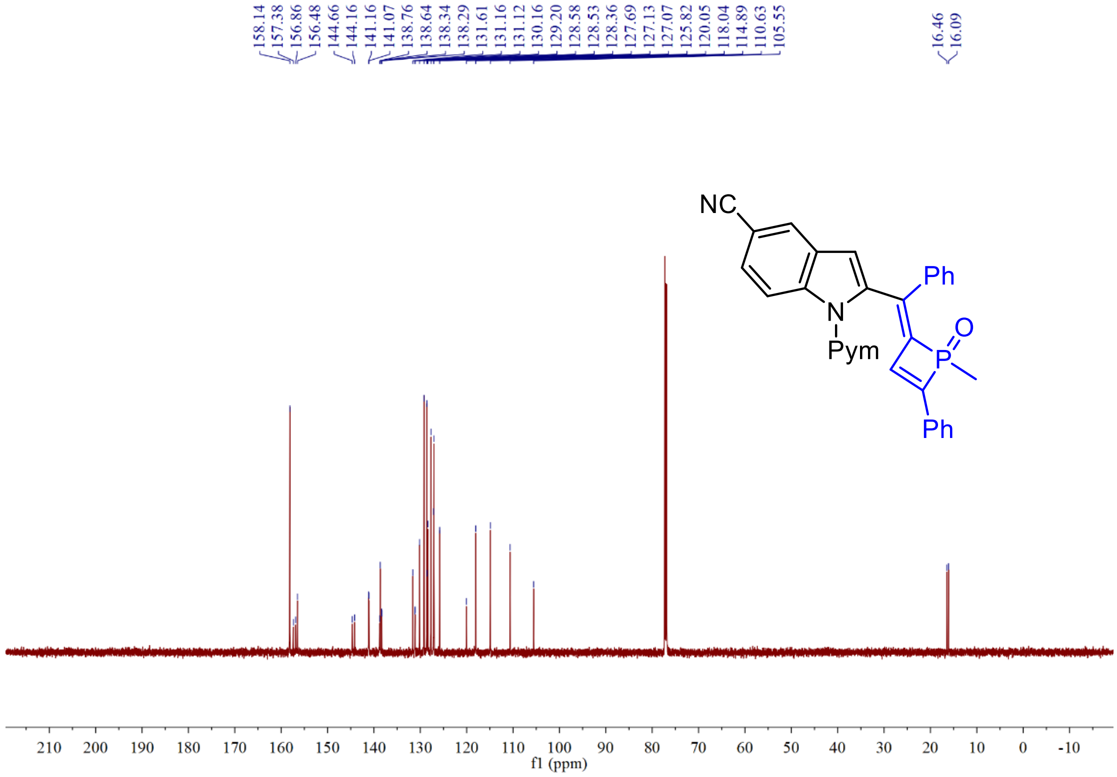

^{13}C NMR spectrum of compound **3l**

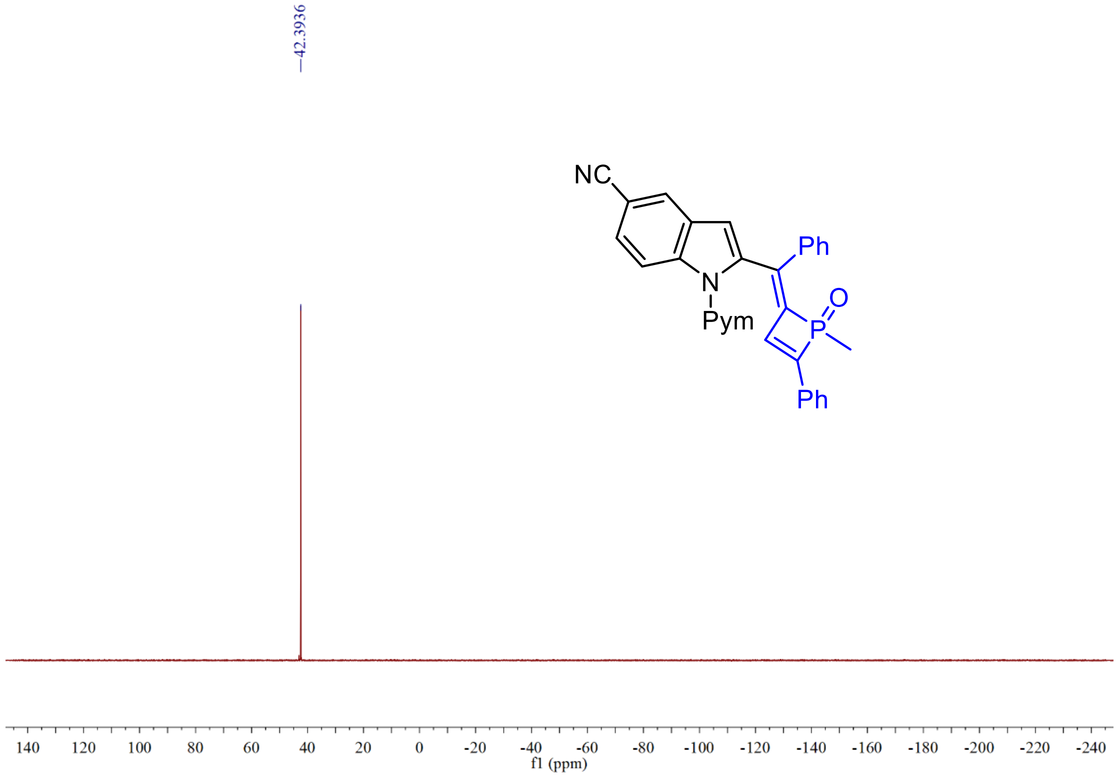

^{31}P NMR spectrum of compound **3l**

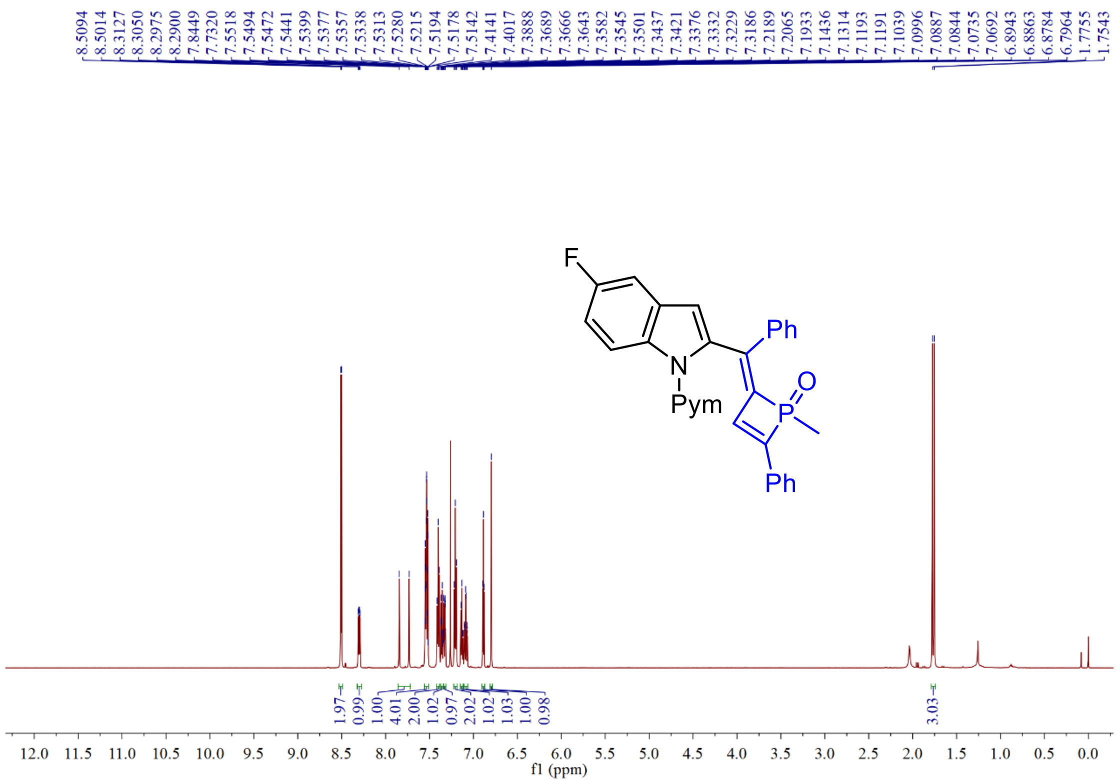

^{1}H NMR spectrum of compound **3m**

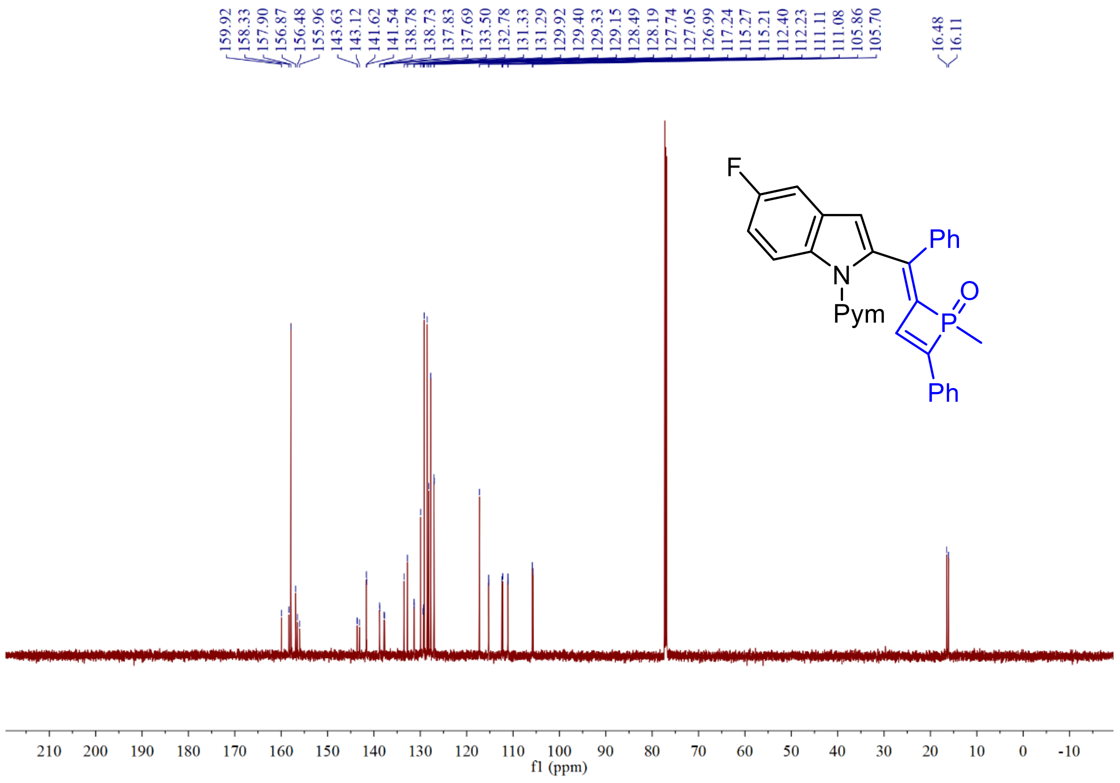

^{13}C NMR spectrum of compound **3m**

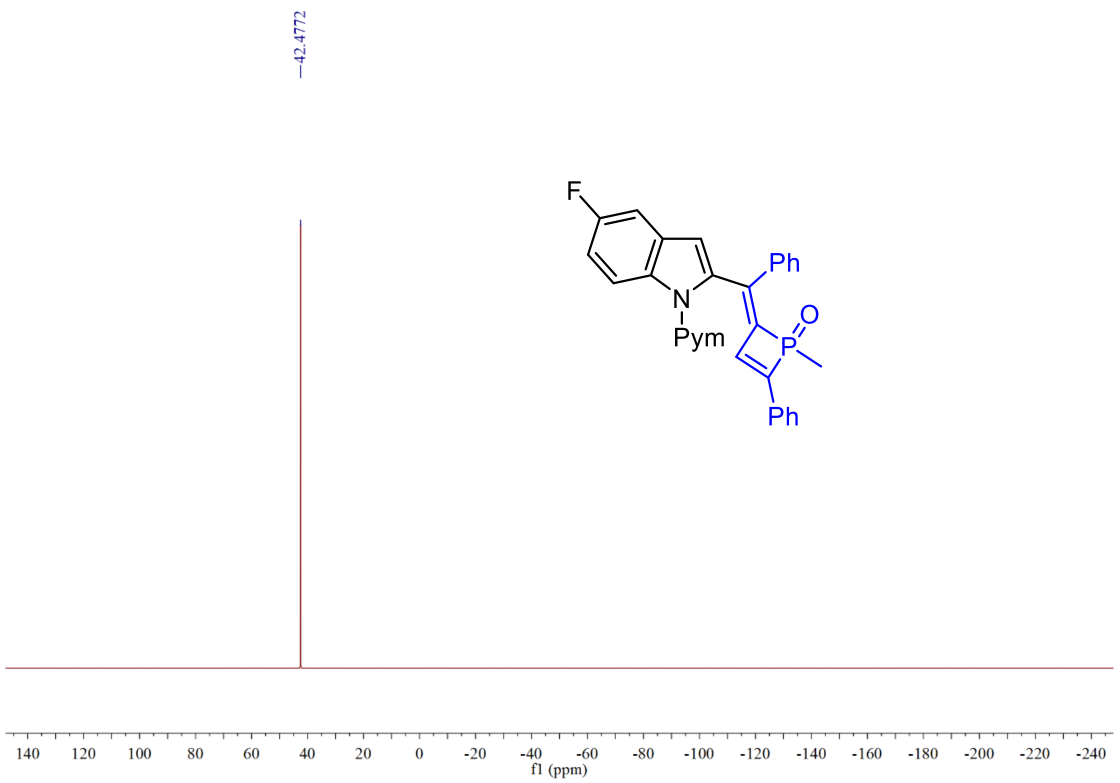

^{31}P NMR spectrum of compound **3m**

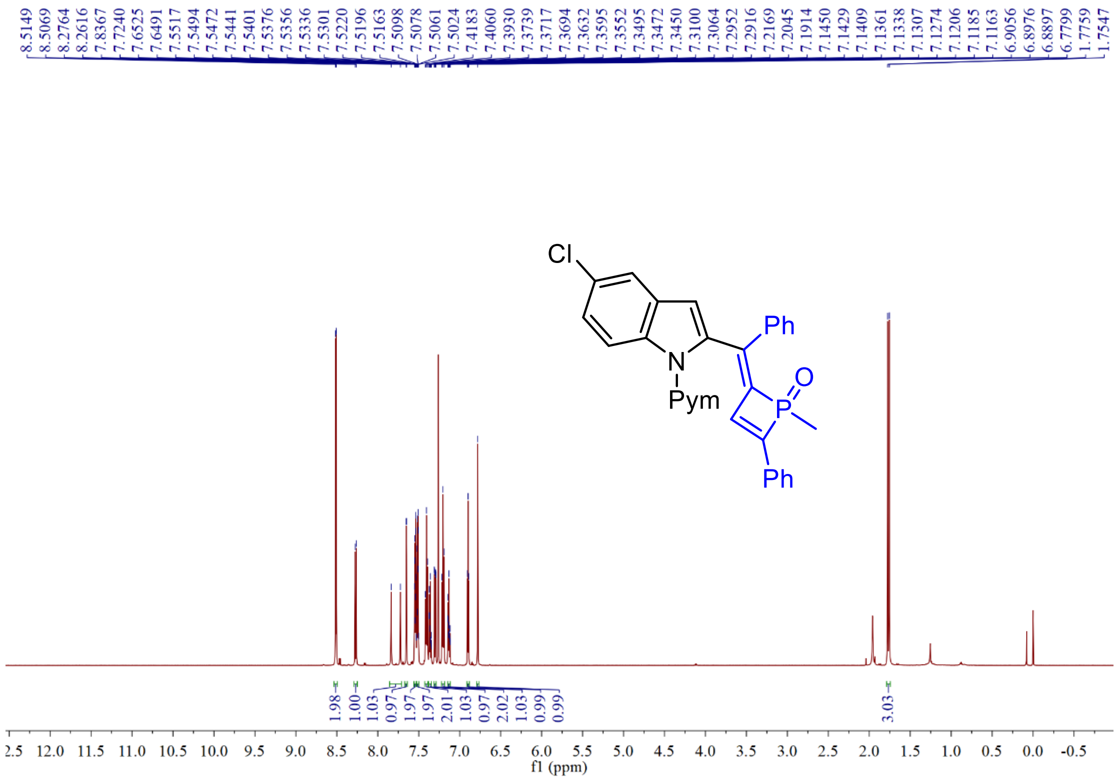

^{1}H NMR spectrum of compound **3n**

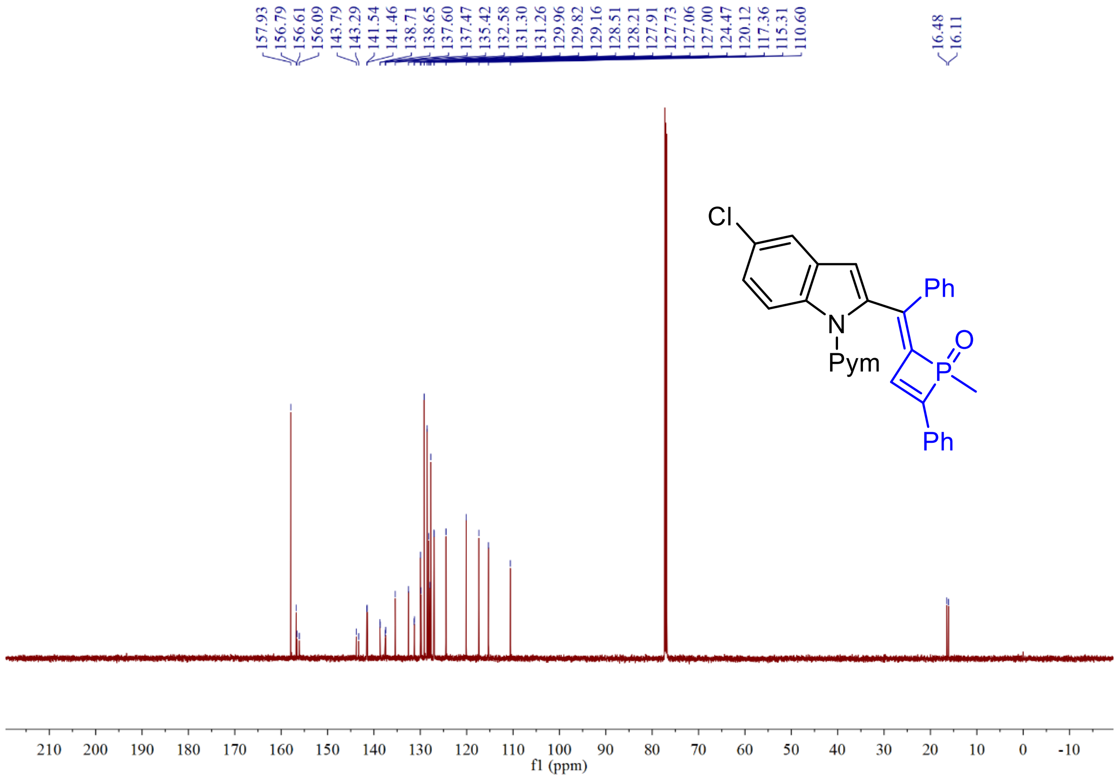

^{13}C NMR spectrum of compound **3n**

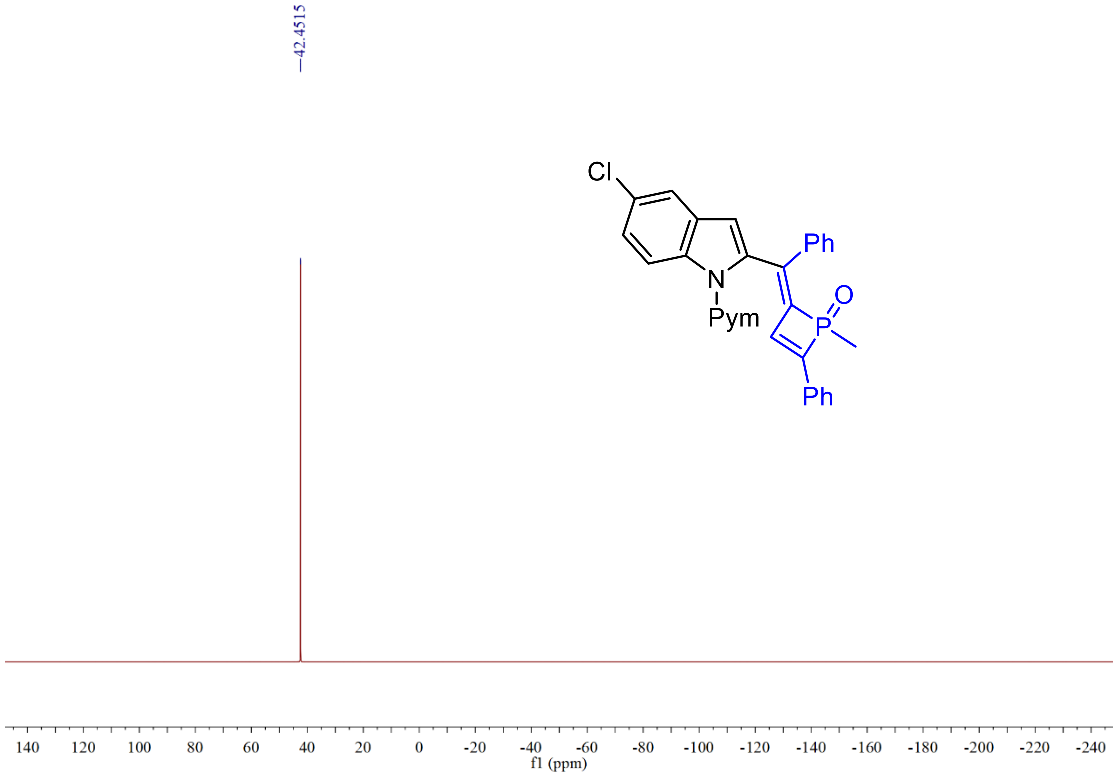

^{31}P NMR spectrum of compound **3n**

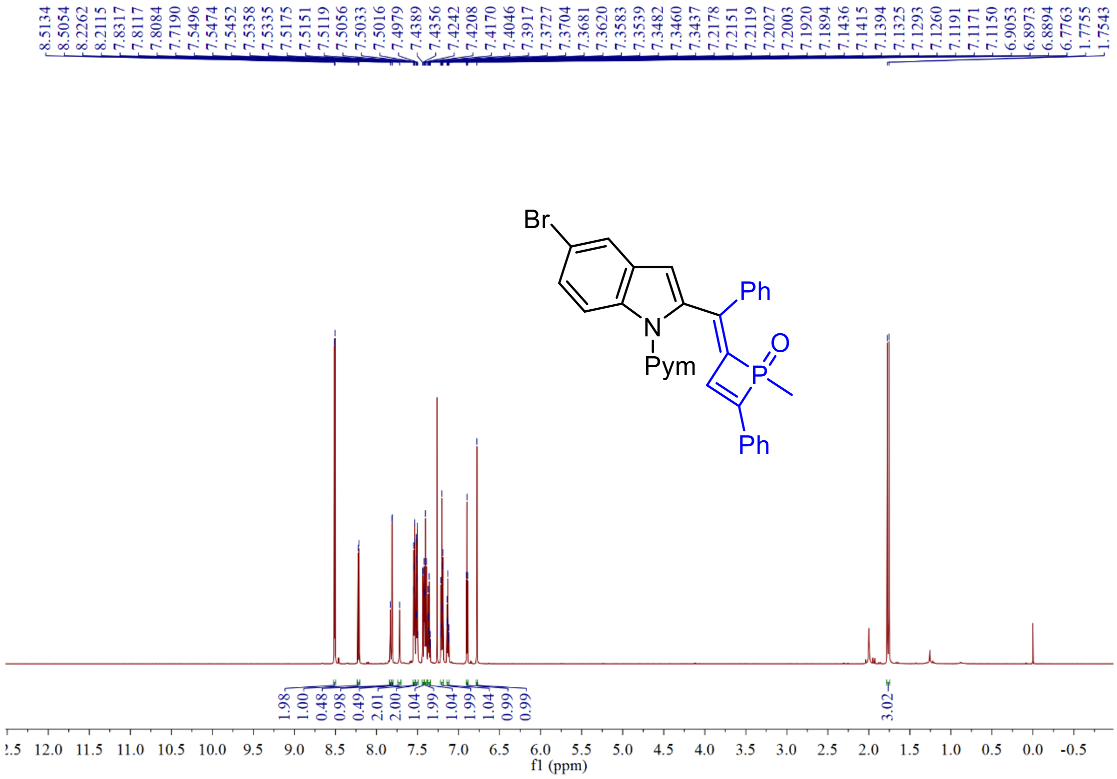

^{1}H NMR spectrum of compound **3o**

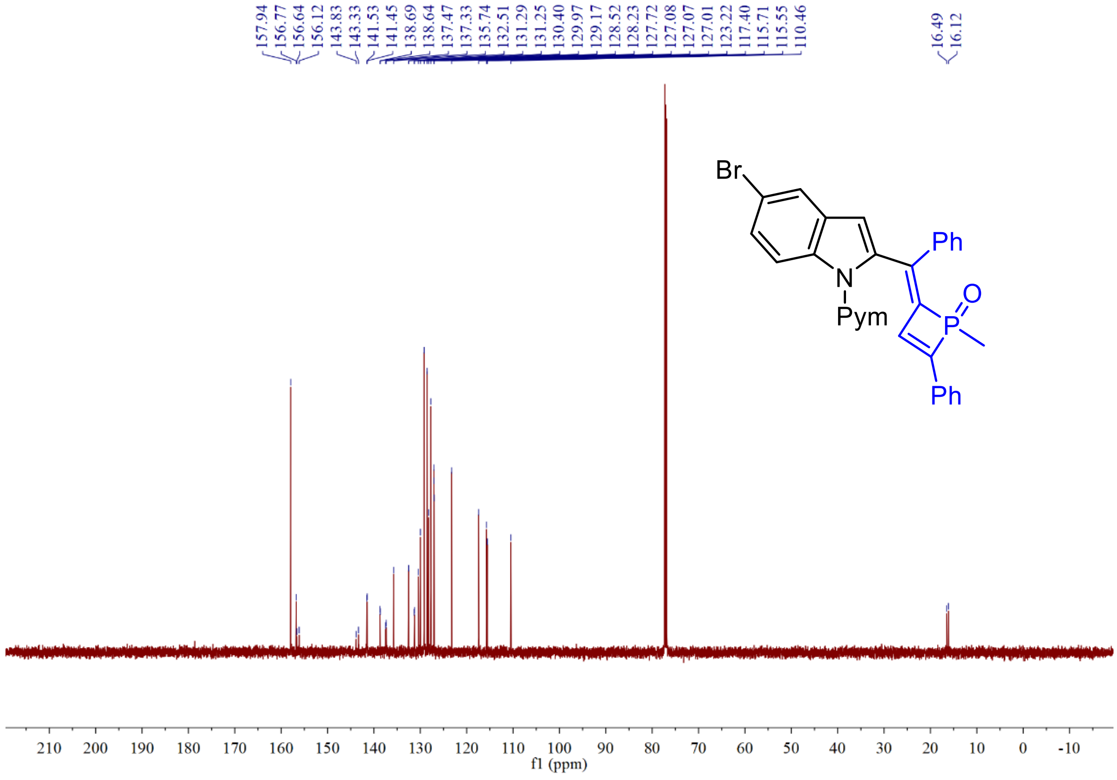

^{13}C NMR spectrum of compound **3o**

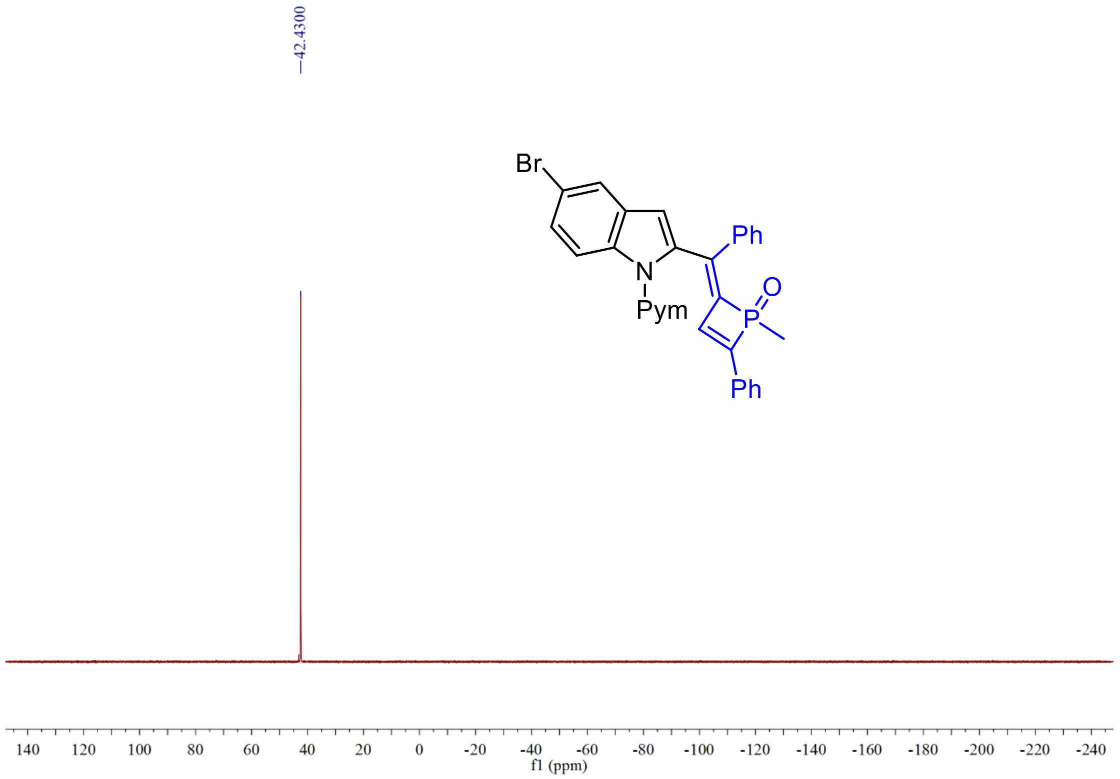

^{31}P NMR spectrum of compound **3o**

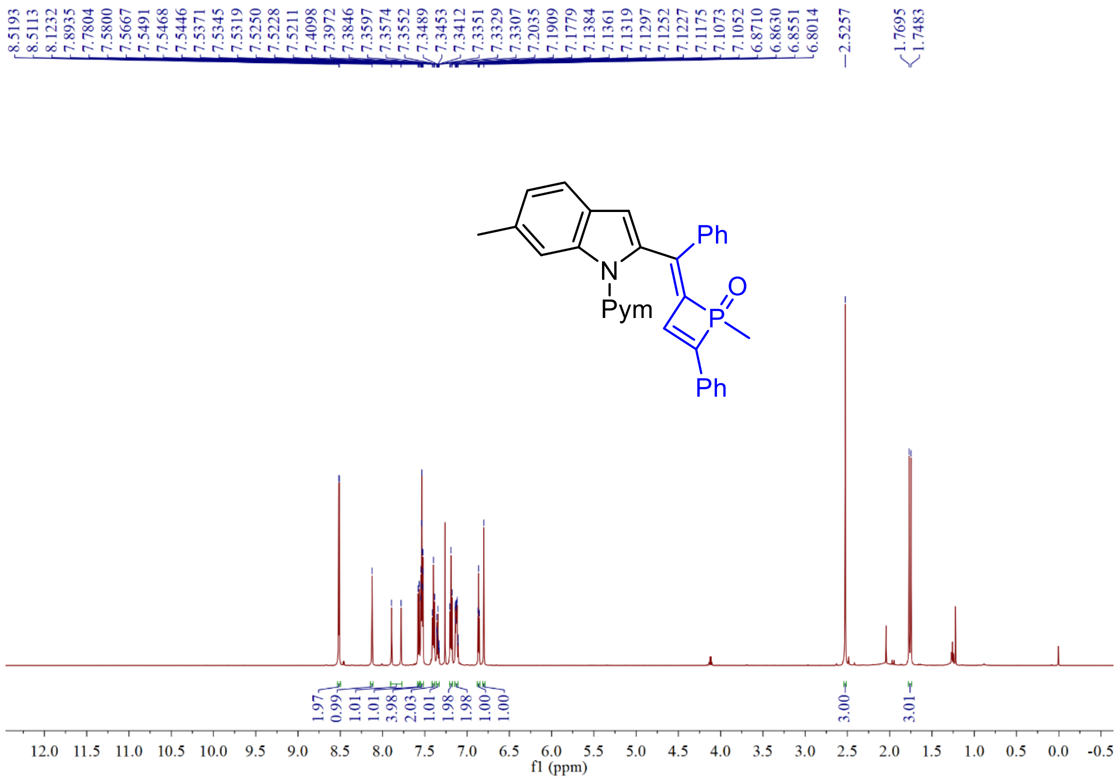

^{1}H NMR spectrum of compound **3p**

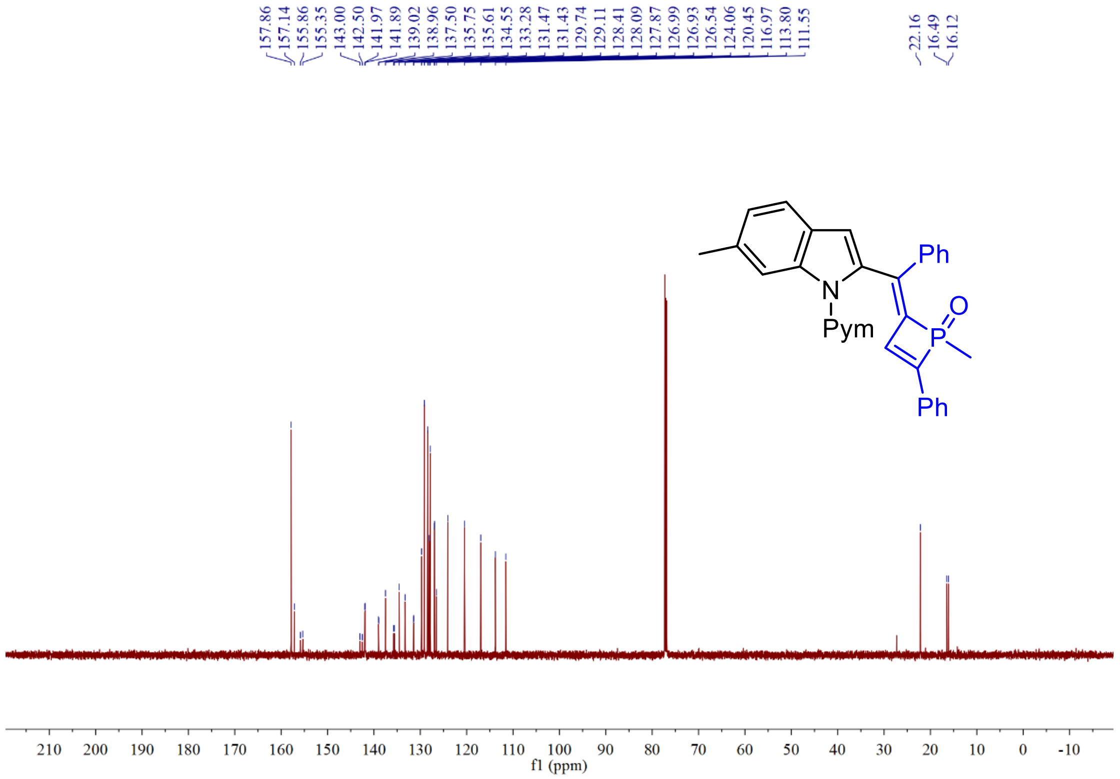

^{13}C NMR spectrum of compound **3p**

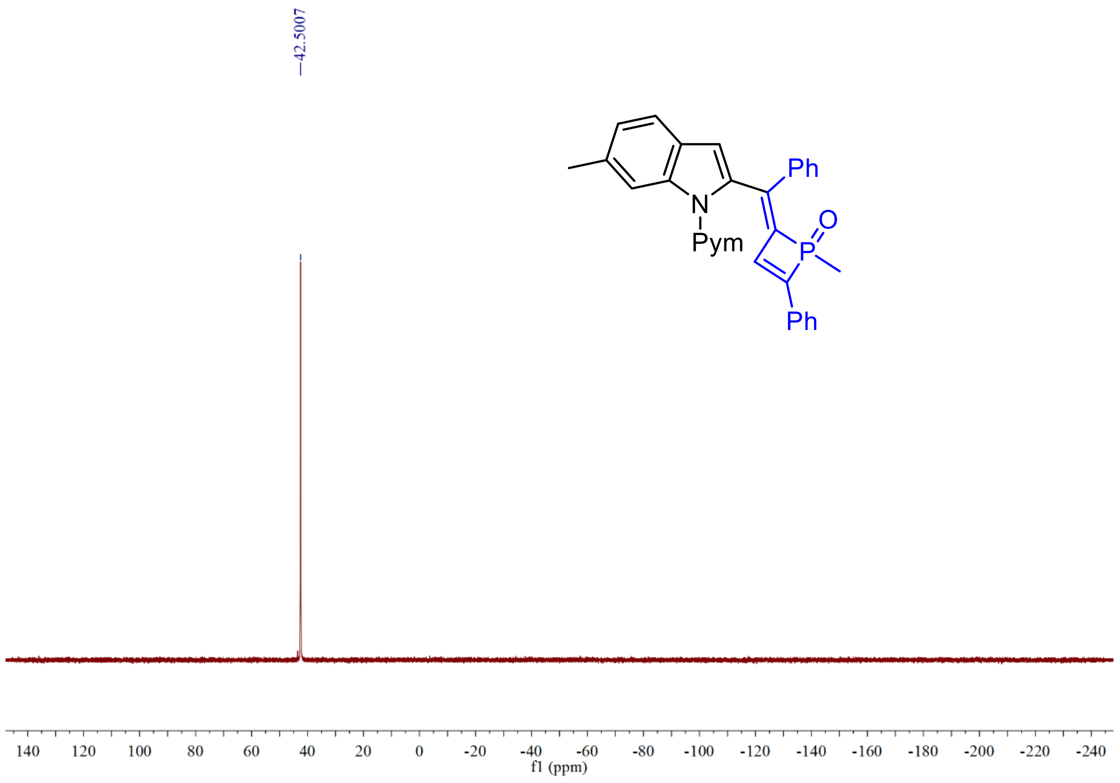

^{31}P NMR spectrum of compound **3p**

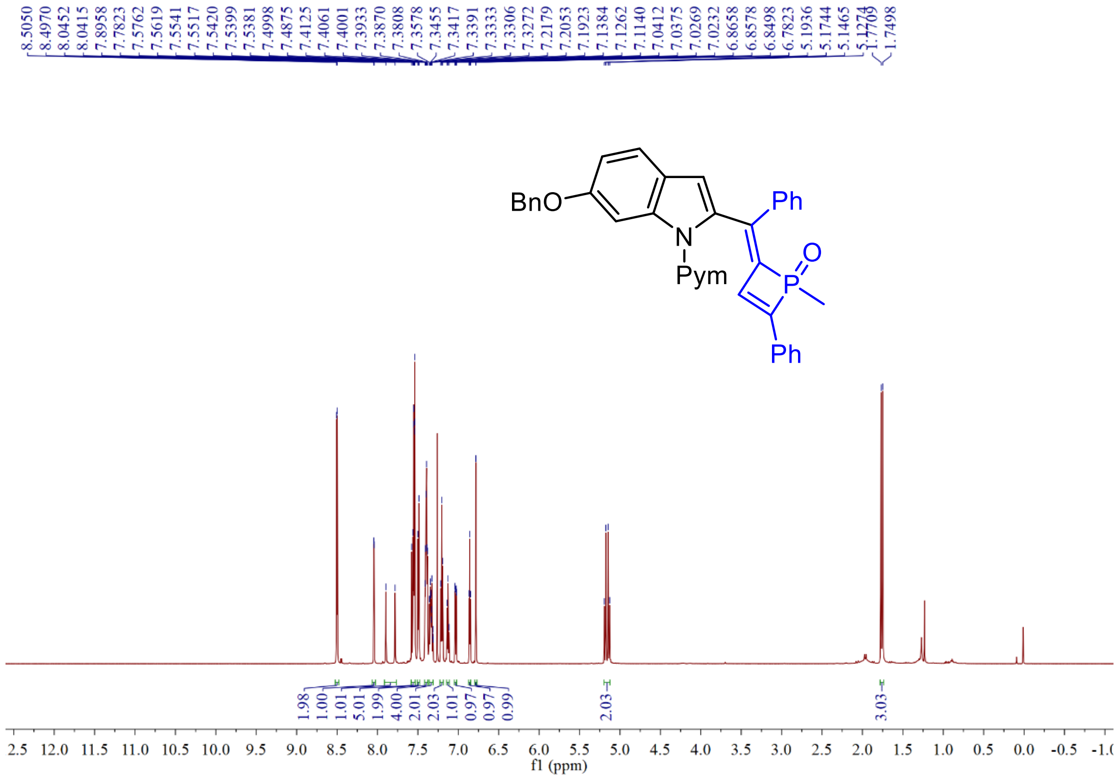

^{1}H NMR spectrum of compound **3q**

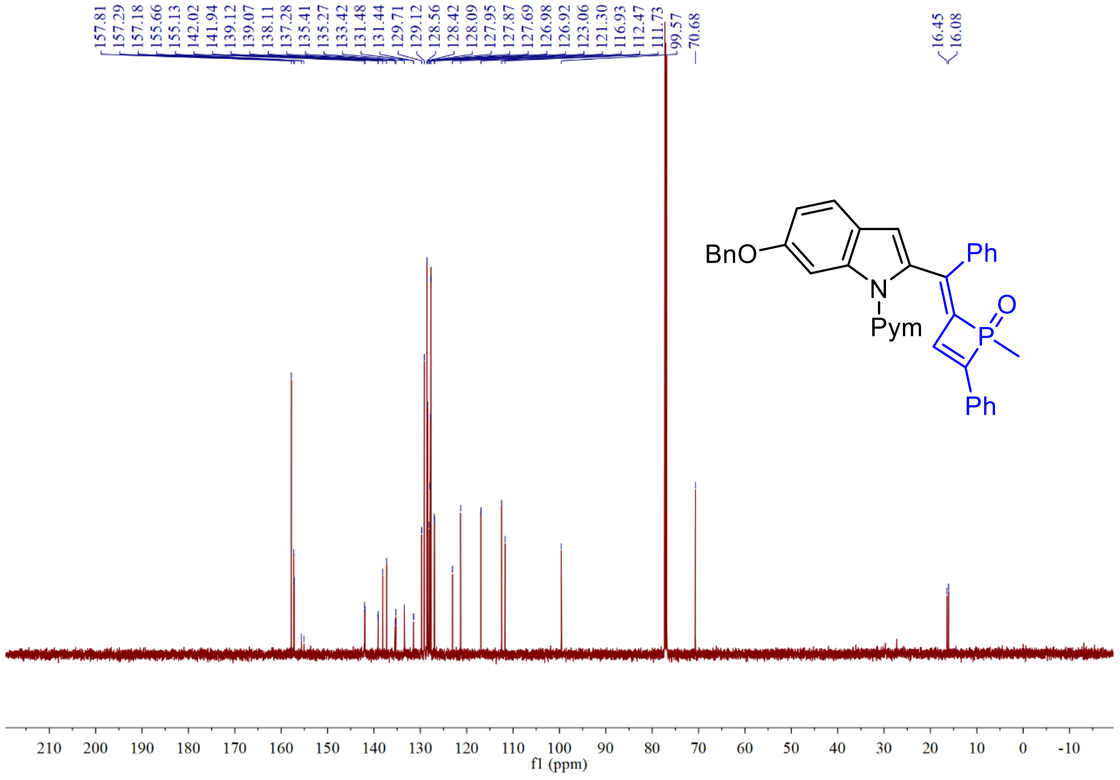

^{13}C NMR spectrum of compound **3q**

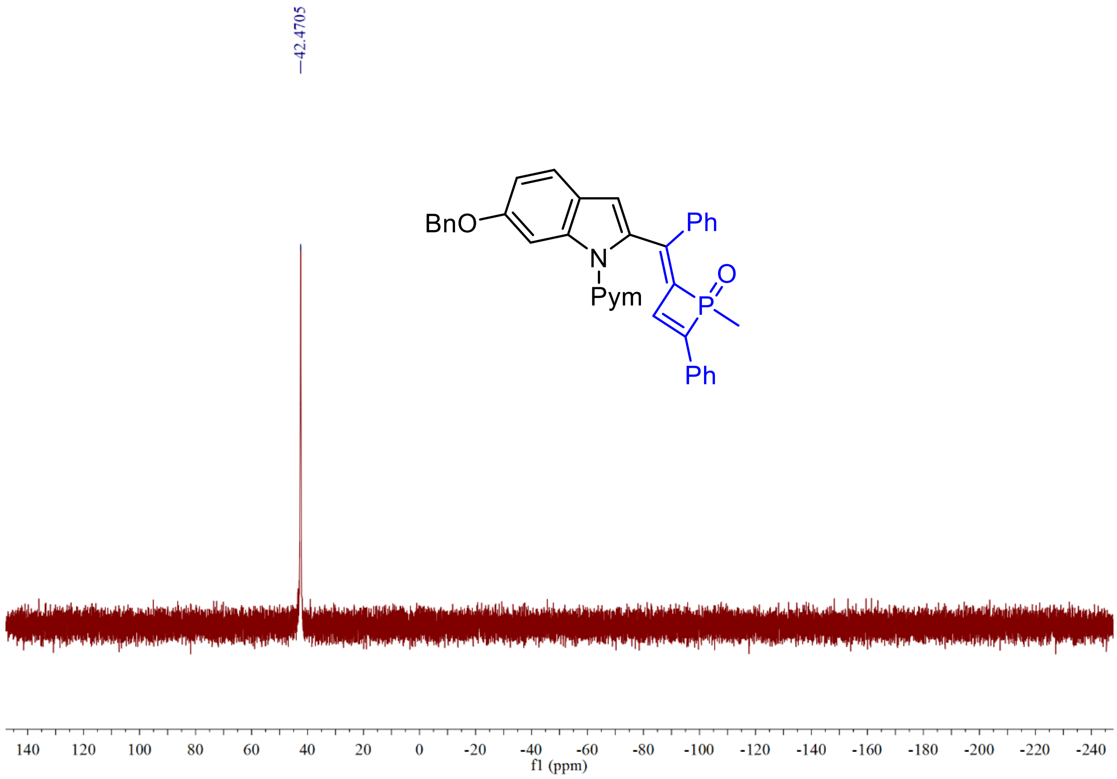

^{31}P NMR spectrum of compound **3q**

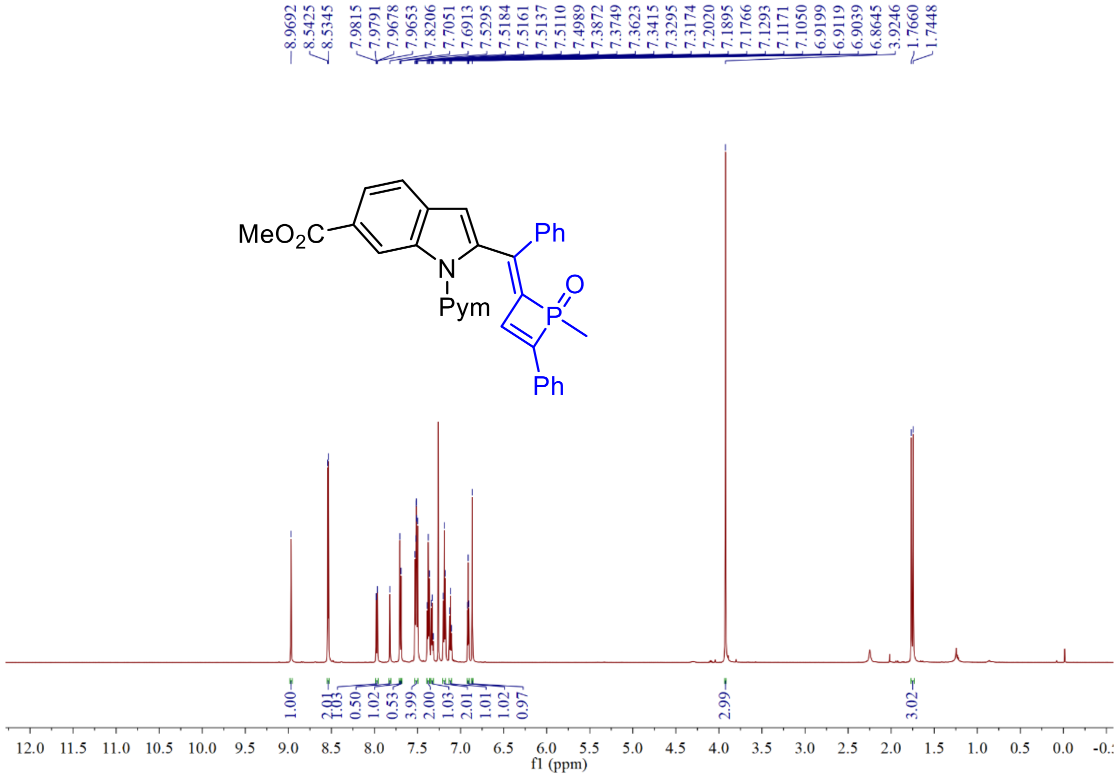

^{1}H NMR spectrum of compound **3r**

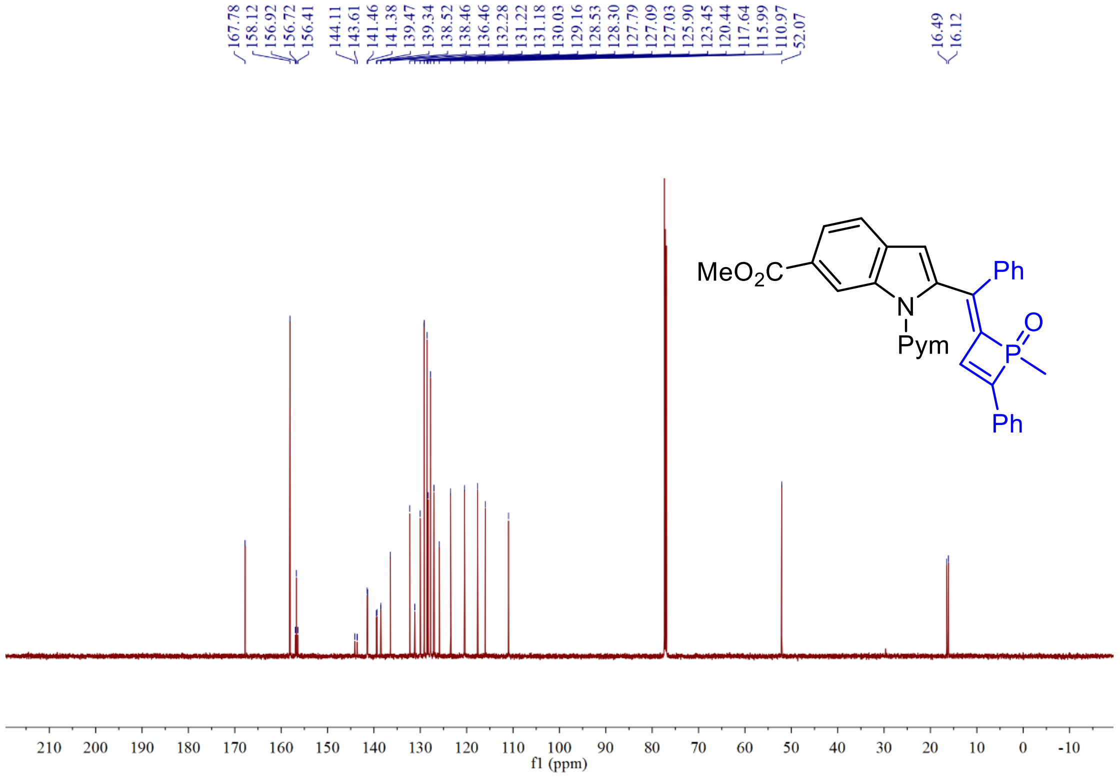

^{13}C NMR spectrum of compound **3r**

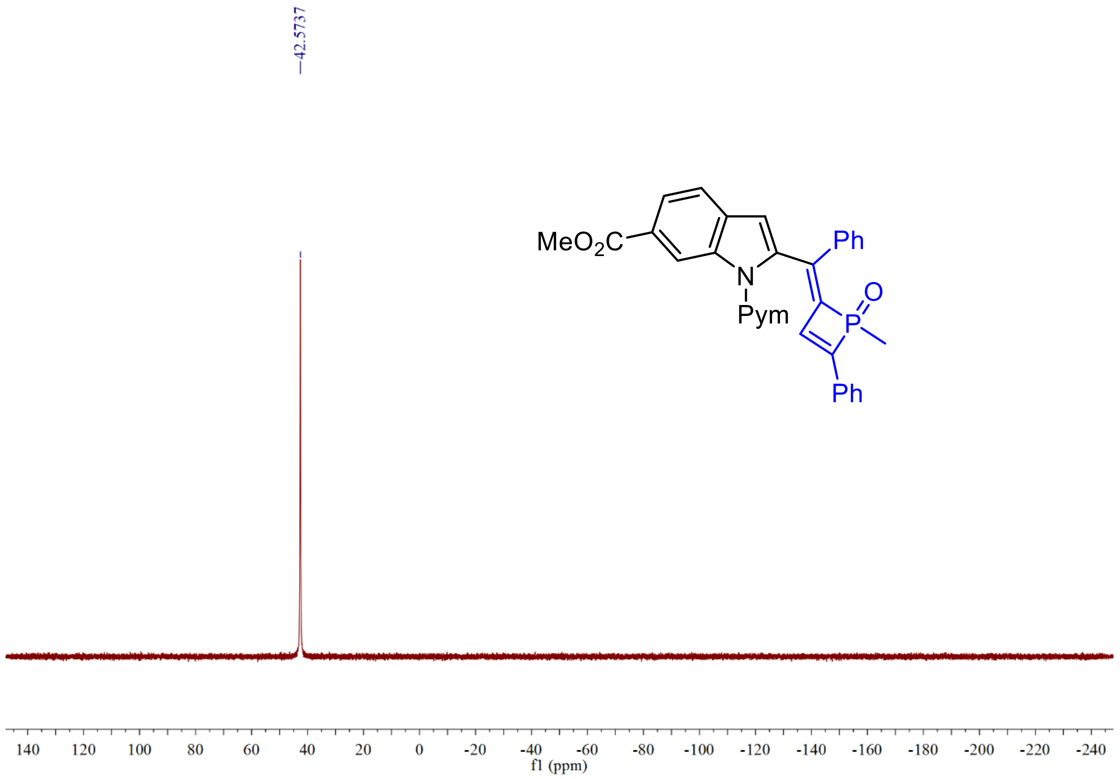

^{31}P NMR spectrum of compound **3r**

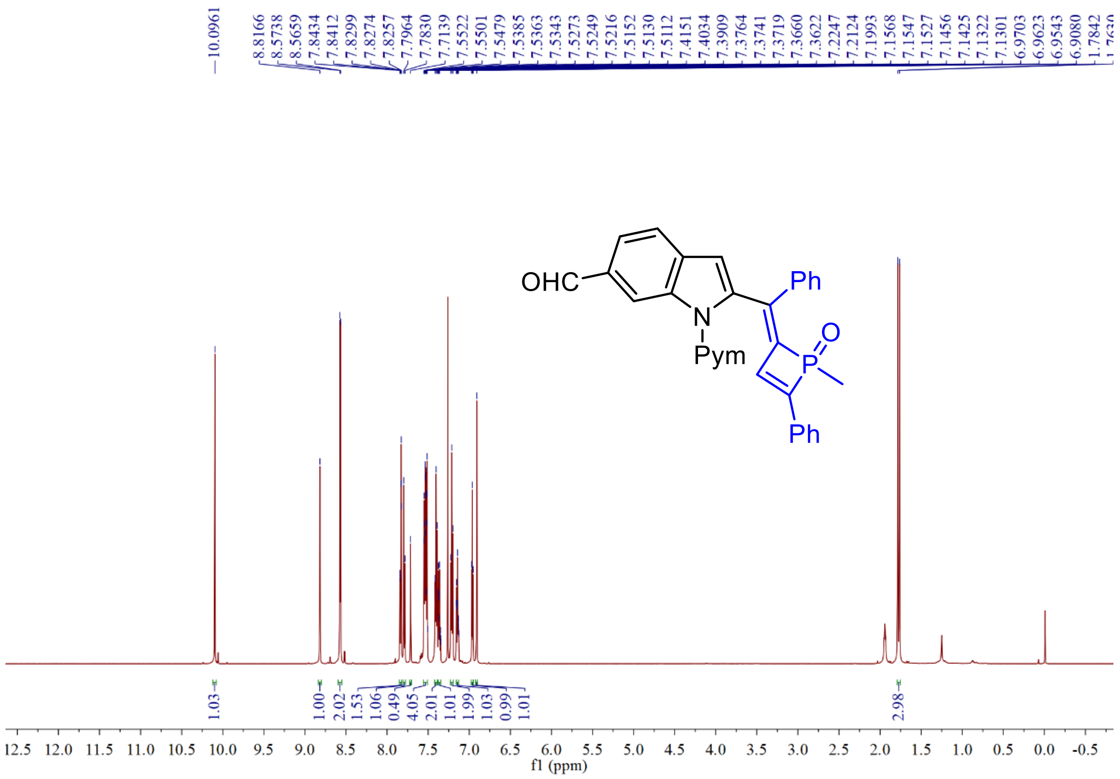

^{1}H NMR spectrum of compound **3s**

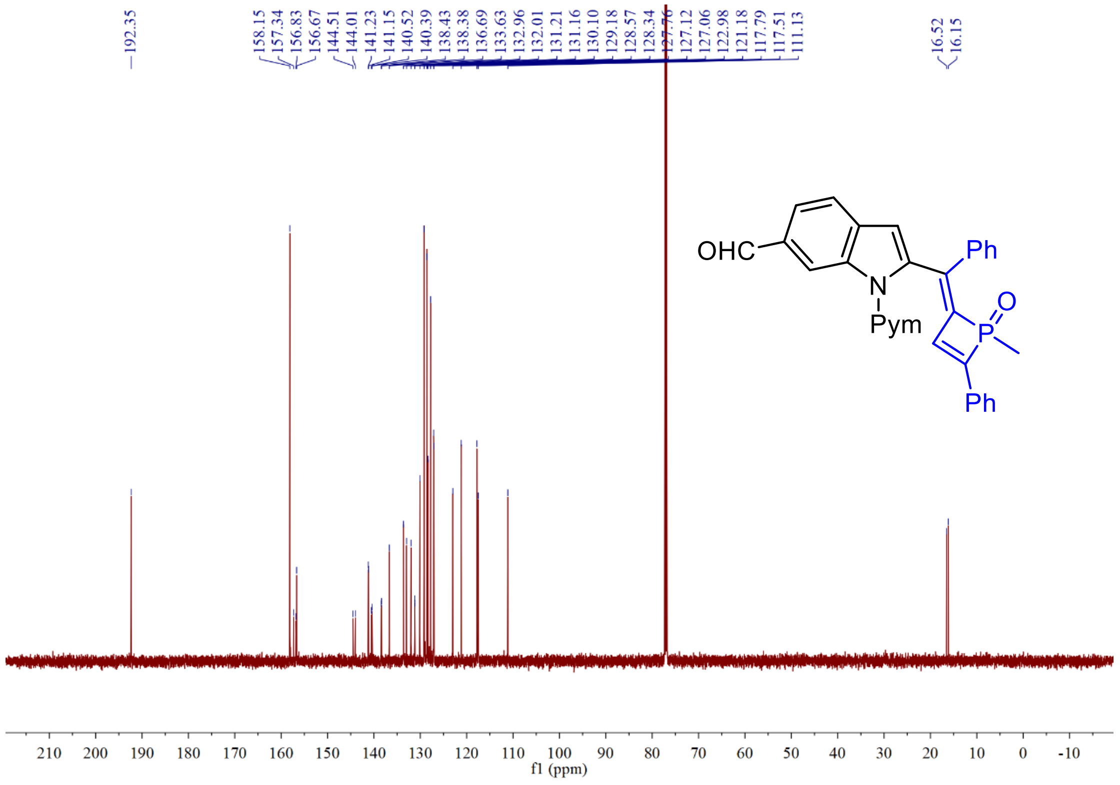

^{13}C NMR spectrum of compound **3s**

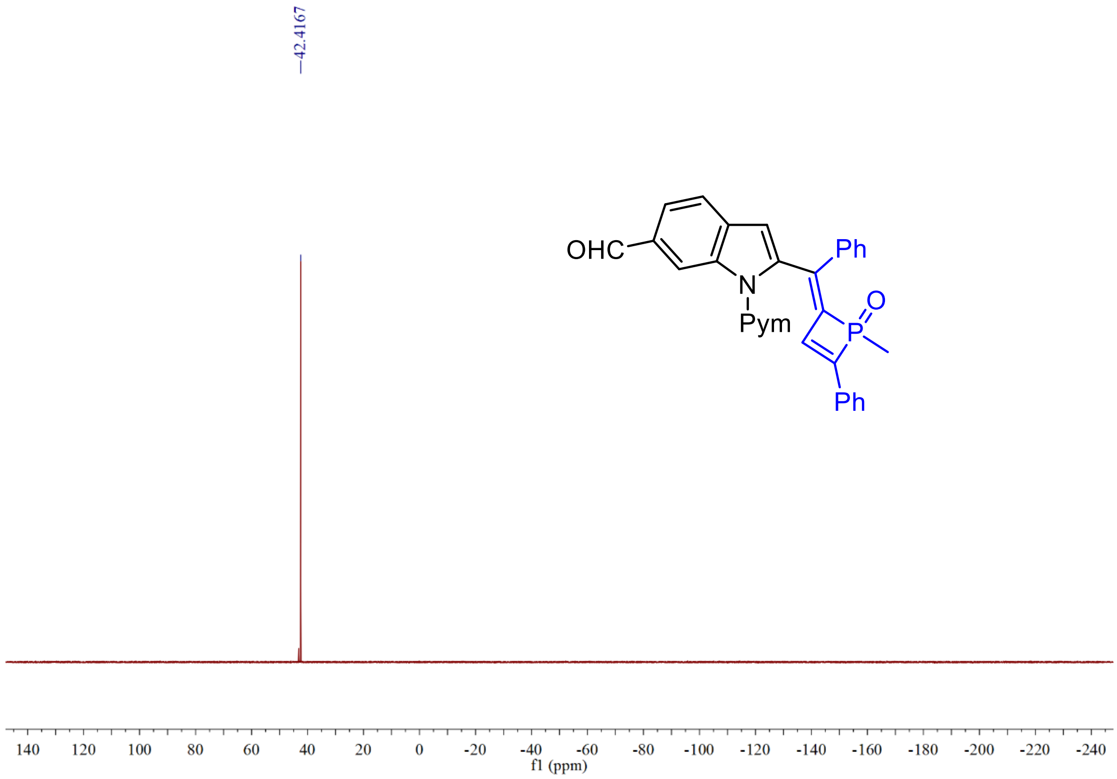

^{31}P NMR spectrum of compound **3s**

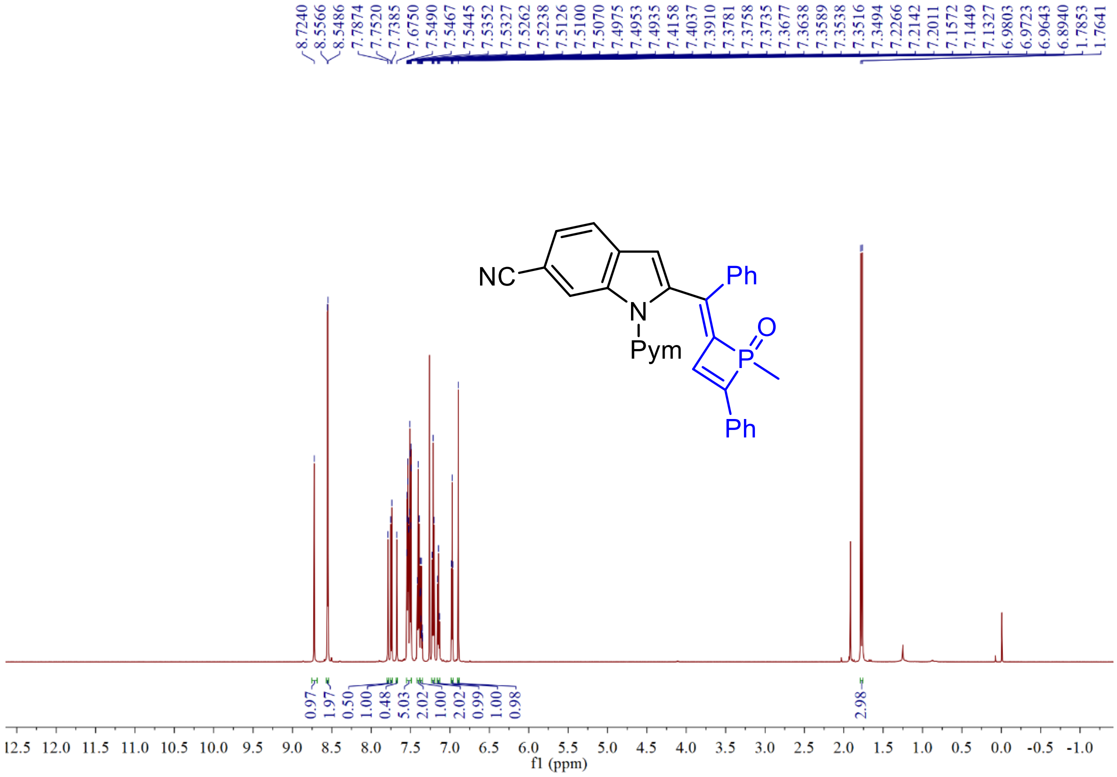

^{1}H NMR spectrum of compound **3t**

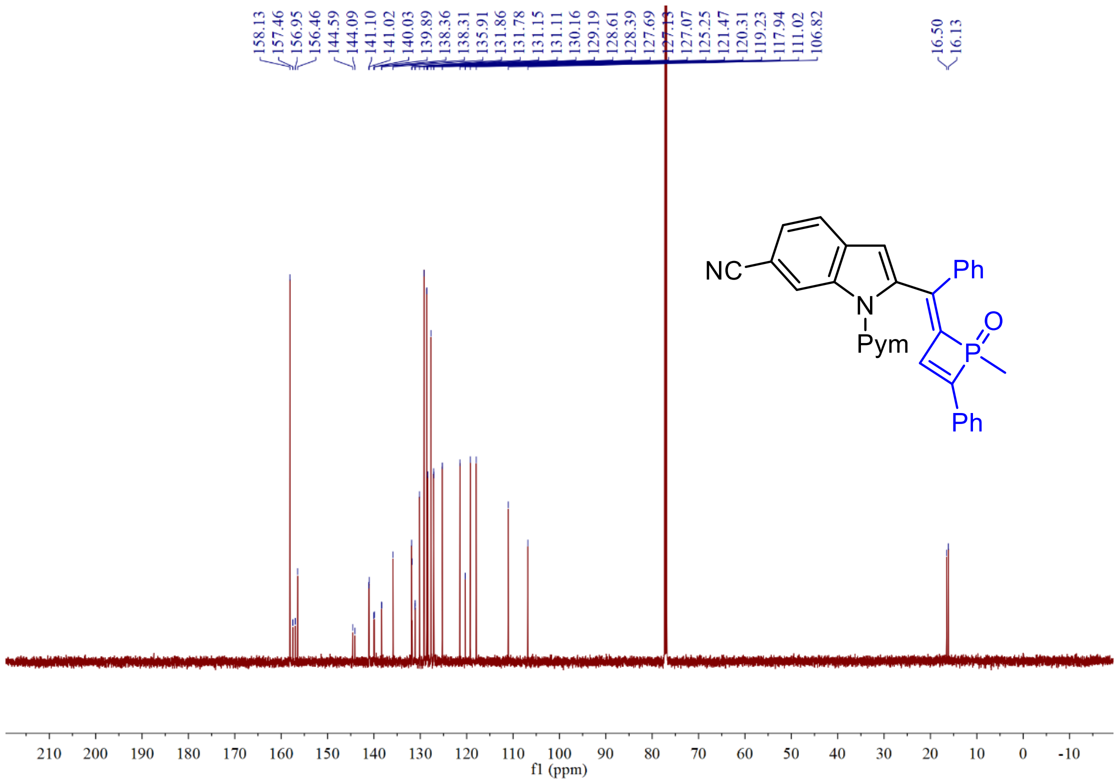

^{13}C NMR spectrum of compound **3t**

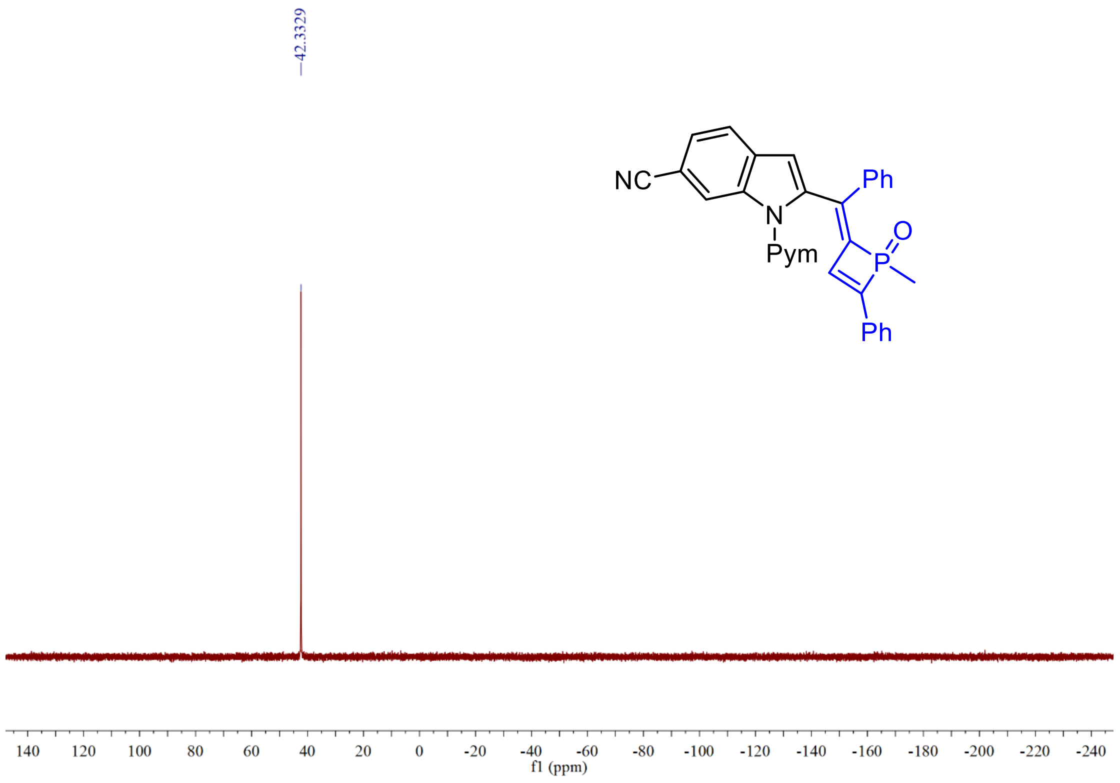

^{31}P NMR spectrum of compound **3t**

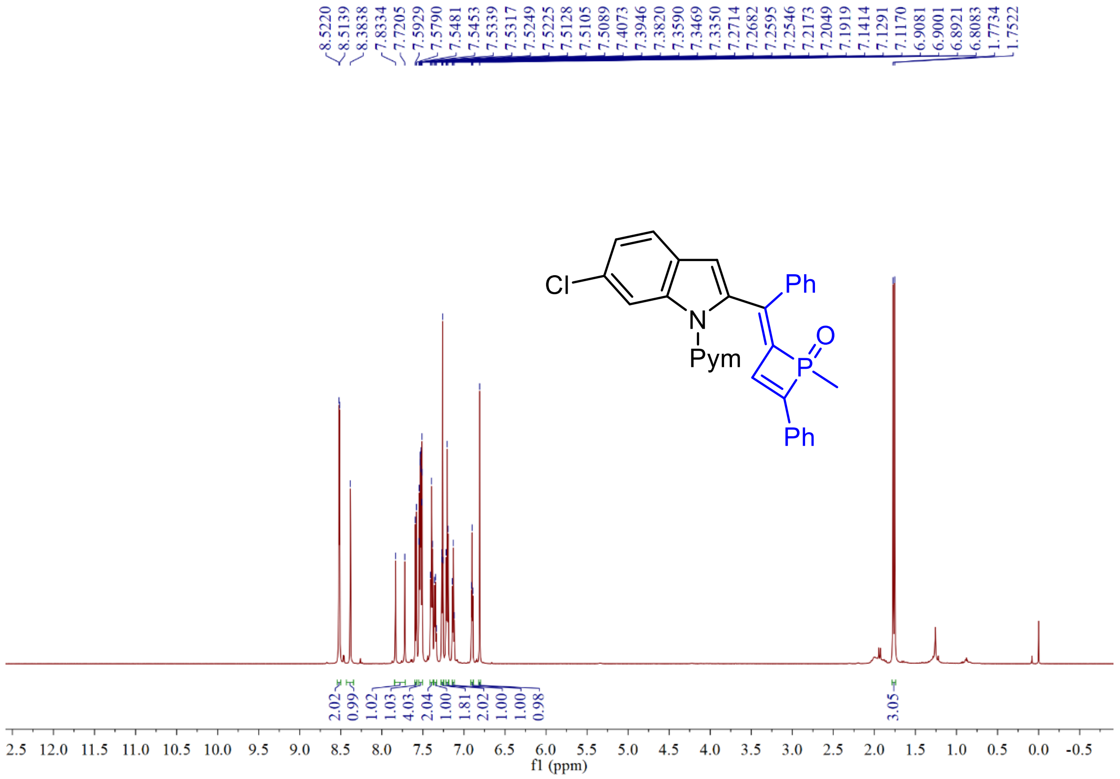

^{1}H NMR spectrum of compound **3u**

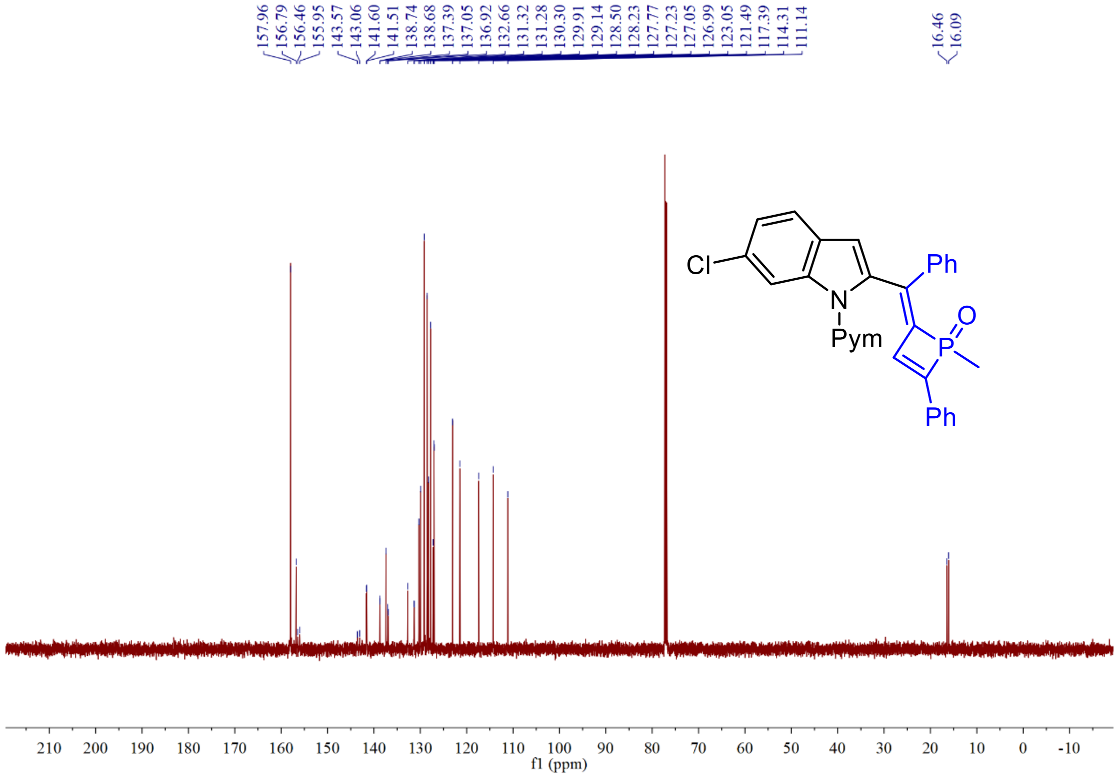

^{13}C NMR spectrum of compound **3u**

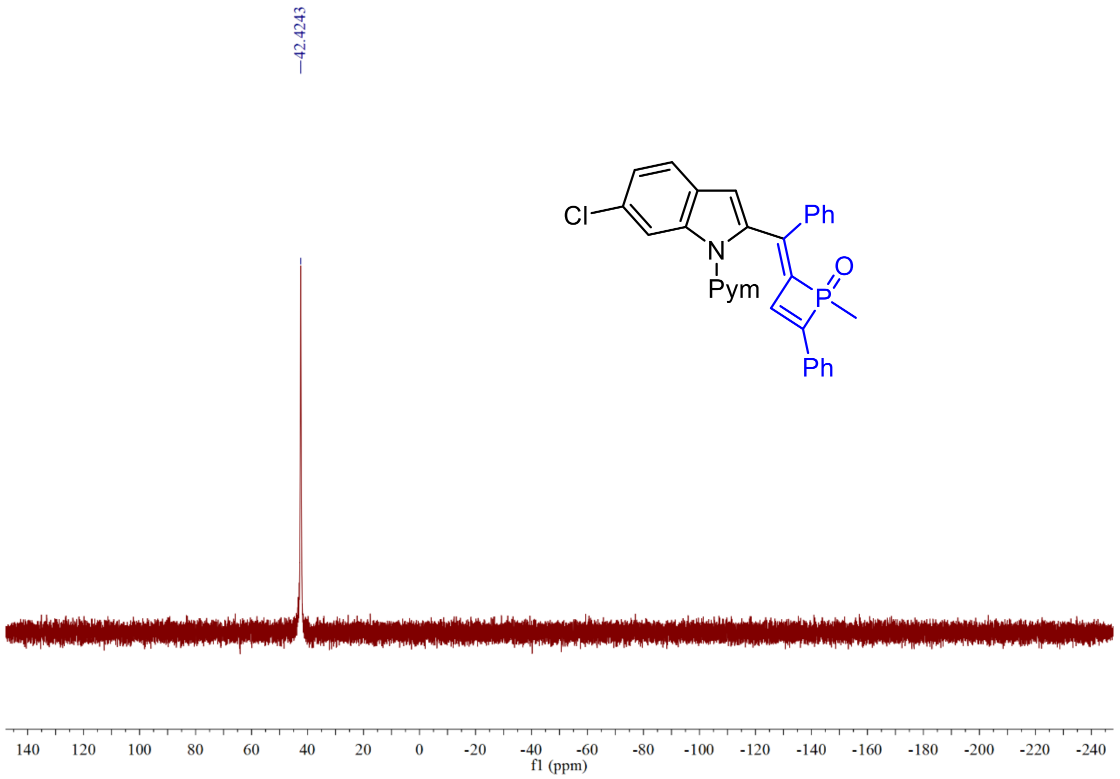

^{31}P NMR spectrum of compound **3u**

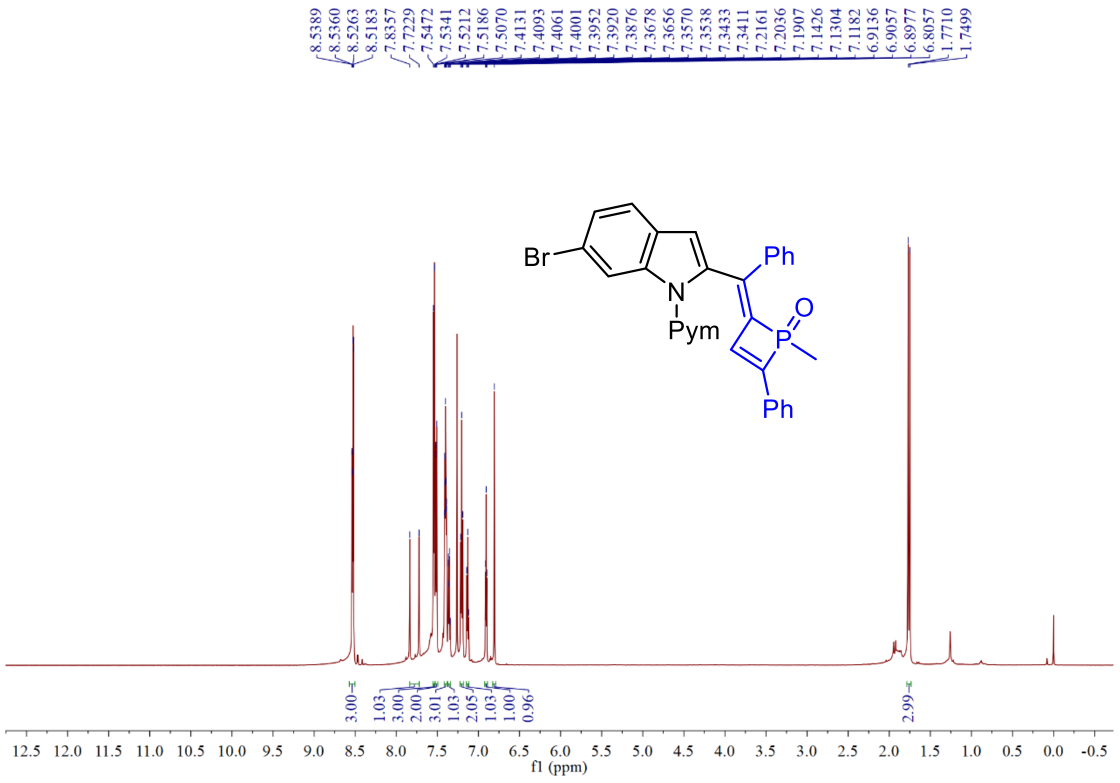

^{1}H NMR spectrum of compound **3v**

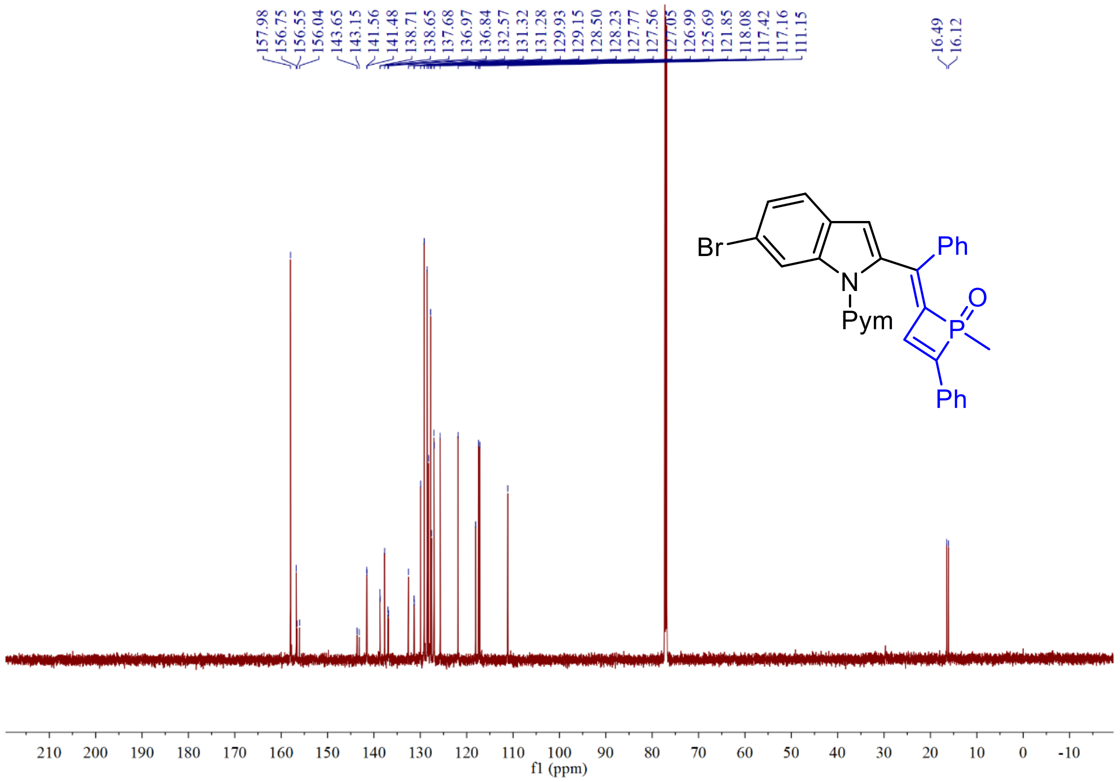

^{13}C NMR spectrum of compound **3v**

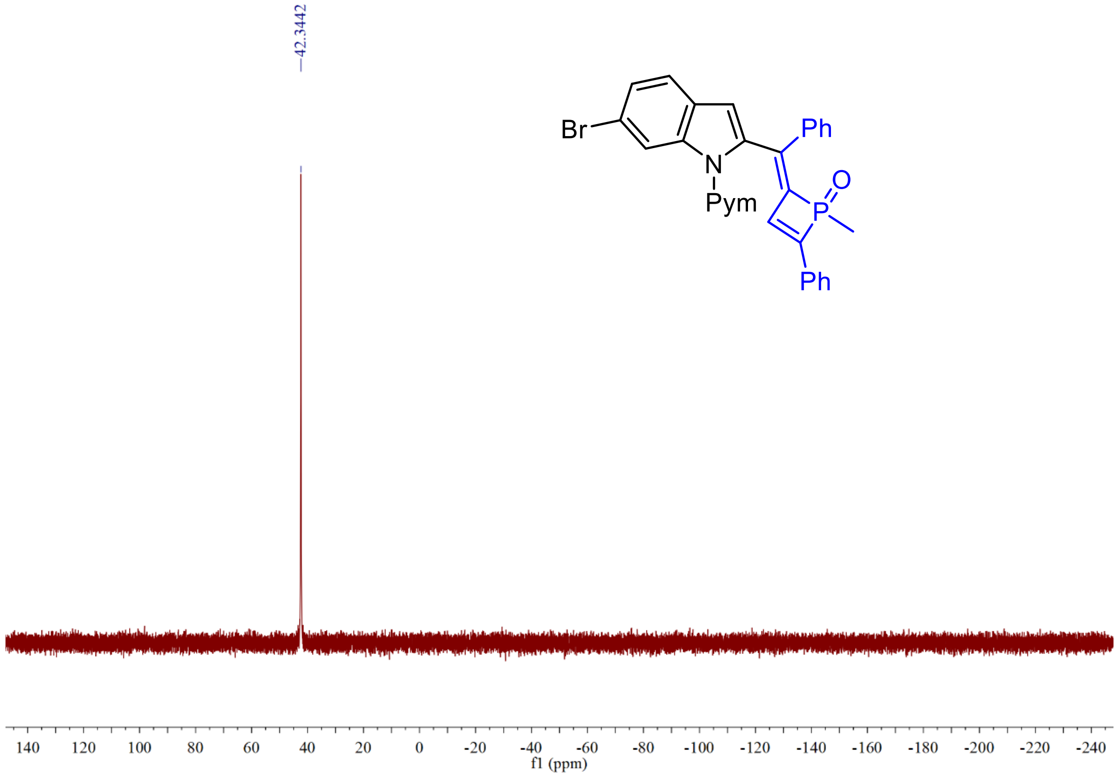

^{31}P NMR spectrum of compound **3v**

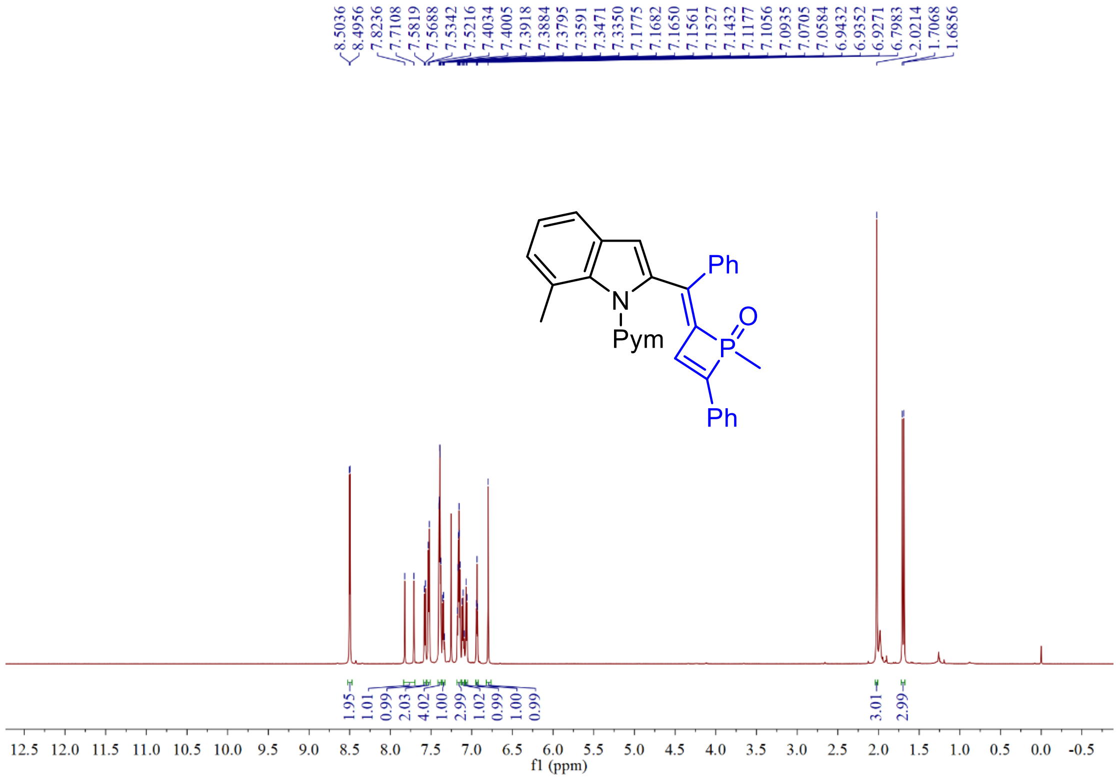

^{1}H NMR spectrum of compound **3w**

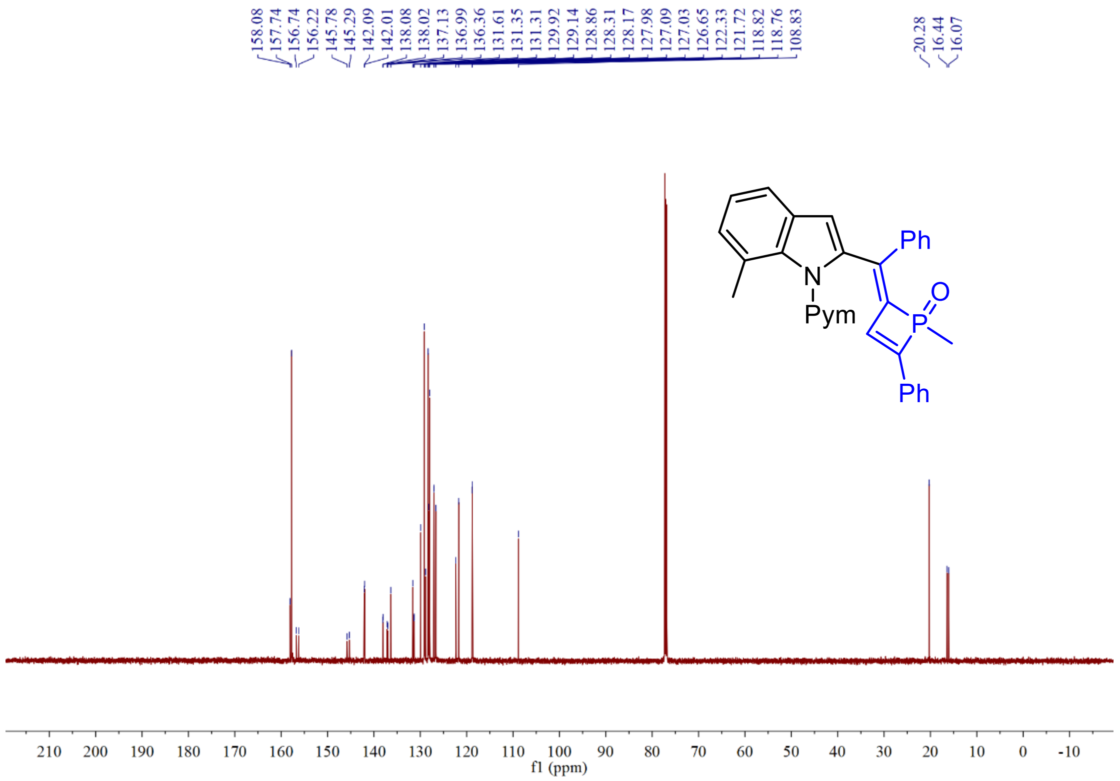

^{13}C NMR spectrum of compound **3w**

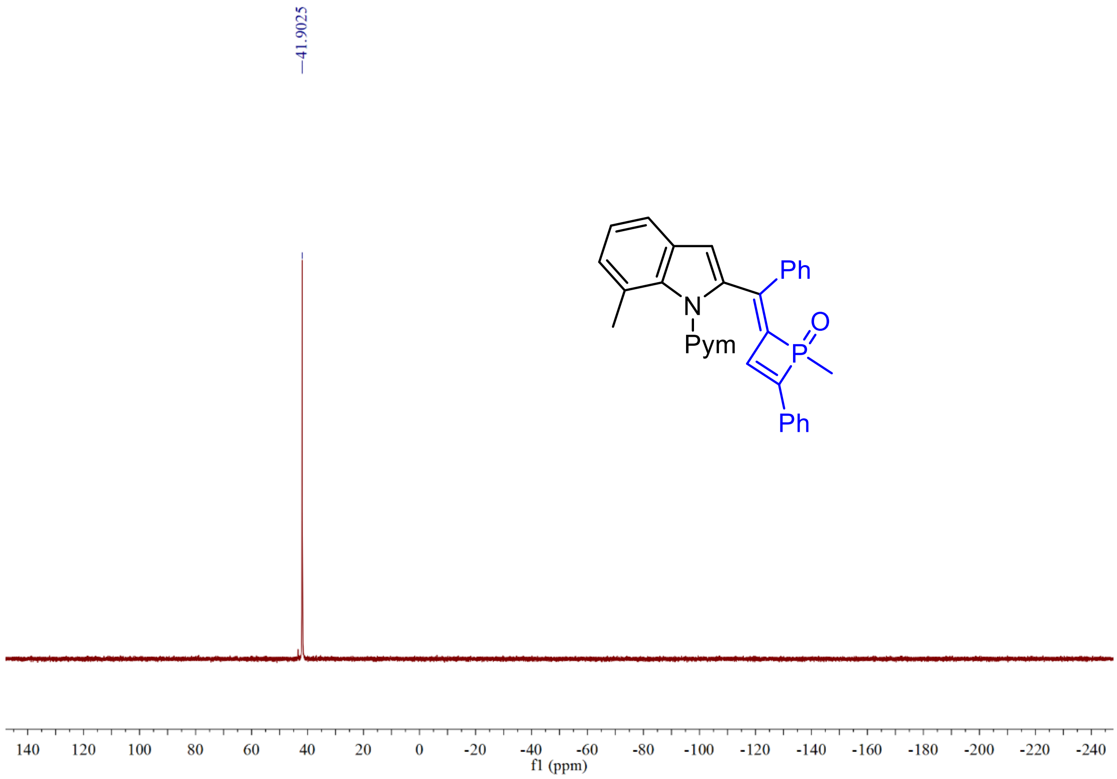

^{31}P NMR spectrum of compound **3w**

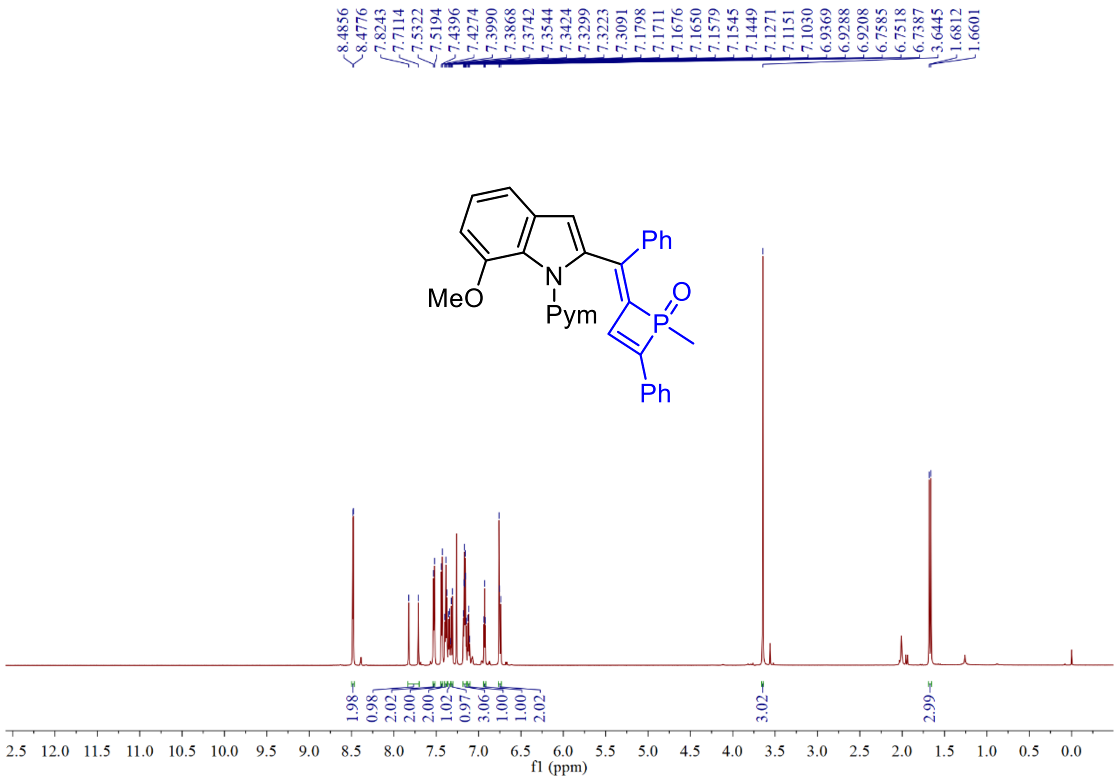

^{1}H NMR spectrum of compound **3x**

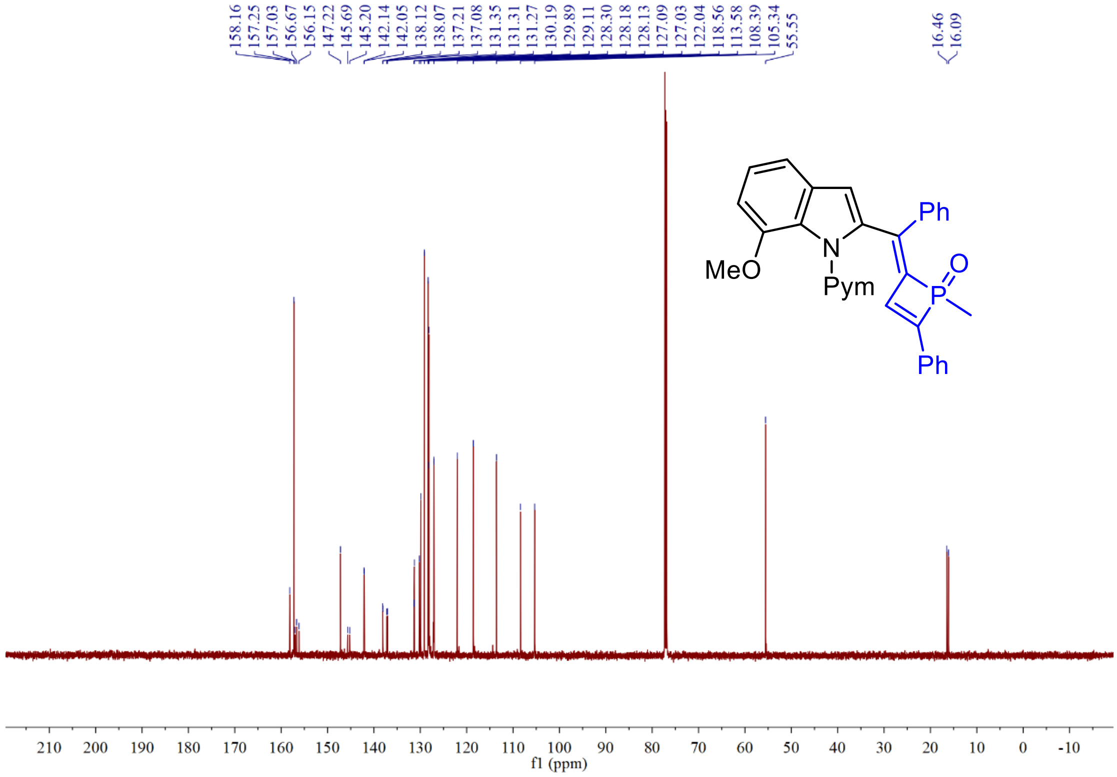

^{13}C NMR spectrum of compound **3x**

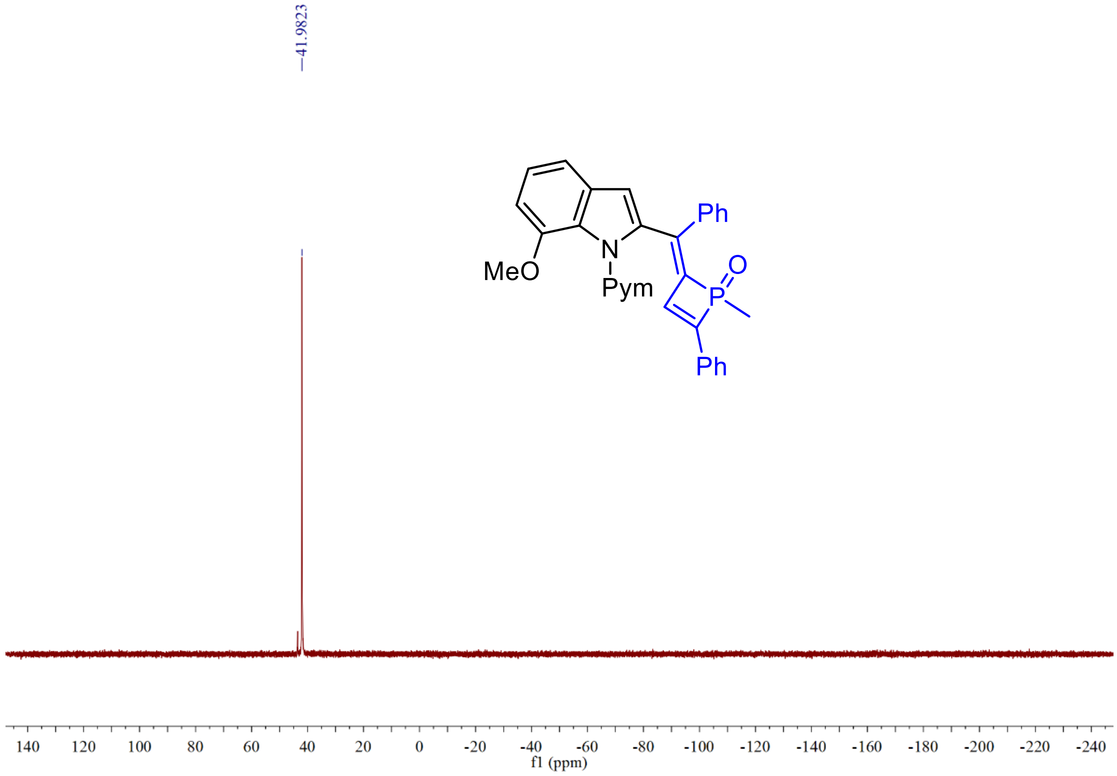

^{31}P NMR spectrum of compound **3x**

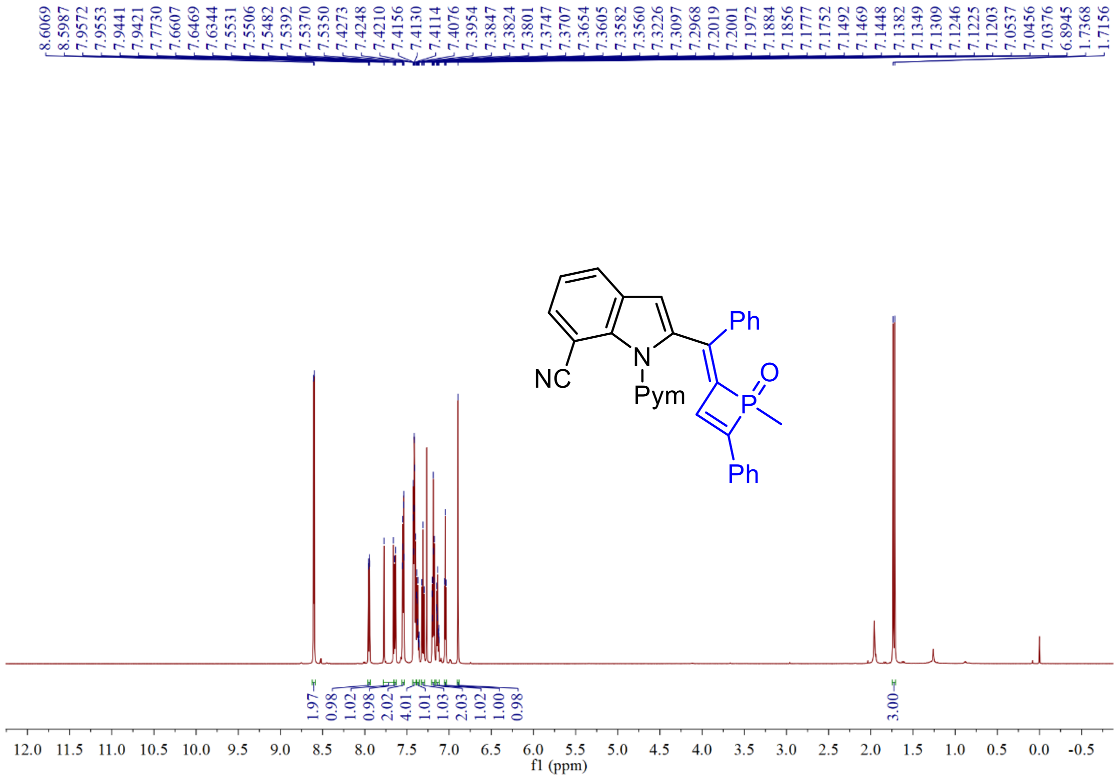

^{1}H NMR spectrum of compound **3y**

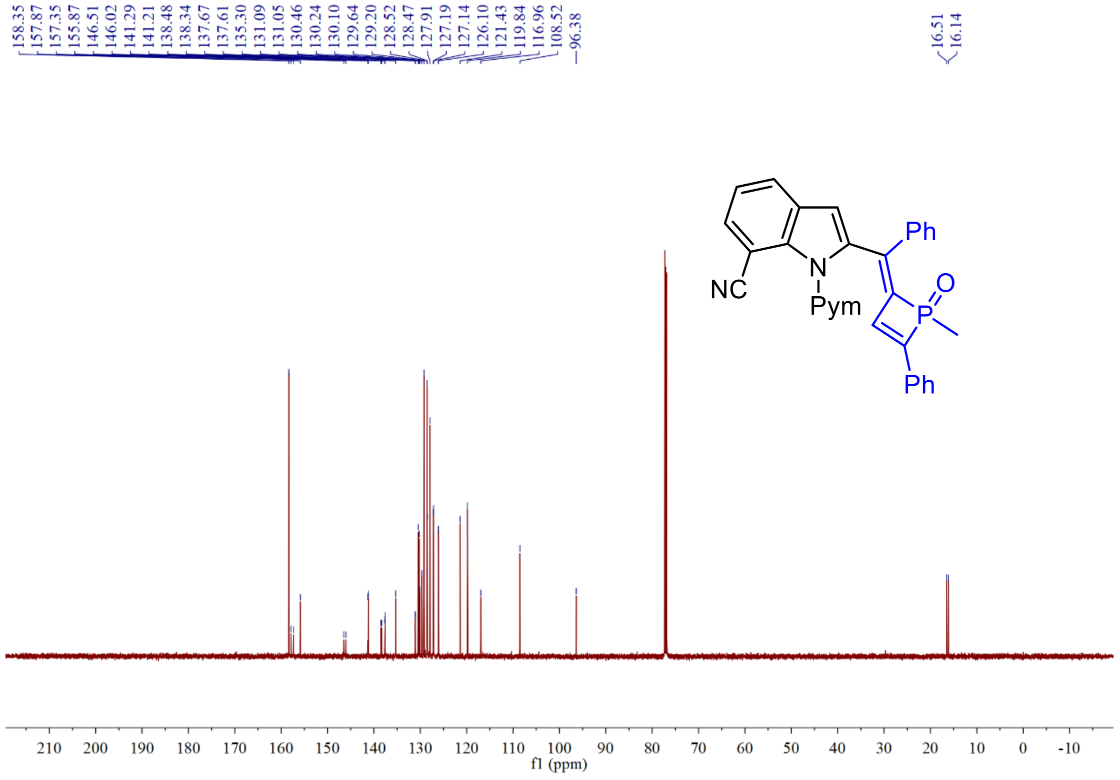

^{13}C NMR spectrum of compound **3y**

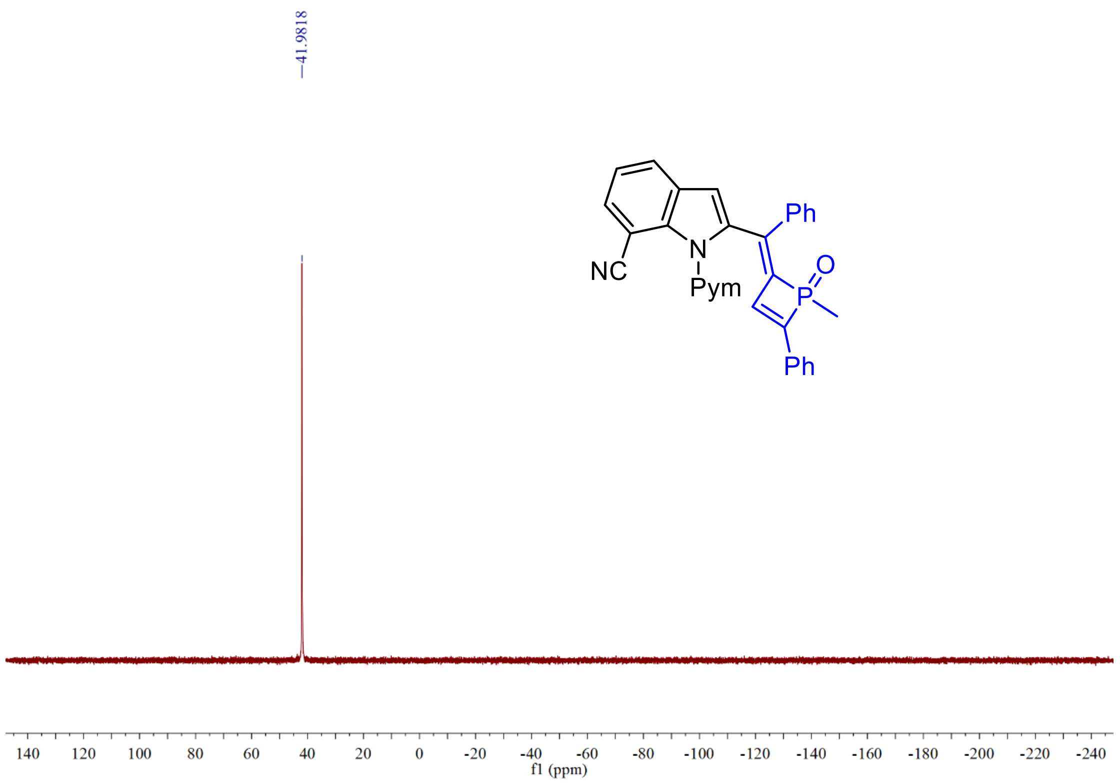

^{31}P NMR spectrum of compound **3y**

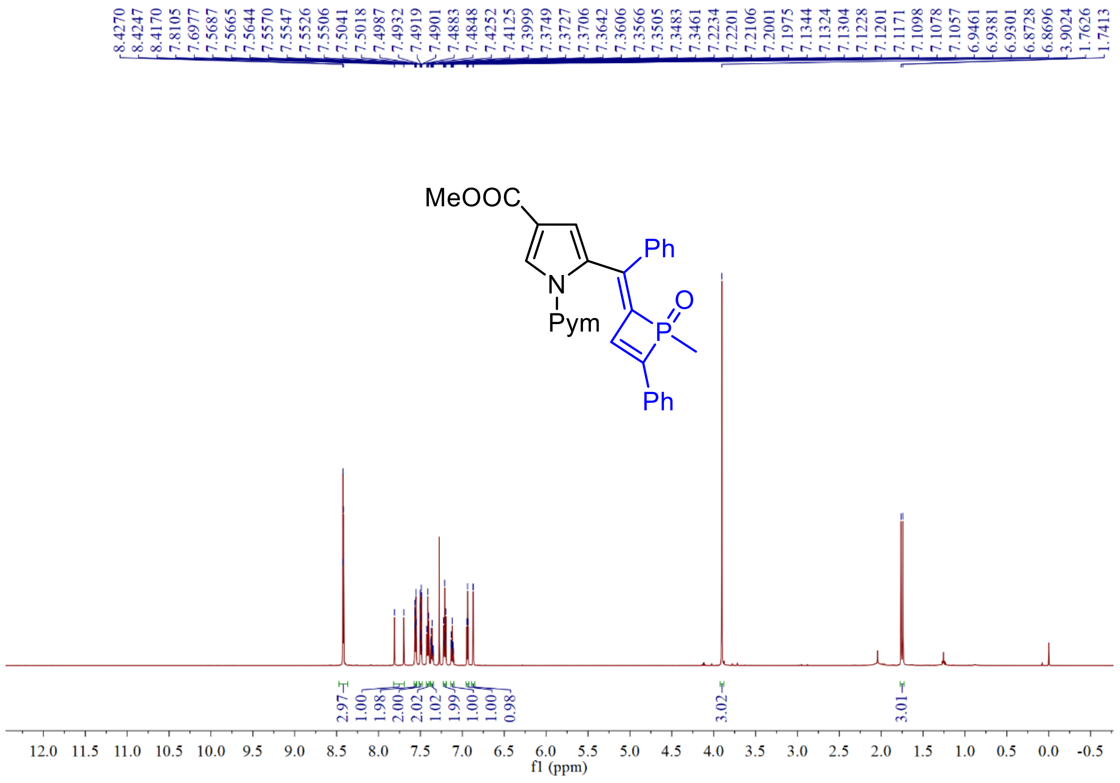

^{1}H NMR spectrum of compound **3z**

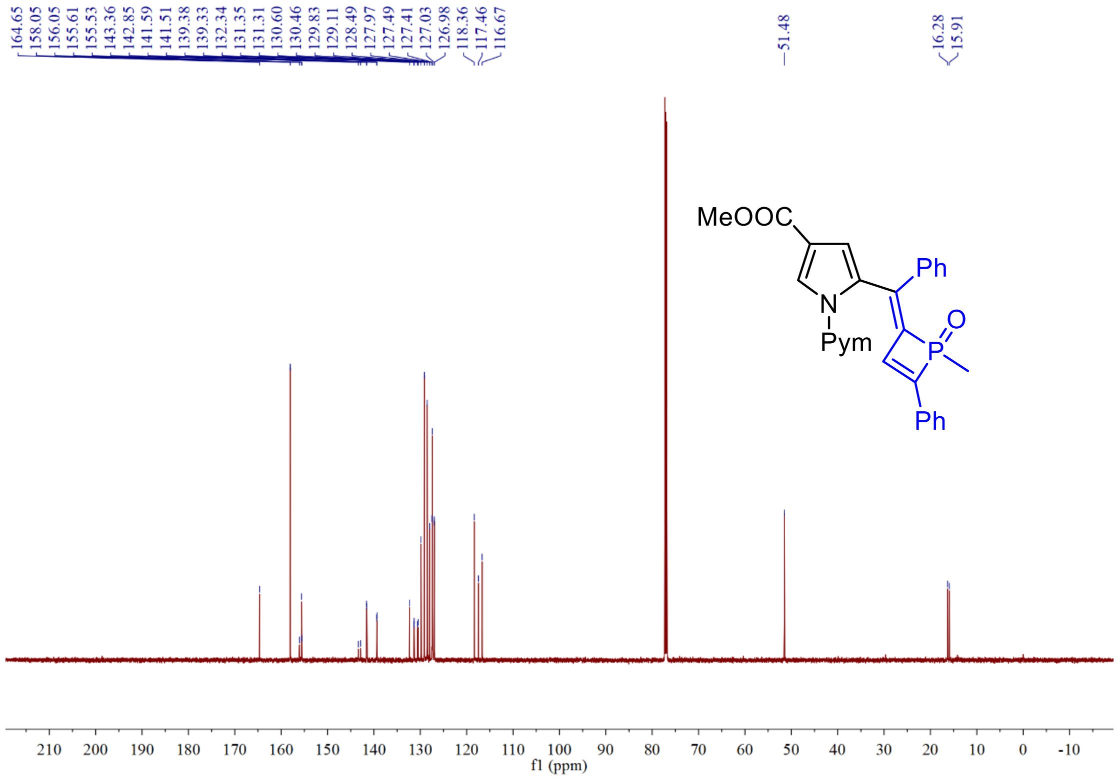

^{13}C NMR spectrum of compound **3z**

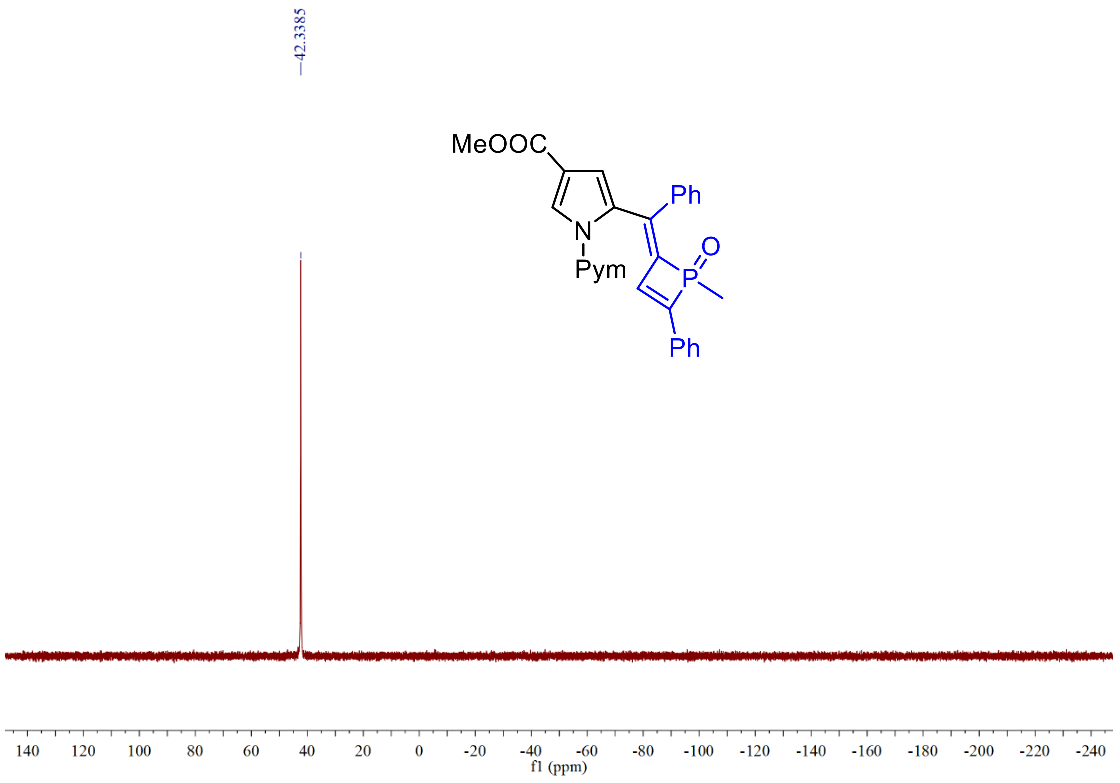

^{31}P NMR spectrum of compound **3z**

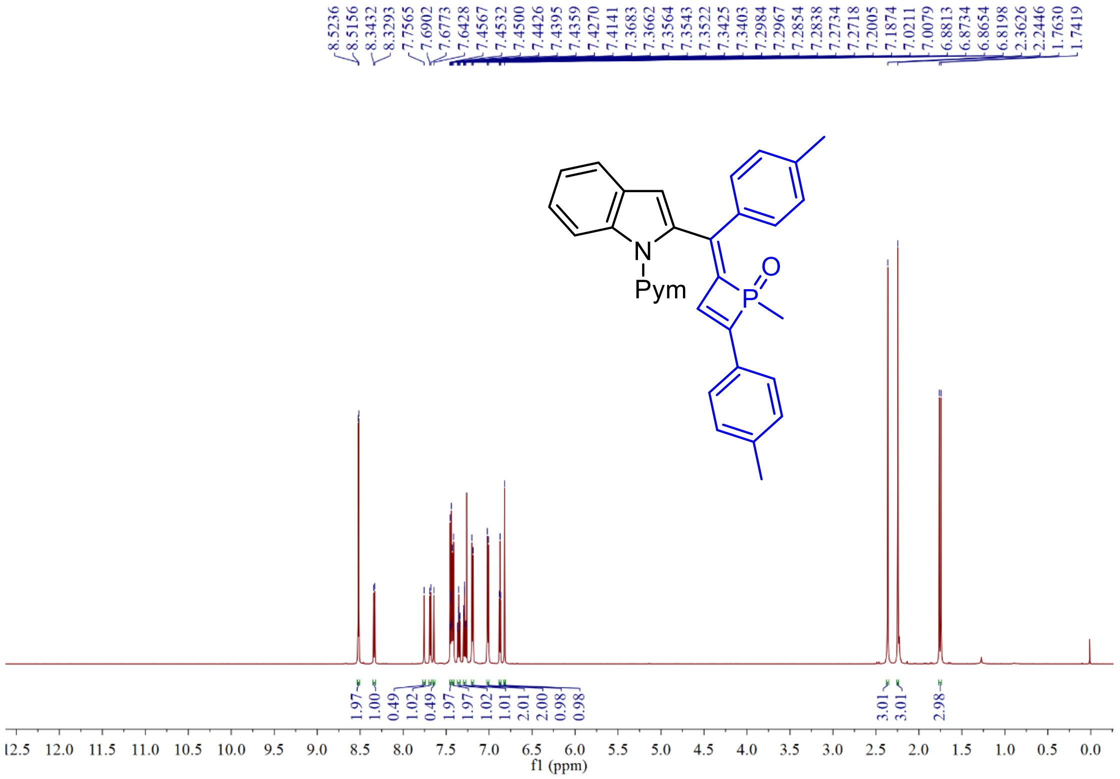

^{1}H NMR spectrum of compound **4a**

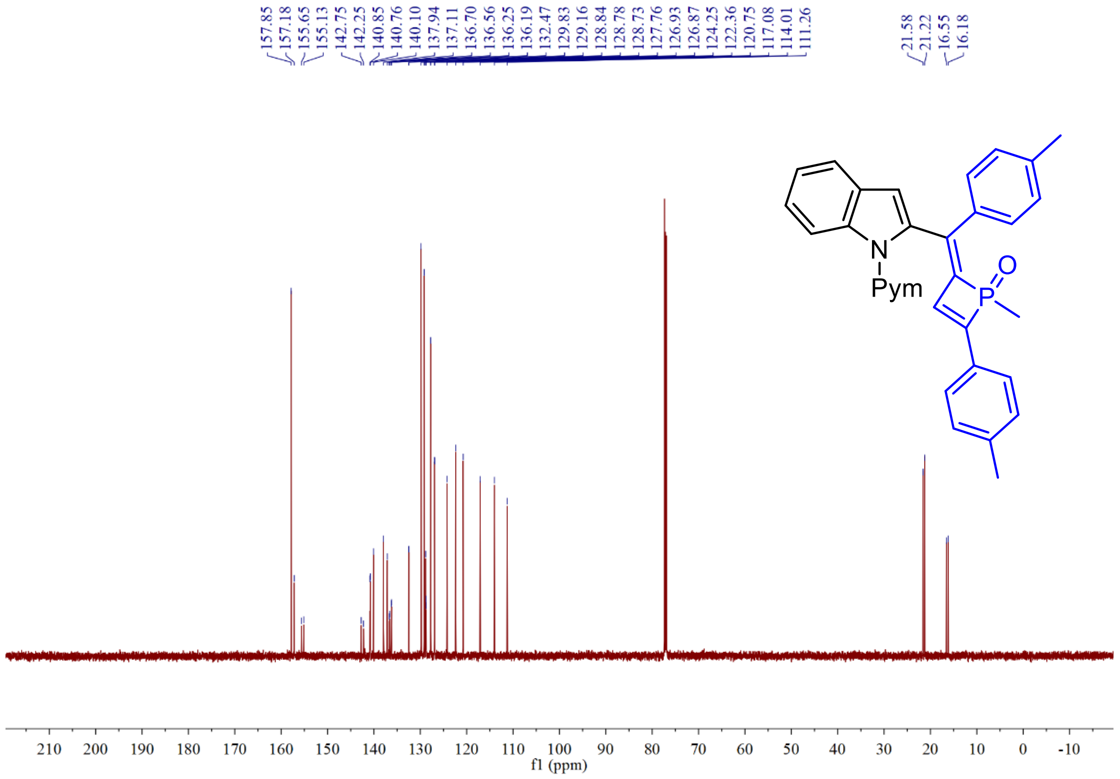

^{13}C NMR spectrum of compound **4a**

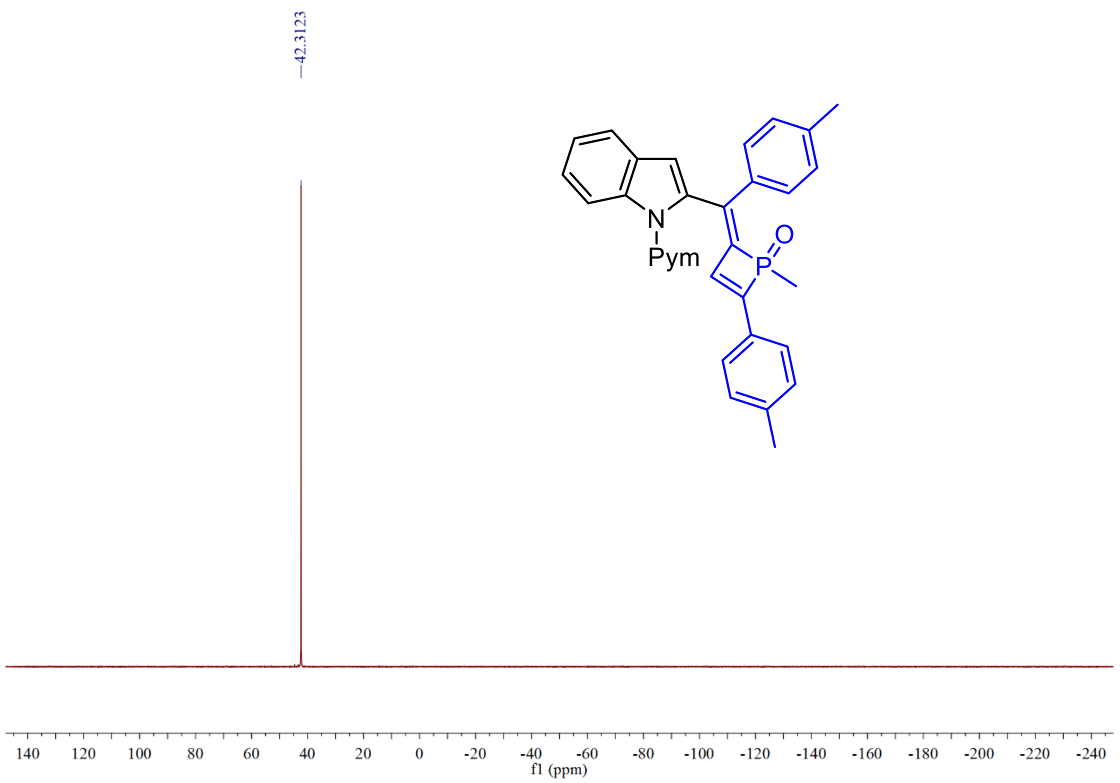

^{31}P NMR spectrum of compound **4a**

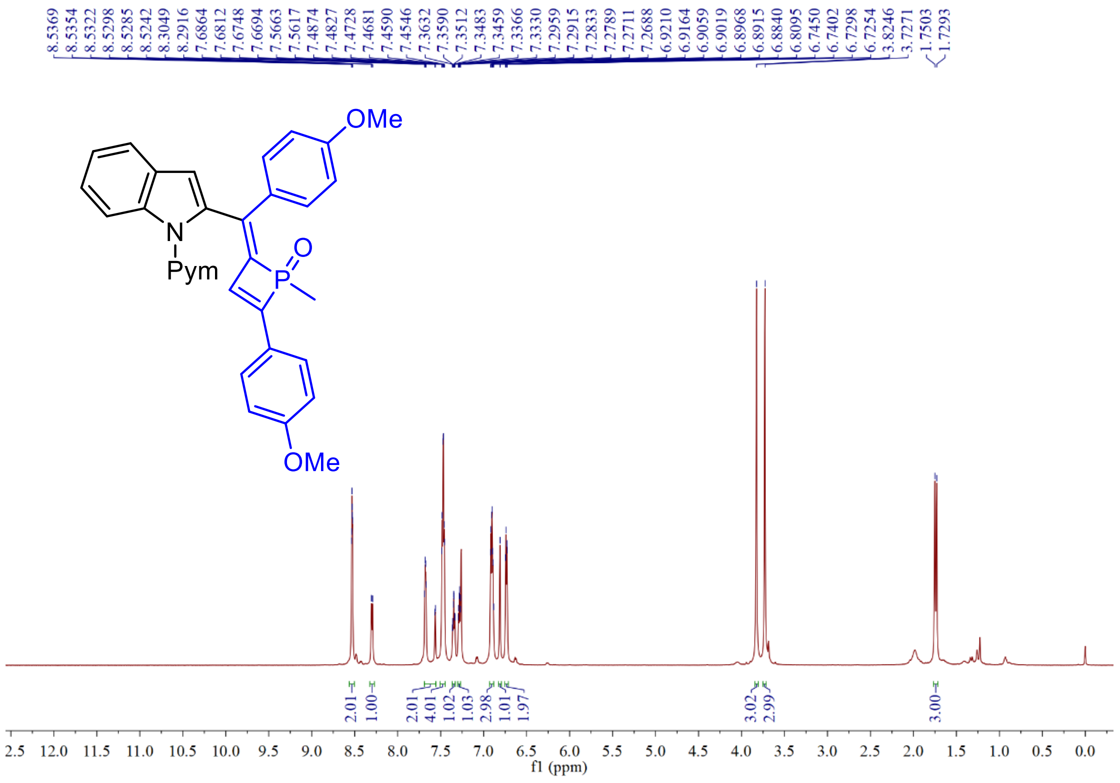

^{1}H NMR spectrum of compound **4b**

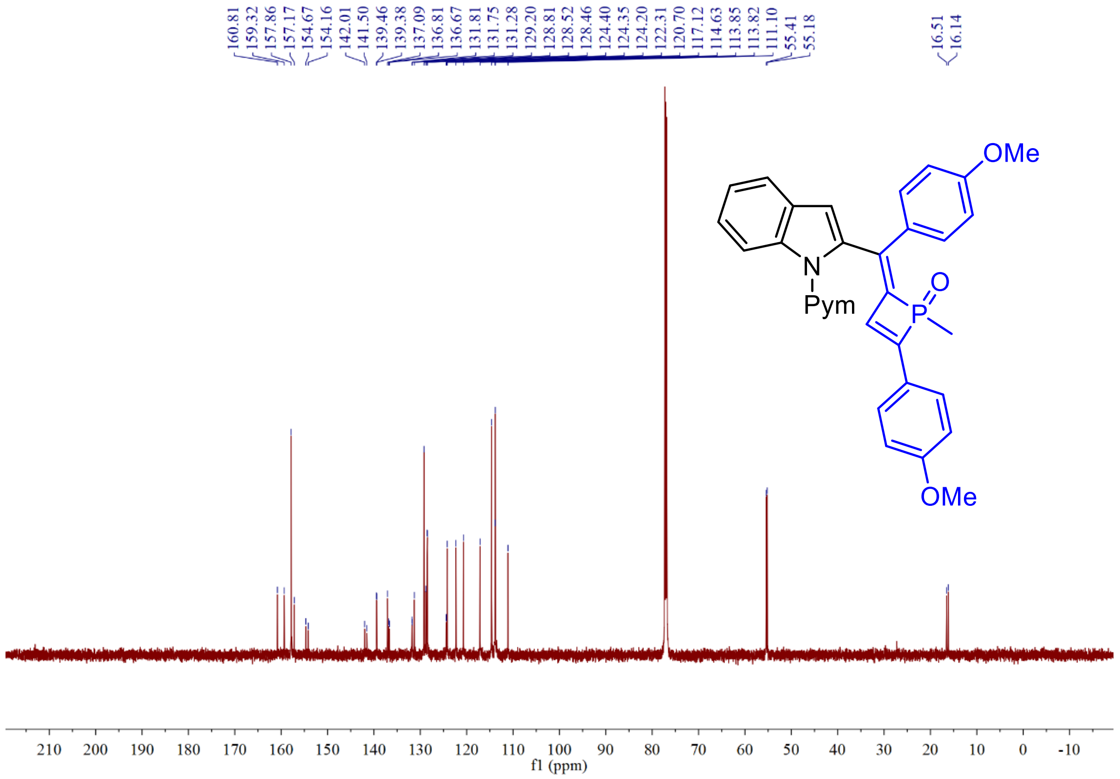

^{13}C NMR spectrum of compound **4b**

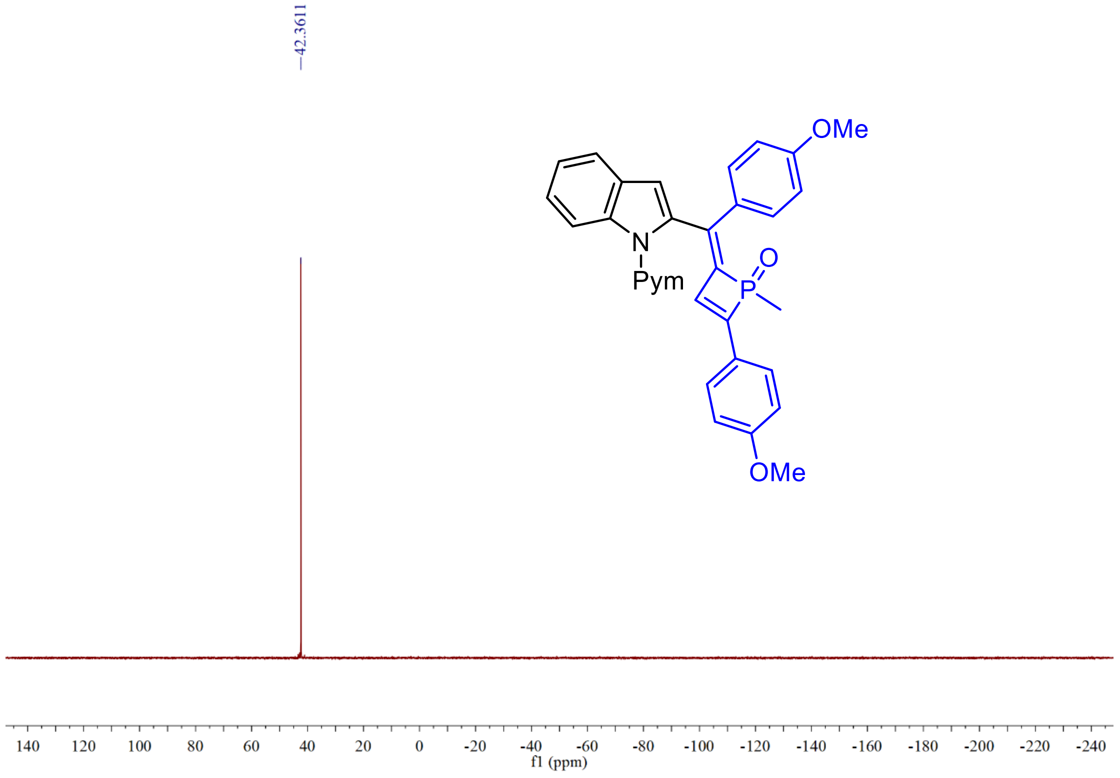

^{31}P NMR spectrum of compound **4b**

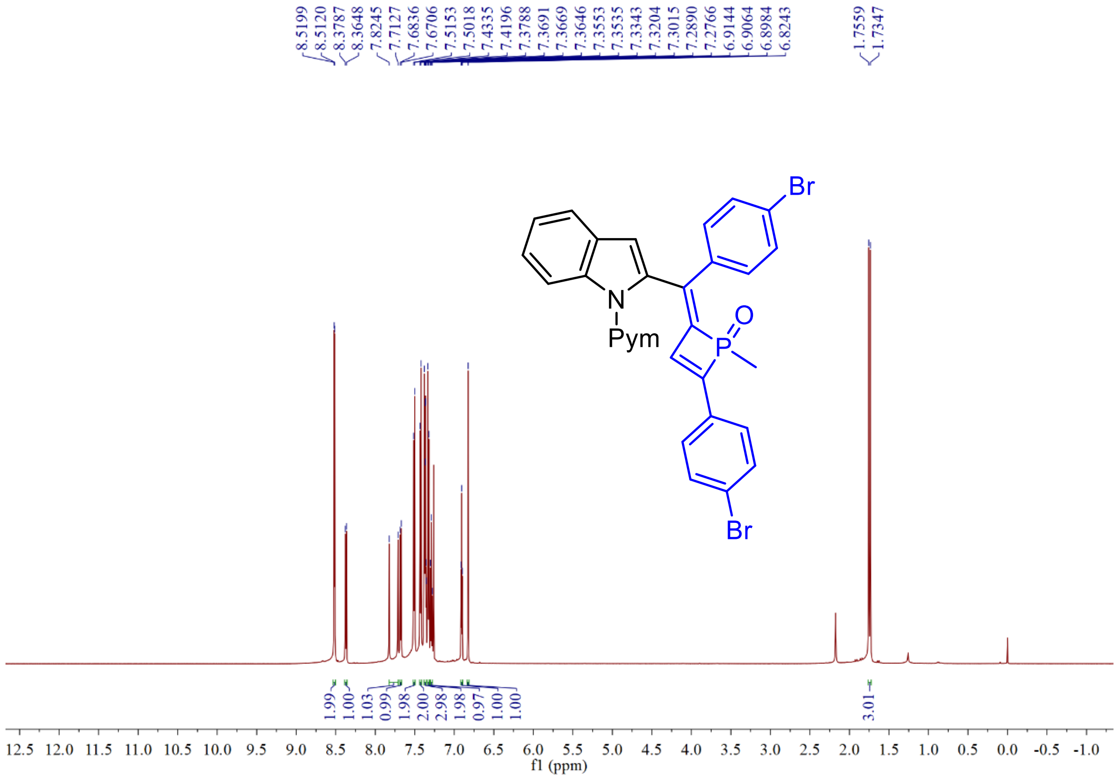

^{1}H NMR spectrum of compound **4c**

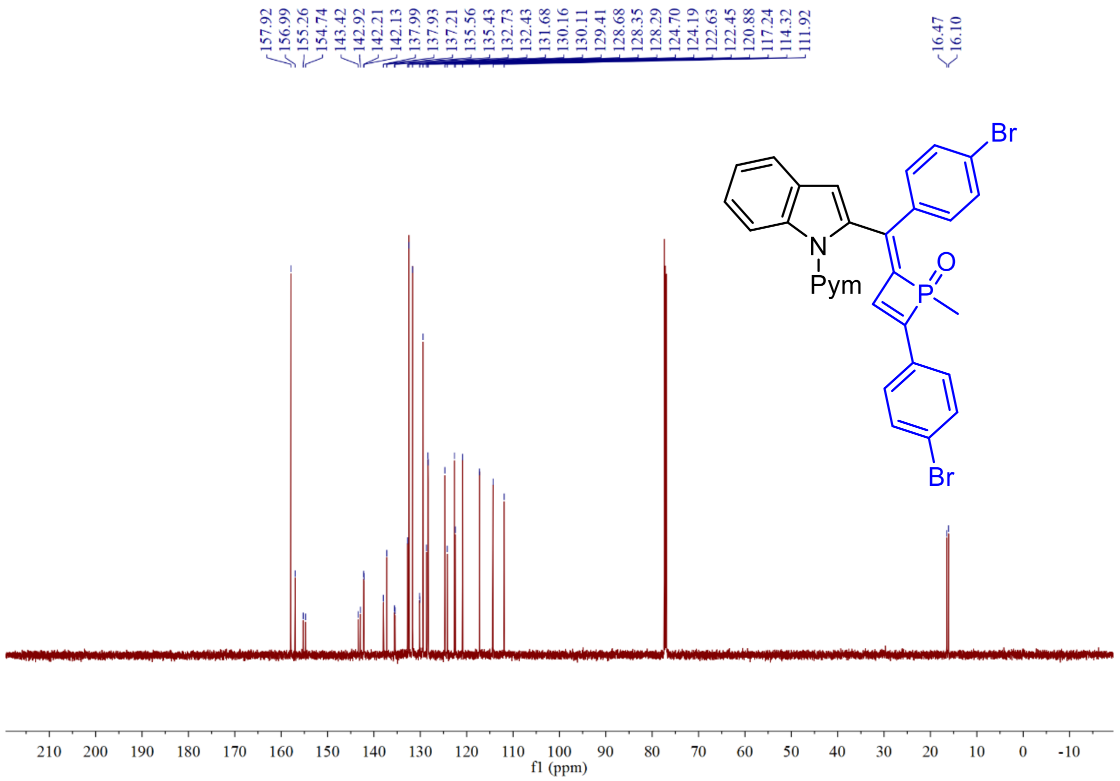

^{13}C NMR spectrum of compound **4c**

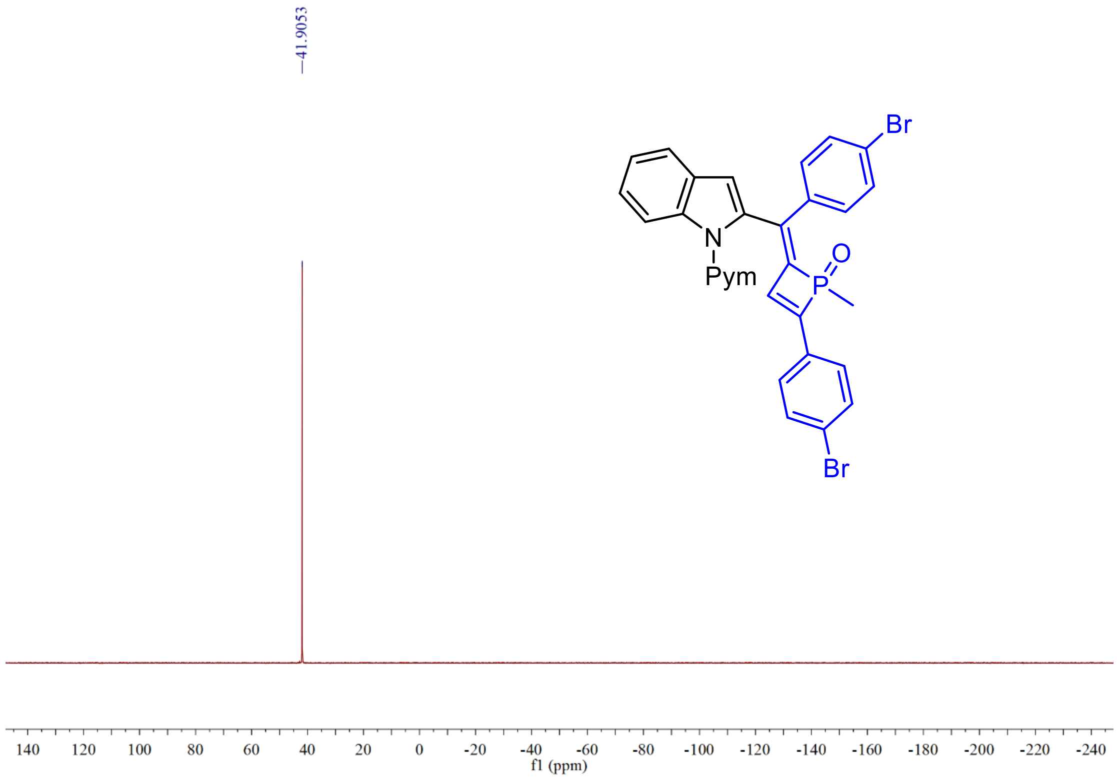

^{31}P NMR spectrum of compound **4c**

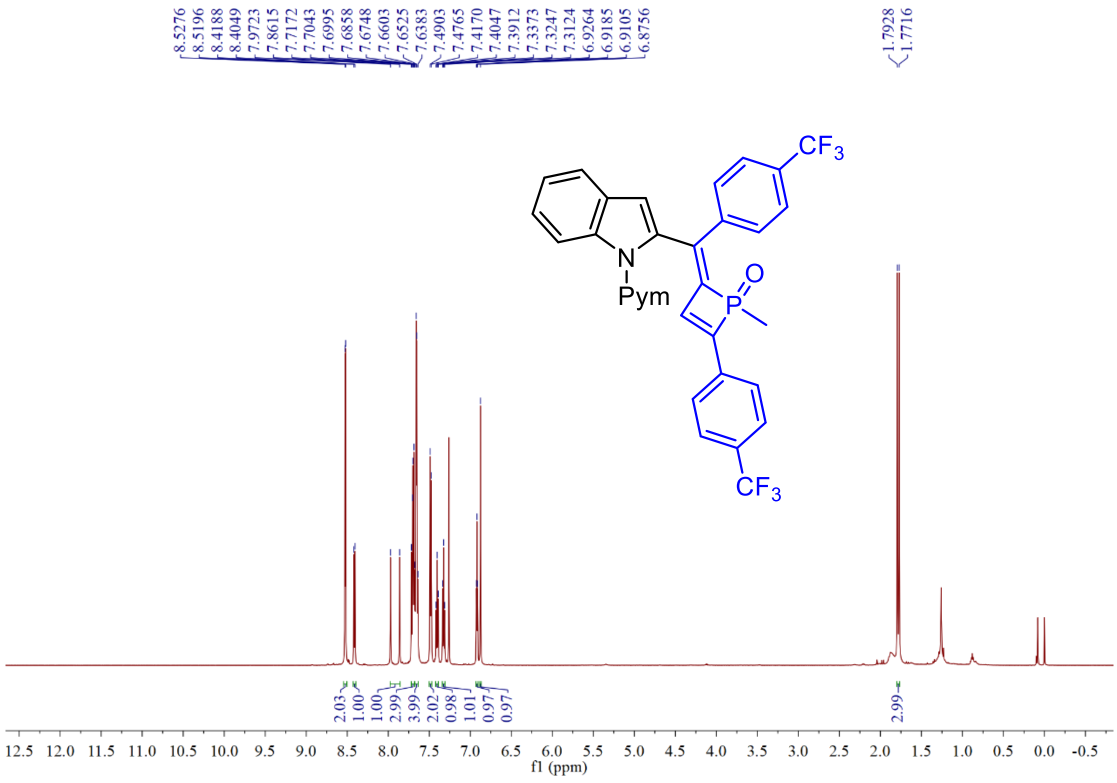

^{1}H NMR spectrum of compound **4d**

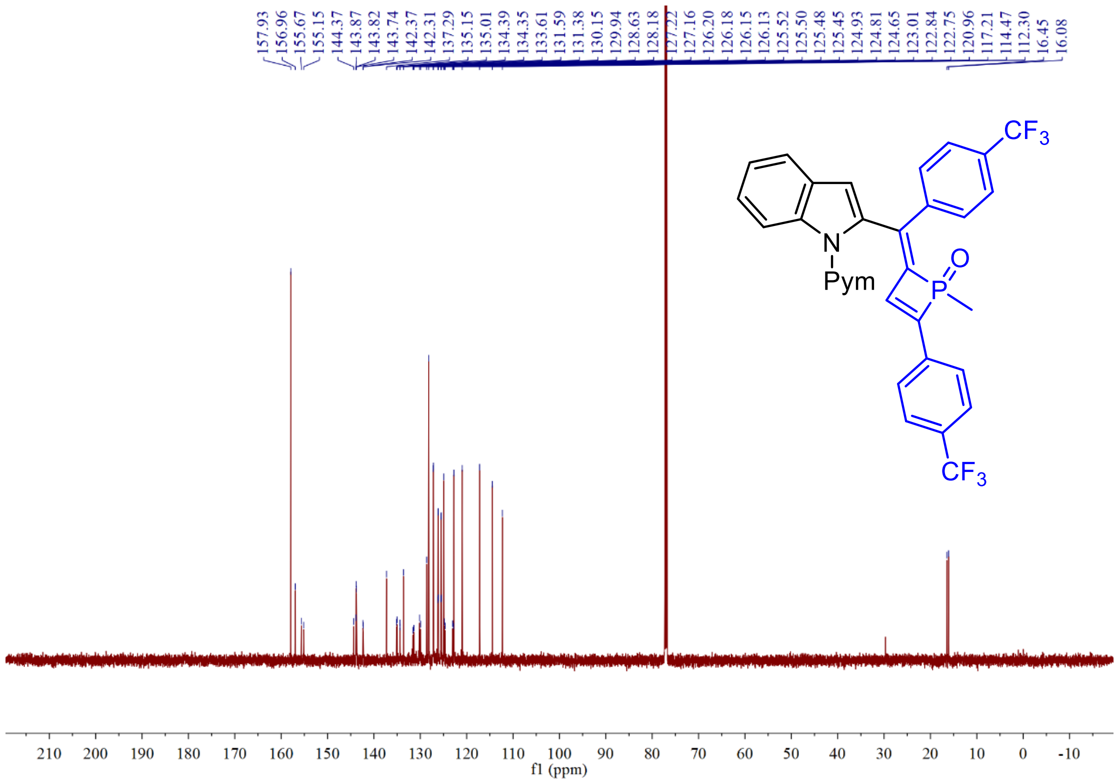

^{13}C NMR spectrum of compound **4d**

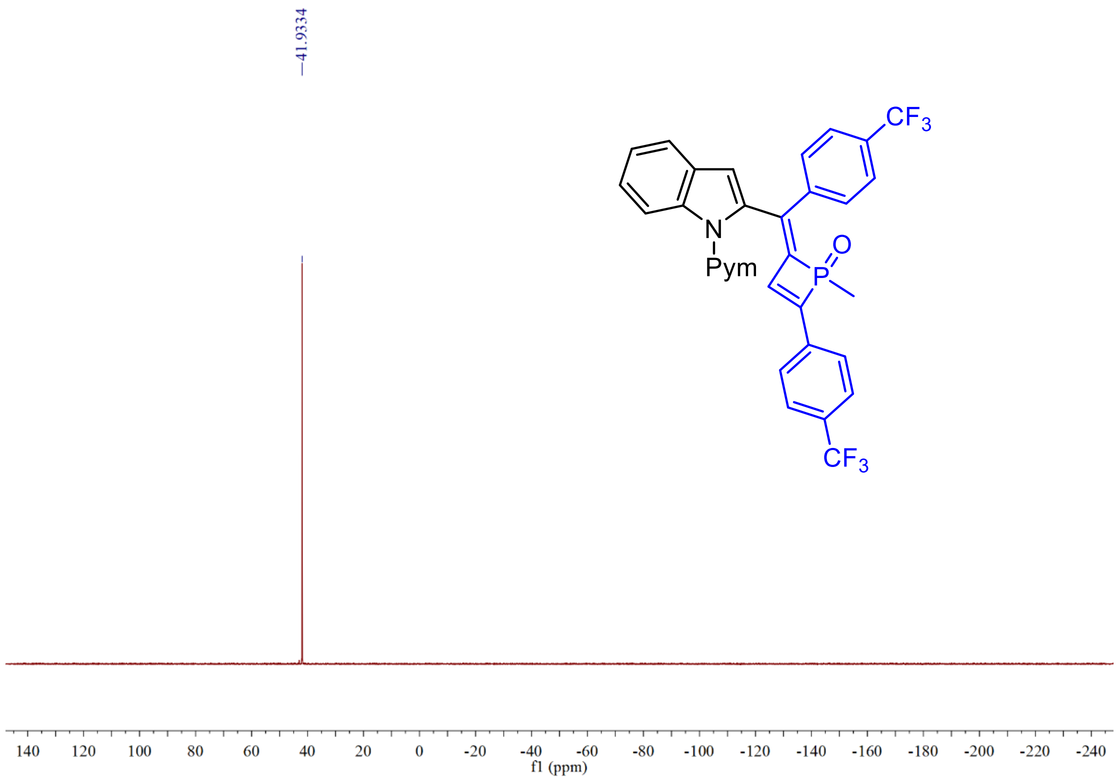

^{31}P NMR spectrum of compound **4d**

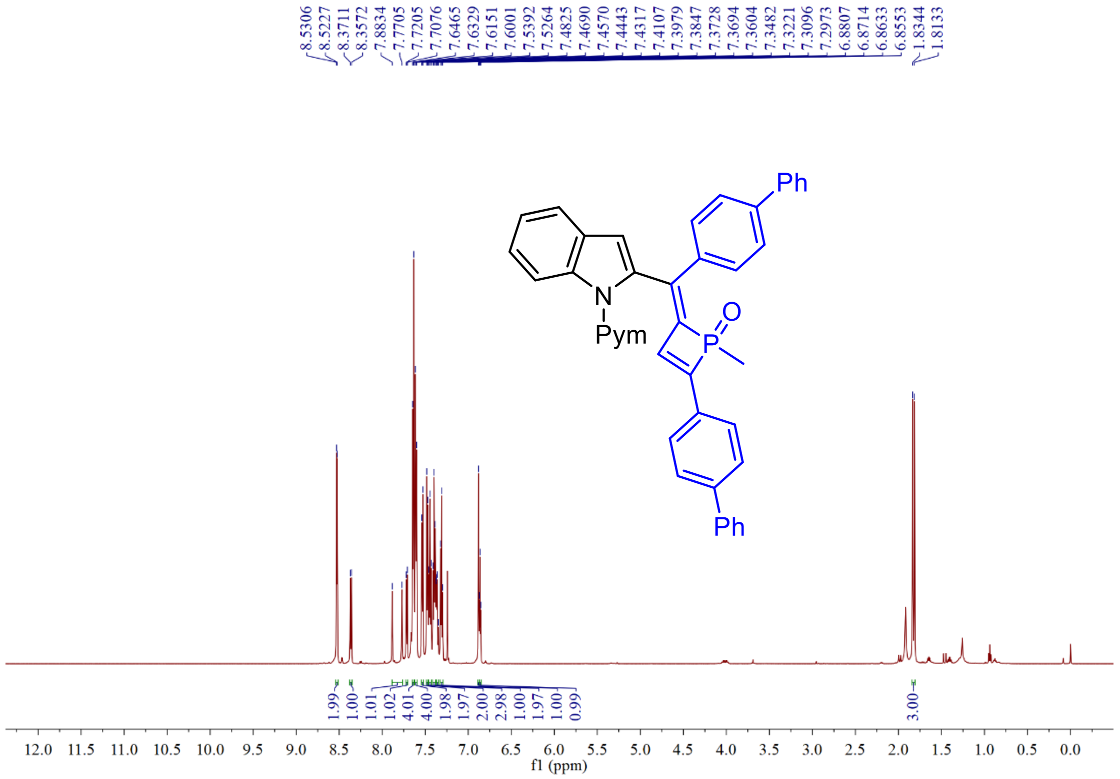

^{1}H NMR spectrum of compound **4e**

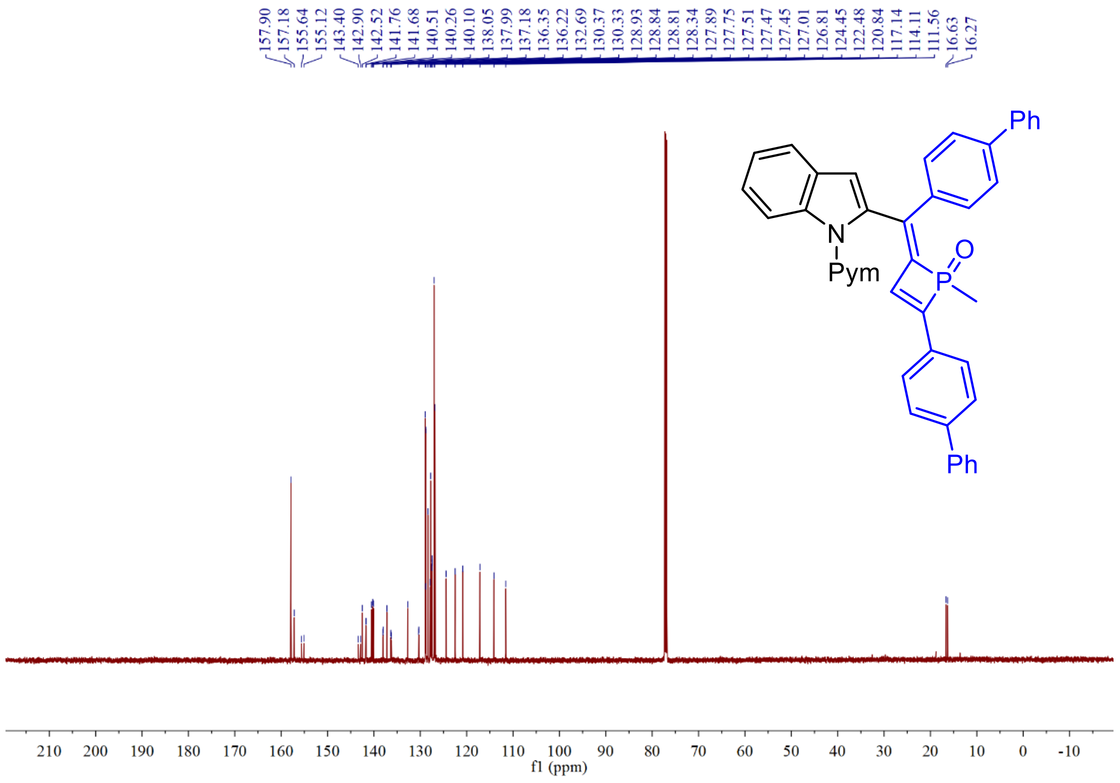

^{13}C NMR spectrum of compound **4e**

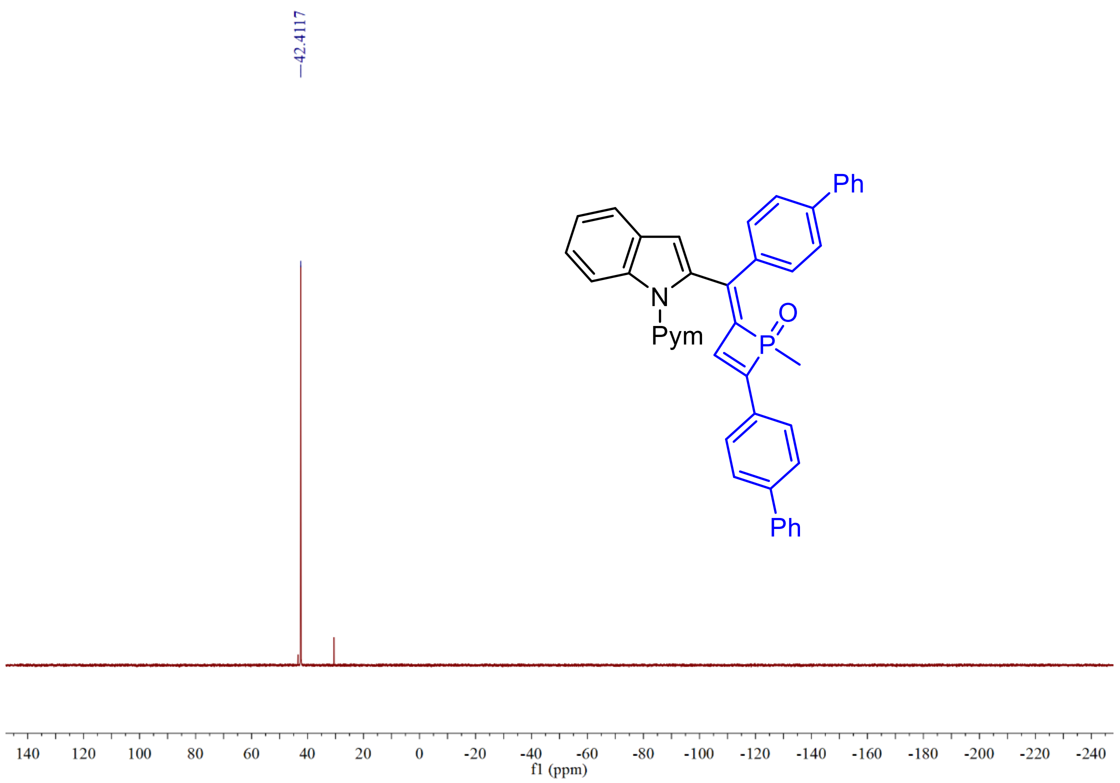

^{31}P NMR spectrum of compound **4e**

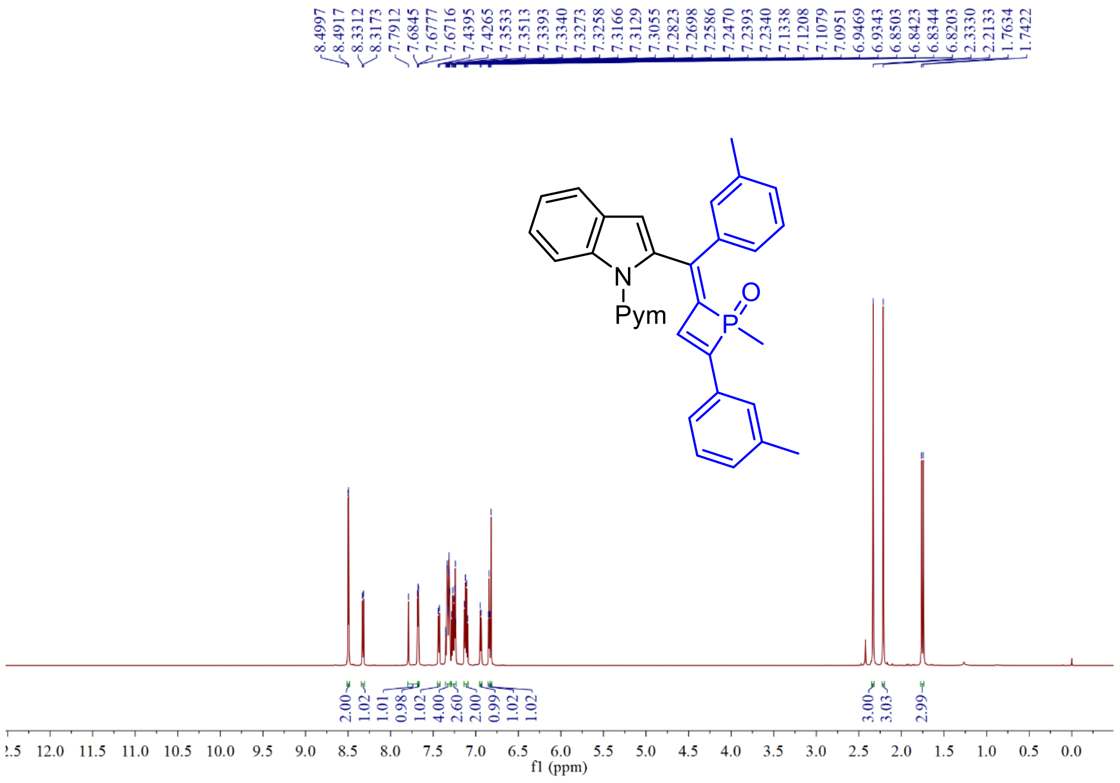

^{1}H NMR spectrum of compound **4f**

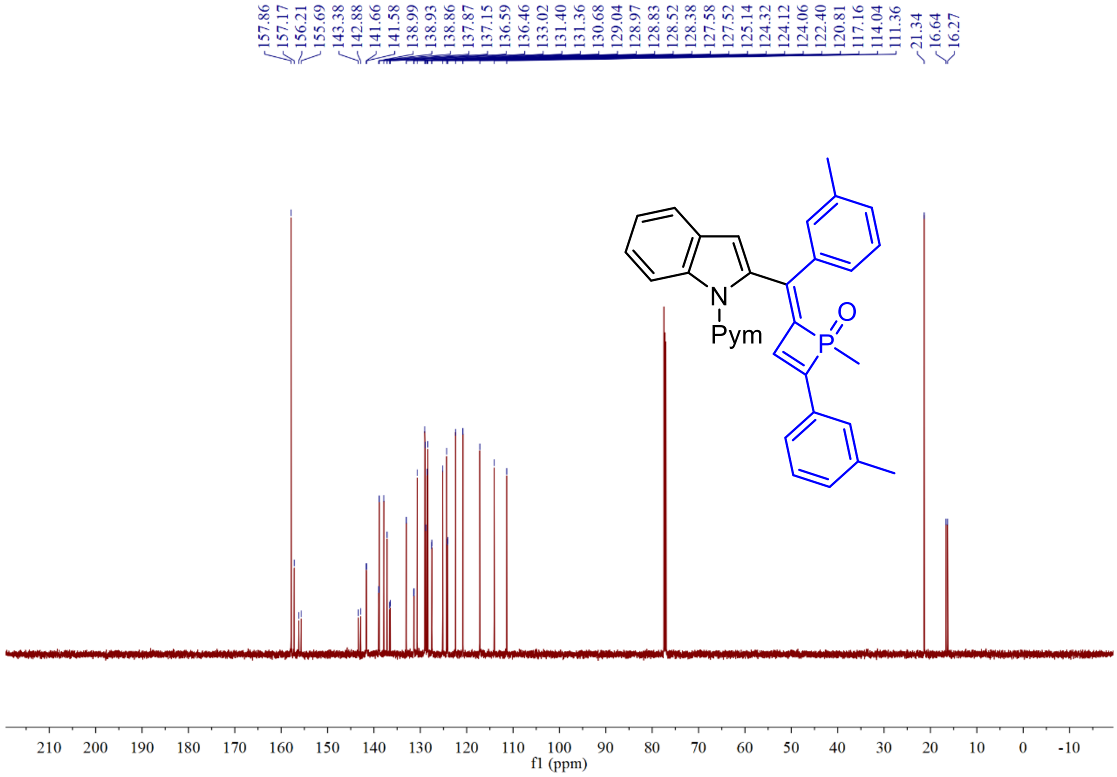

^{13}C NMR spectrum of compound **4f**

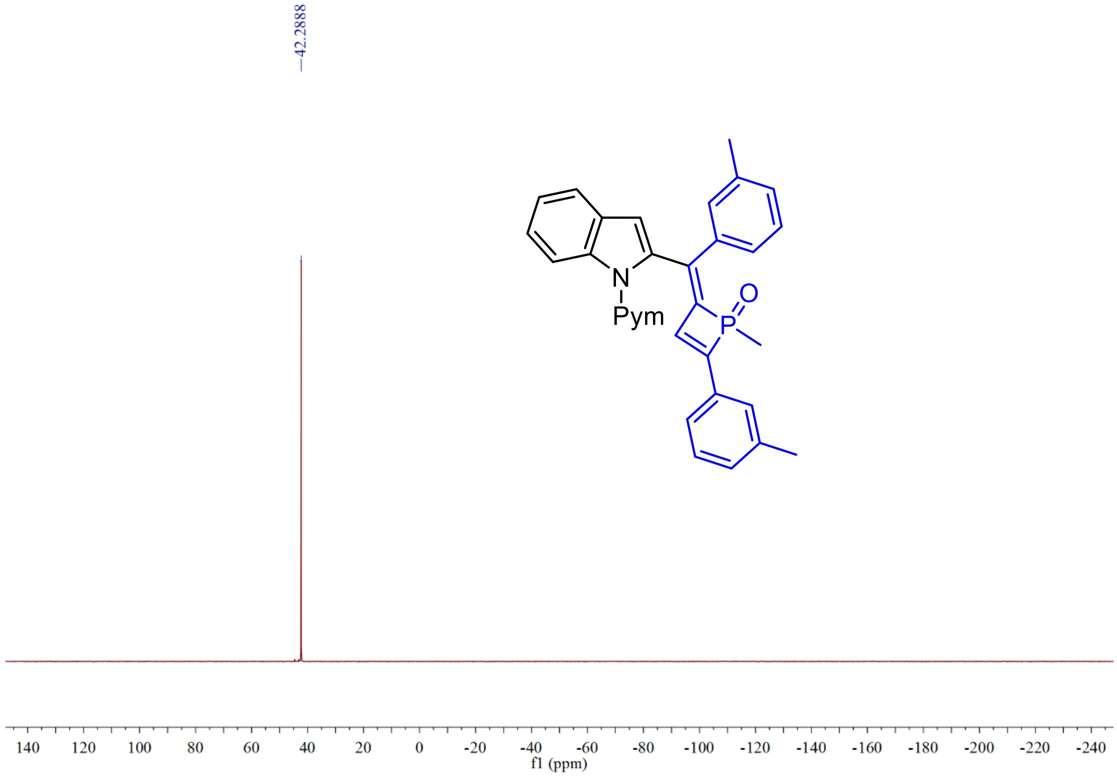

^{31}P NMR spectrum of compound **4f**

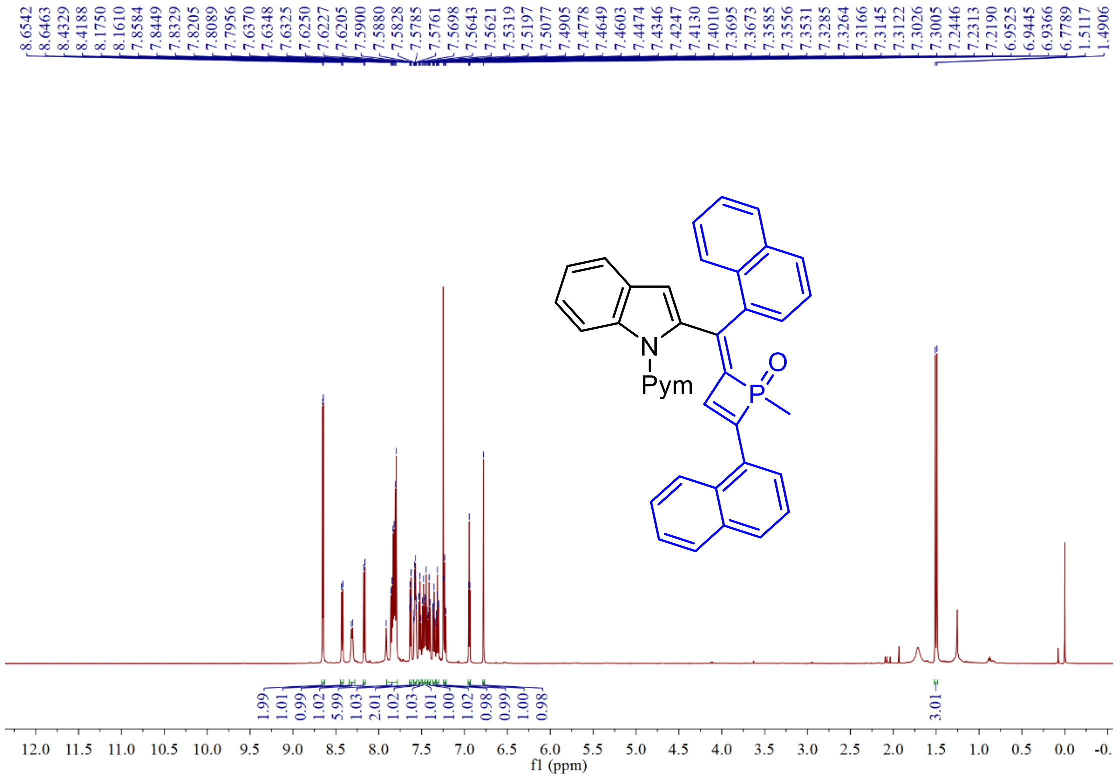

^{1}H NMR spectrum of compound **4g**

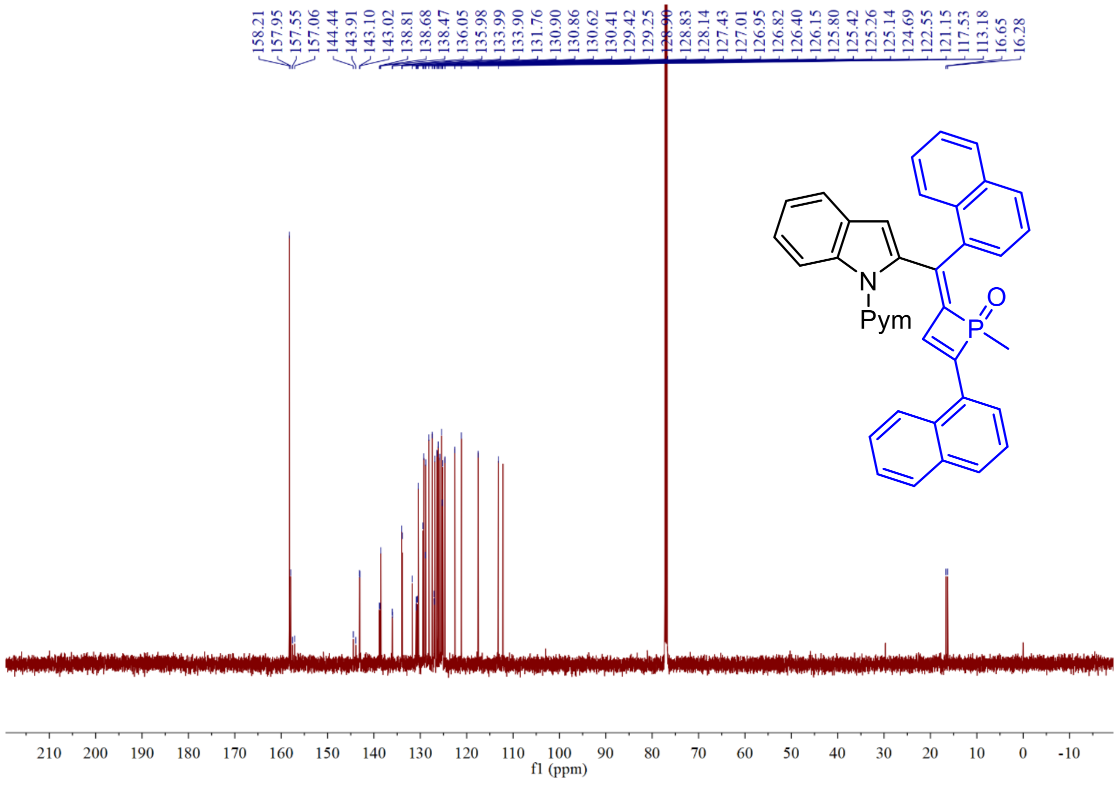

^{13}C NMR spectrum of compound **4g**

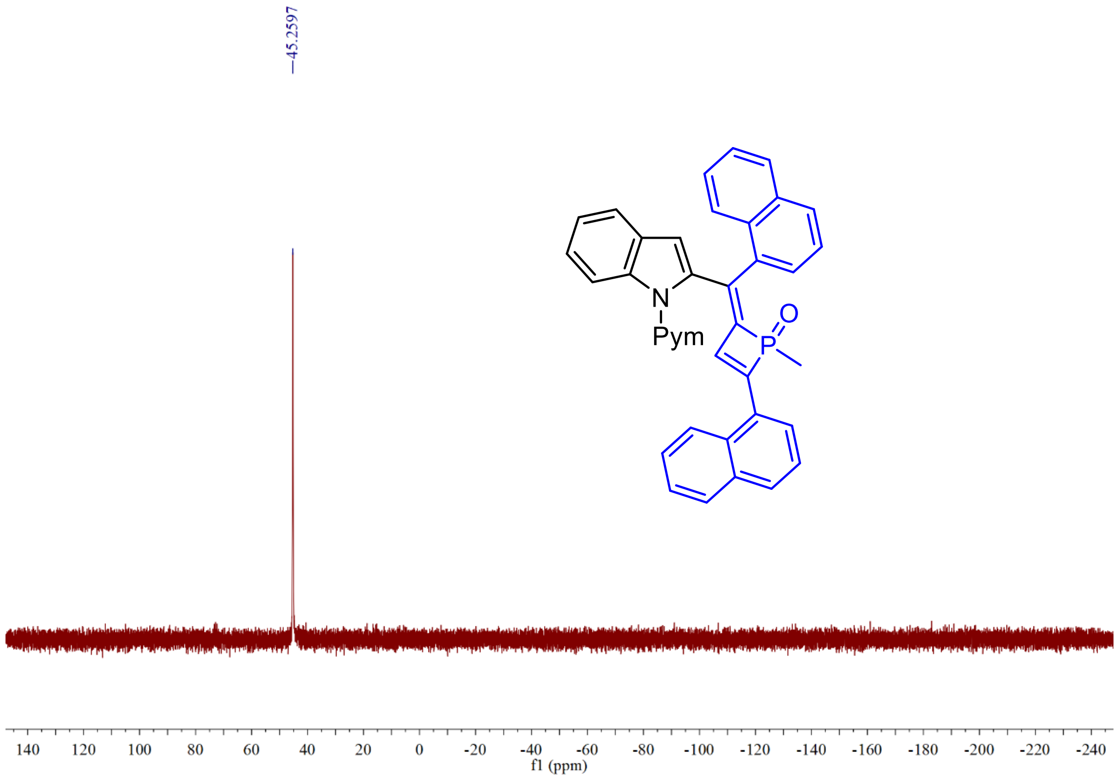

^{31}P NMR spectrum of compound **4g**

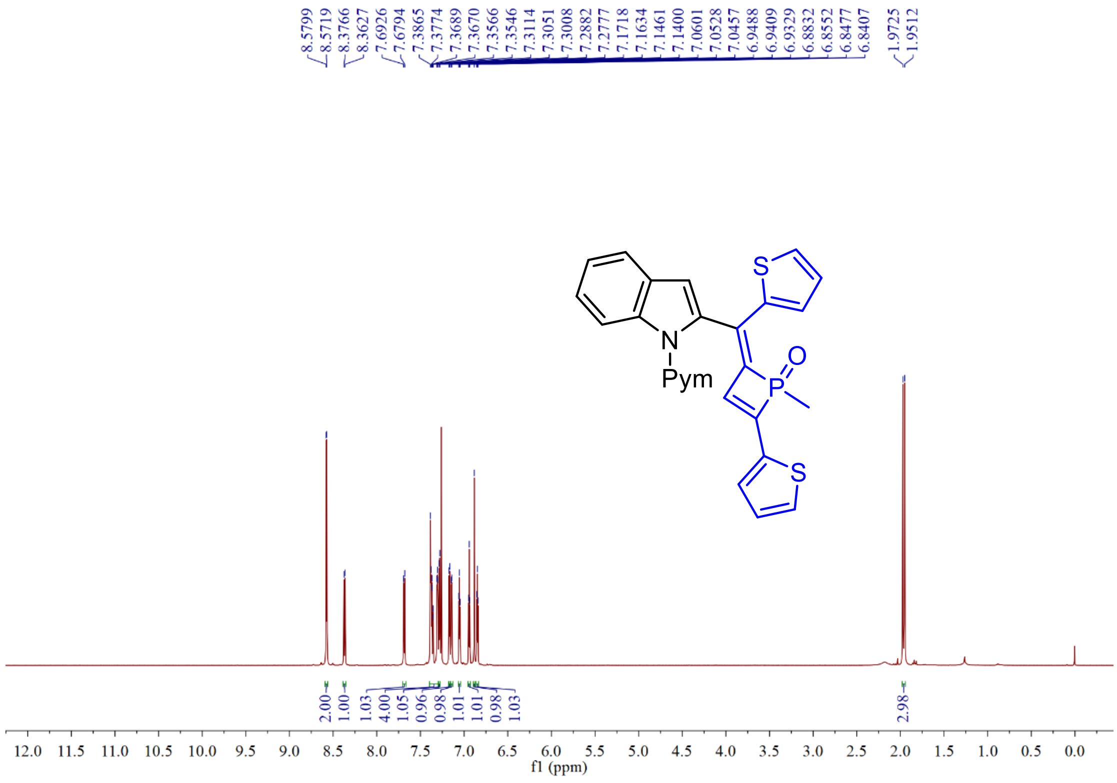

^{1}H NMR spectrum of compound **4h**

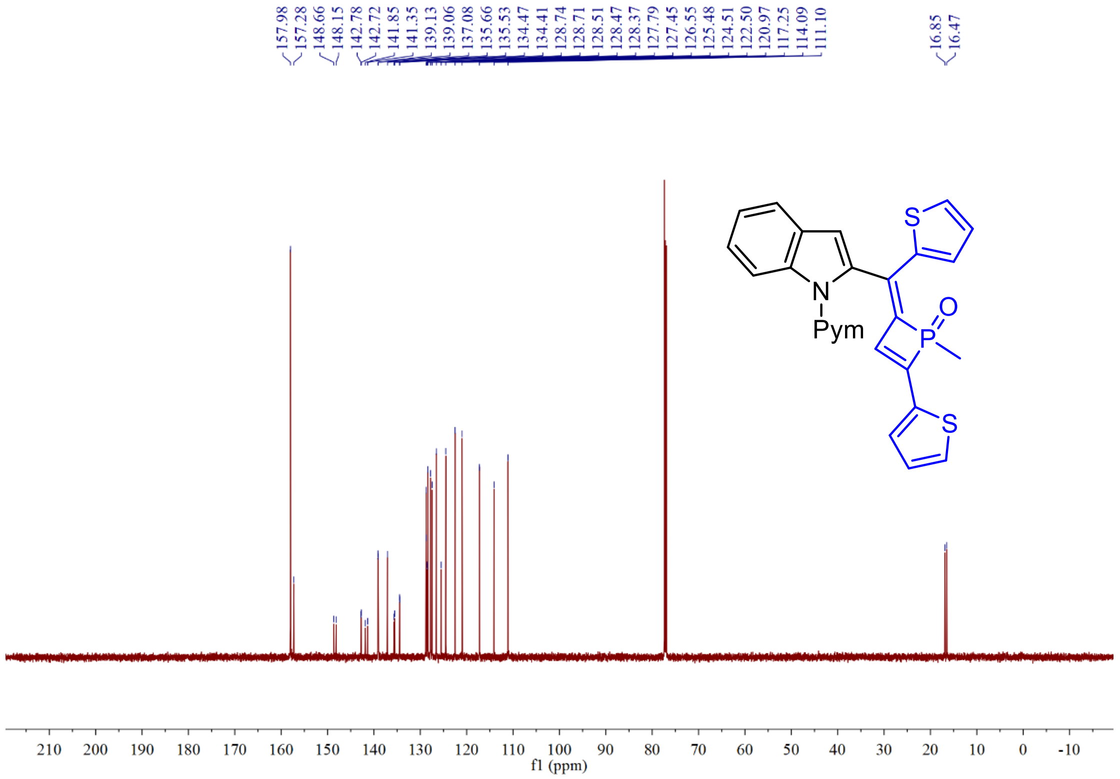

^{13}C NMR spectrum of compound **4h**

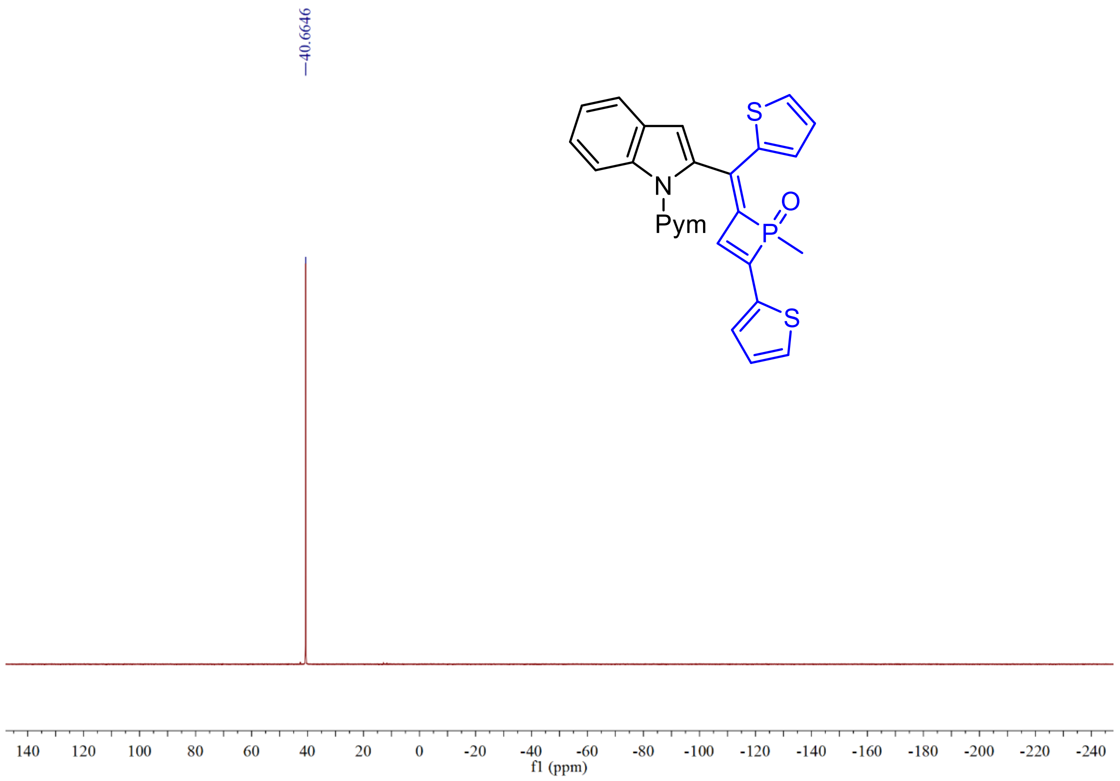

^{31}P NMR spectrum of compound **4h**

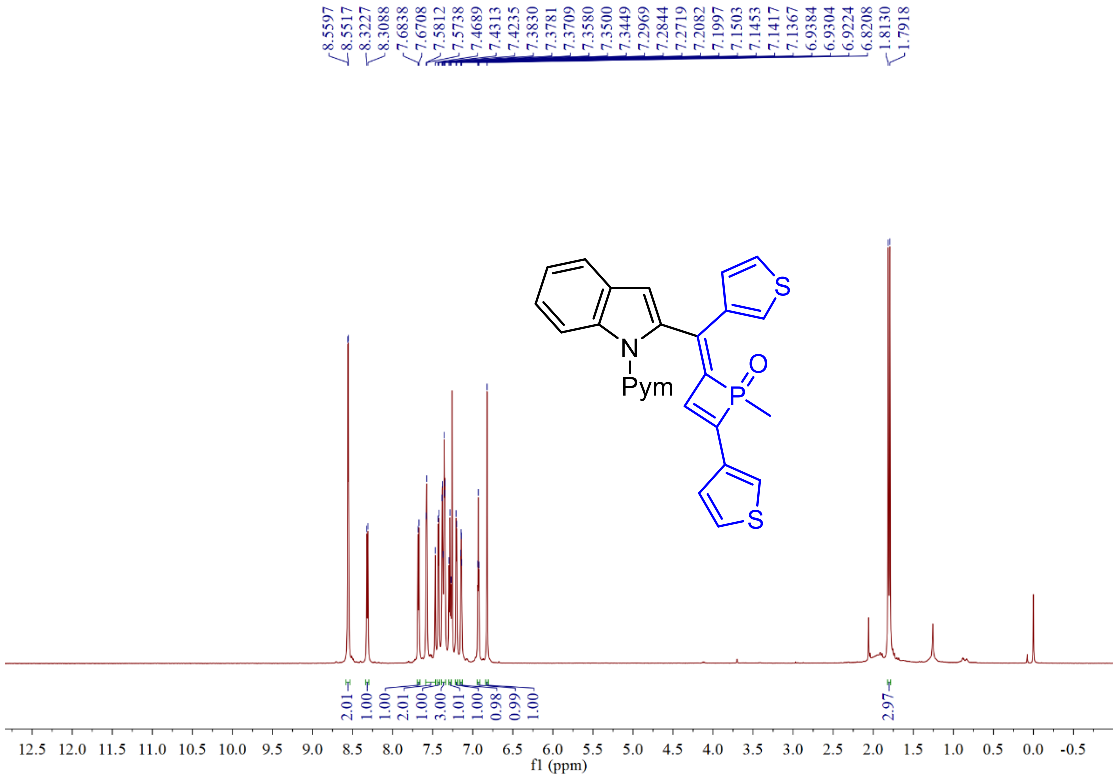

^{1}H NMR spectrum of compound **4i**

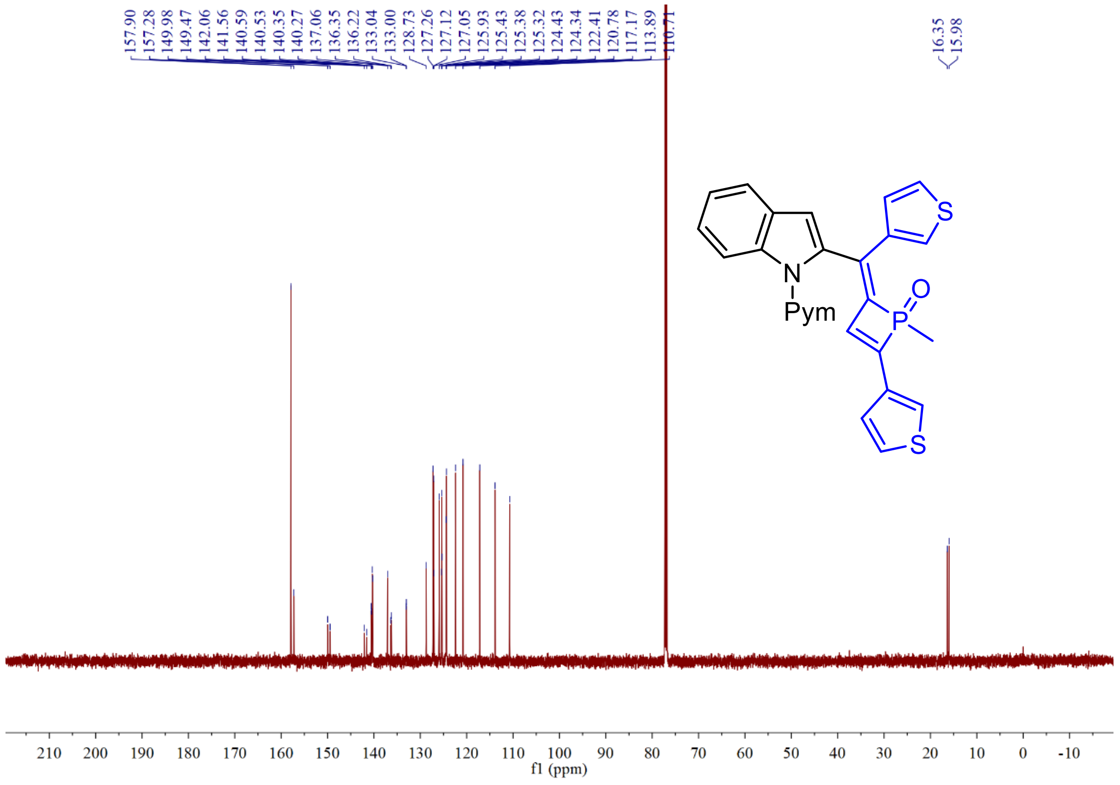

^{13}C NMR spectrum of compound **4i**

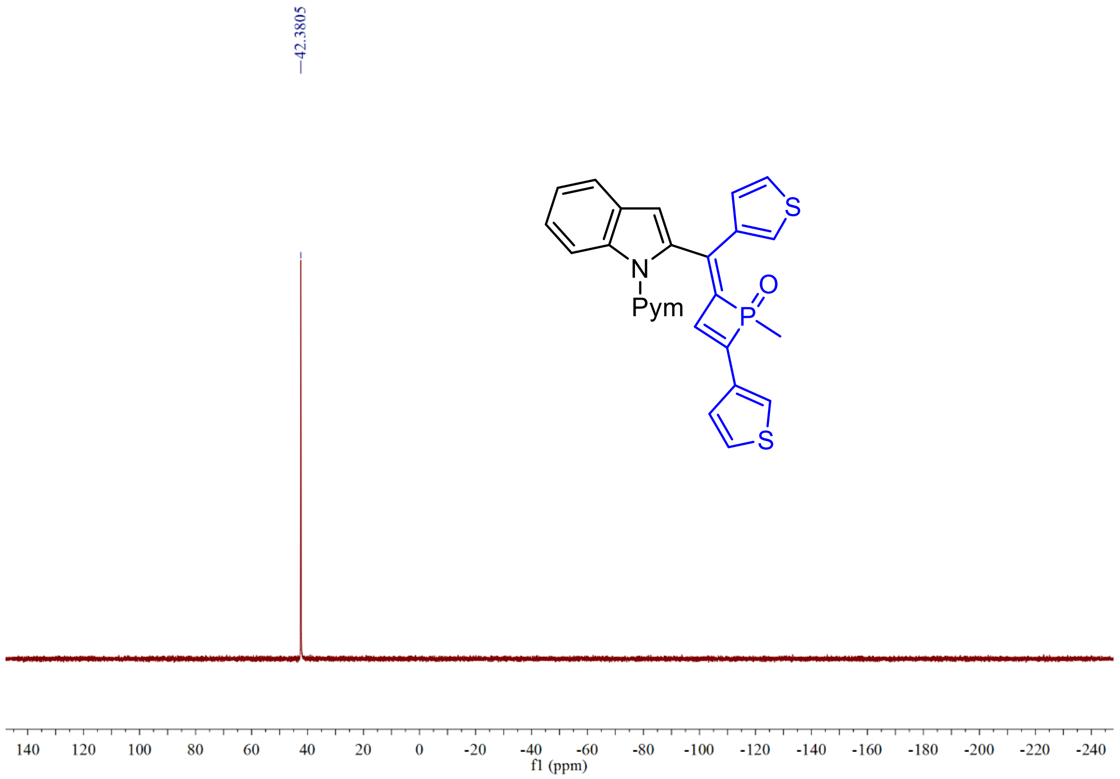

^{31}P NMR spectrum of compound **4i**

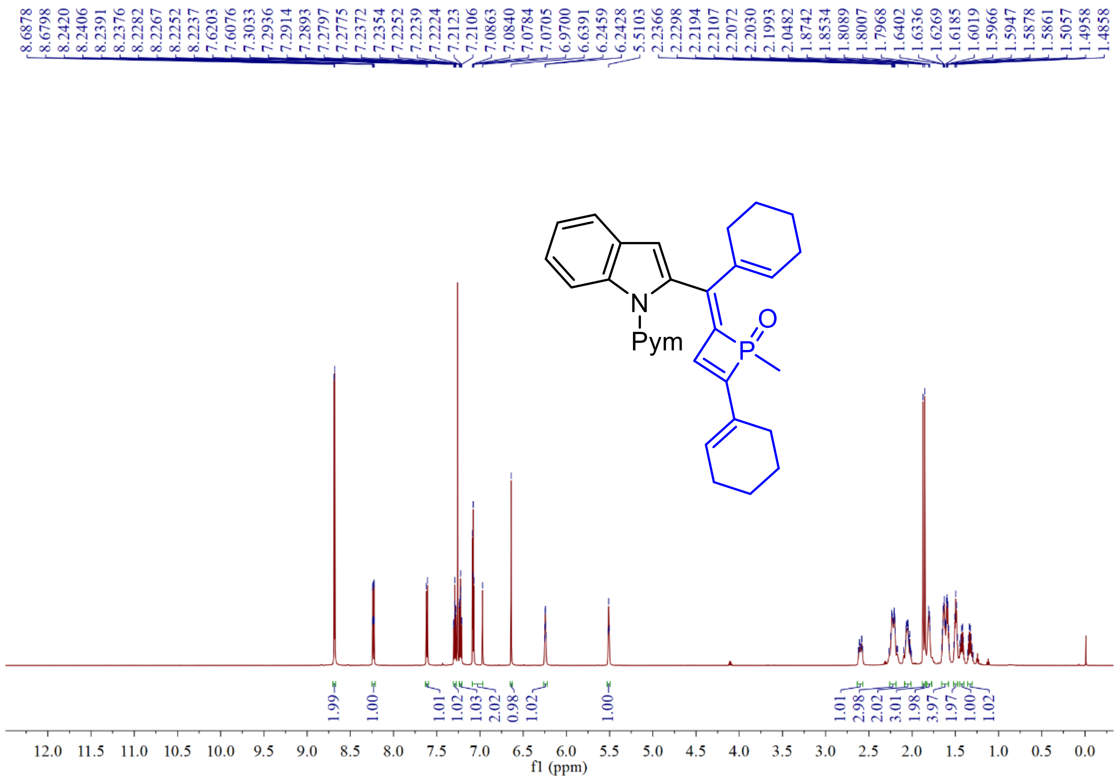

^{1}H NMR spectrum of compound **4j**

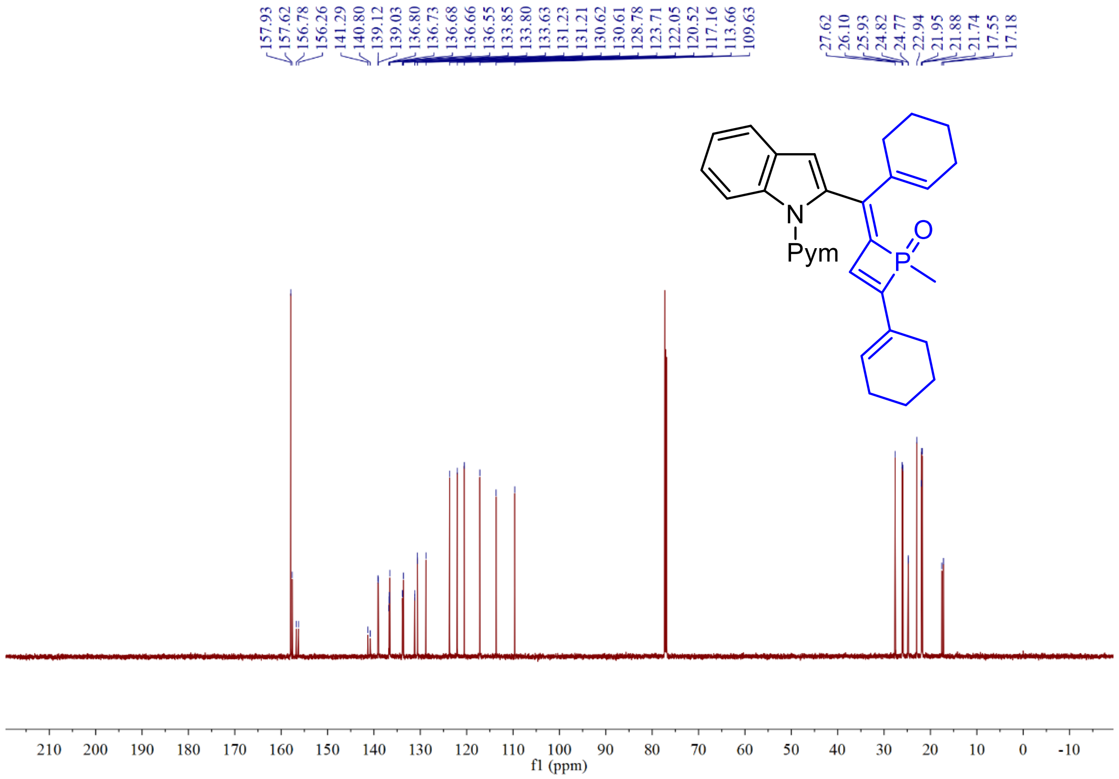

^{13}C NMR spectrum of compound **4j**

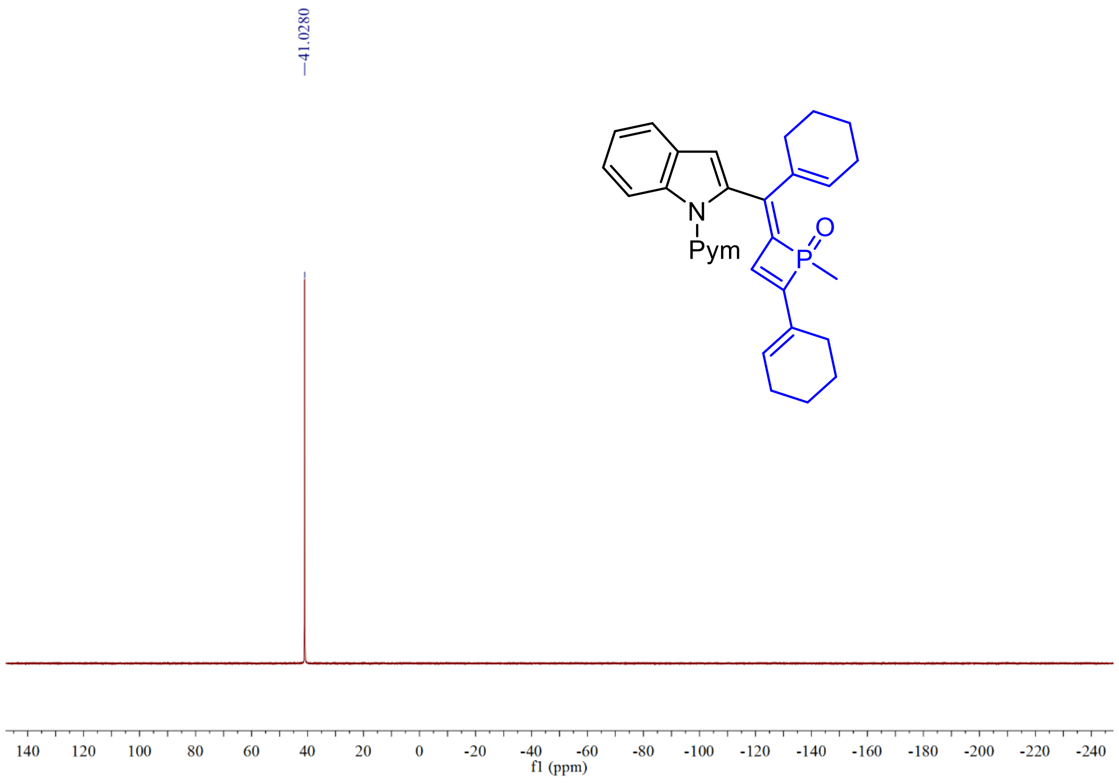

^{31}P NMR spectrum of compound **4j**

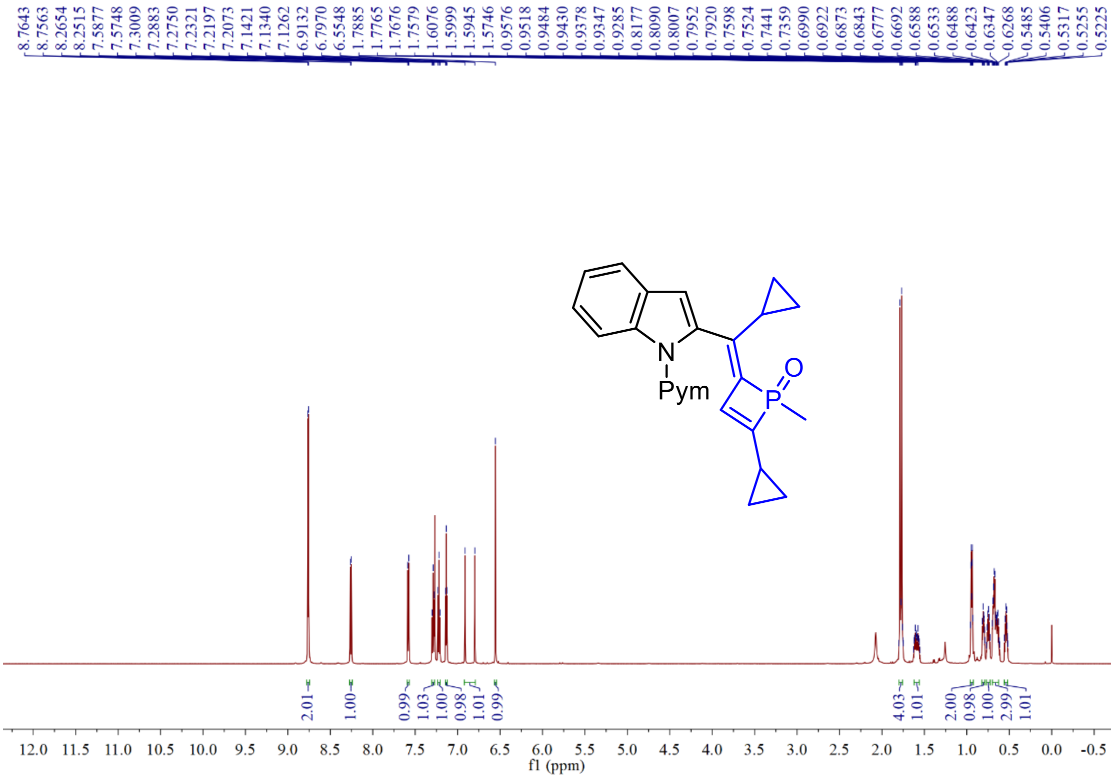

^{1}H NMR spectrum of compound **4k**

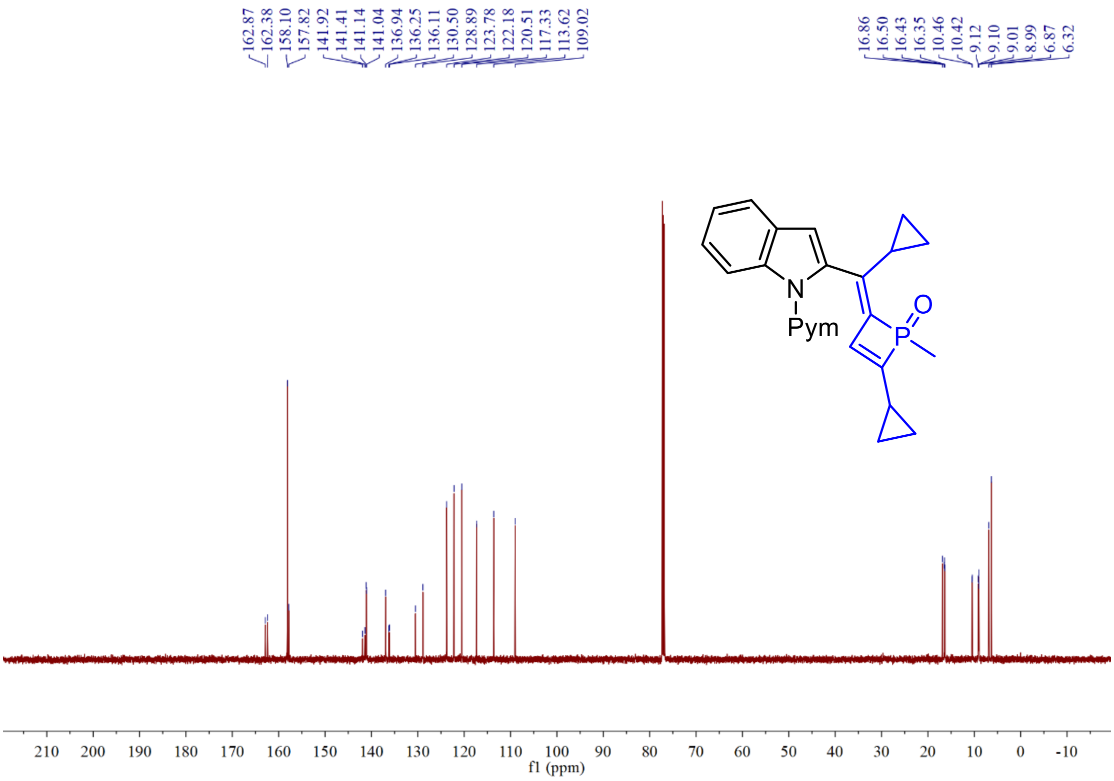

^{13}C NMR spectrum of compound **4k**

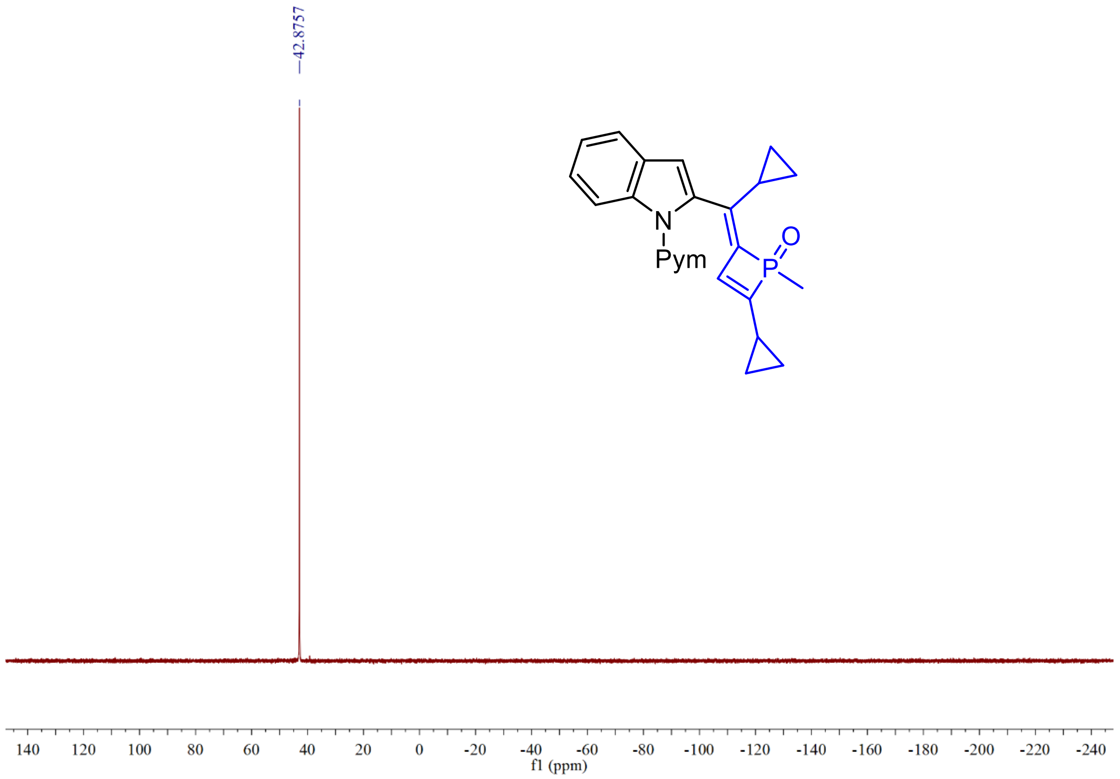

^{31}P NMR spectrum of compound **4k**

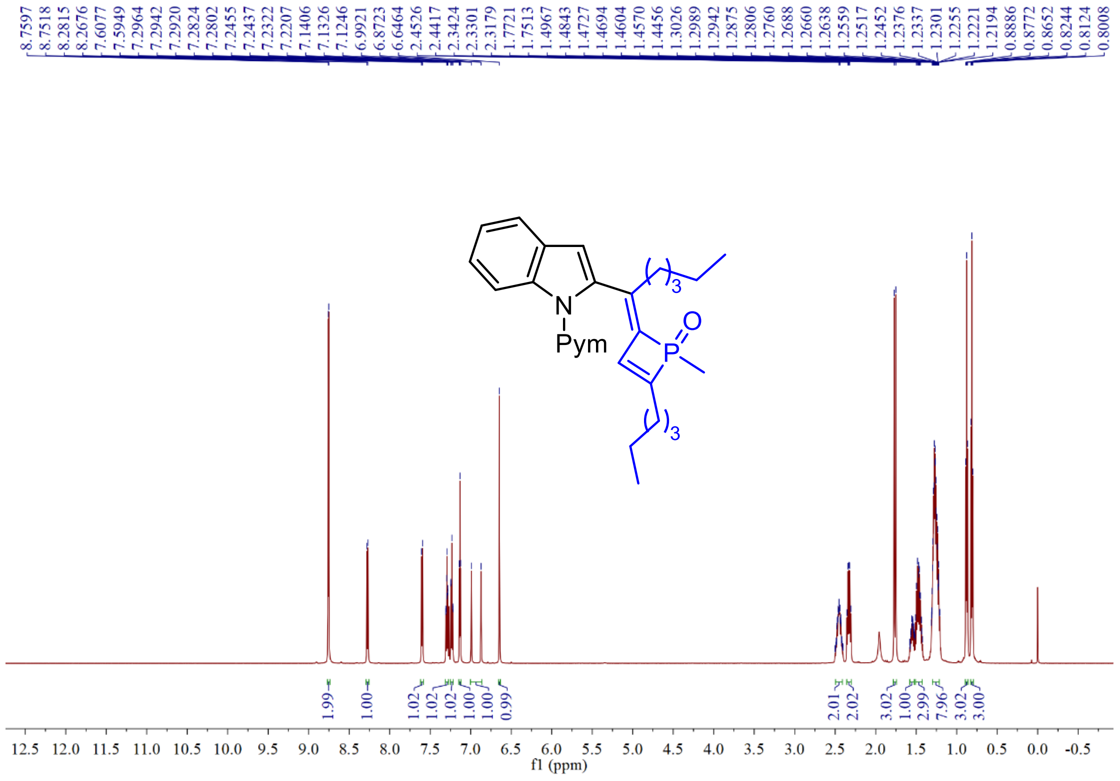

^{1}H NMR spectrum of compound **4l**

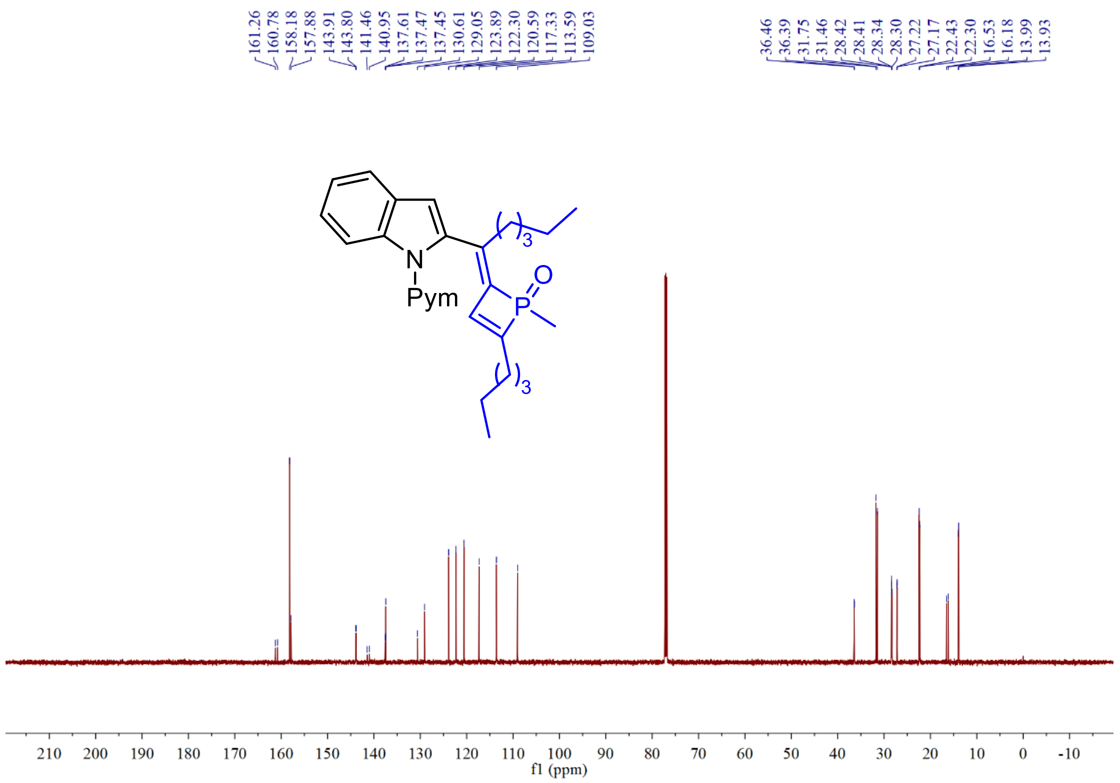

^{13}C NMR spectrum of compound **4l**

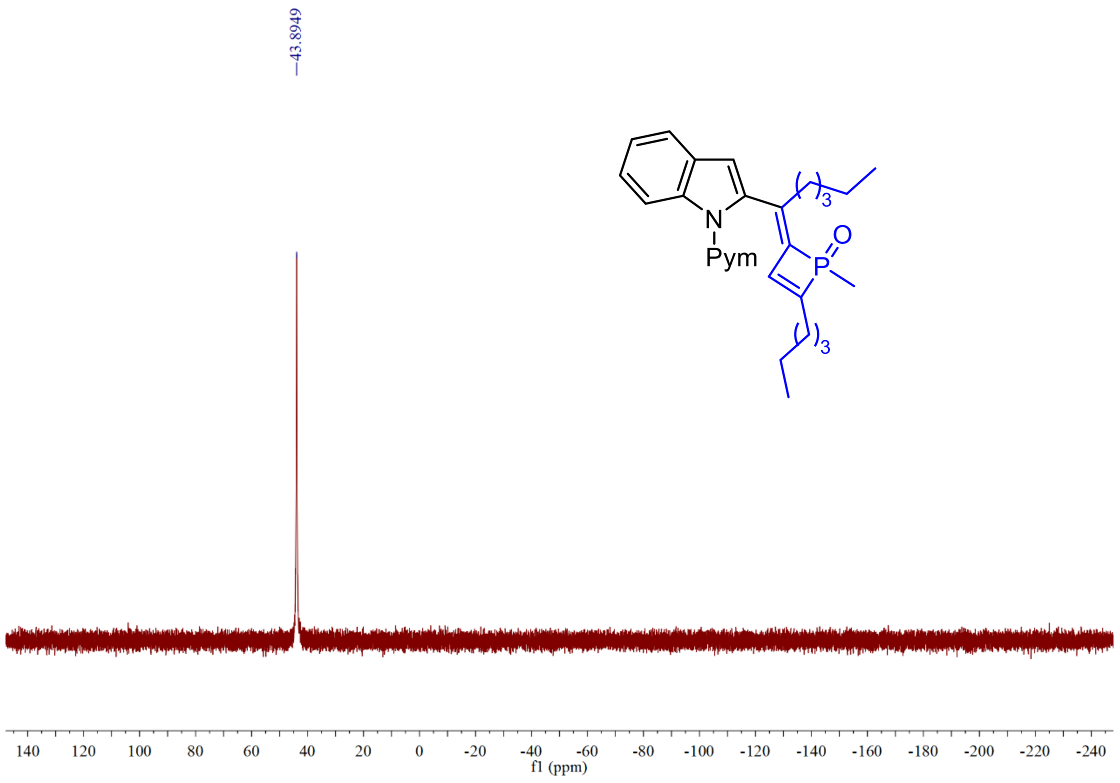

^{31}P NMR spectrum of compound **4l**

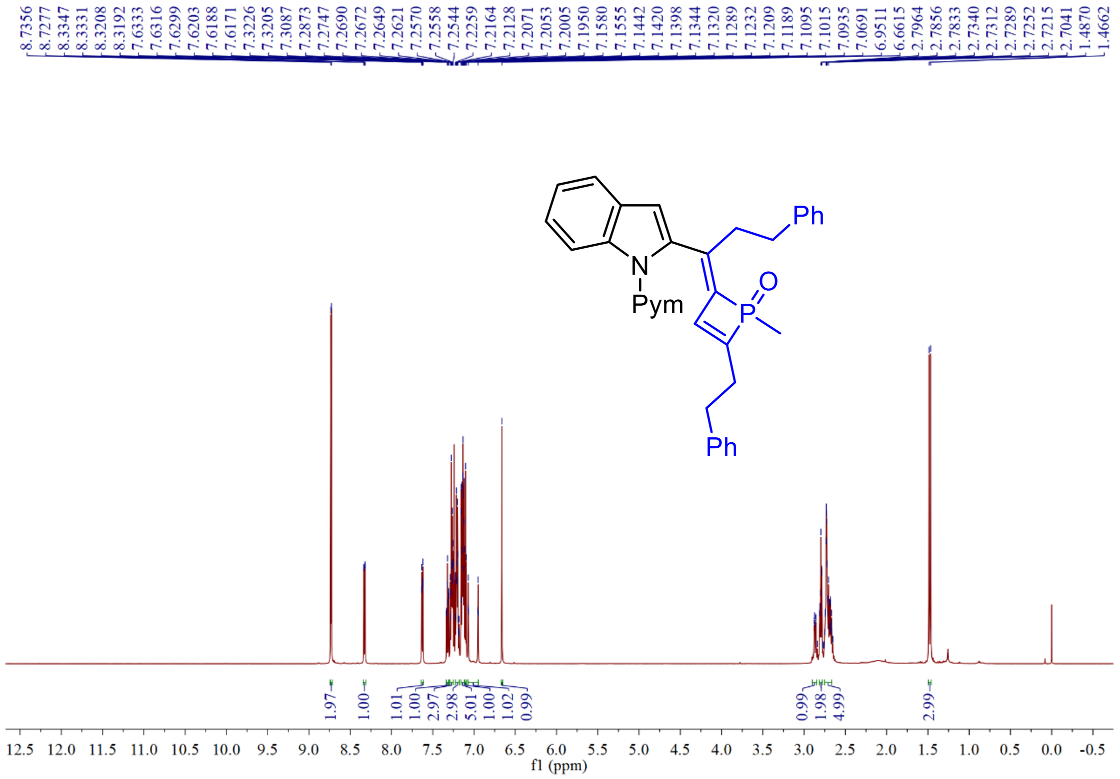

^{1}H NMR spectrum of compound **4m**

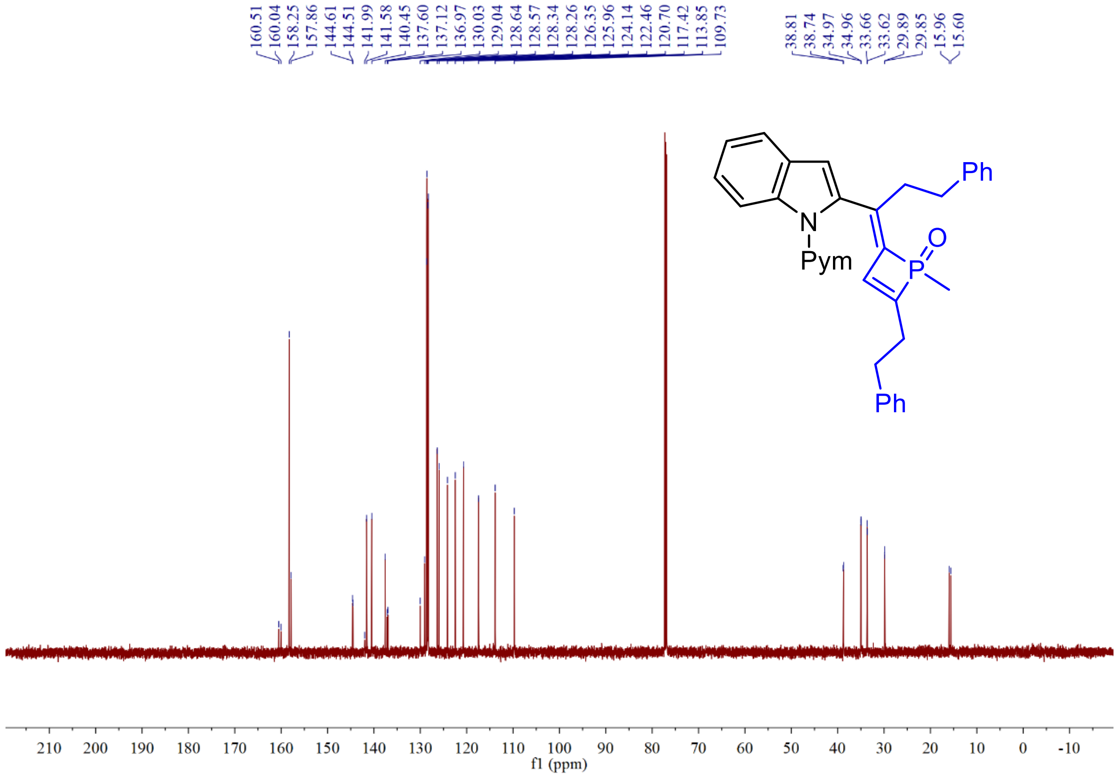

^{13}C NMR spectrum of compound **4m**

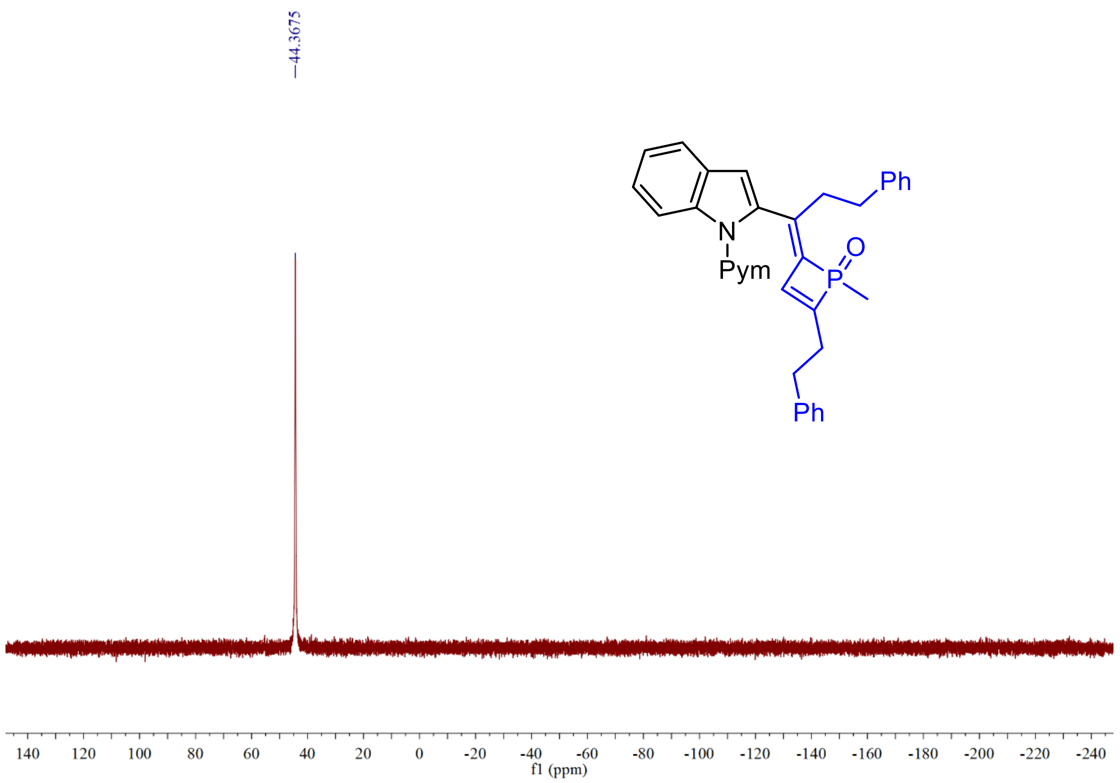

^{31}P NMR spectrum of compound **4m**

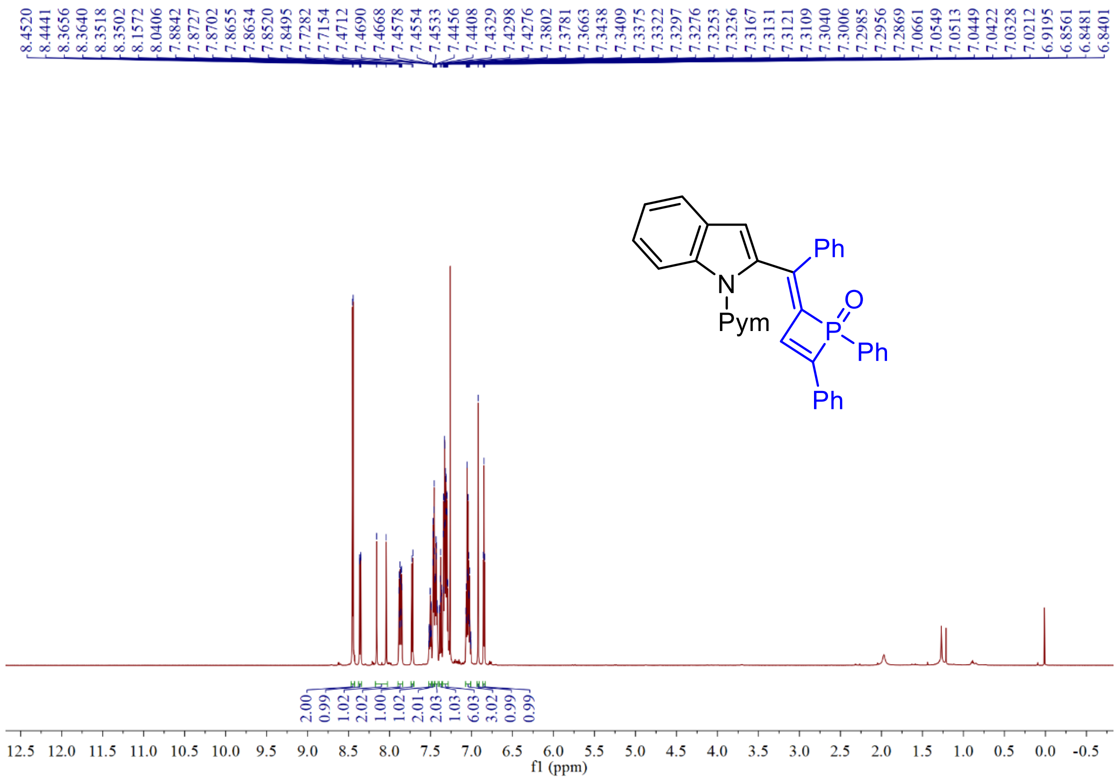

^{1}H NMR spectrum of compound **4n**

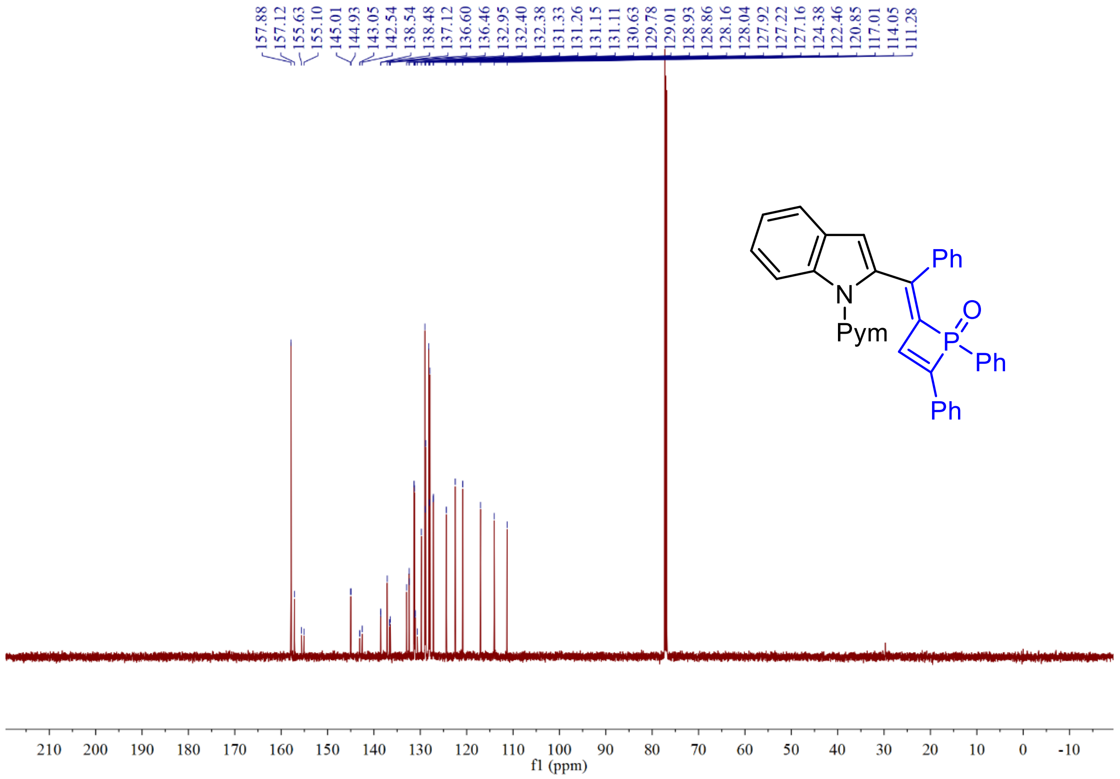

^{13}C NMR spectrum of compound **4n**

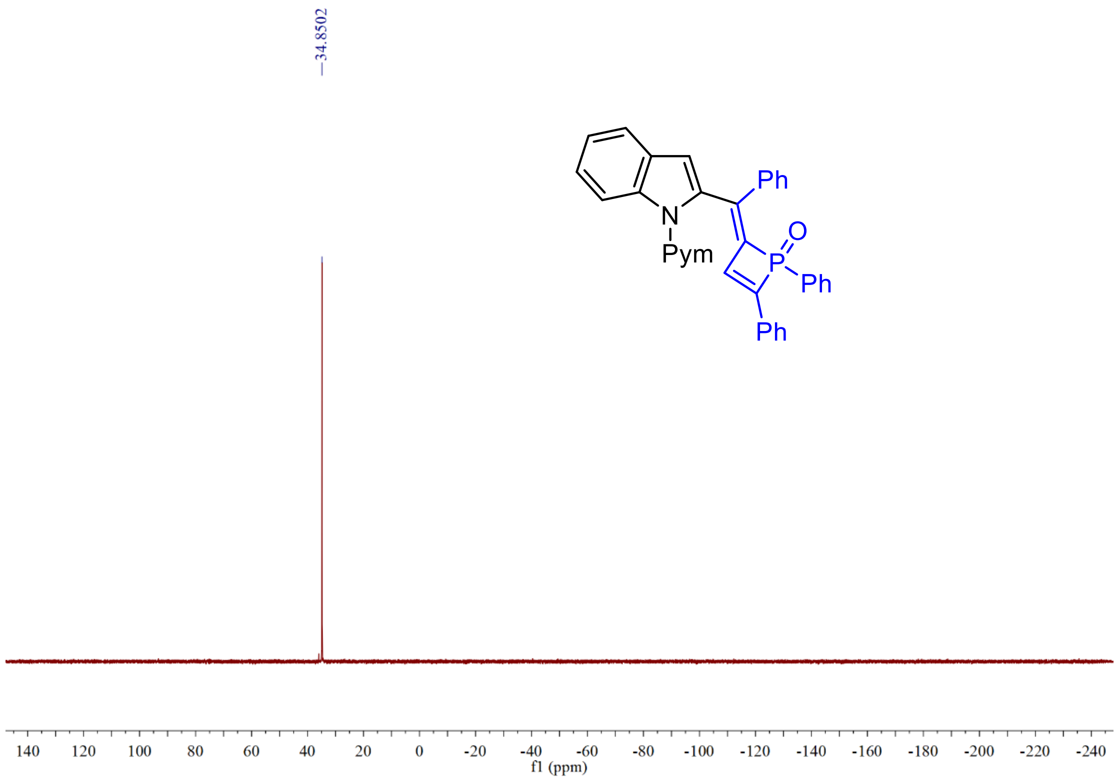

^{31}P NMR spectrum of compound **4n**

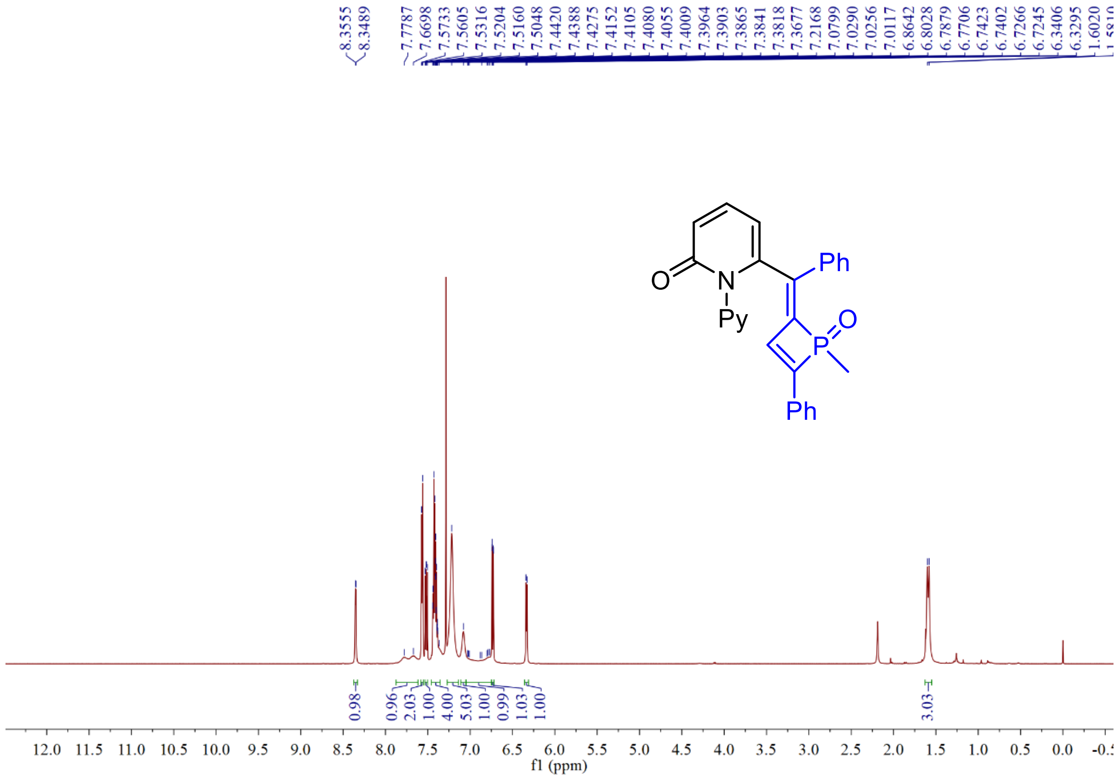

^{1}H NMR spectrum of compound **6a**

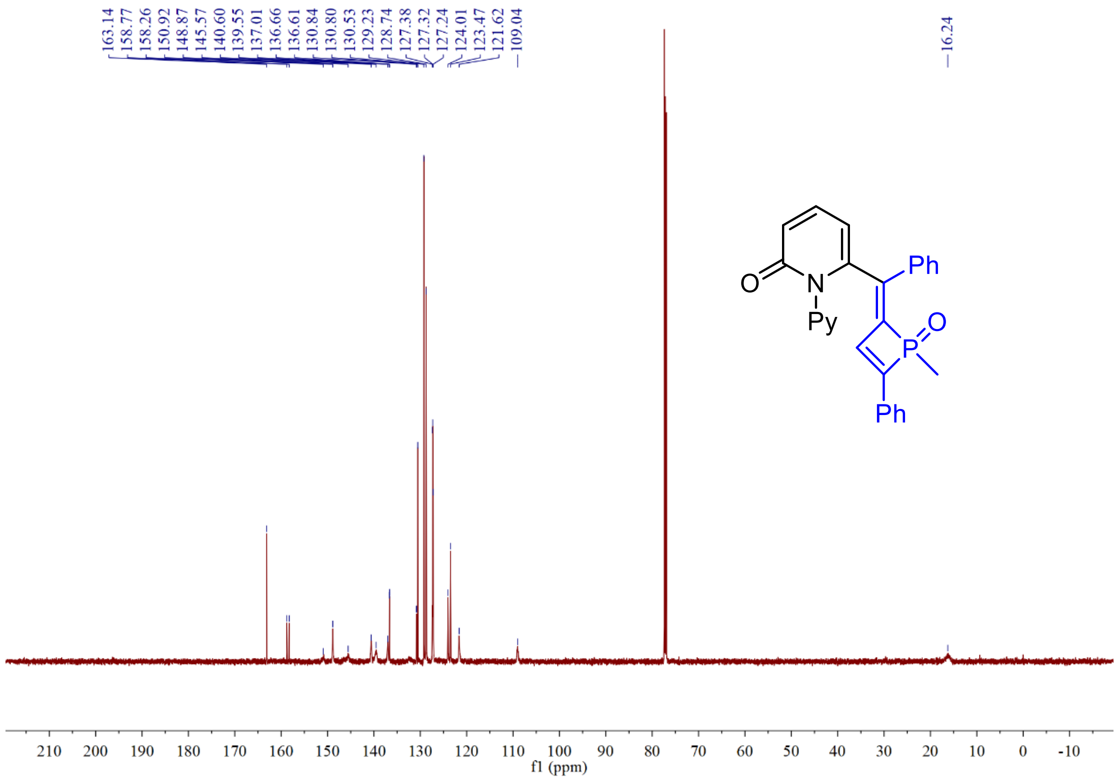

^{13}C NMR spectrum of compound **6a**

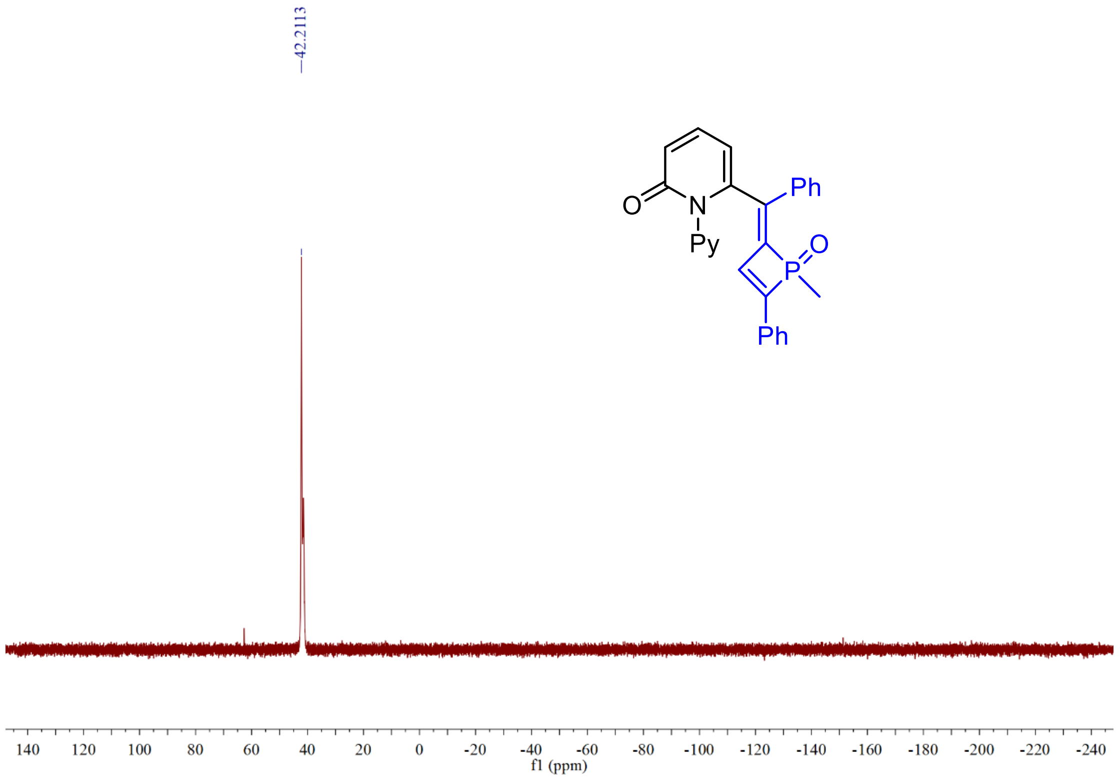

^{31}P NMR spectrum of compound **6a**

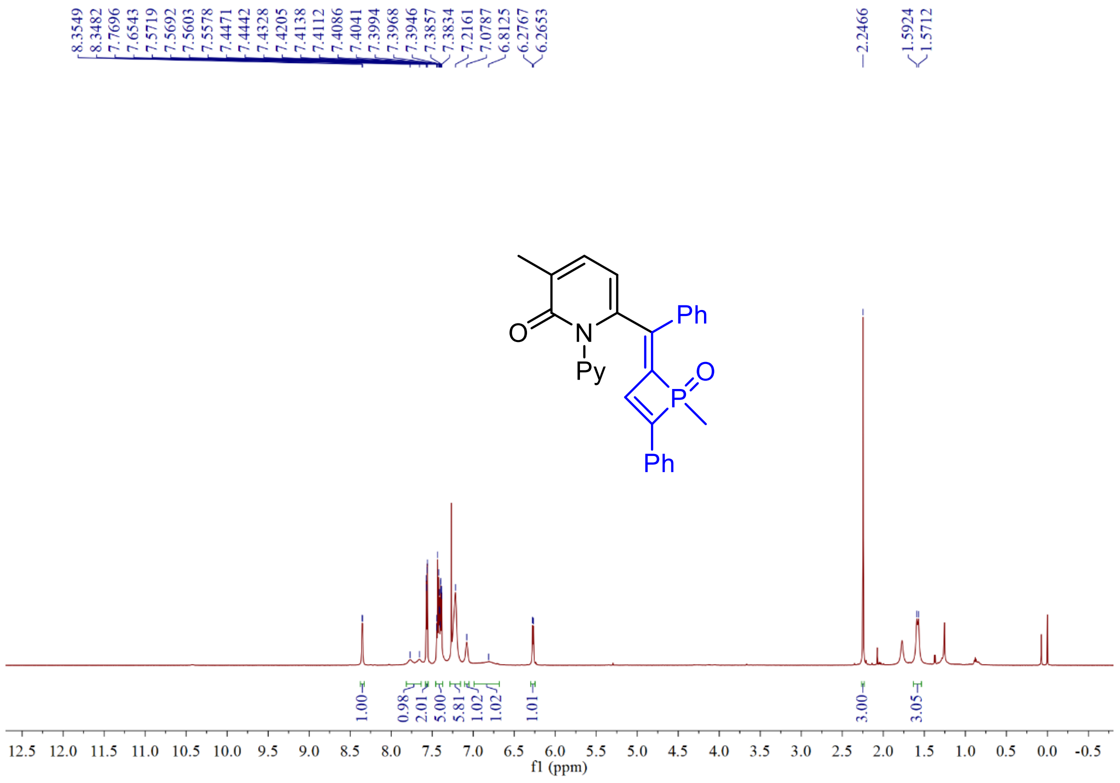

^{1}H NMR spectrum of compound **6b**

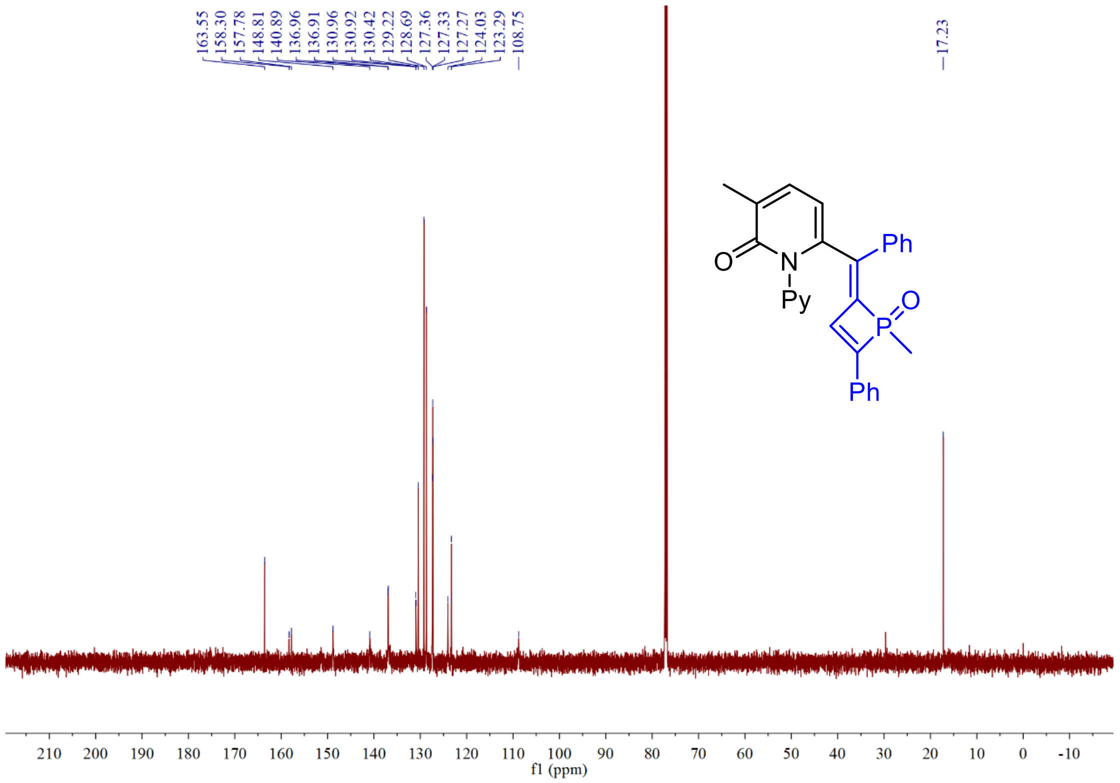

^{13}C NMR spectrum of compound **6b**

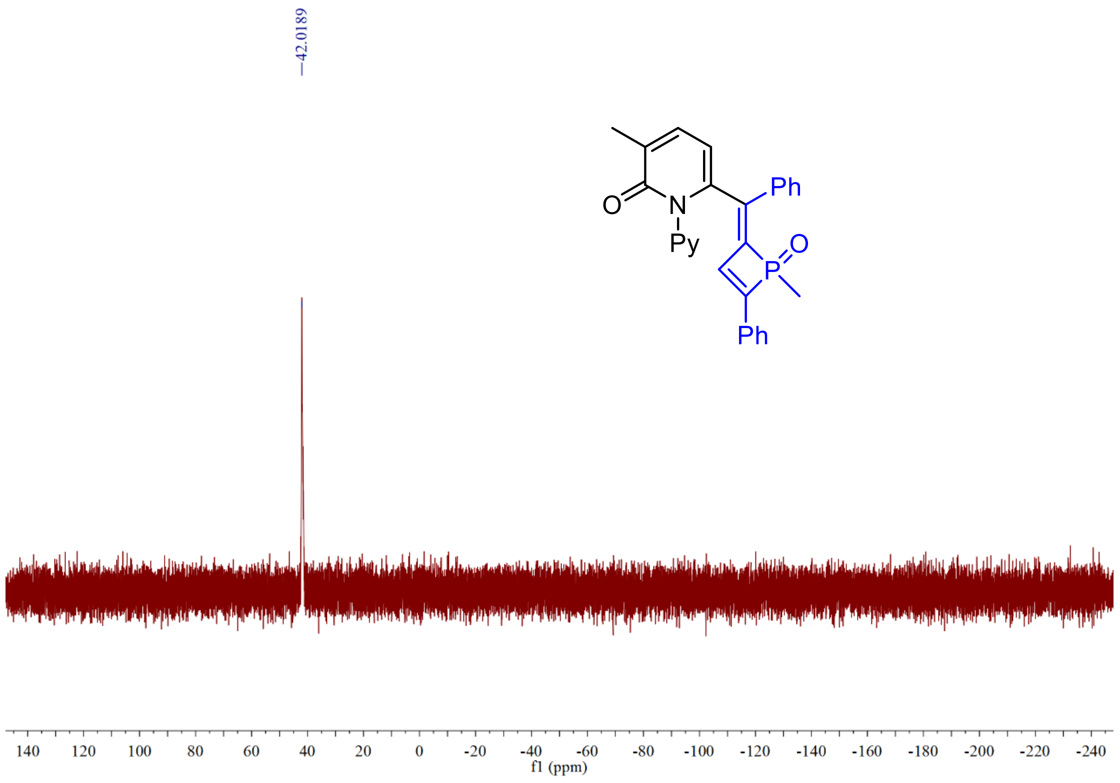

^{31}P NMR spectrum of compound **6b**

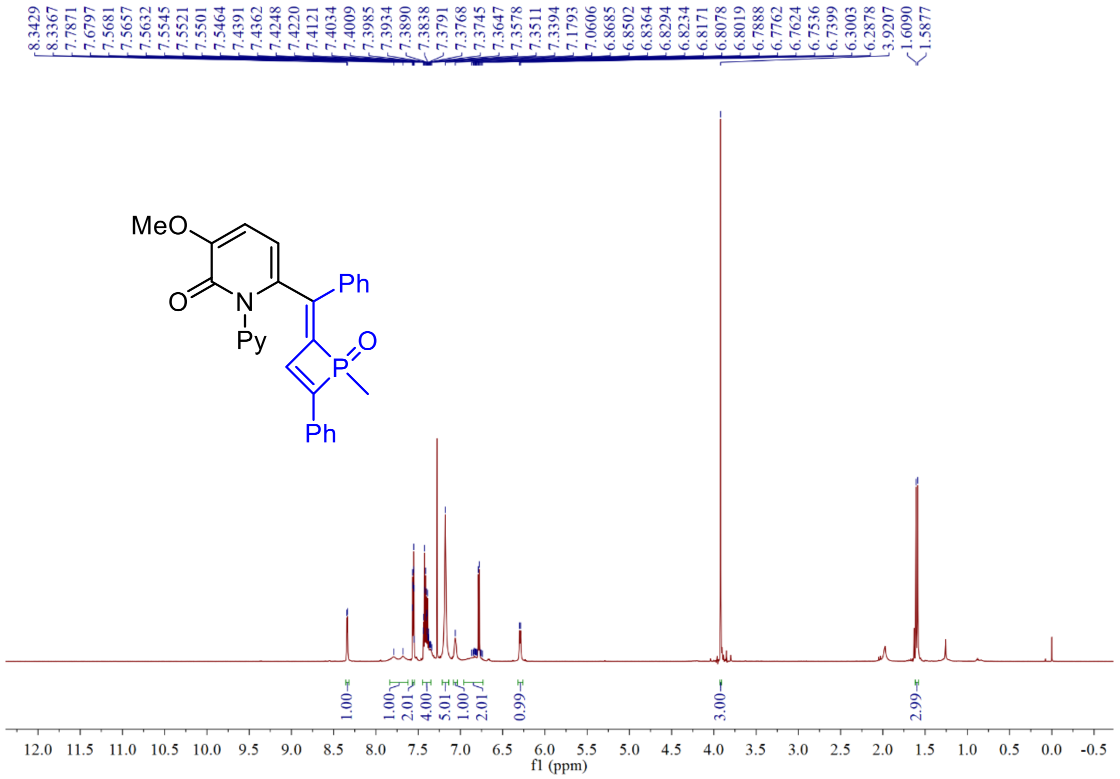

^{1}H NMR spectrum of compound **6c**

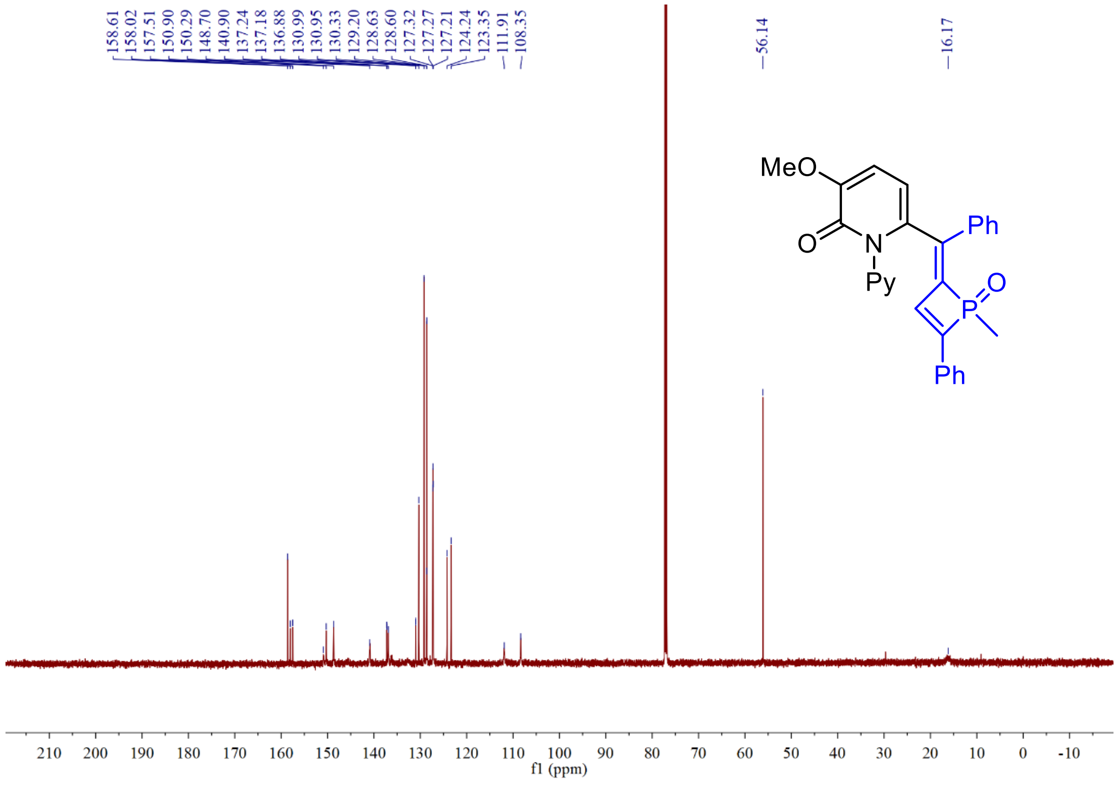

^{13}C NMR spectrum of compound **6c**

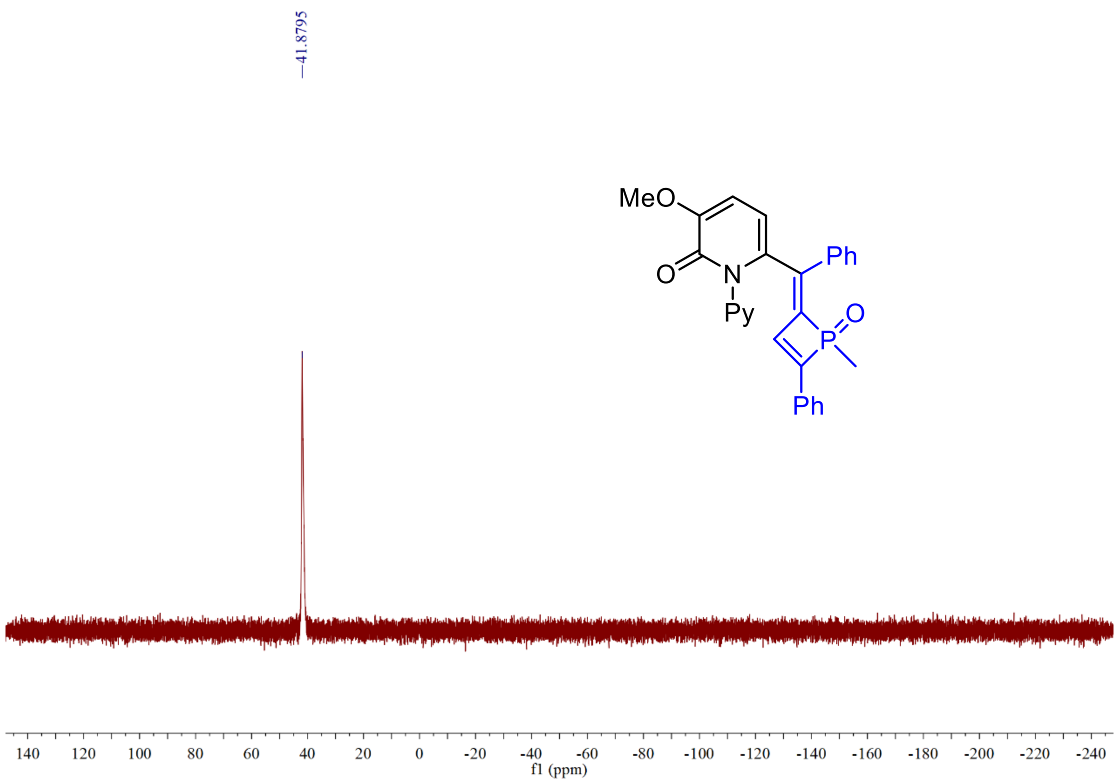

^{31}P NMR spectrum of compound **6c**

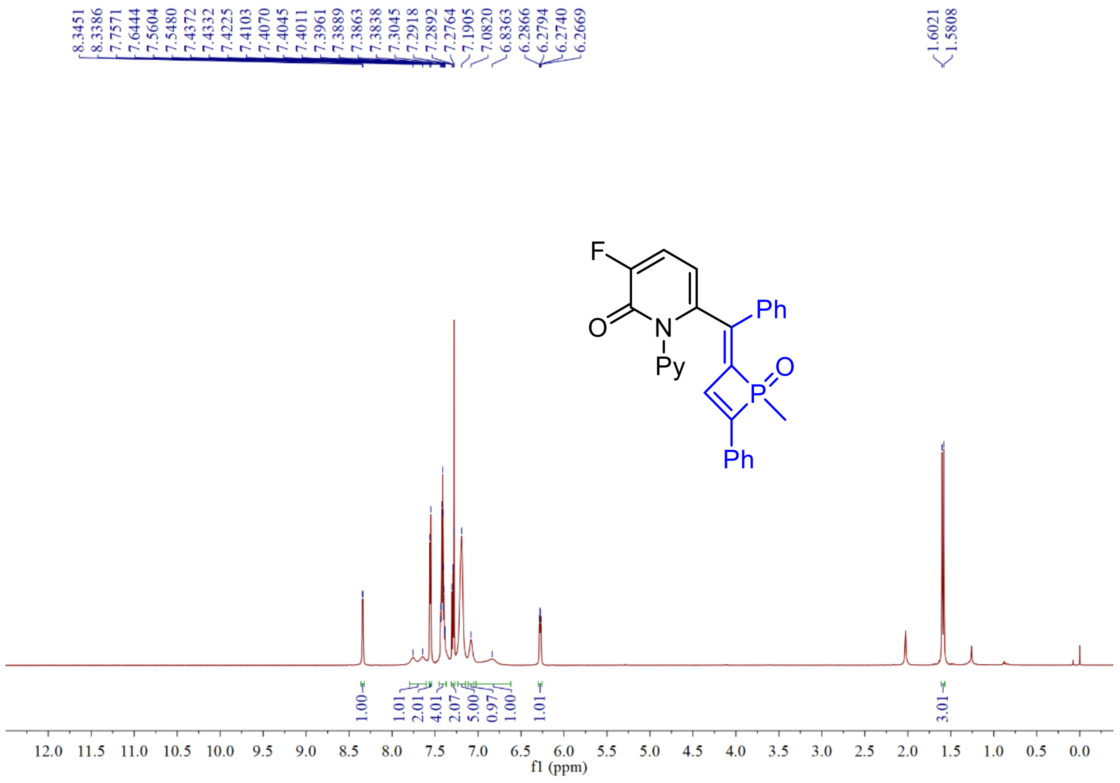

^{1}H NMR spectrum of compound **6d**

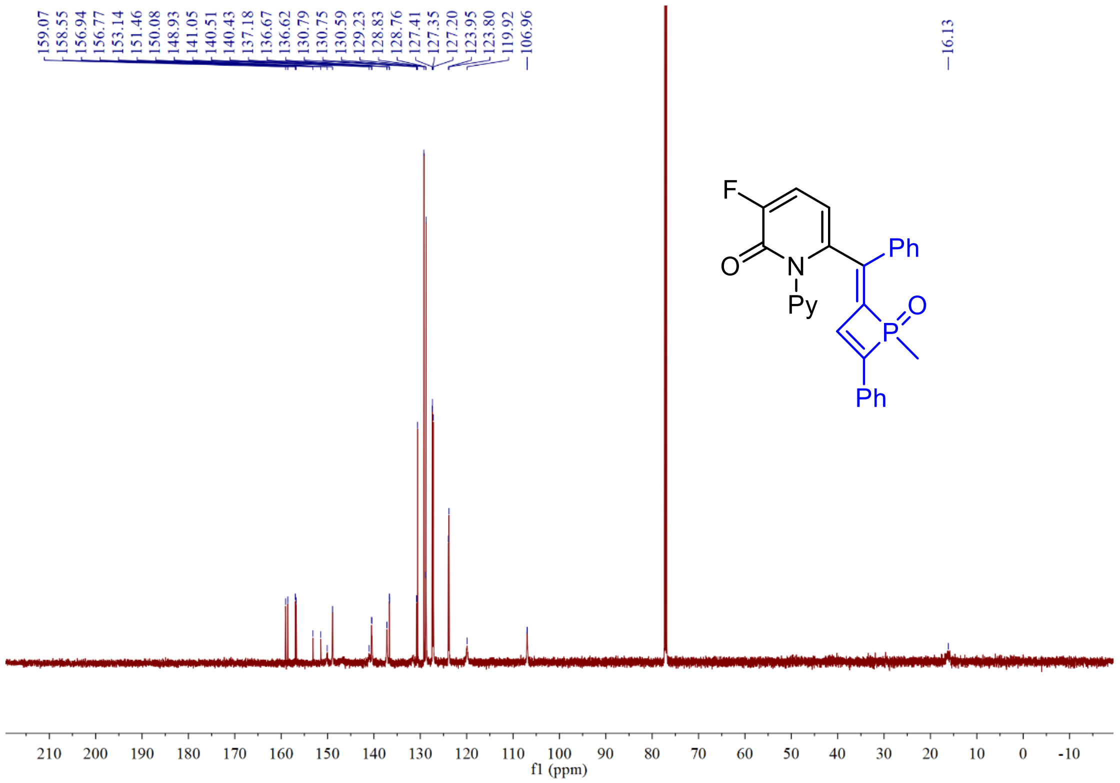

^{13}C NMR spectrum of compound **6d**

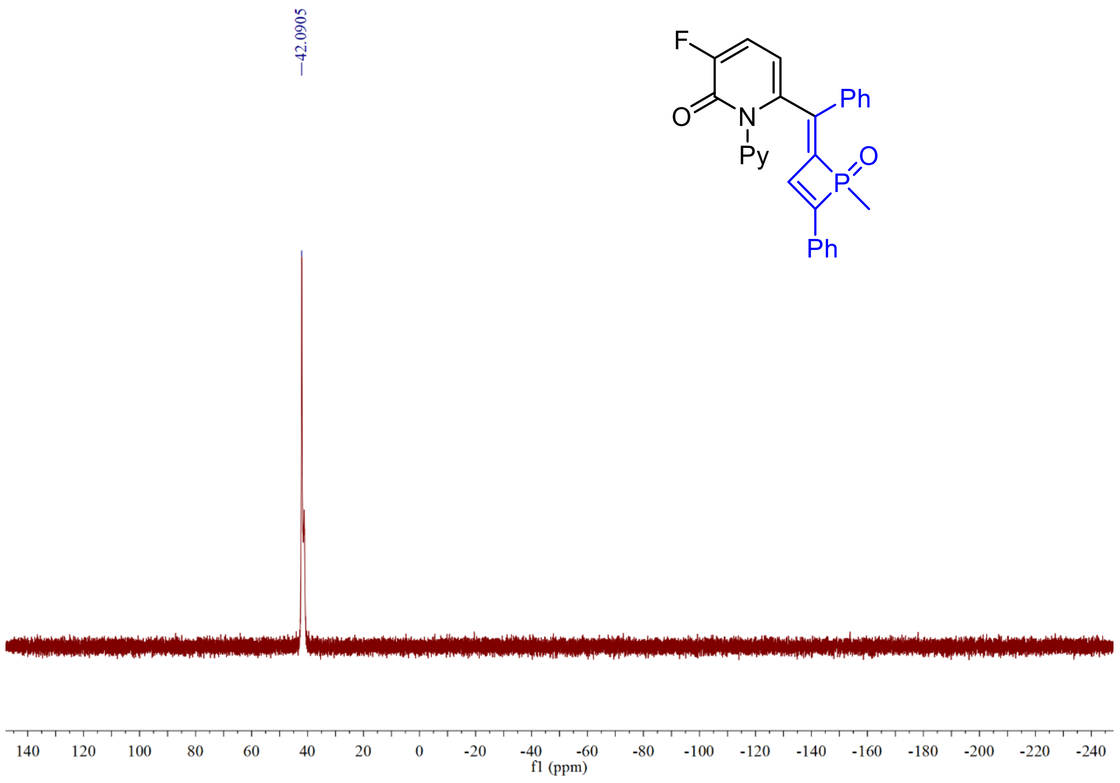

^{31}P NMR spectrum of compound **6d**

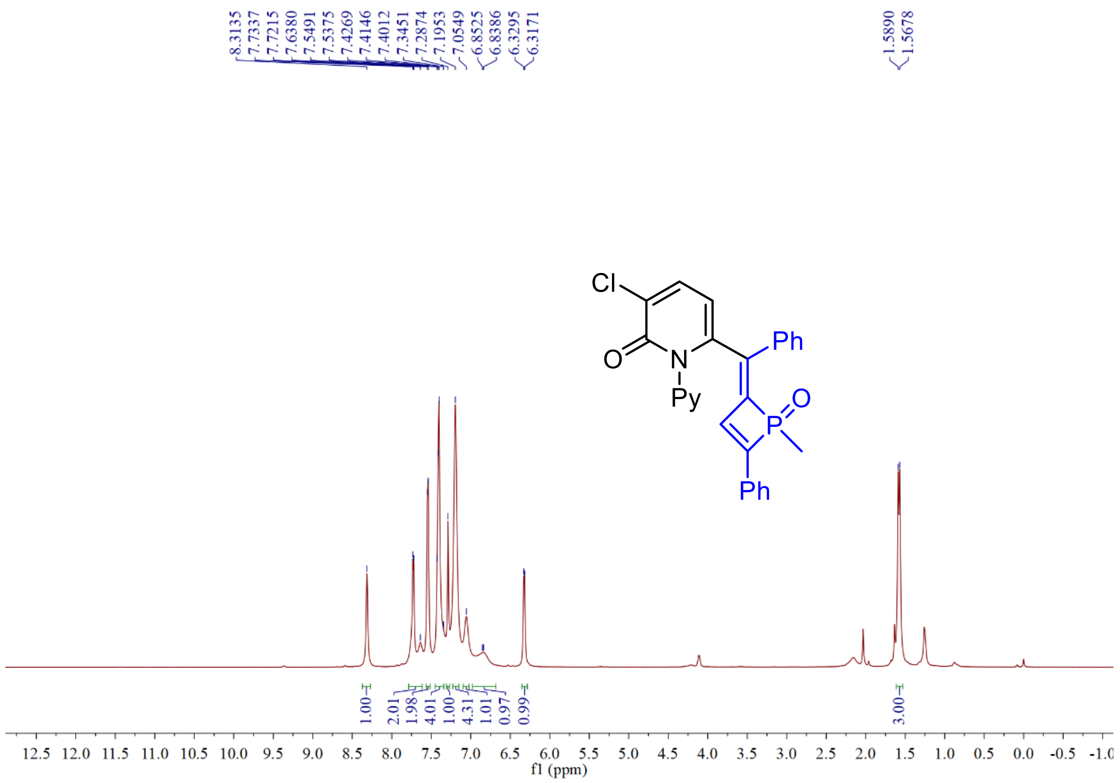

^{1}H NMR spectrum of compound **6e**

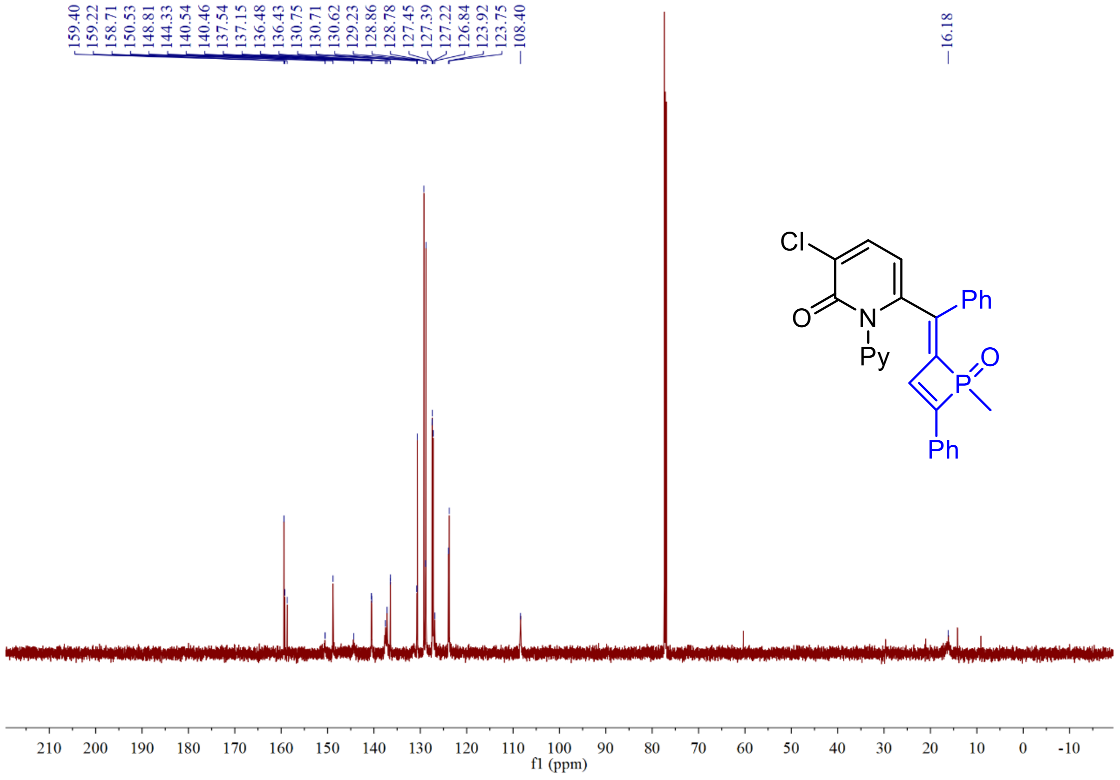

^{13}C NMR spectrum of compound **6e**

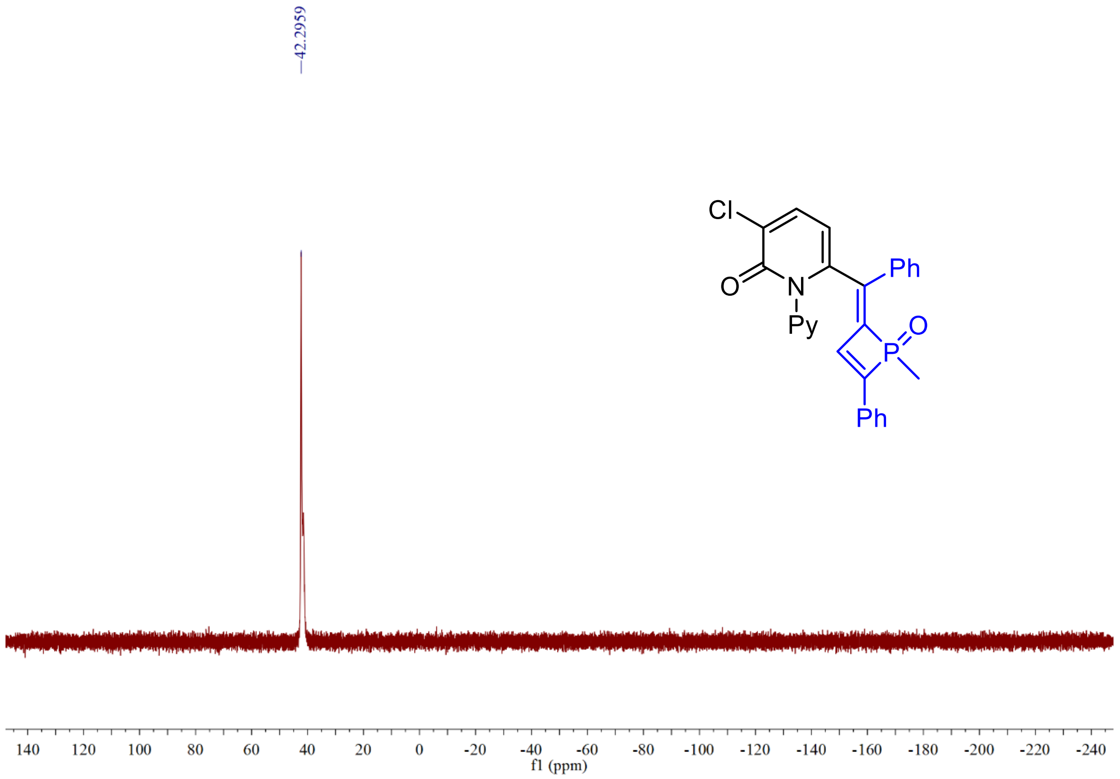

^{31}P NMR spectrum of compound **6e**

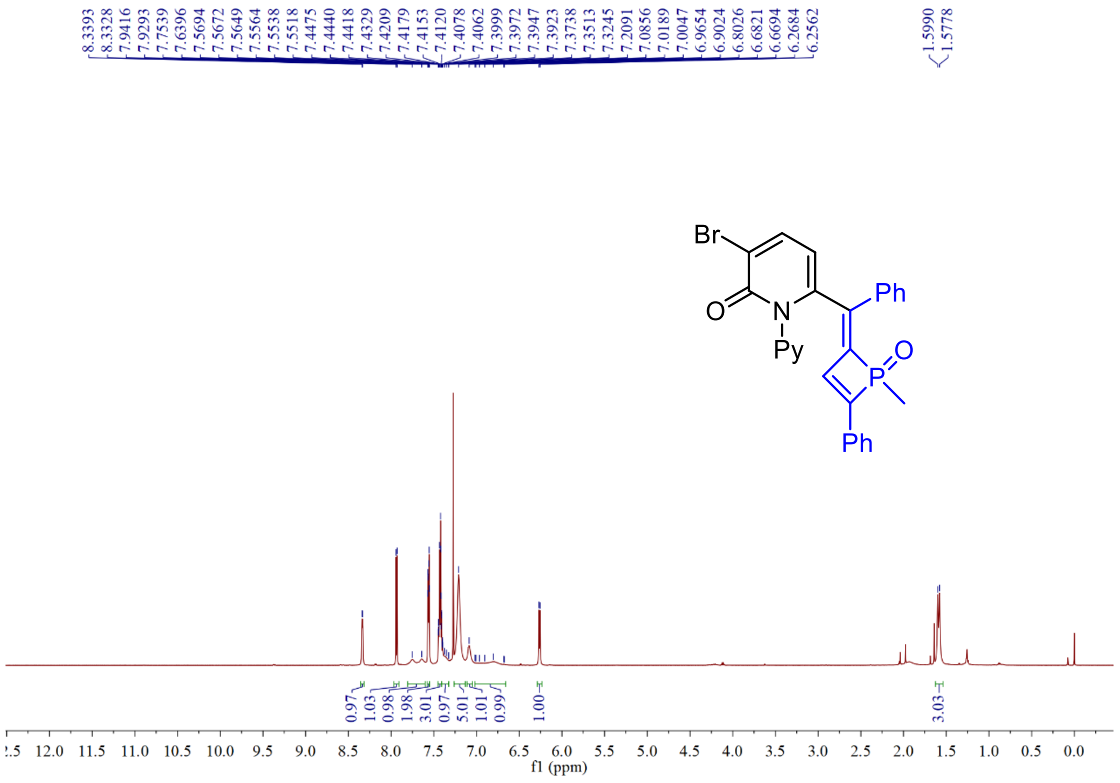

^{1}H NMR spectrum of compound **6f**

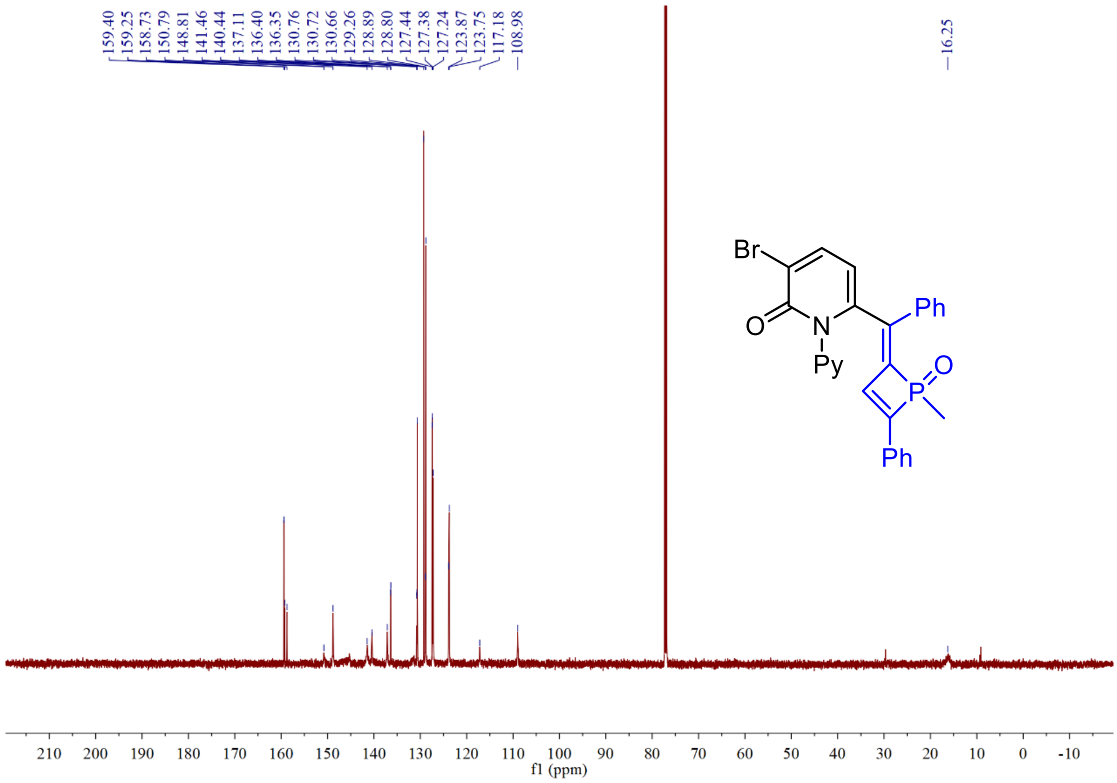

^{13}C NMR spectrum of compound **6f**

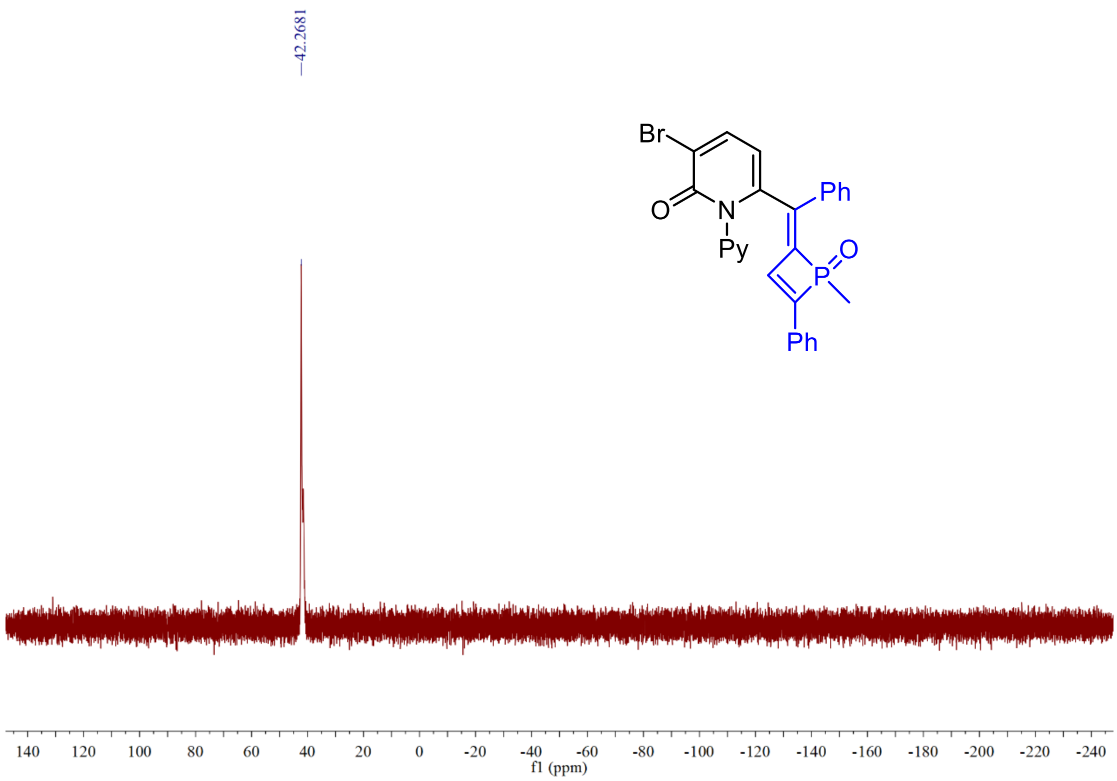

^{31}P NMR spectrum of compound **6f**

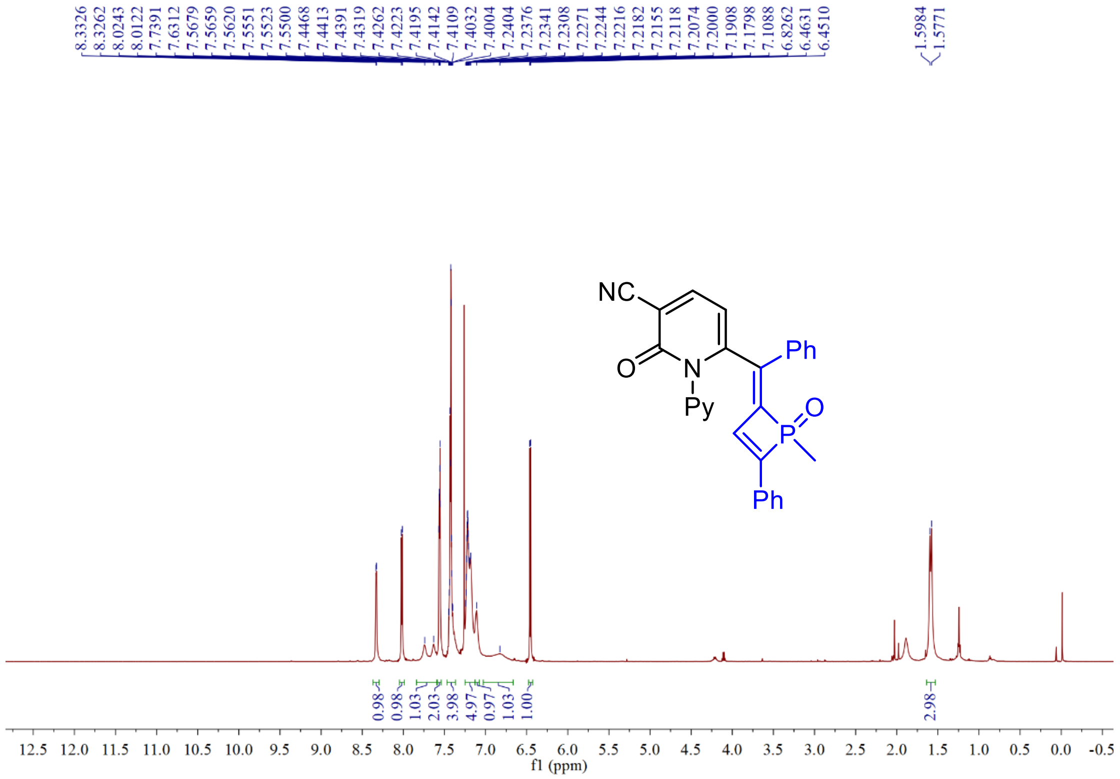

^{1}H NMR spectrum of compound **6g**

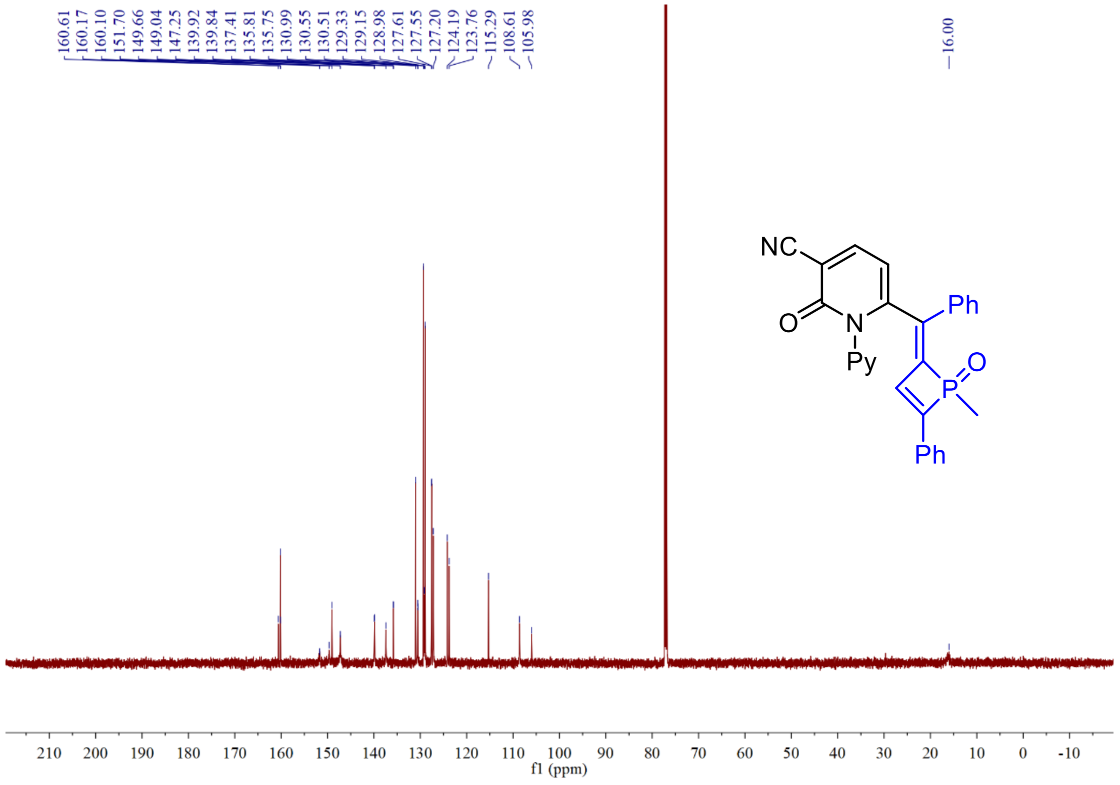

^{13}C NMR spectrum of compound **6g**

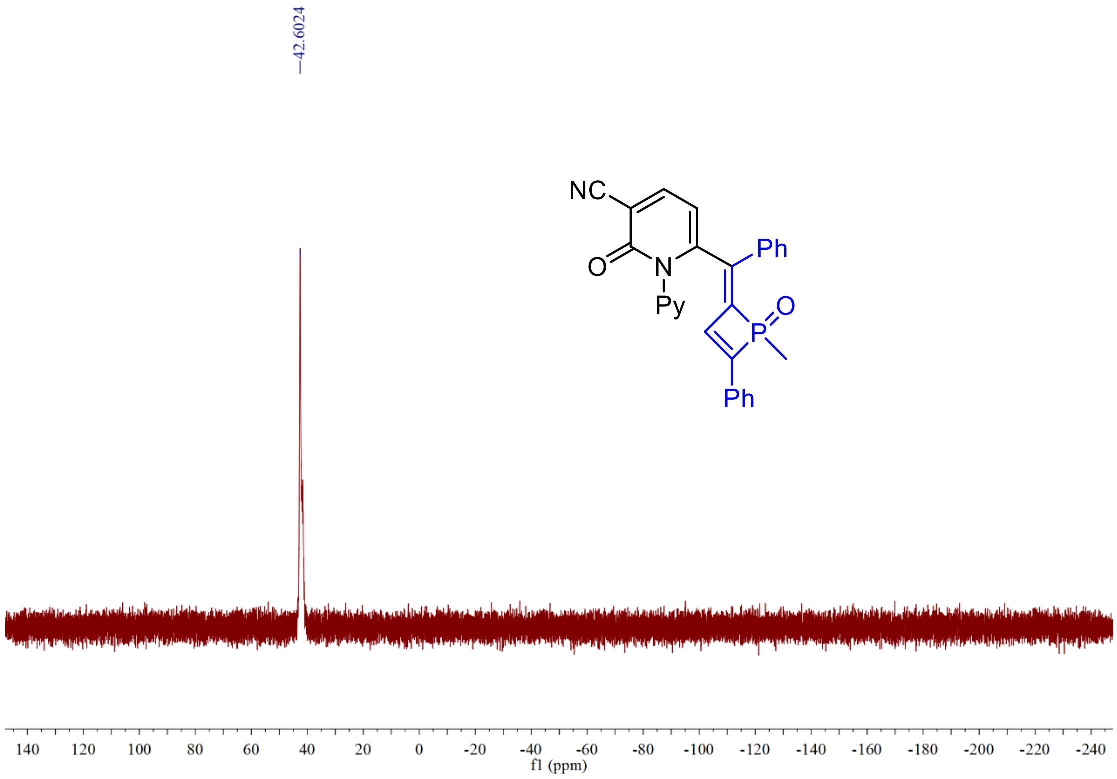

^{31}P NMR spectrum of compound **6g**

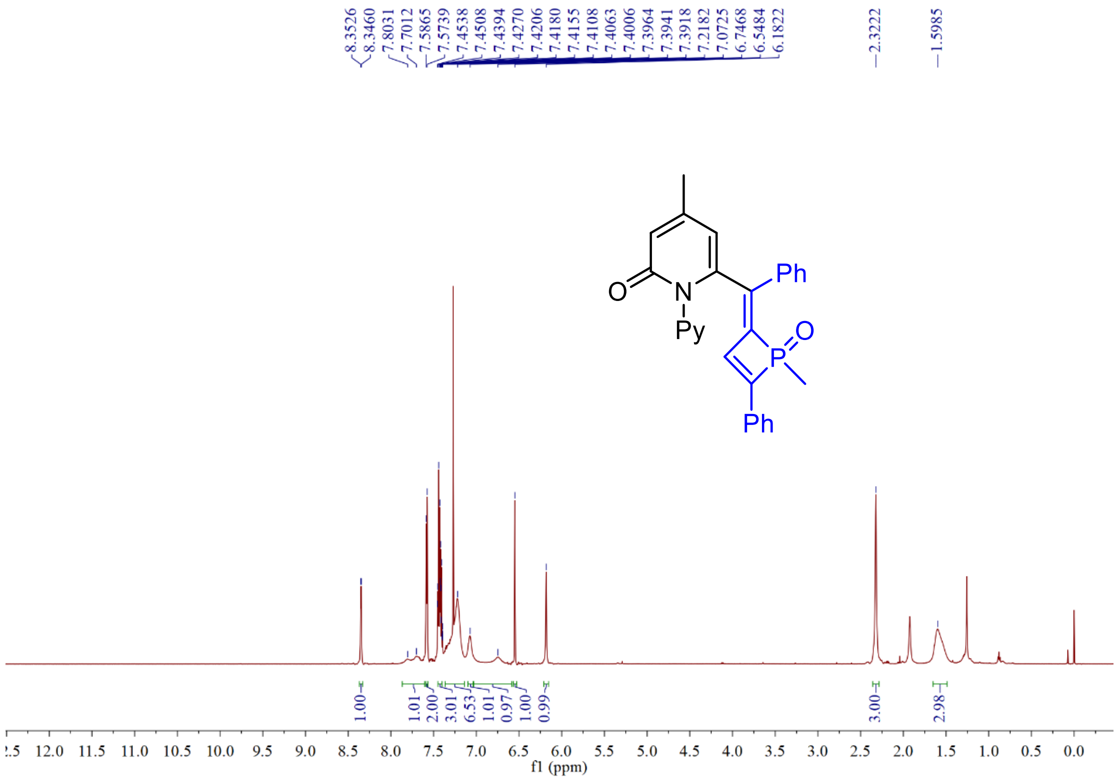

^{1}H NMR spectrum of compound **6h**

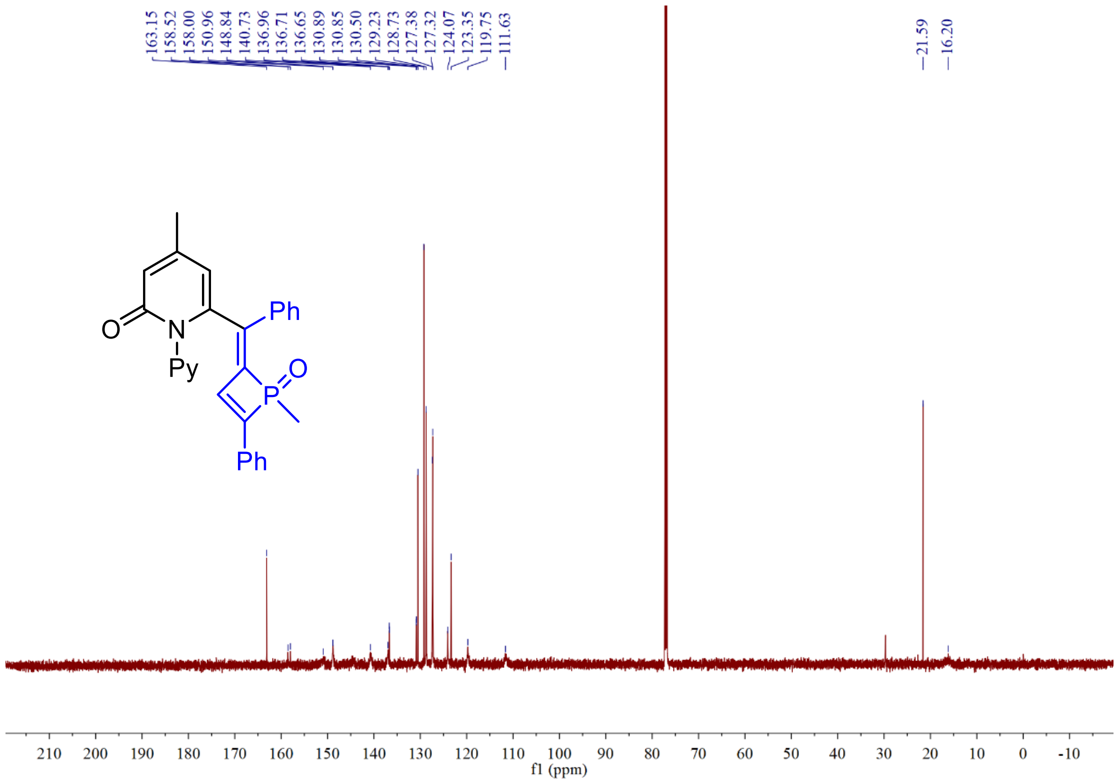

^{13}C NMR spectrum of compound **6h**

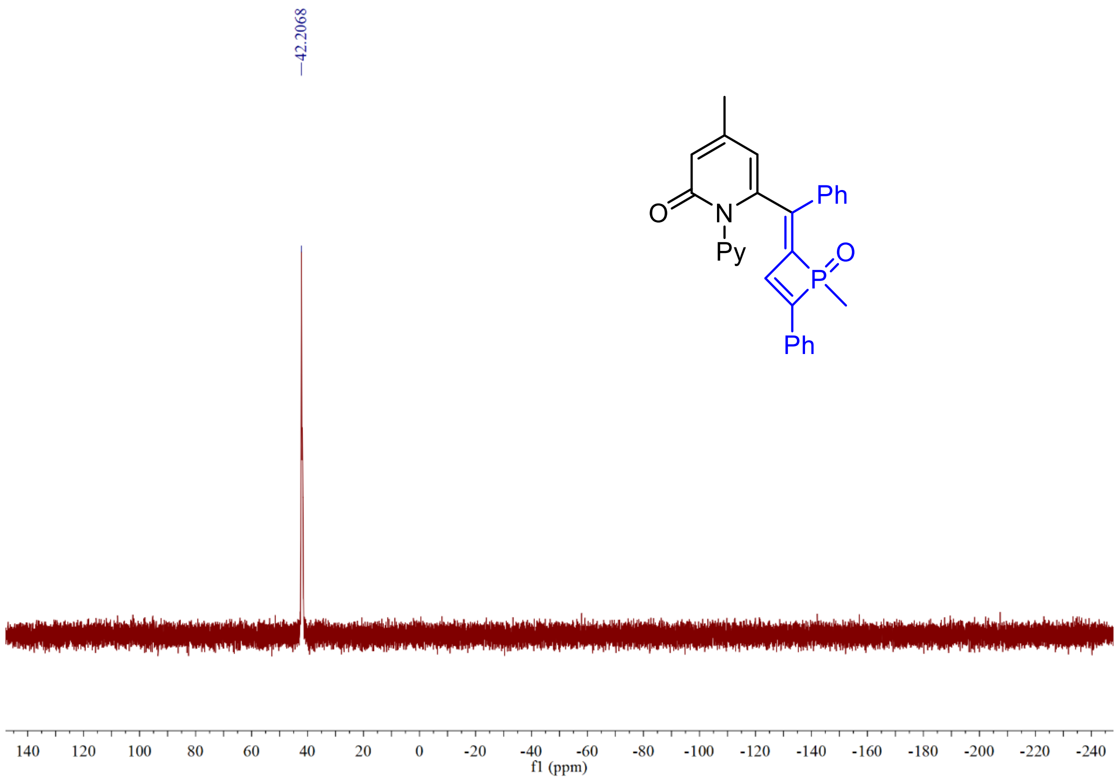

^{31}P NMR spectrum of compound **6h**

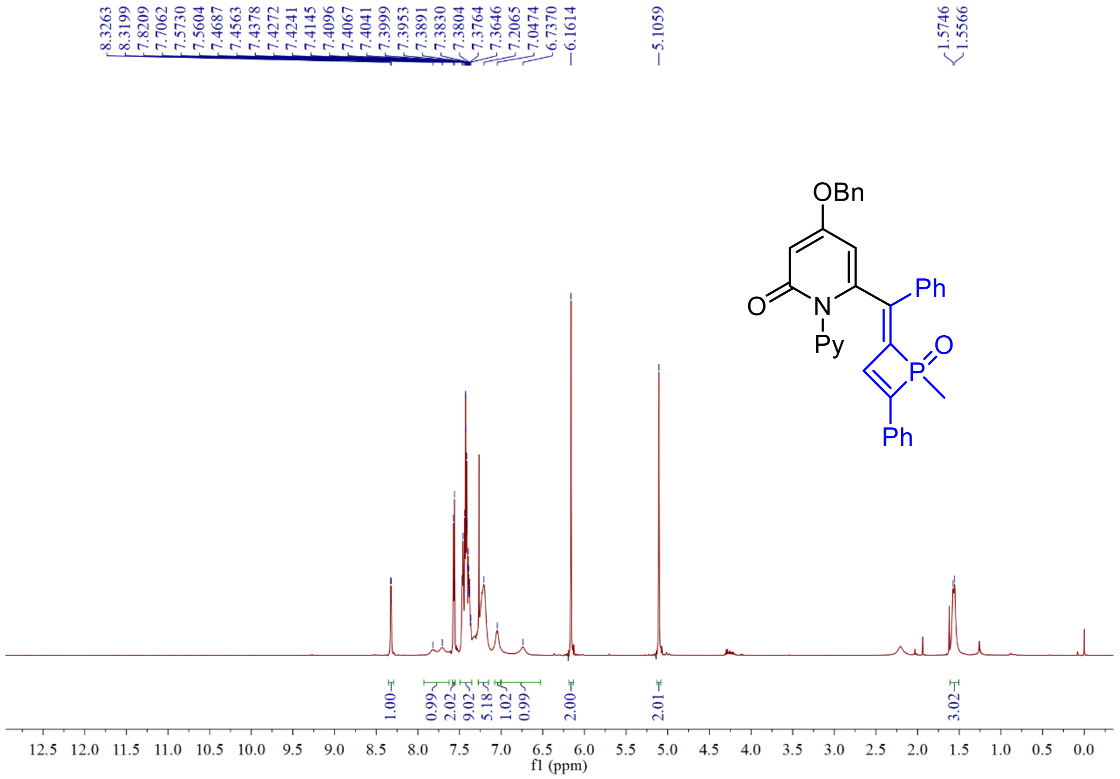

^{1}H NMR spectrum of compound **6i**

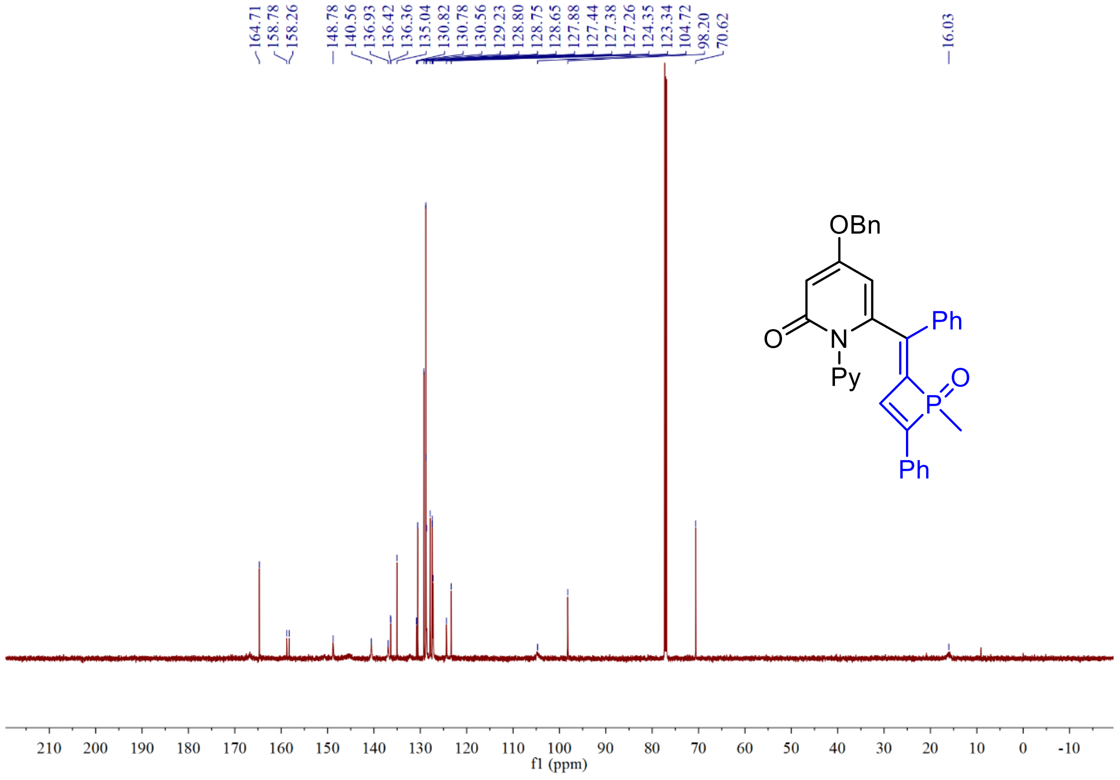

^{13}C NMR spectrum of compound **6i**

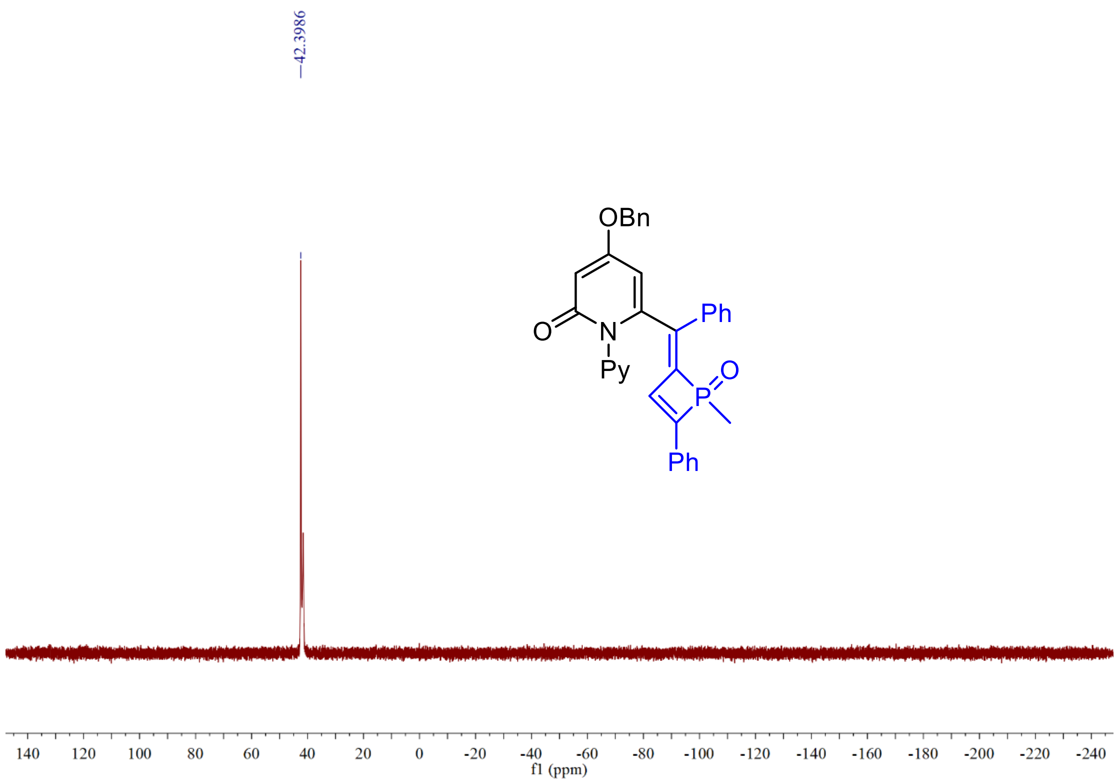

^{31}P NMR spectrum of compound **6i**

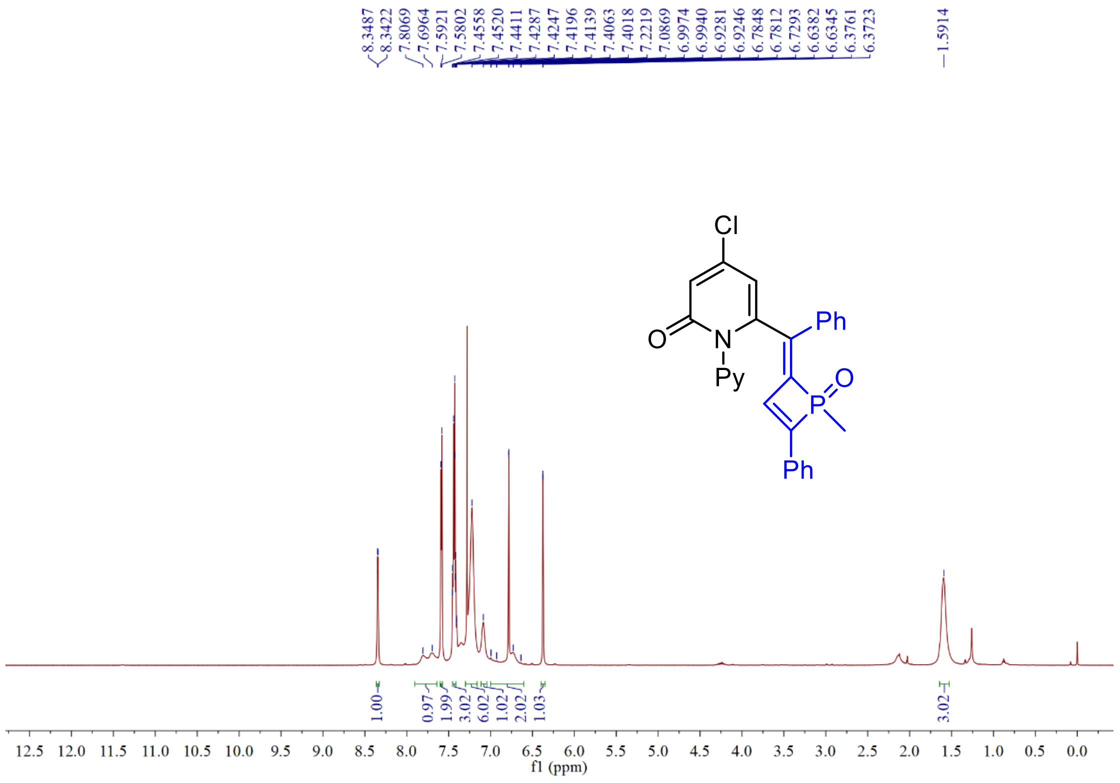

^{1}H NMR spectrum of compound **6j**

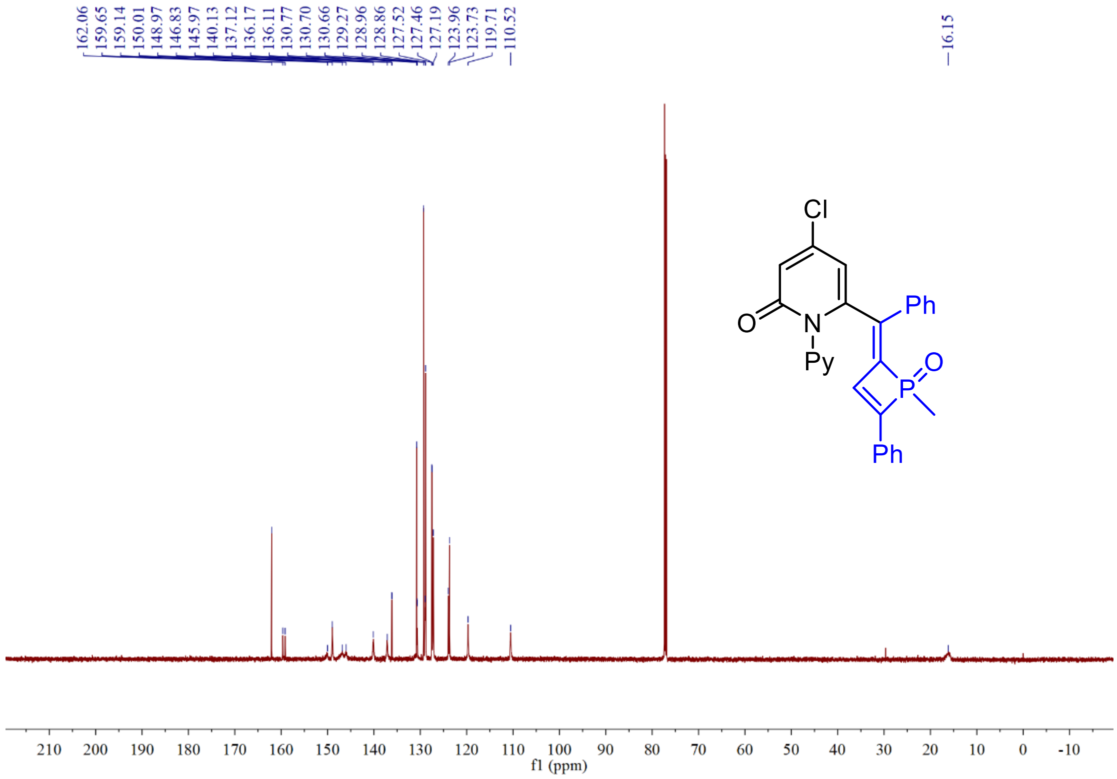

^{13}C NMR spectrum of compound **6j**

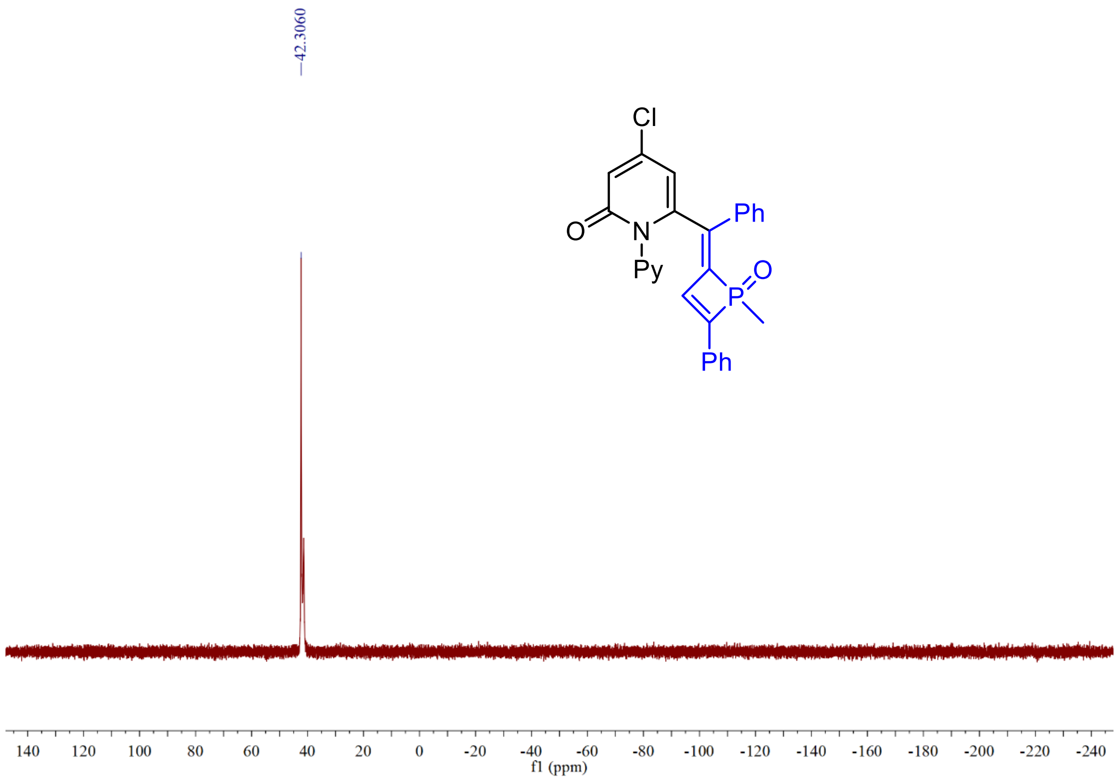

^{31}P NMR spectrum of compound **6j**

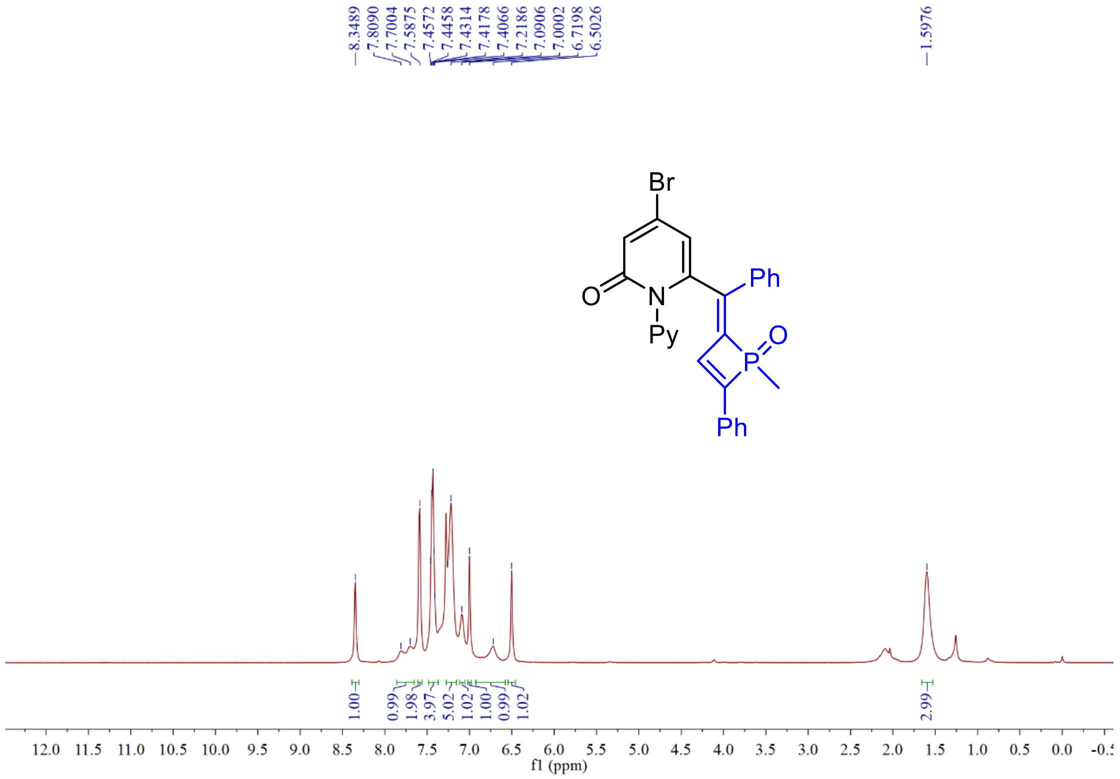

^{1}H NMR spectrum of compound **6k**

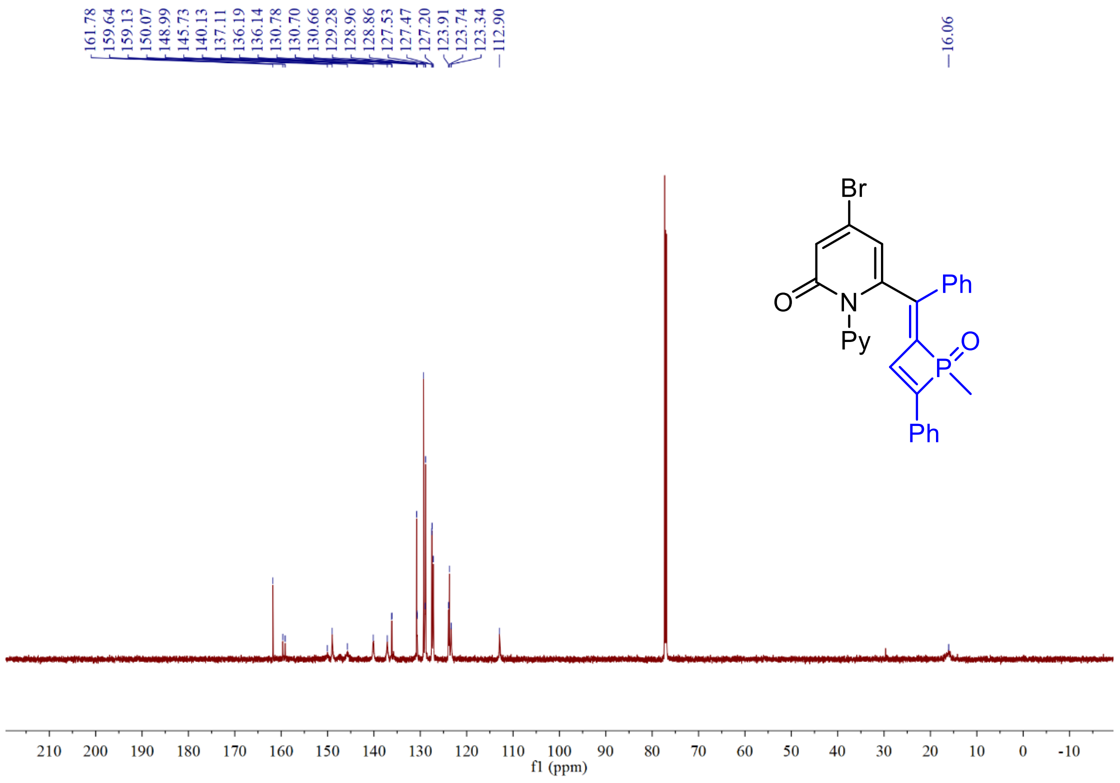

^{13}C NMR spectrum of compound **6k**

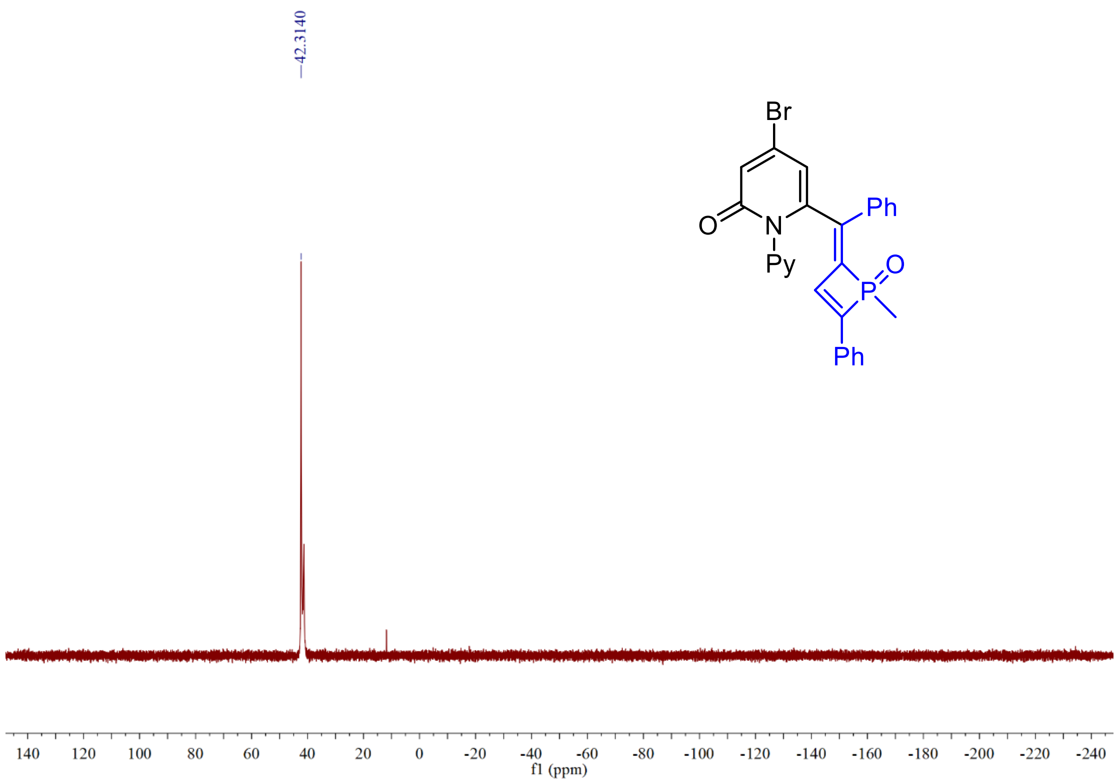

^{31}P NMR spectrum of compound **6k**

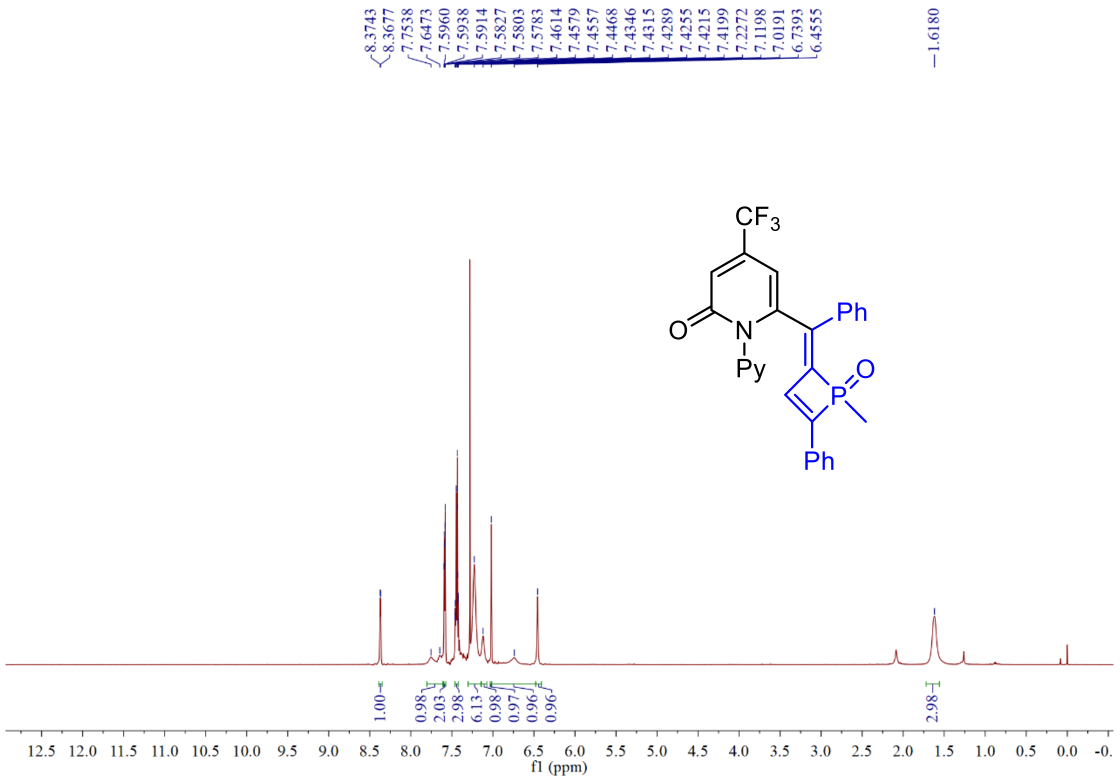

^{1}H NMR spectrum of compound **6l**

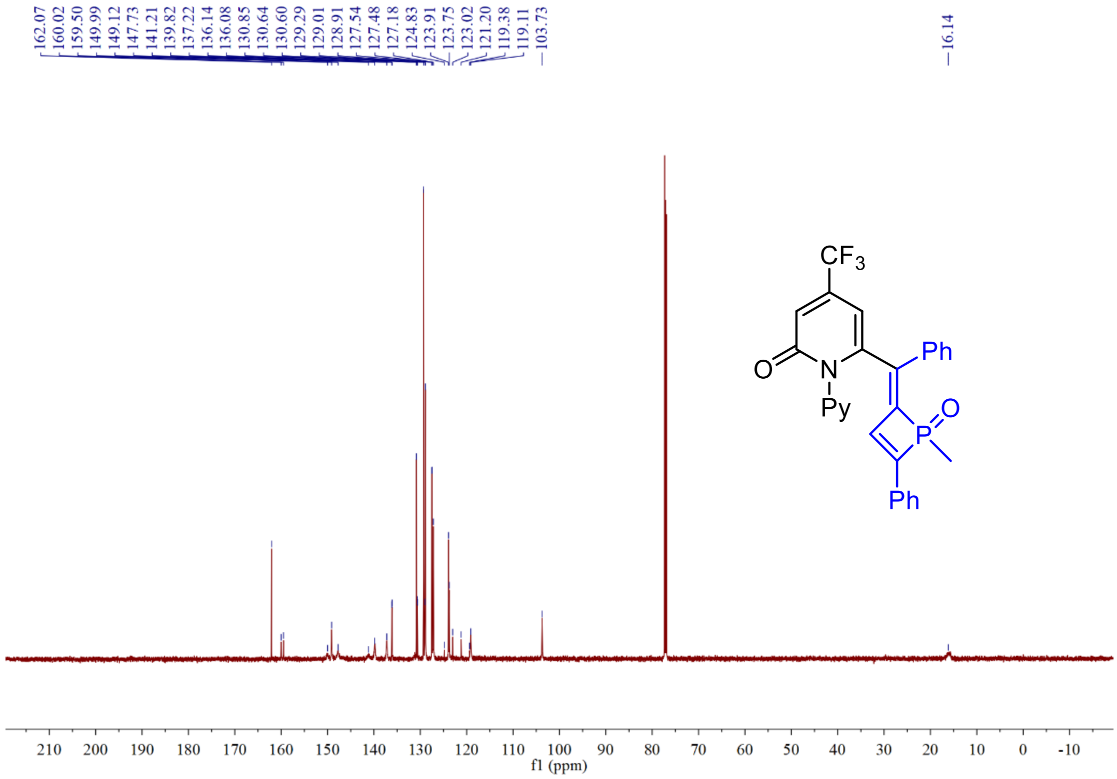

^{13}C NMR spectrum of compound **6l**

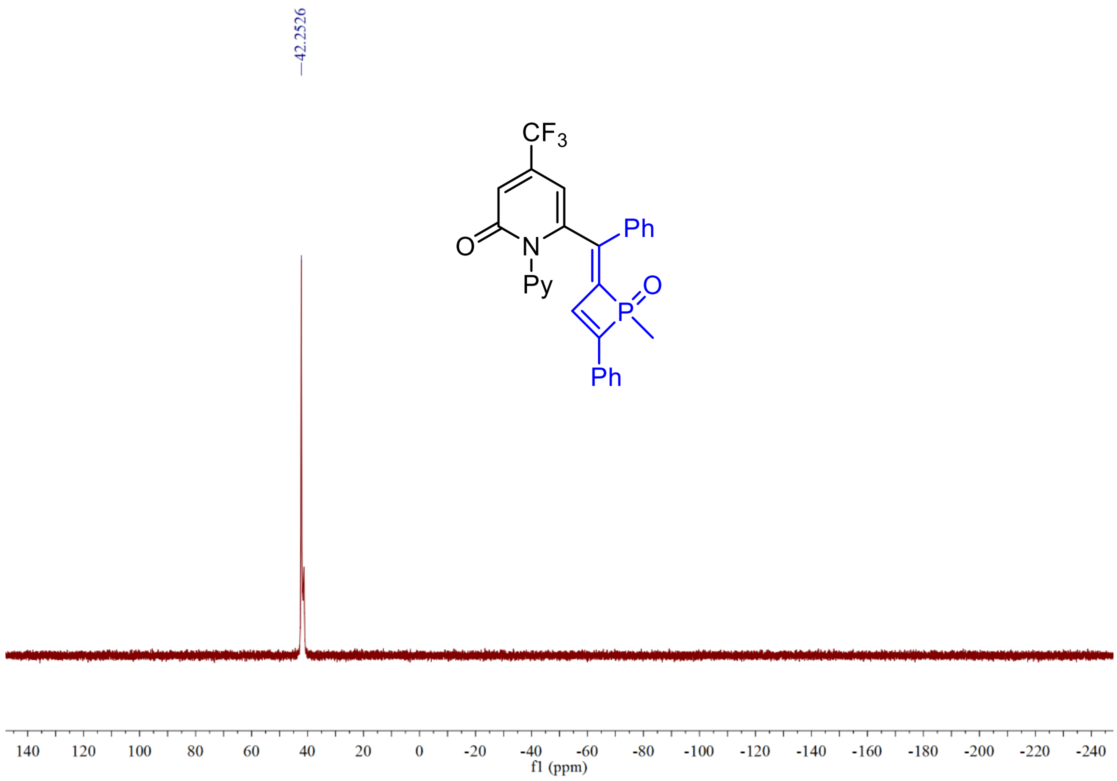

^{31}P NMR spectrum of compound **6l**

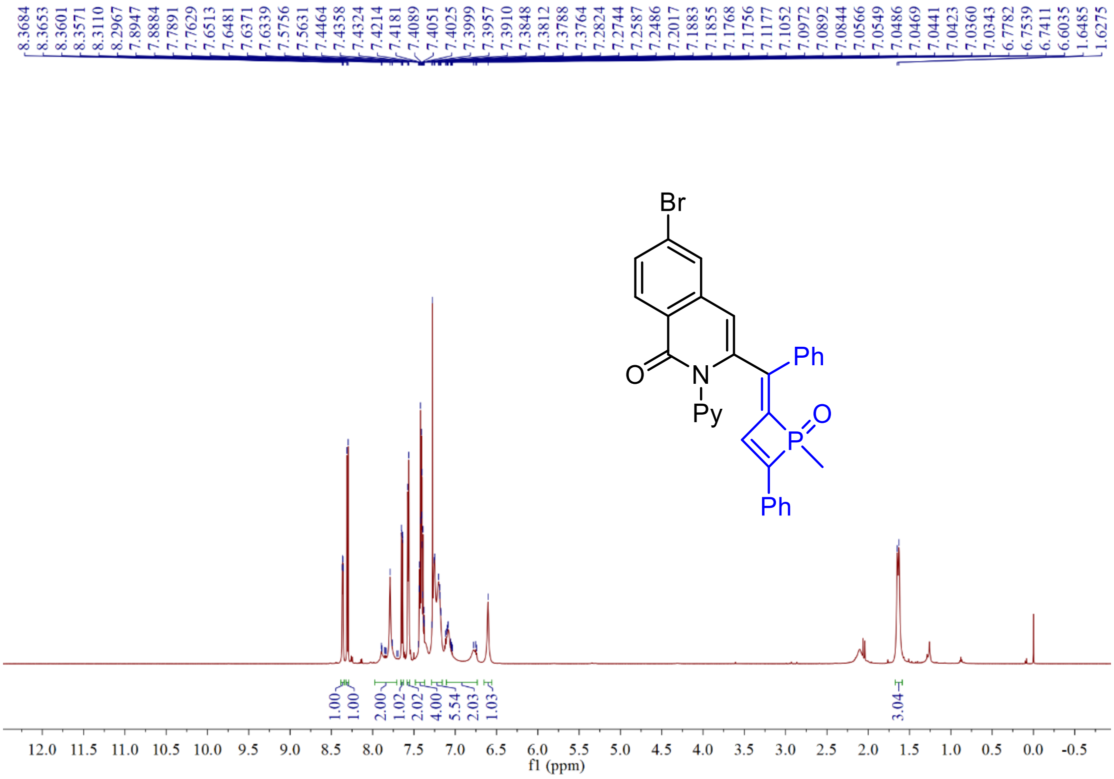

^{1}H NMR spectrum of compound **6m**

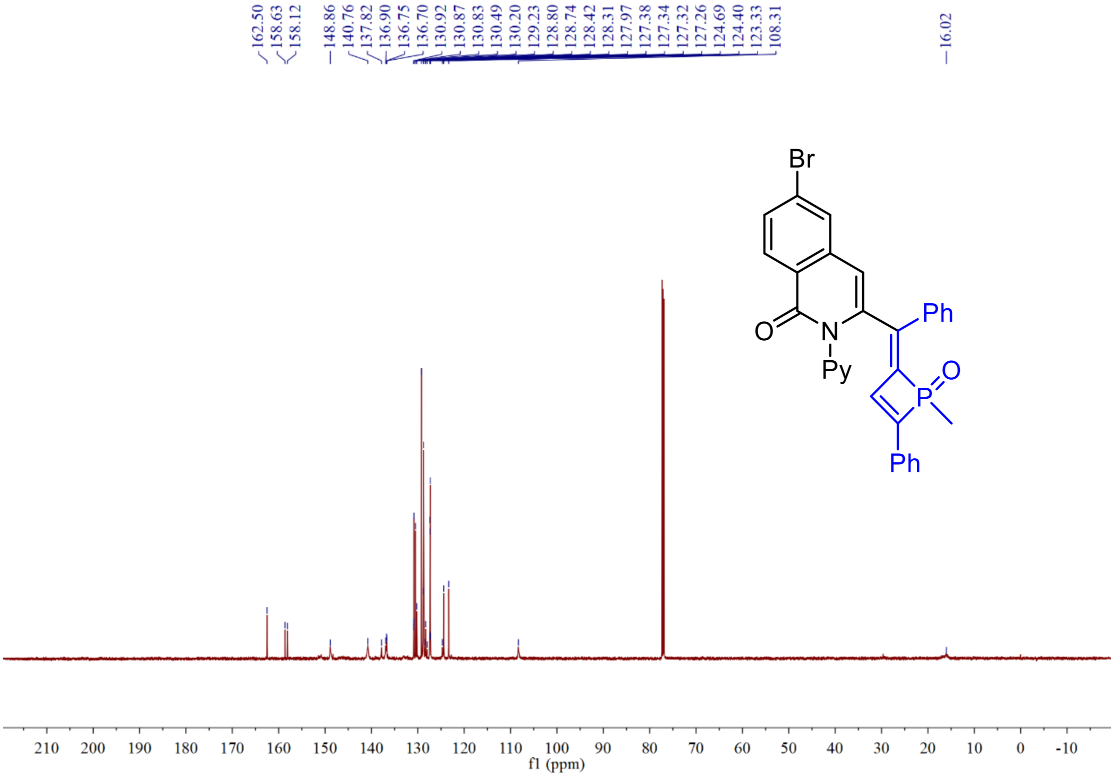

^{13}C NMR spectrum of compound **6m**

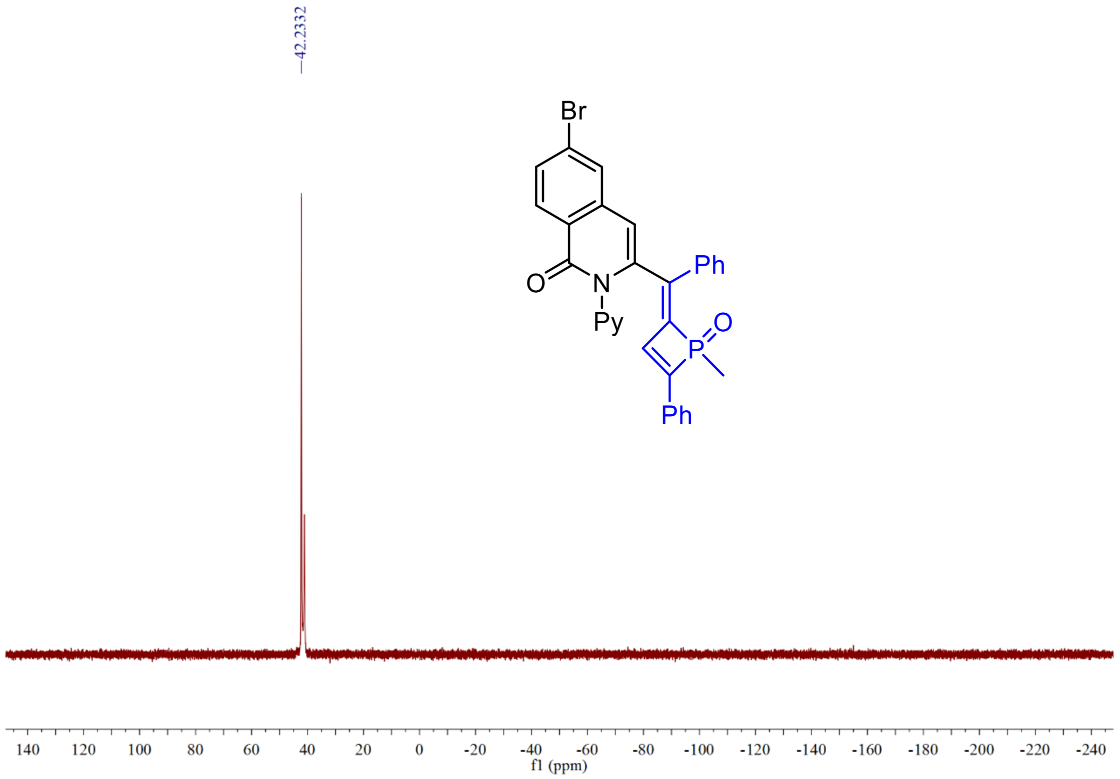

^{31}P NMR spectrum of compound **6m**

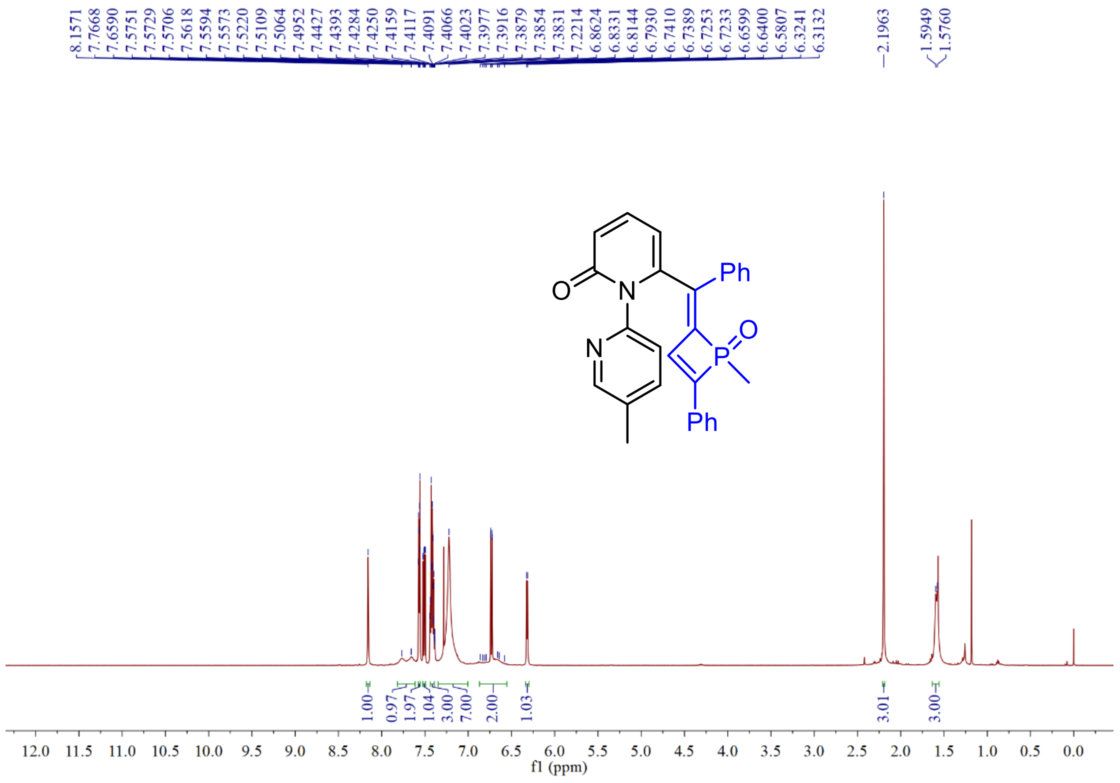

^{1}H NMR spectrum of compound **6n**

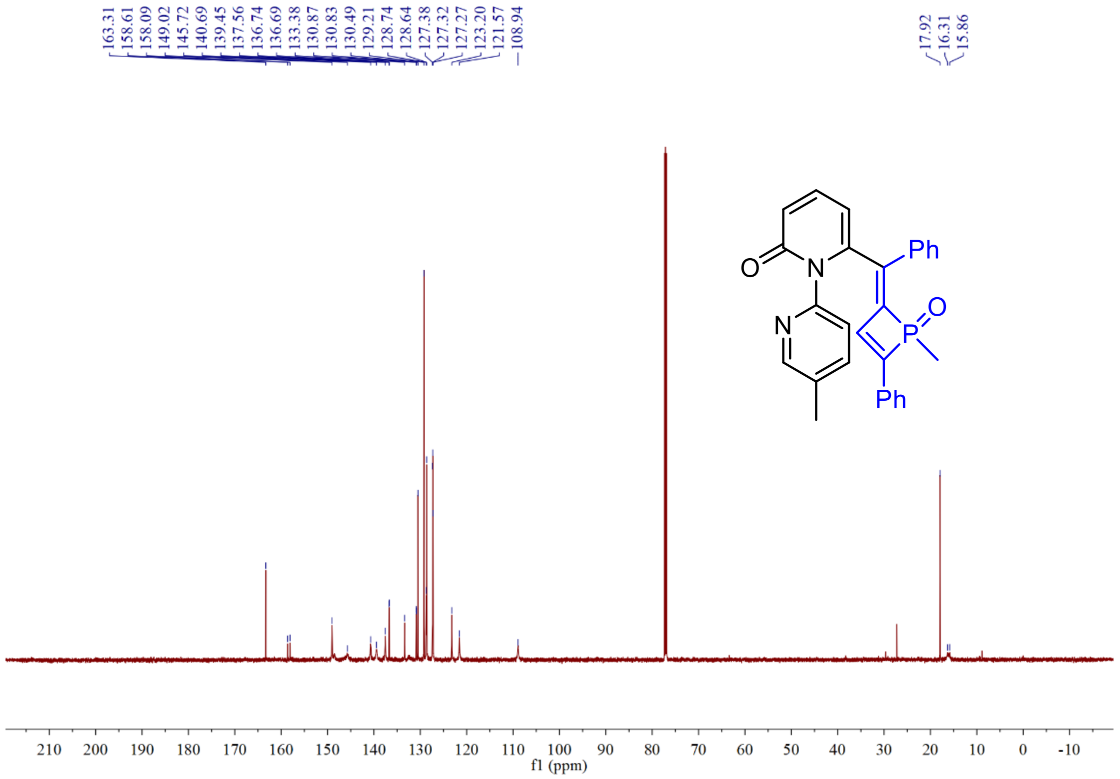

^{13}C NMR spectrum of compound **6n**

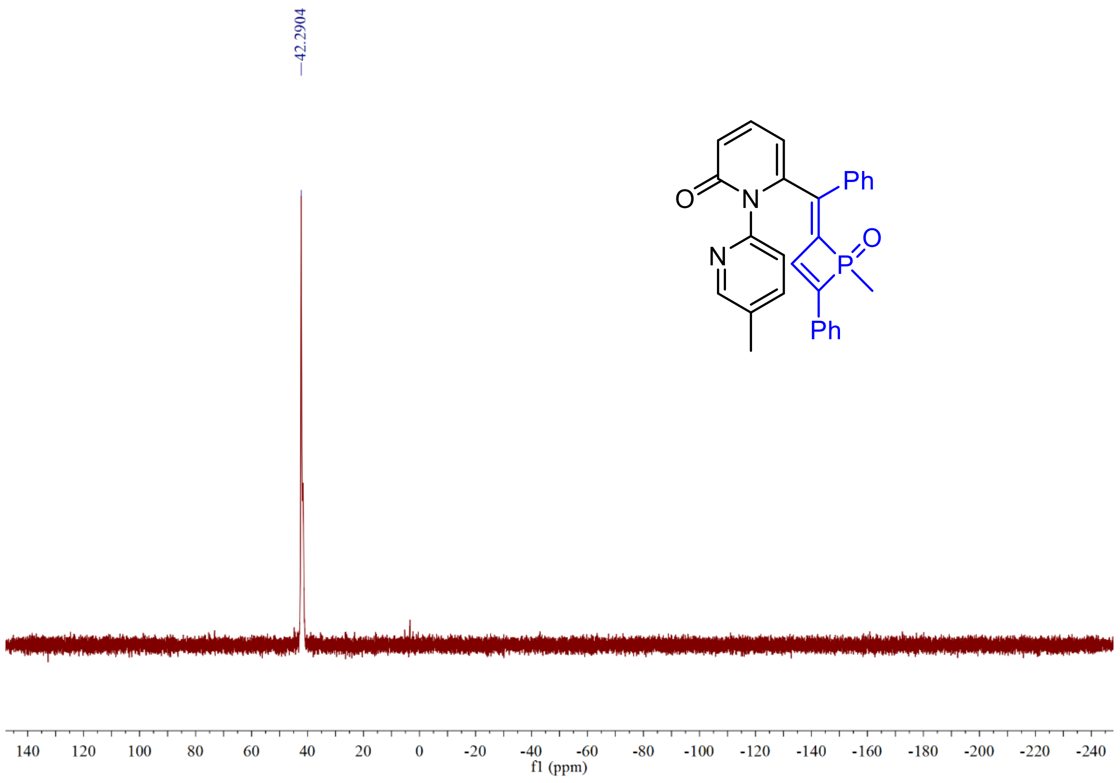

^{31}P NMR spectrum of compound **6n**

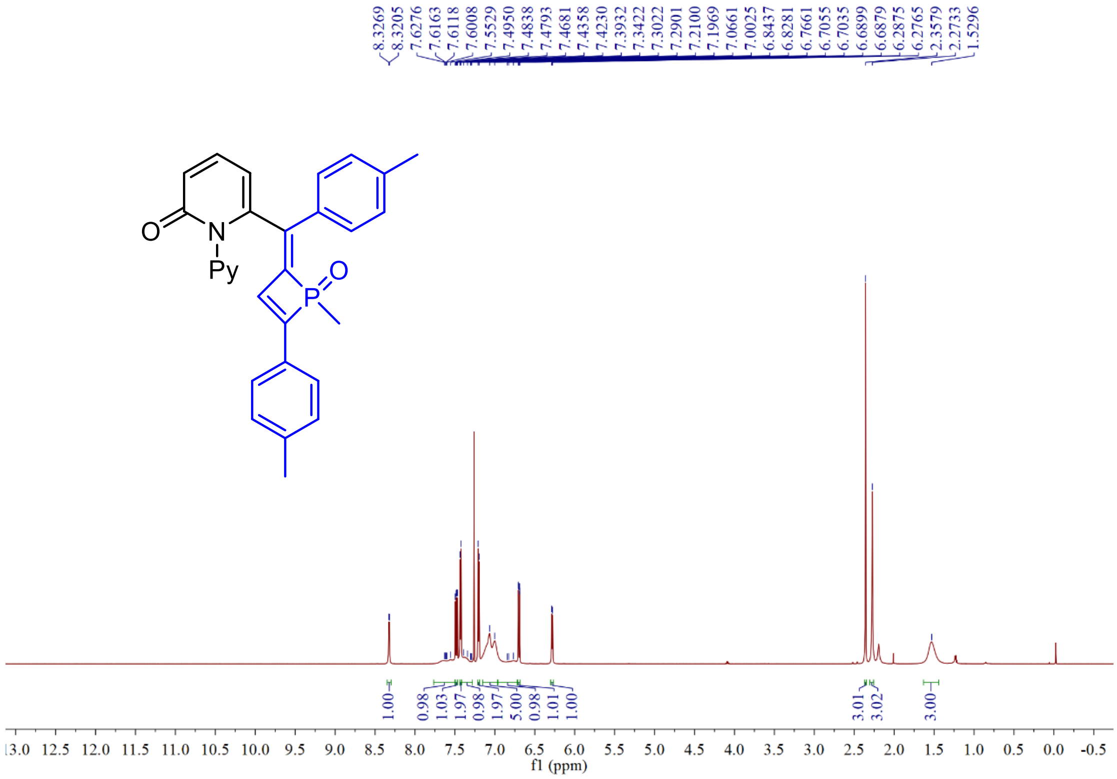

^{1}H NMR spectrum of compound **6o**

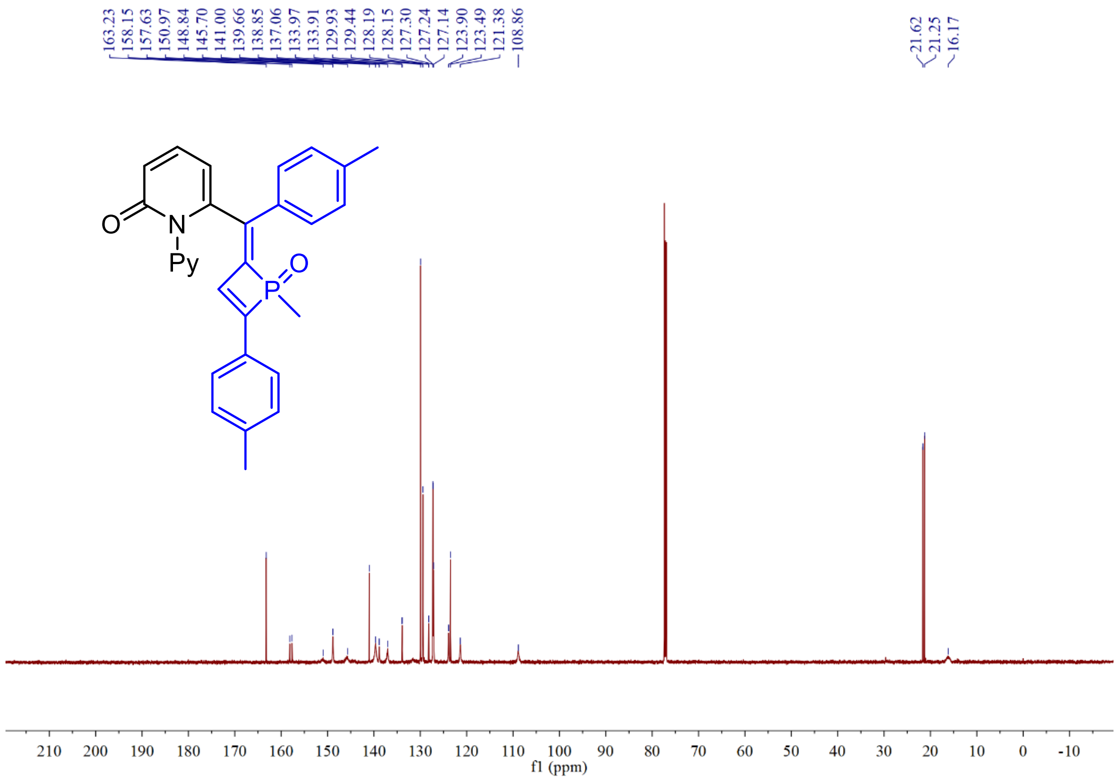

^{13}C NMR spectrum of compound **6o**

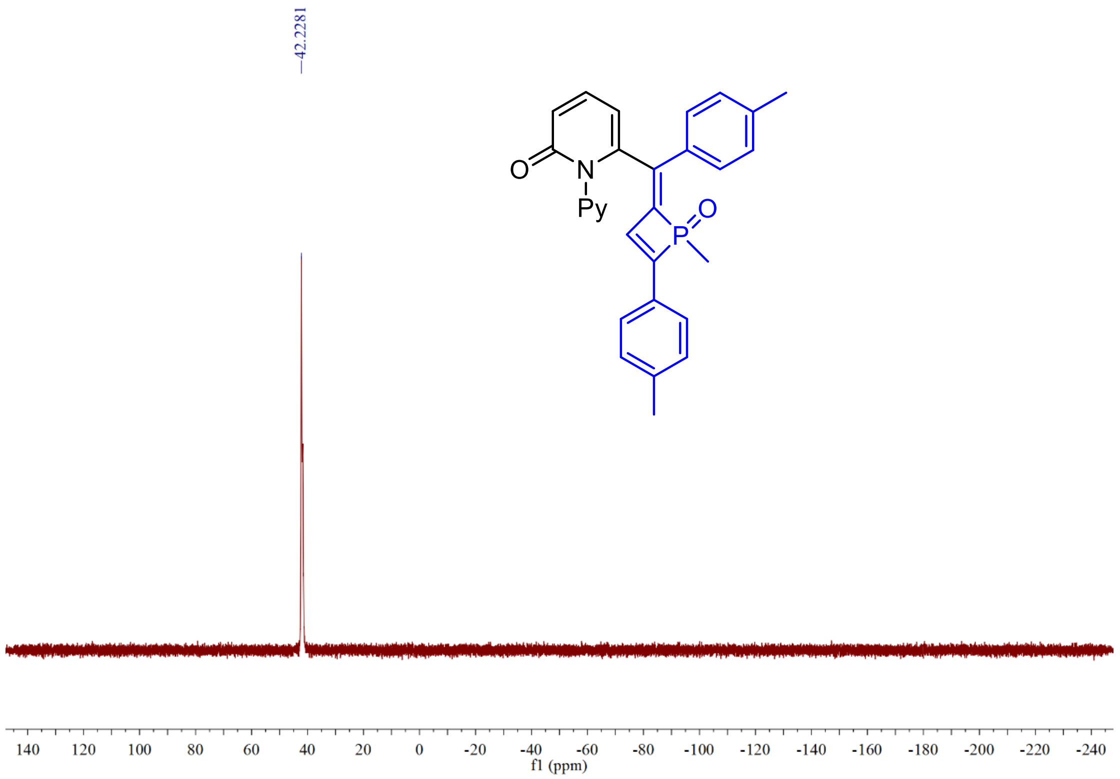

^{31}P NMR spectrum of compound **6o**

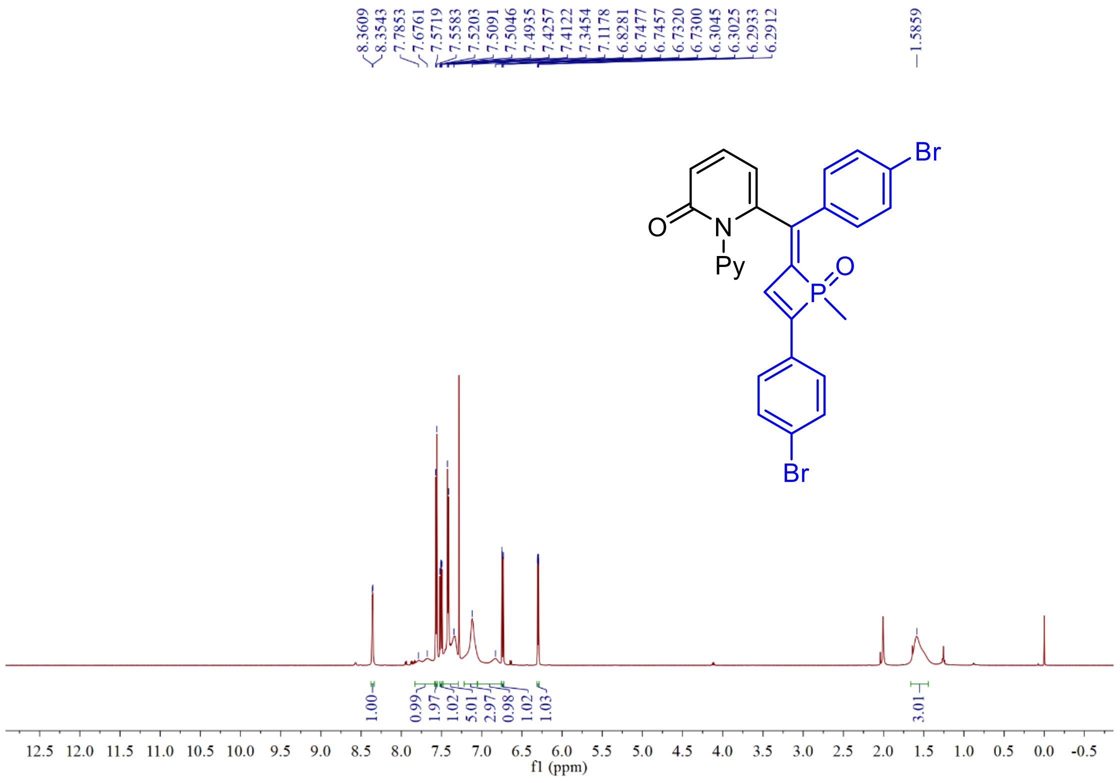

^{1}H NMR spectrum of compound **6p**

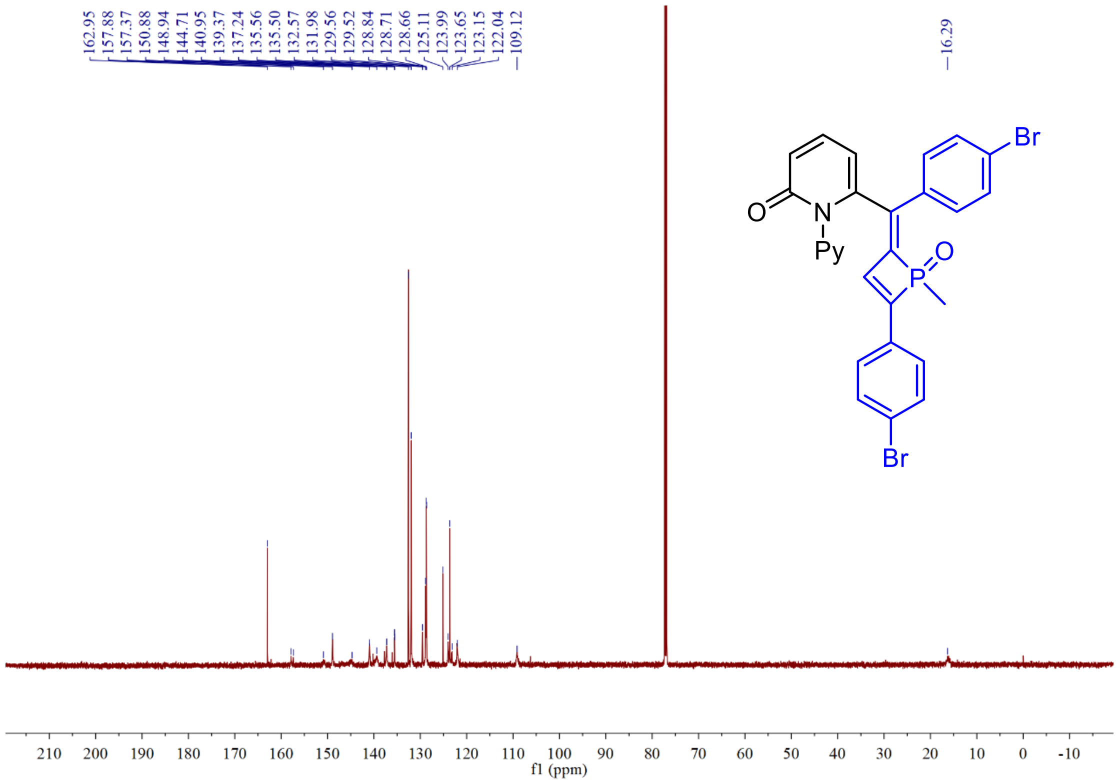

^{13}C NMR spectrum of compound **6p**

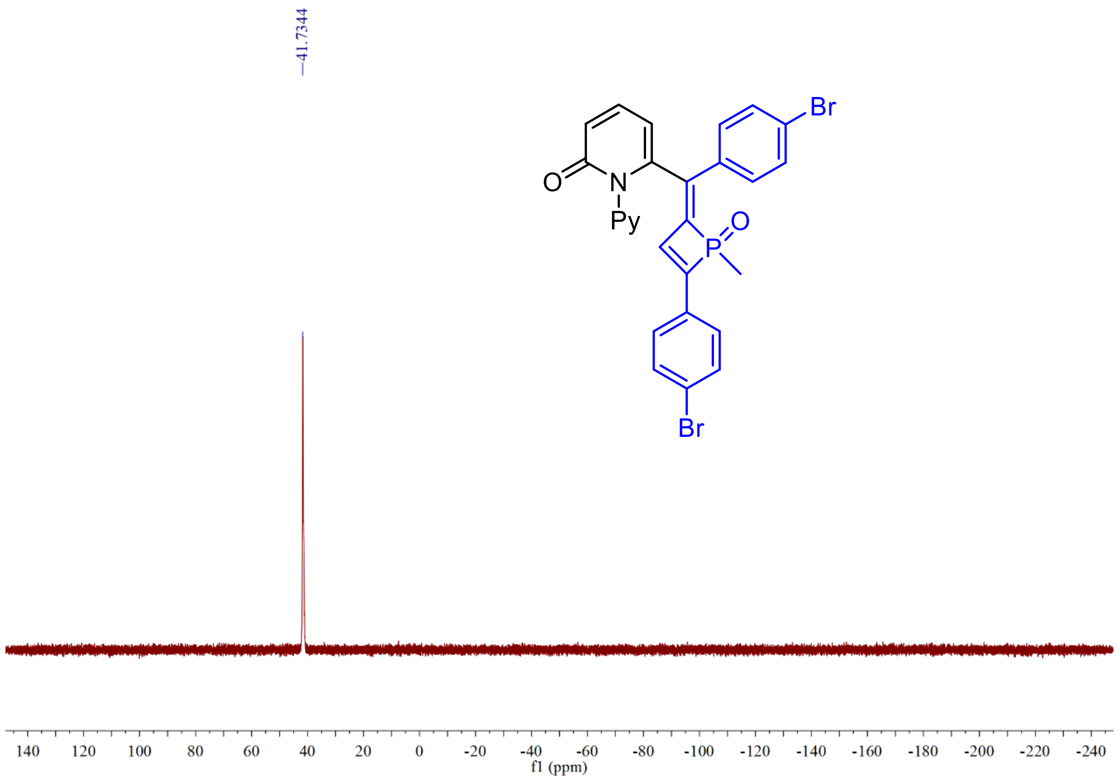

^{31}P NMR spectrum of compound **6p**

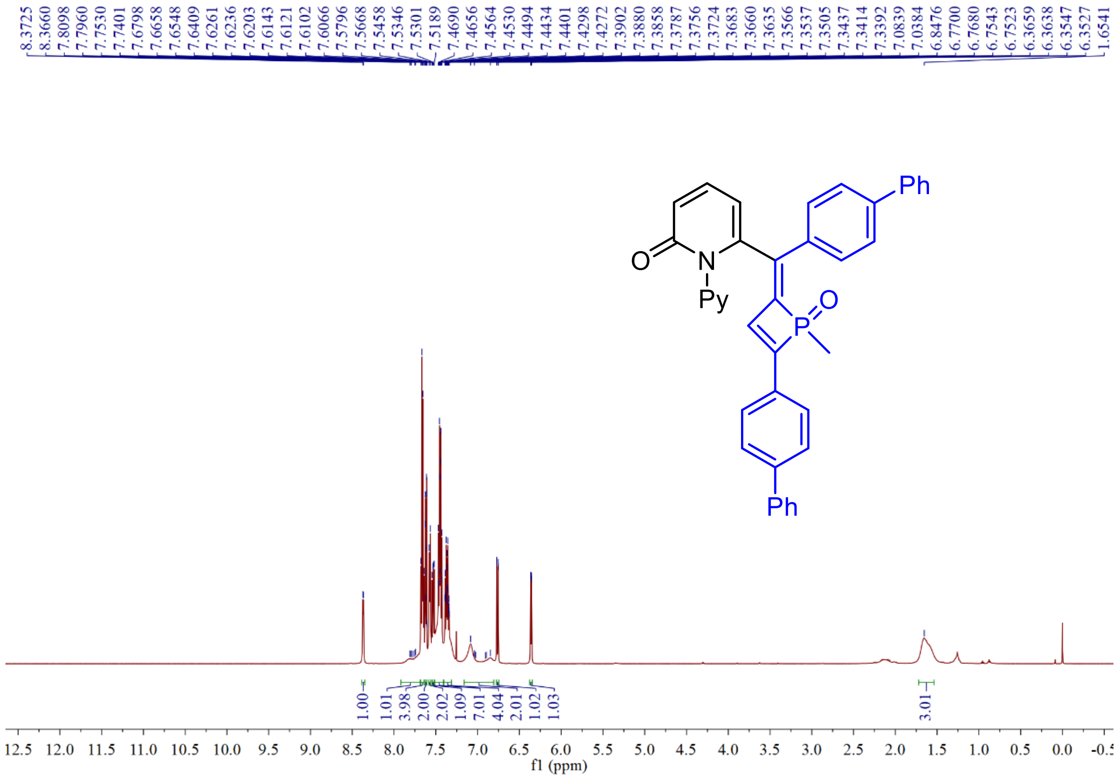

^{1}H NMR spectrum of compound **6q**

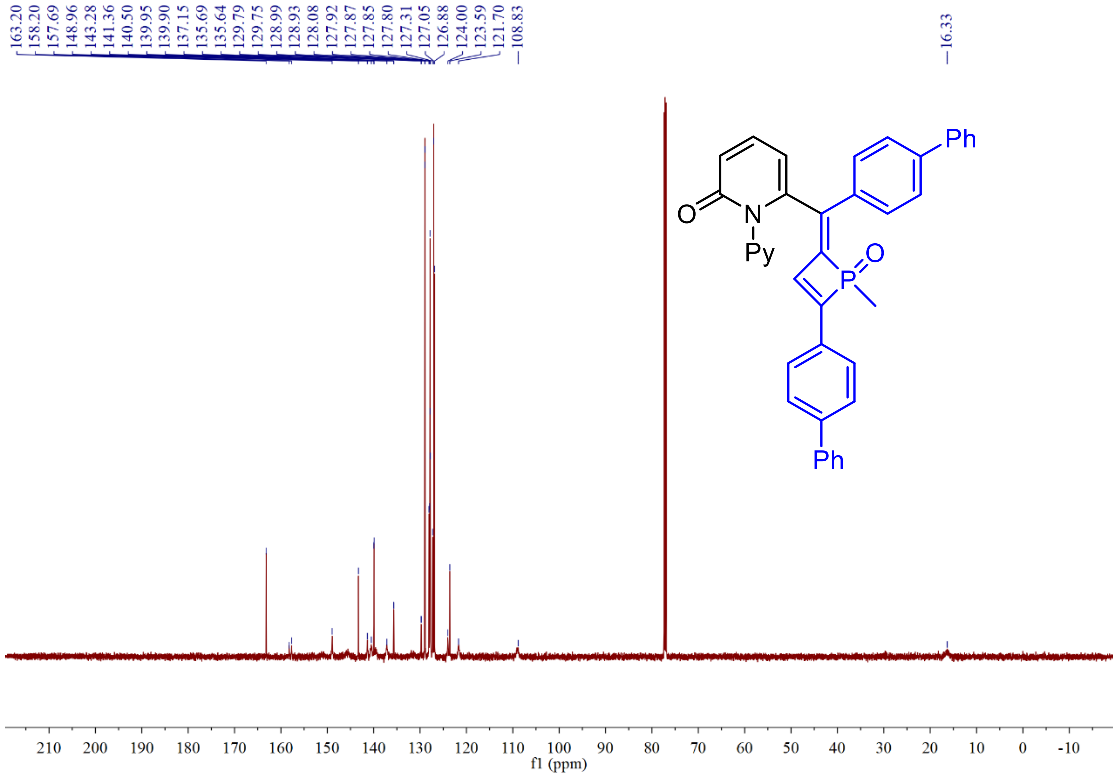

^{13}C NMR spectrum of compound **6q**

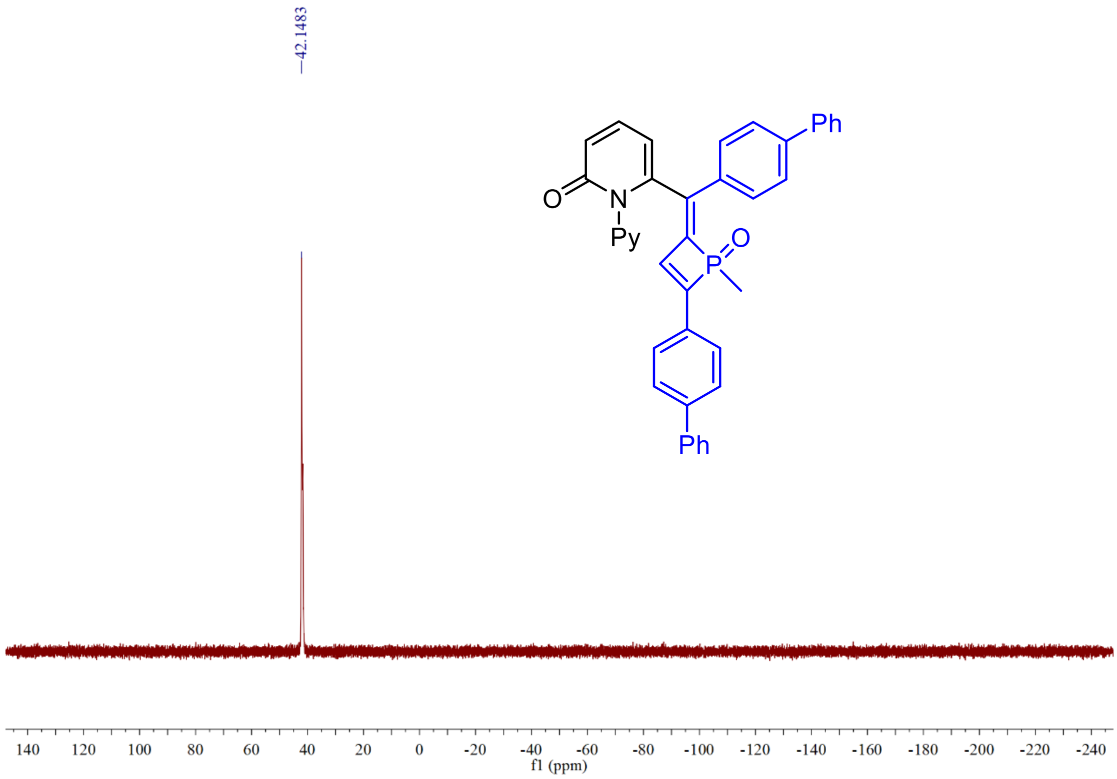

^{31}P NMR spectrum of compound **6q**

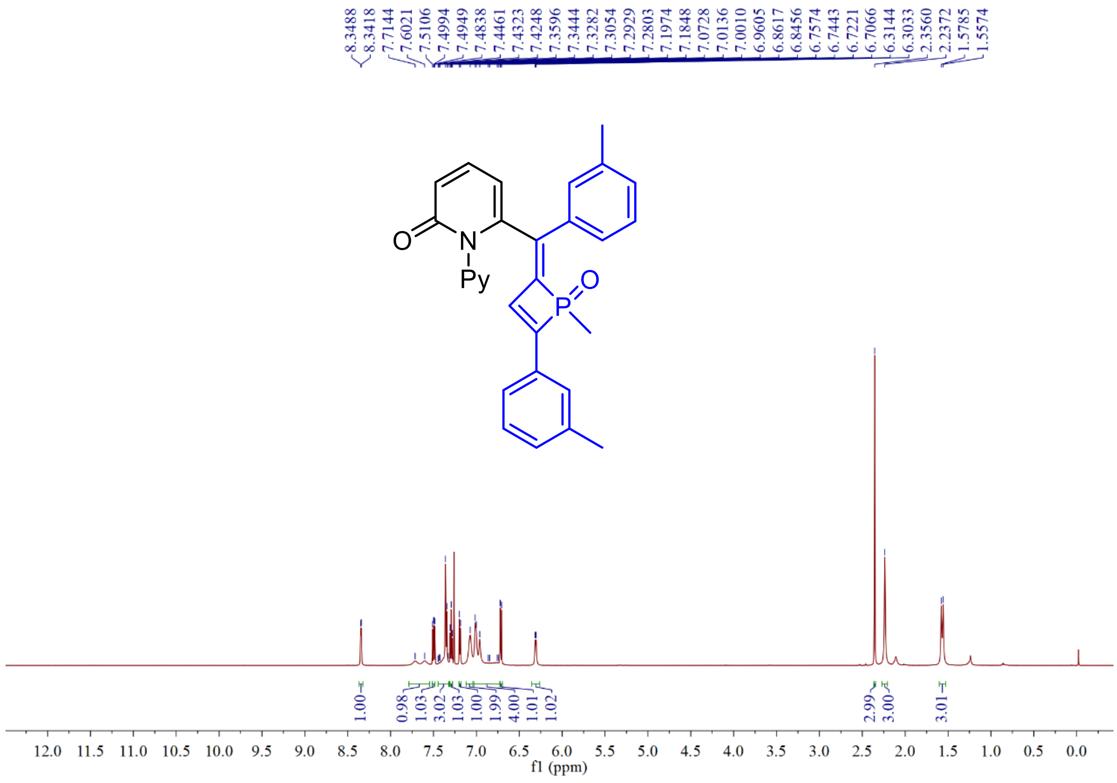

^{1}H NMR spectrum of compound **6r**

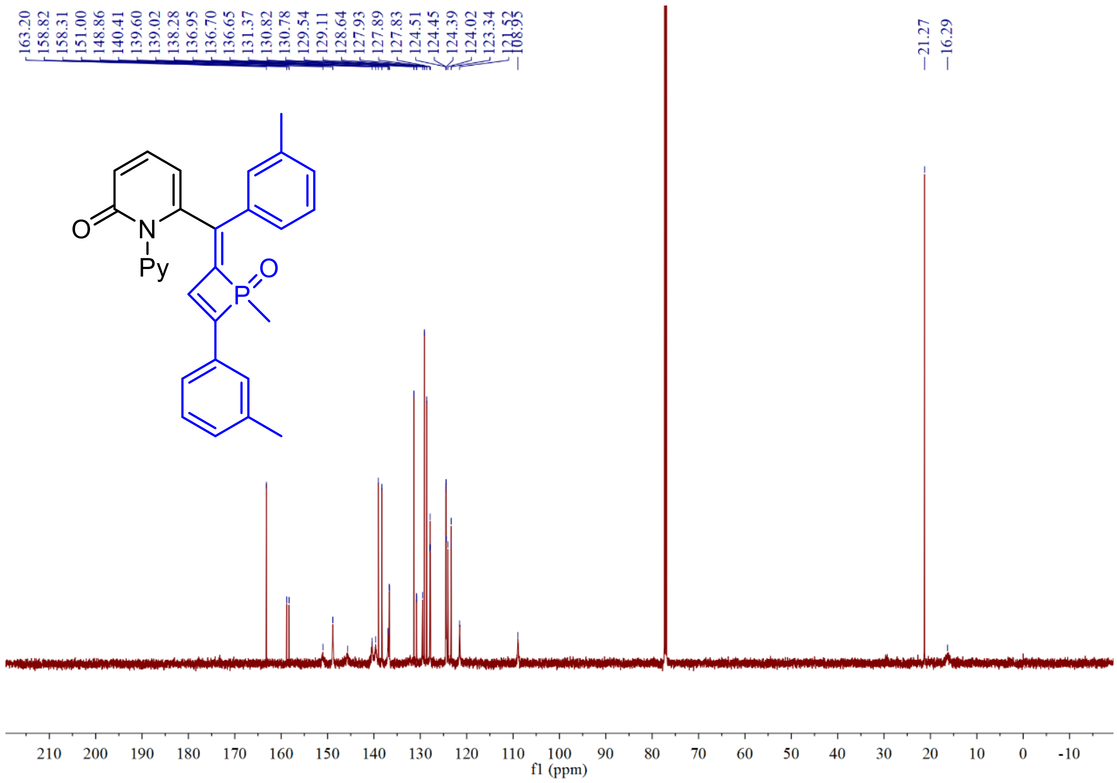

^{13}C NMR spectrum of compound **6r**

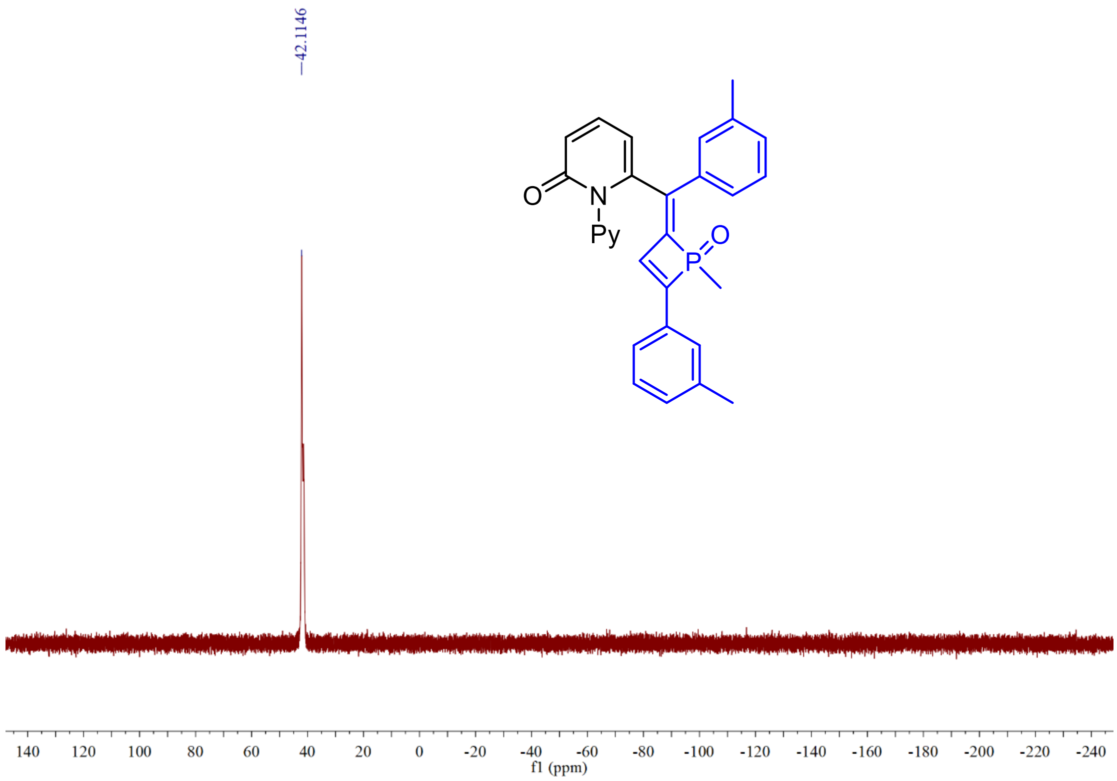

^{31}P NMR spectrum of compound **6r**

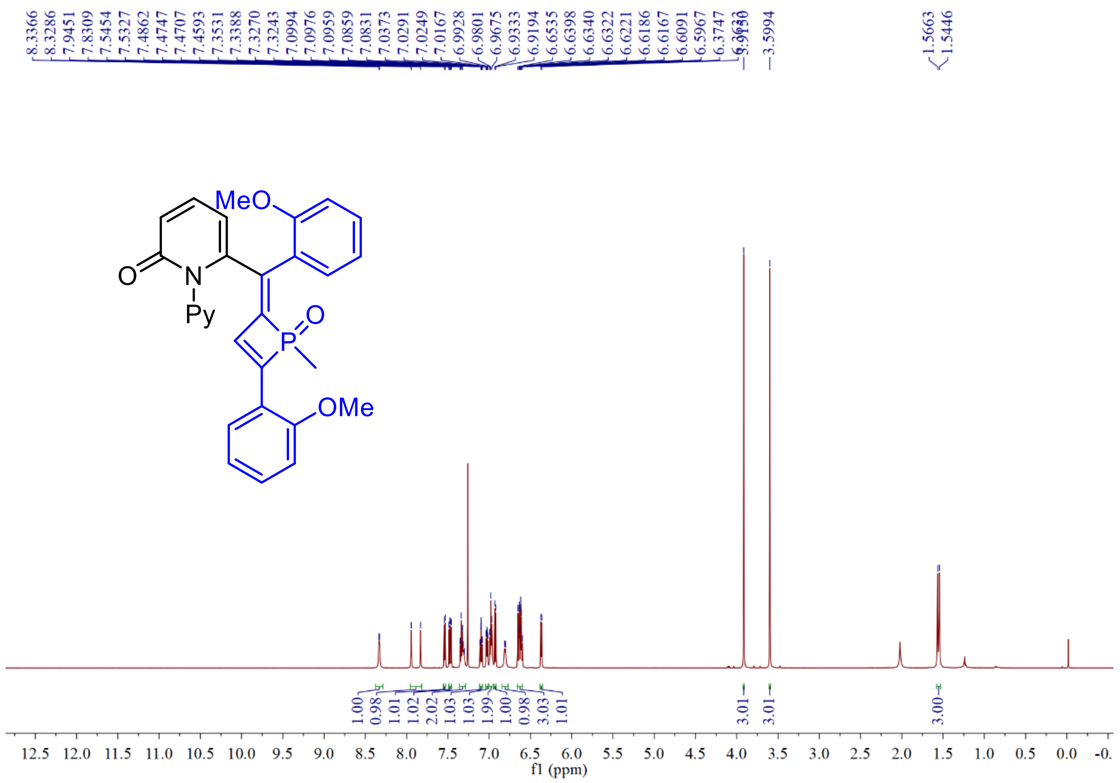

^{1}H NMR spectrum of compound **6s**

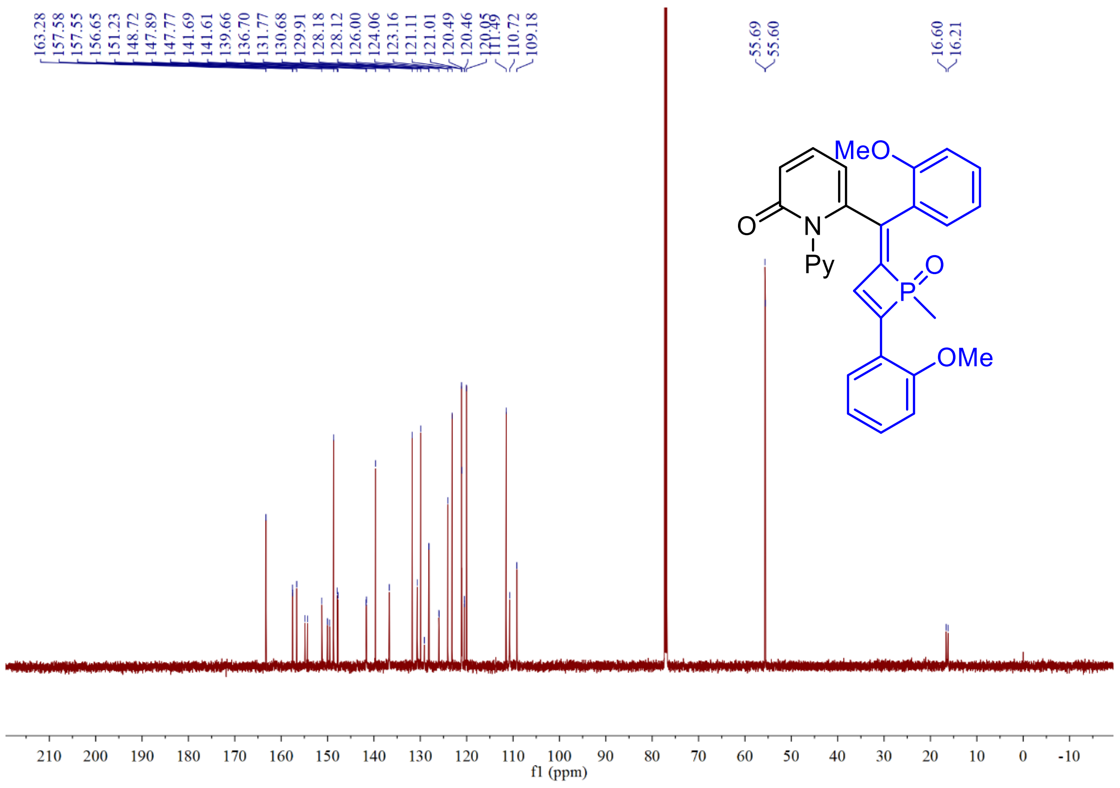

^{13}C NMR spectrum of compound **6s**

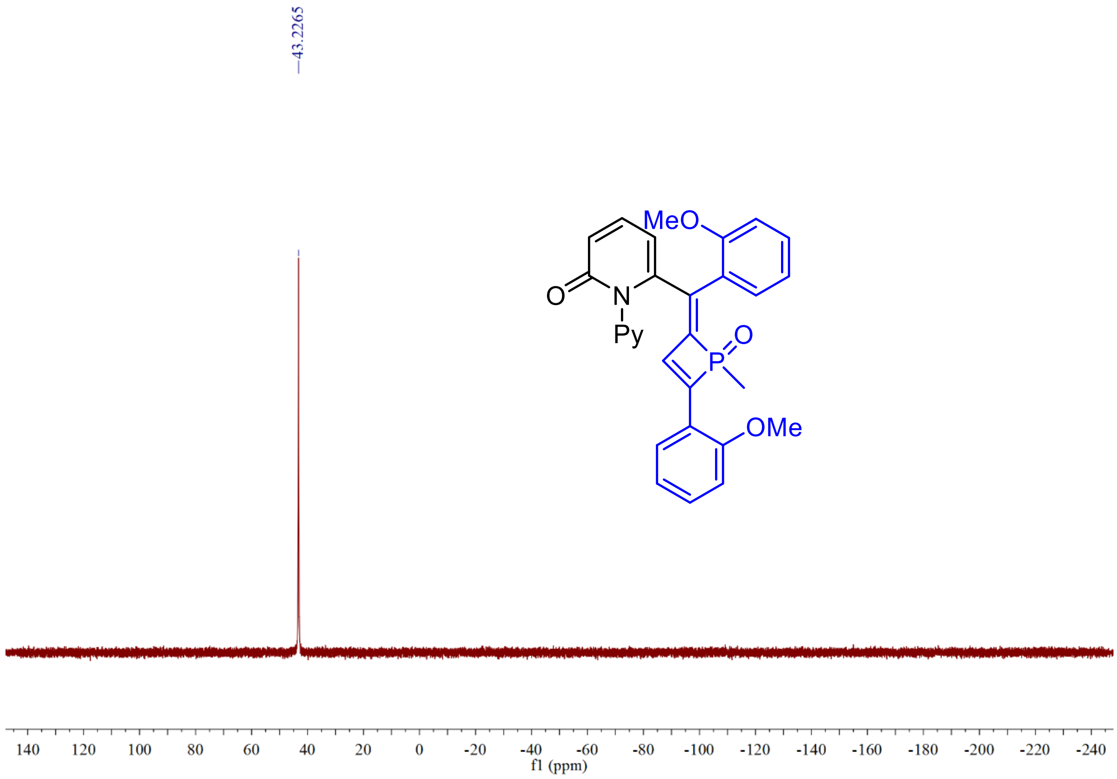

^{31}P NMR spectrum of compound **6s**

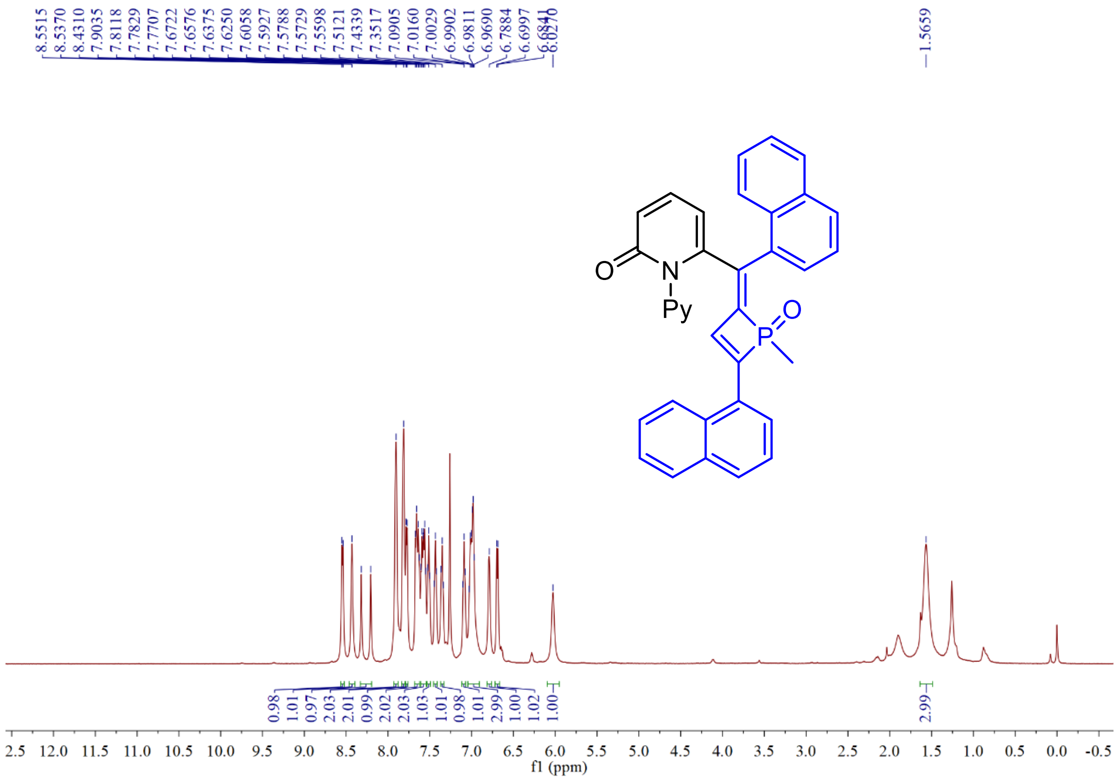

^{1}H NMR spectrum of compound **6t**

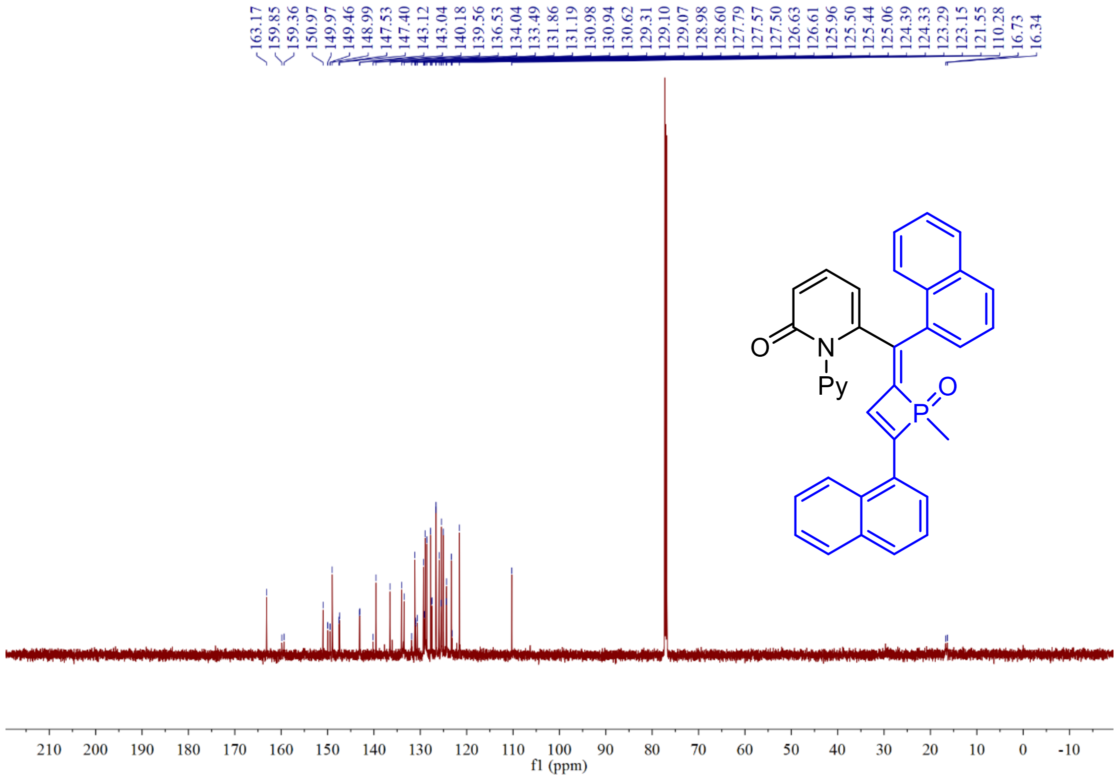

^{13}C NMR spectrum of compound **6t**

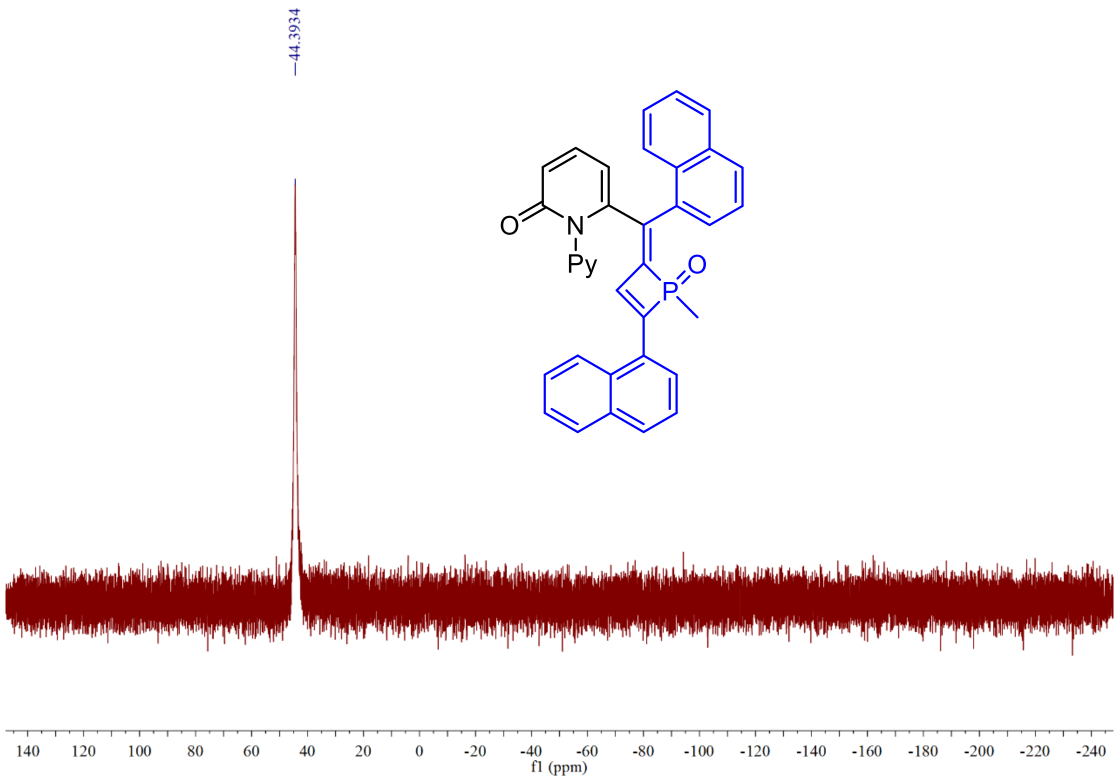

^{31}P NMR spectrum of compound **6t**

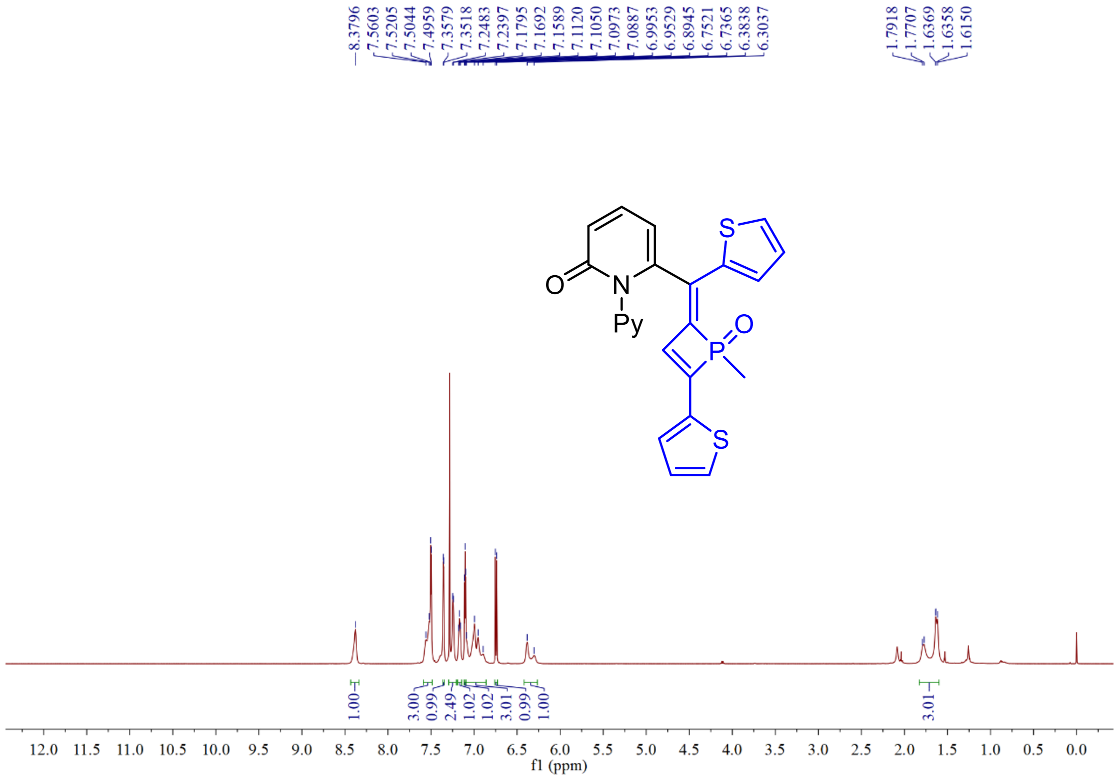

^{1}H NMR spectrum of compound **6u**

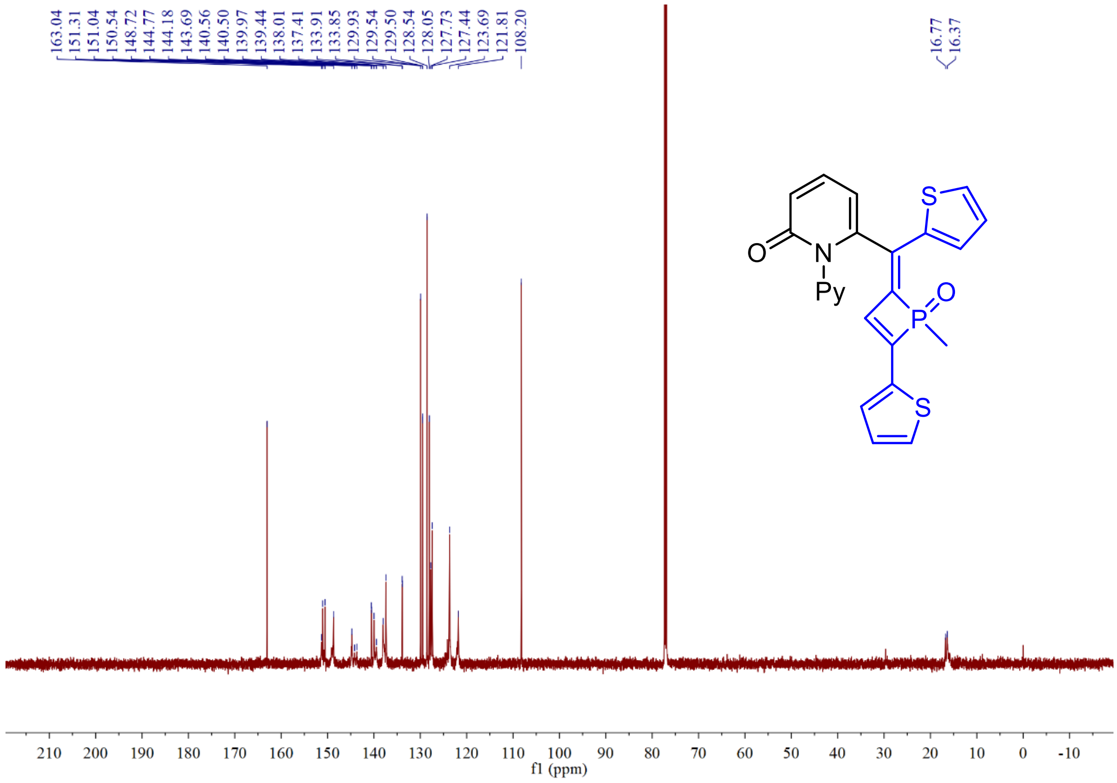

^{13}C NMR spectrum of compound **6u**

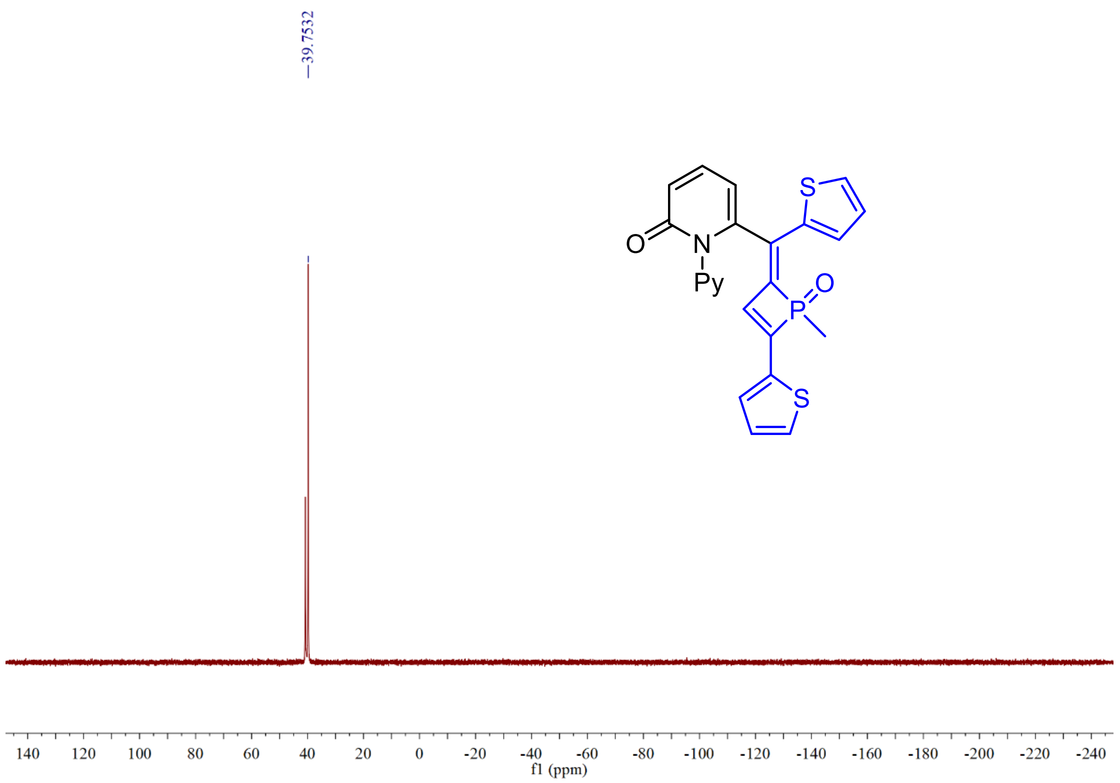

^{31}P NMR spectrum of compound **6u**

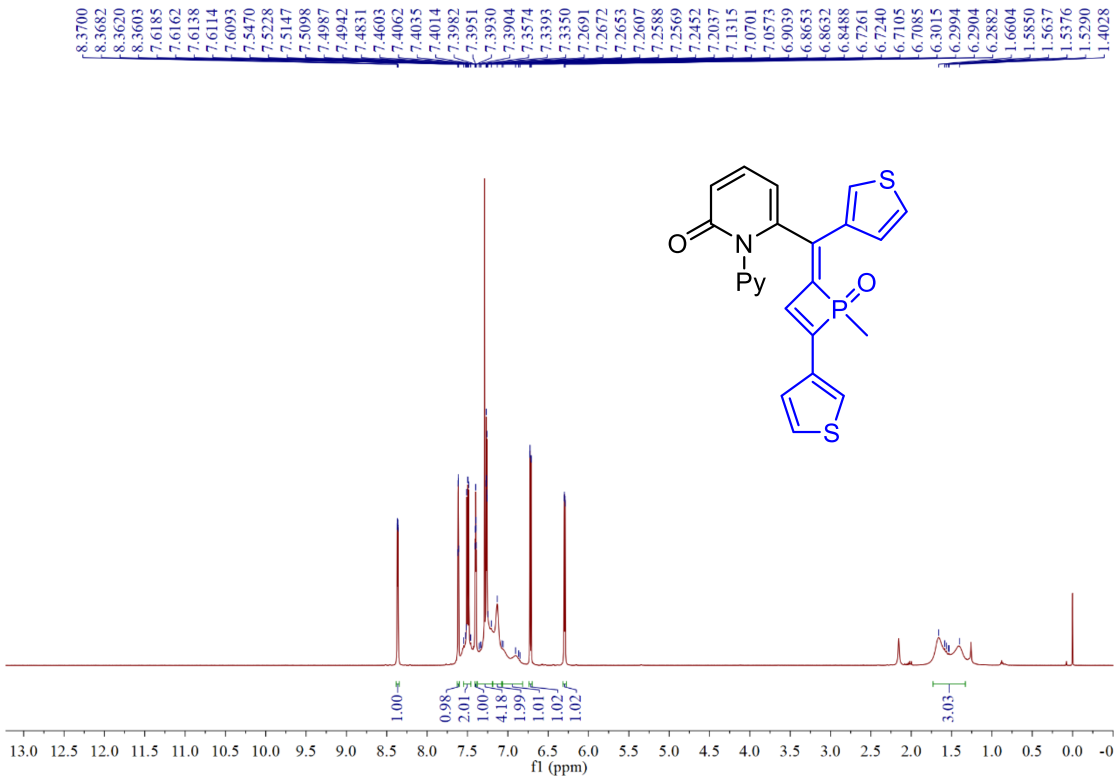

^{1}H NMR spectrum of compound **6v**

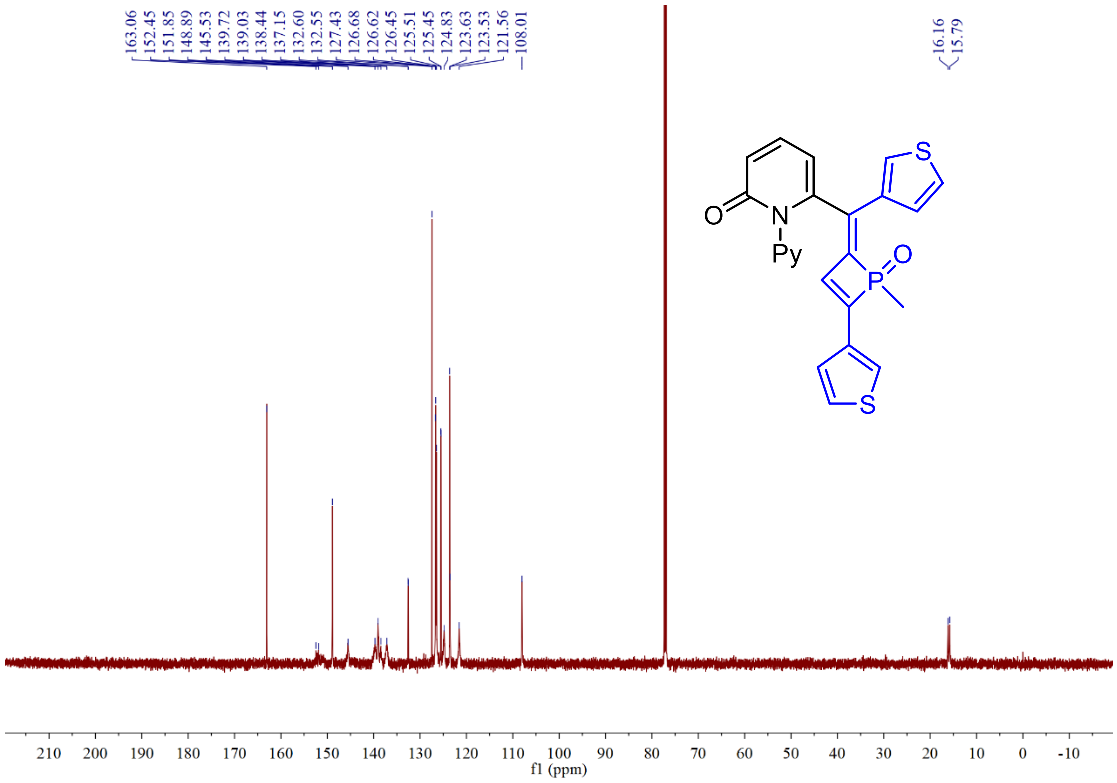

^{13}C NMR spectrum of compound **6v**

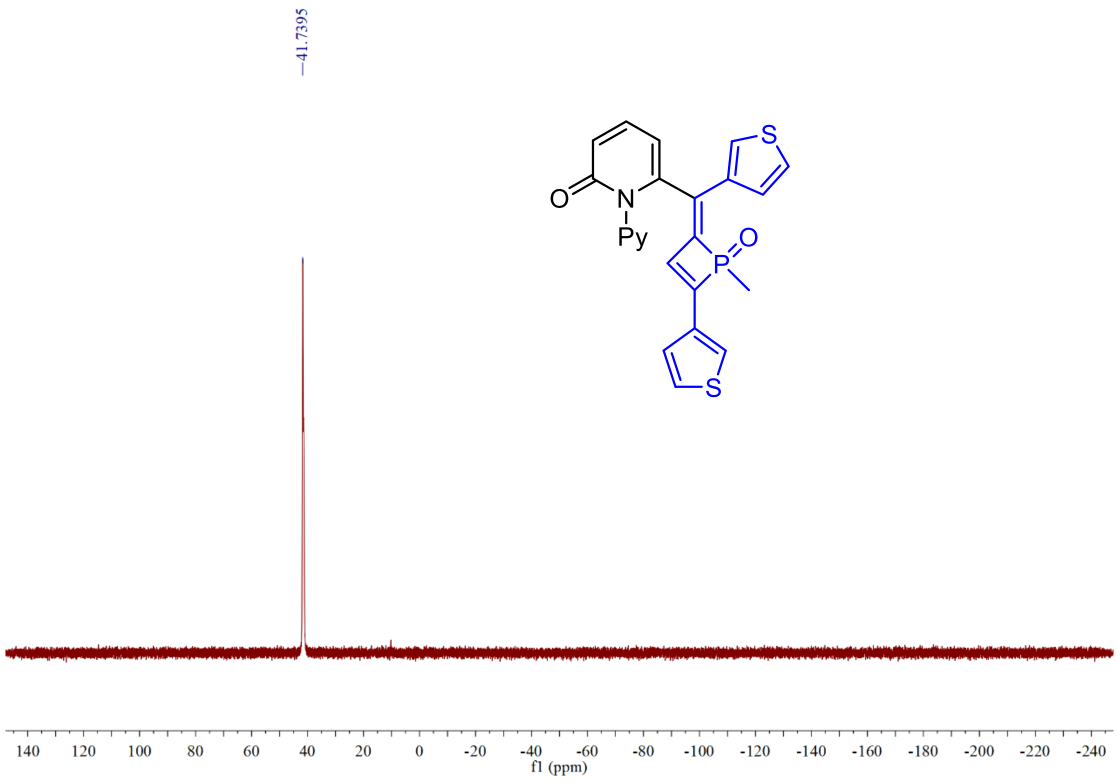

^{31}P NMR spectrum of compound **6v**

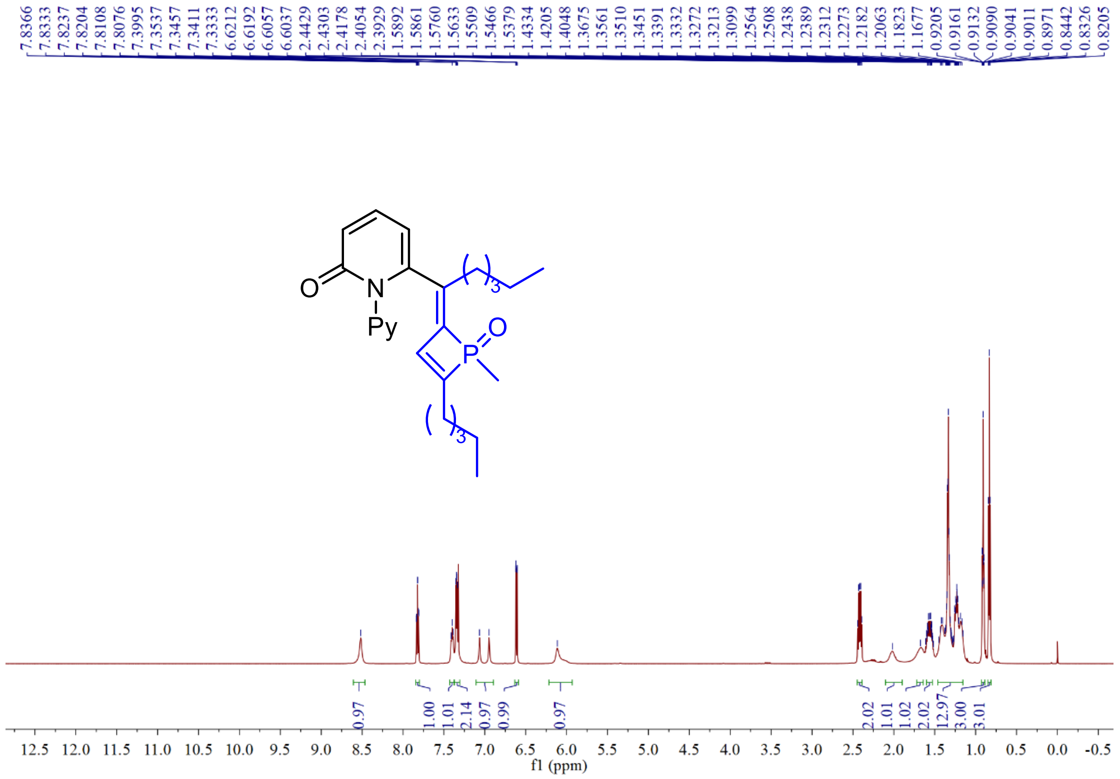

^{1}H NMR spectrum of compound **6w**

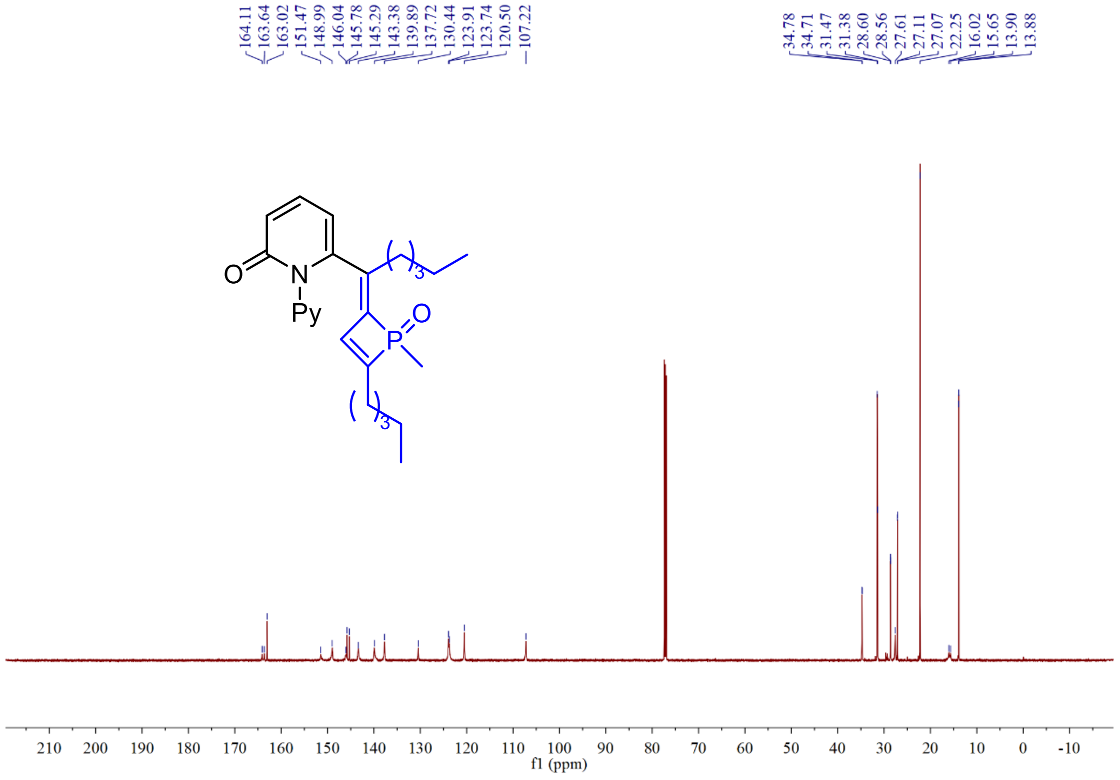

^{13}C NMR spectrum of compound **6w**

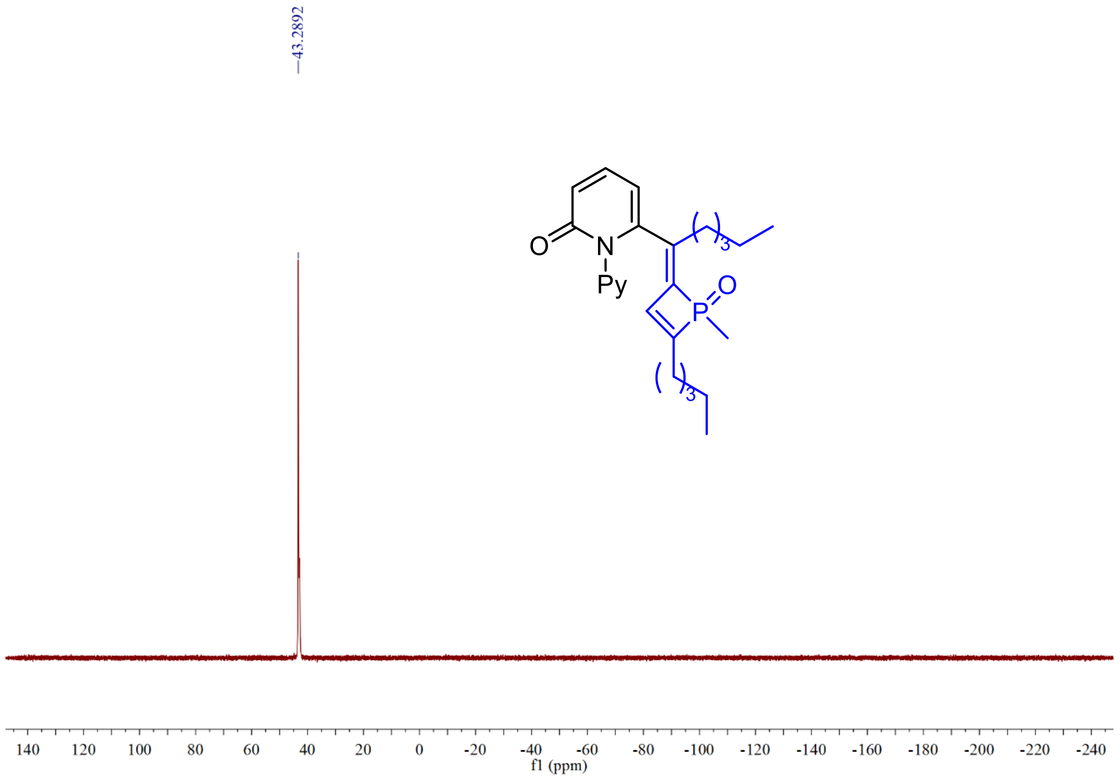

^{31}P NMR spectrum of compound **6w**

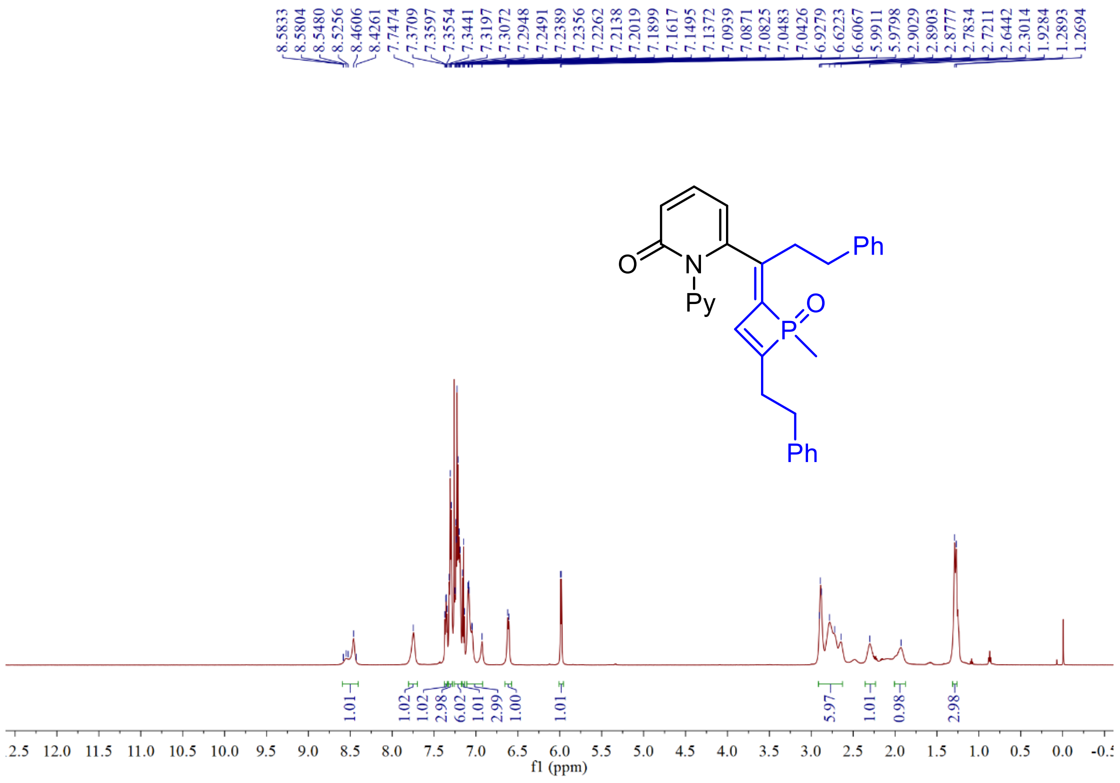

^{1}H NMR spectrum of compound **6x**

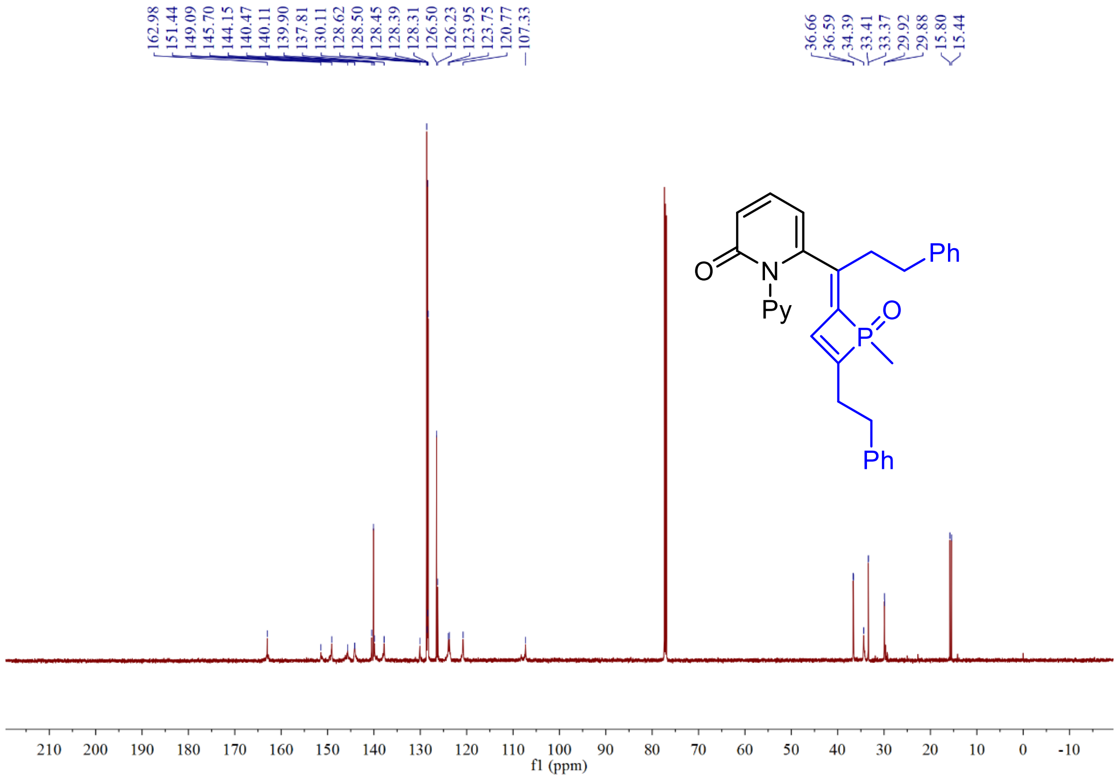

^{13}C NMR spectrum of compound **6x**

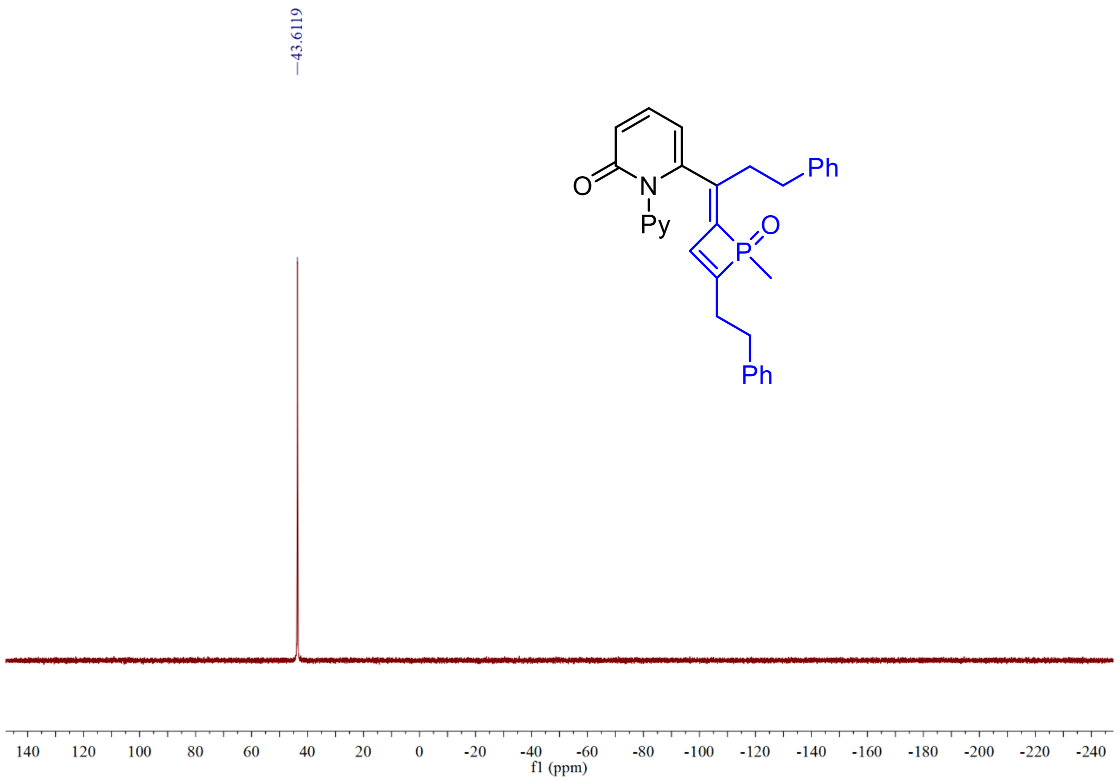

^{31}P NMR spectrum of compound **6x**

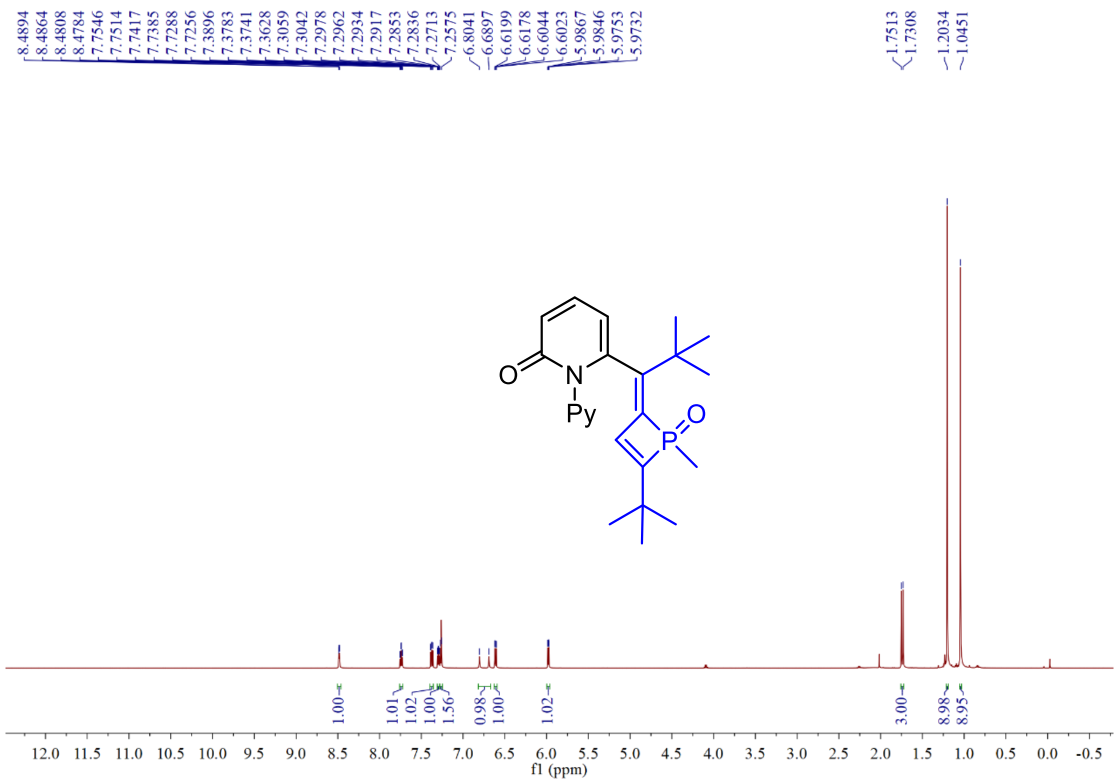

^{1}H NMR spectrum of compound **6y**

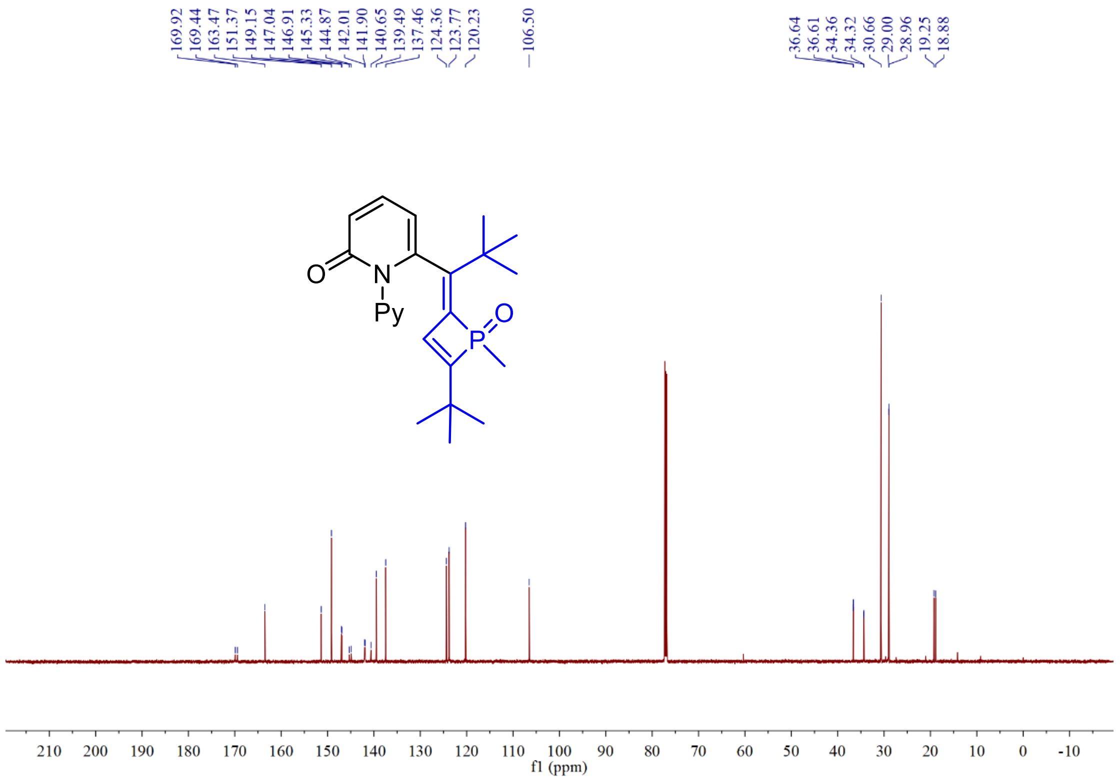

^{13}C NMR spectrum of compound **6y**

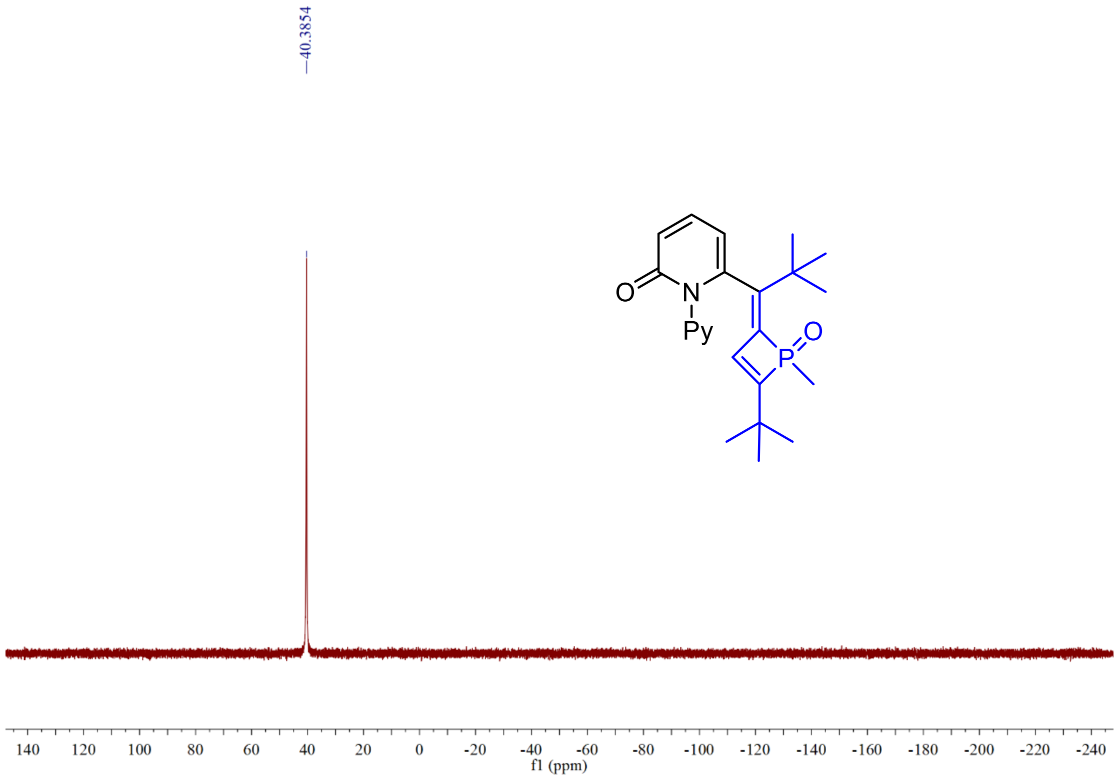

^{31}P NMR spectrum of compound **6y**

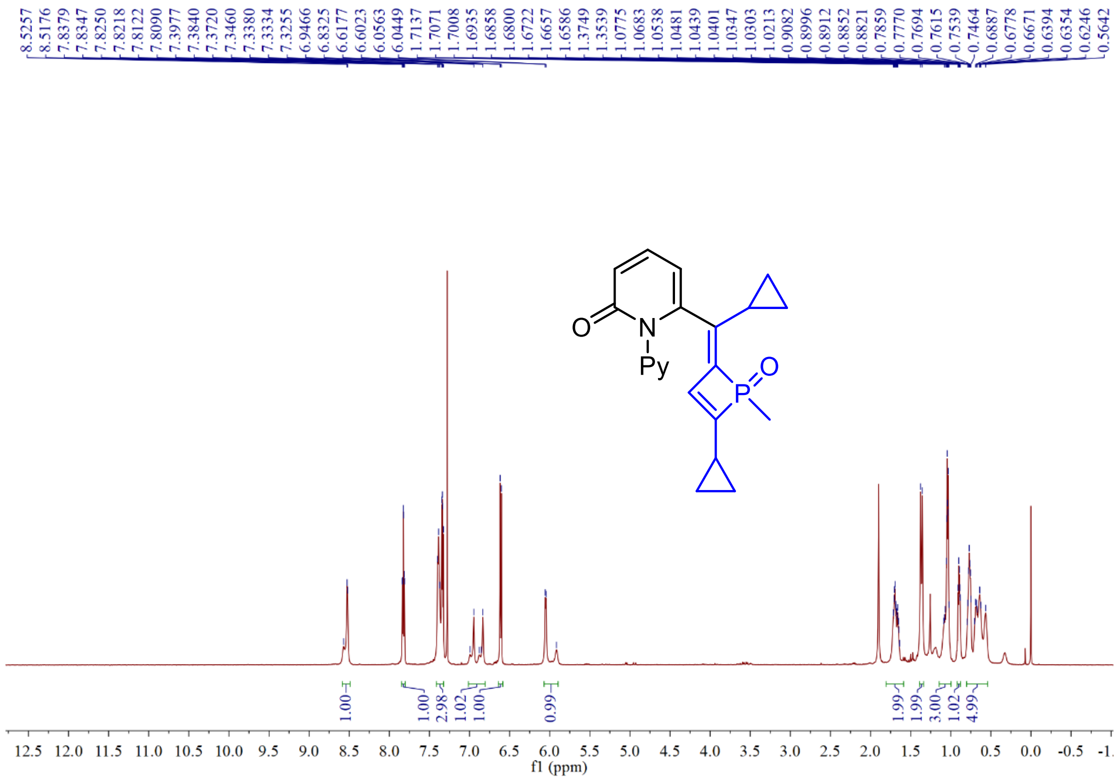

^{1}H NMR spectrum of compound **6z**

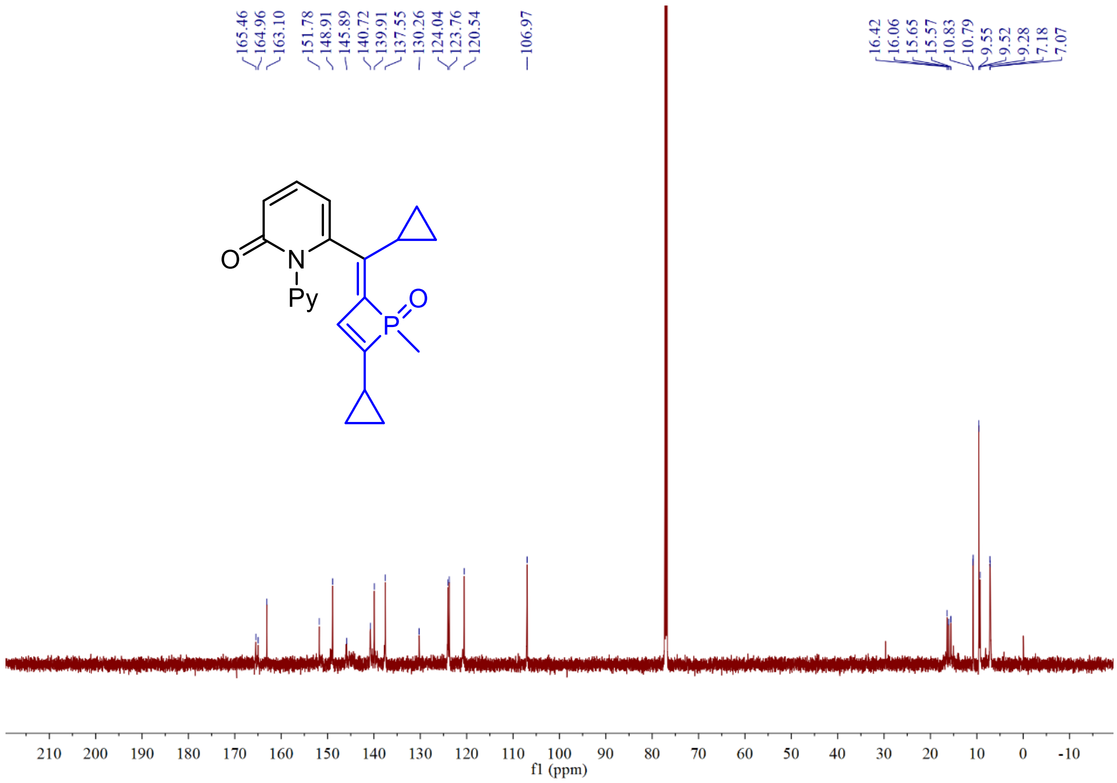

^{13}C NMR spectrum of compound **6z**

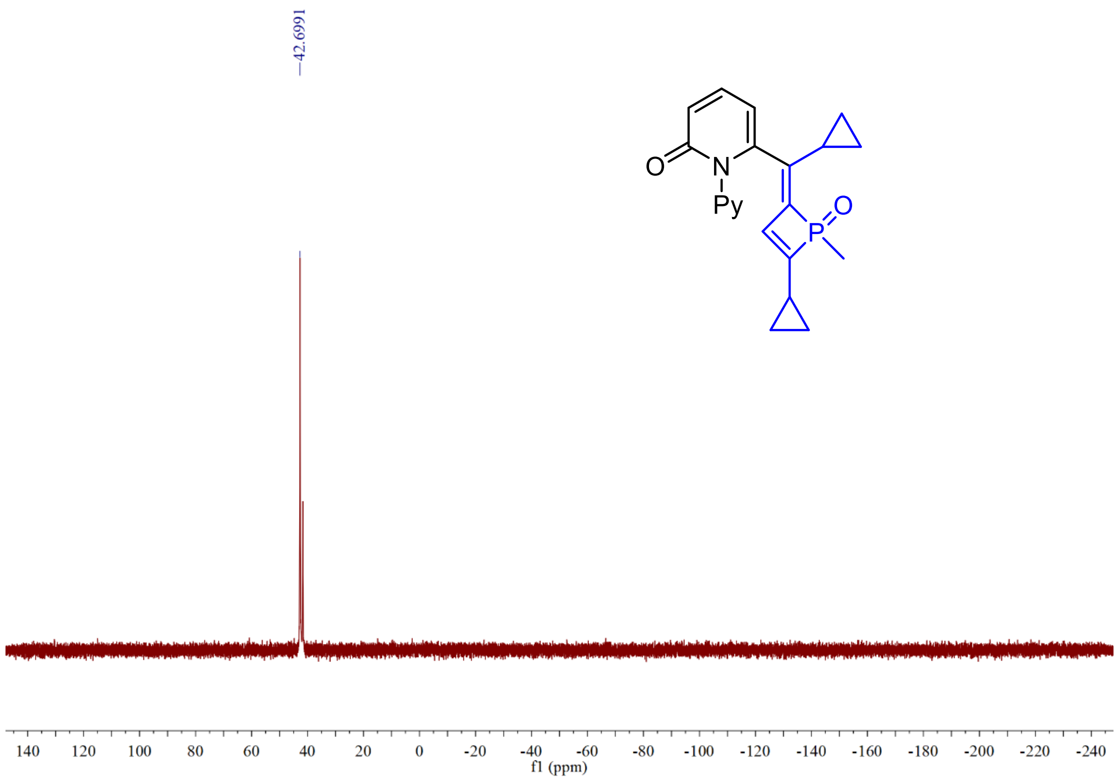

^{31}P NMR spectrum of compound **6z**

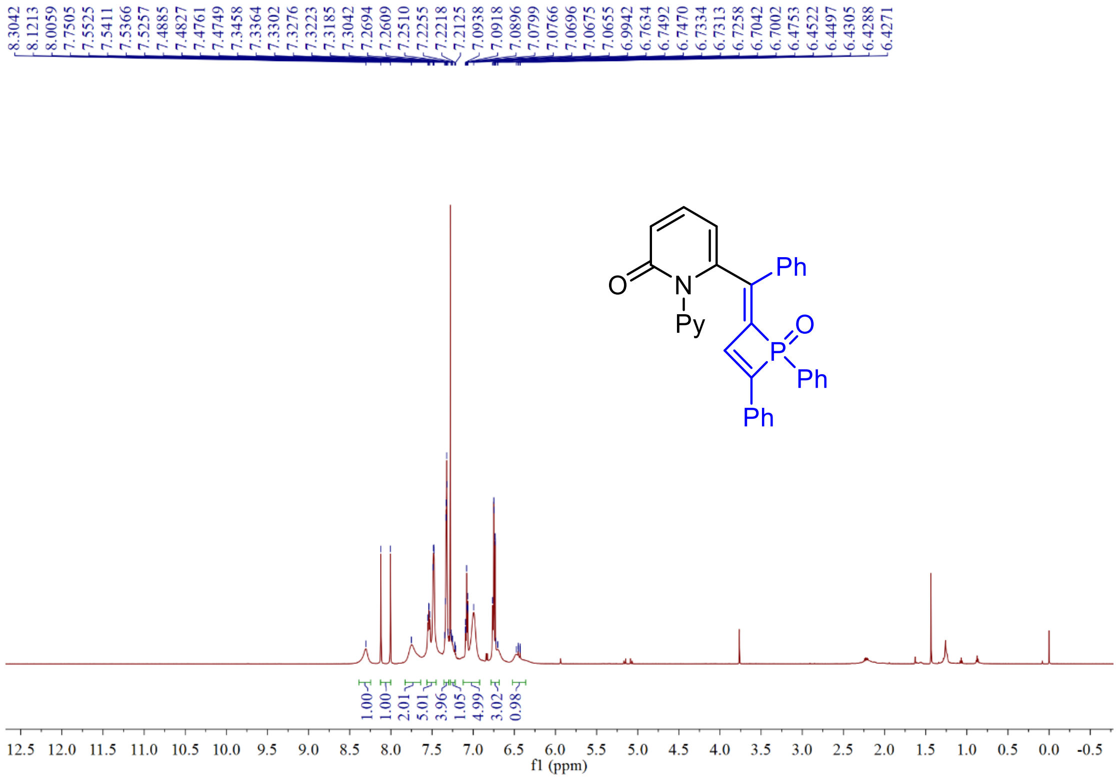

^{1}H NMR spectrum of compound **6aa**

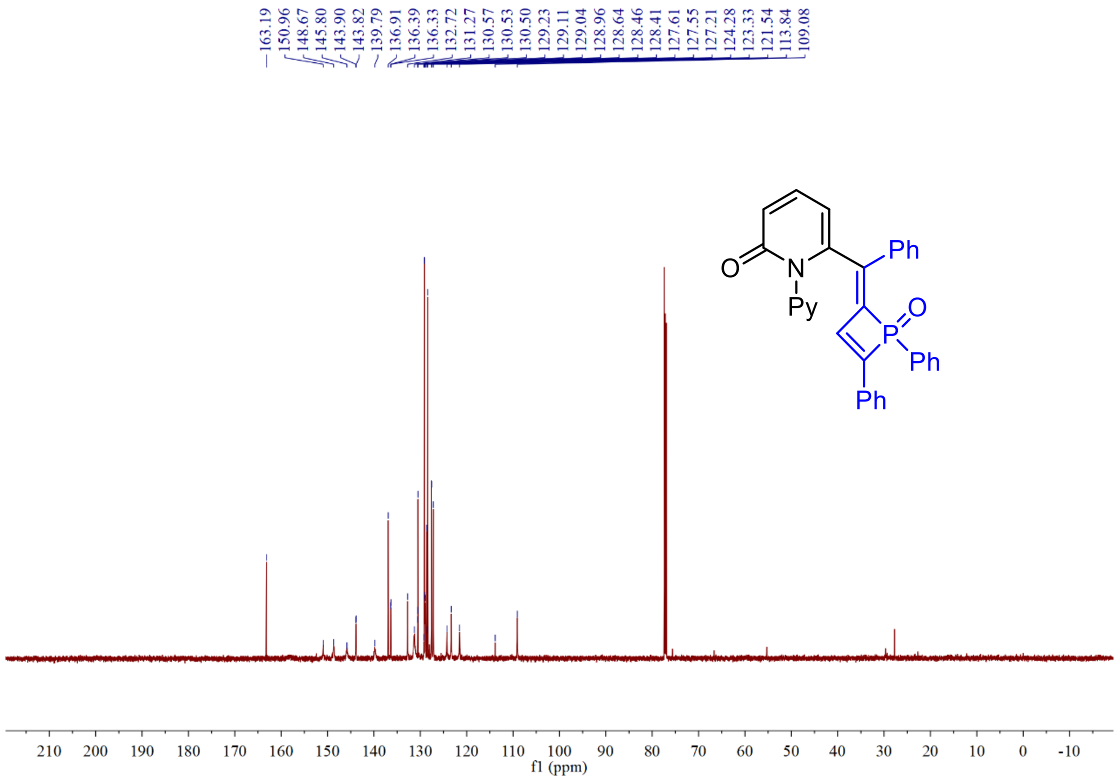

^{13}C NMR spectrum of compound **6aa**

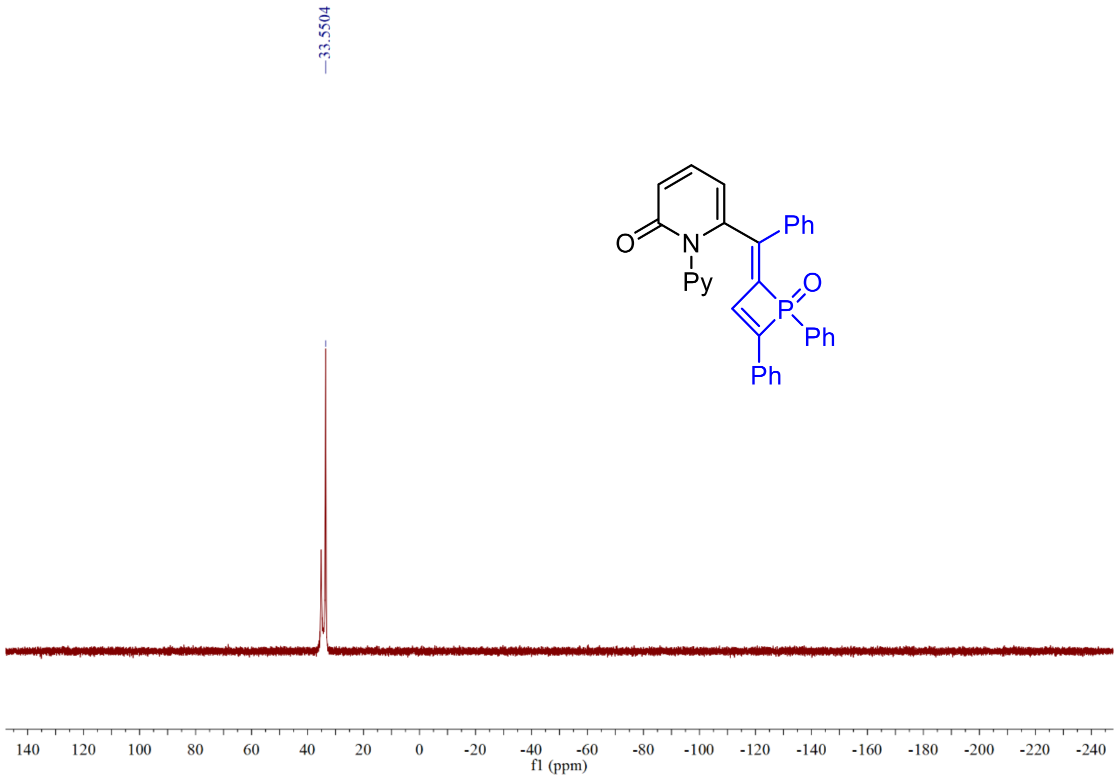

^{31}P NMR spectrum of compound **6aa**

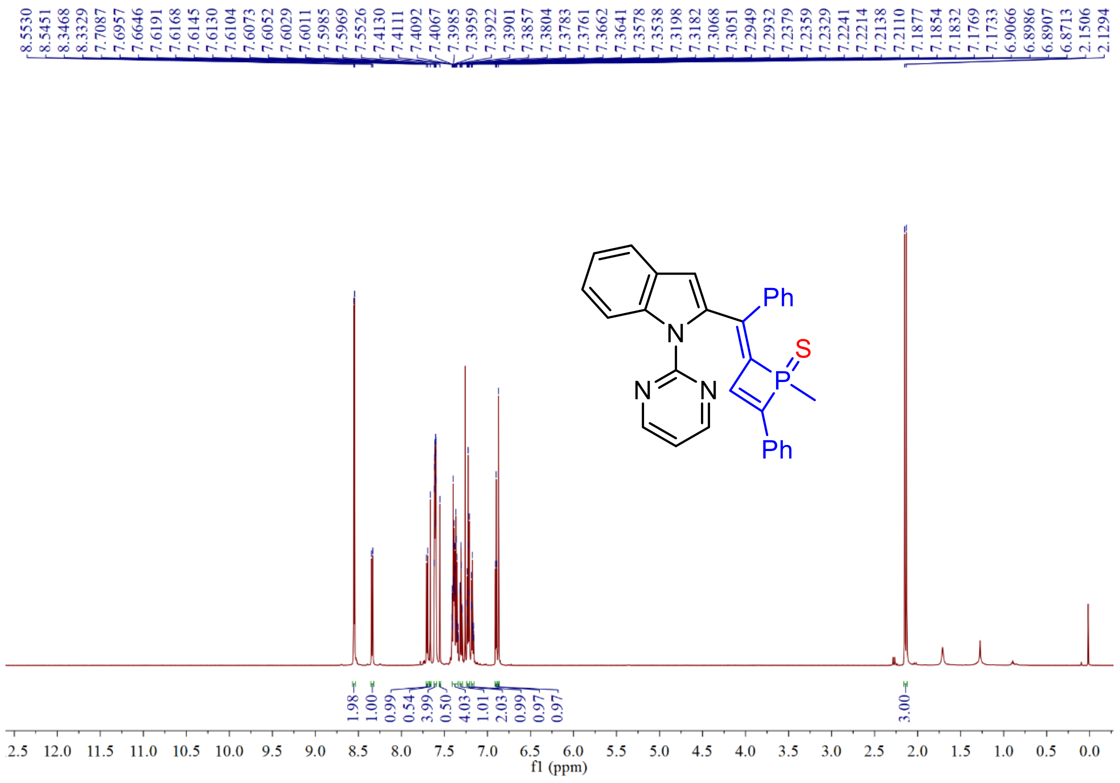

^{1}H NMR spectrum of compound **7**

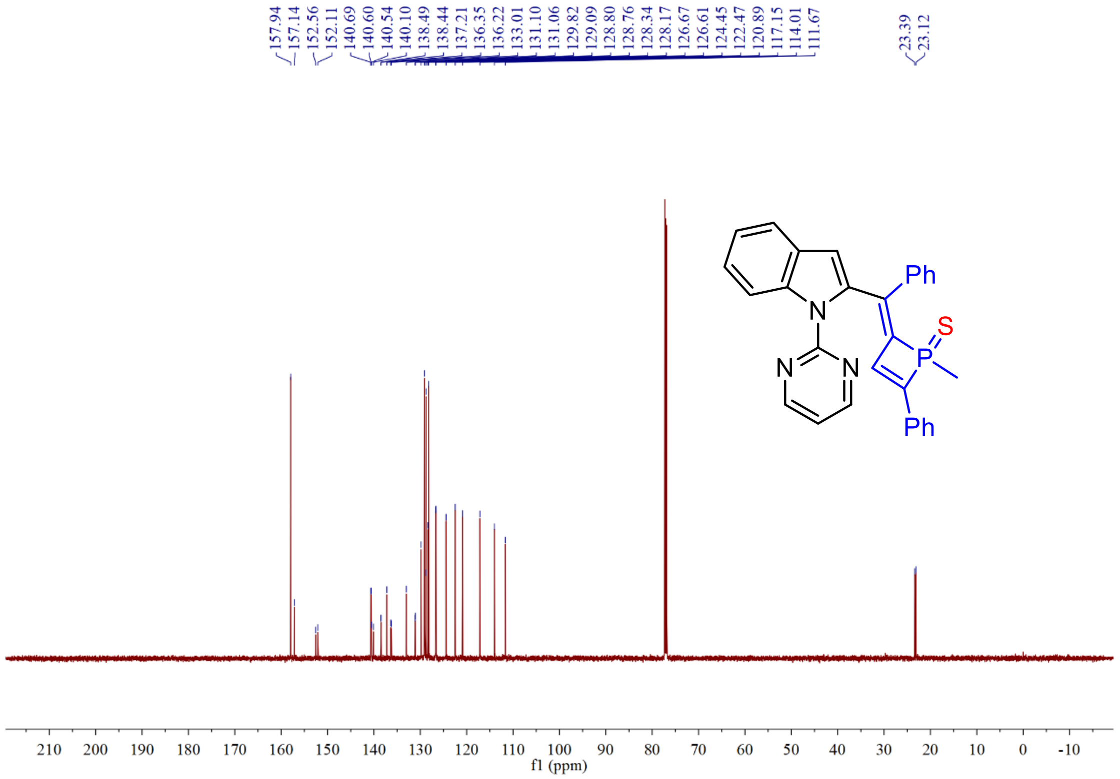

^{13}C NMR spectrum of compound **7**

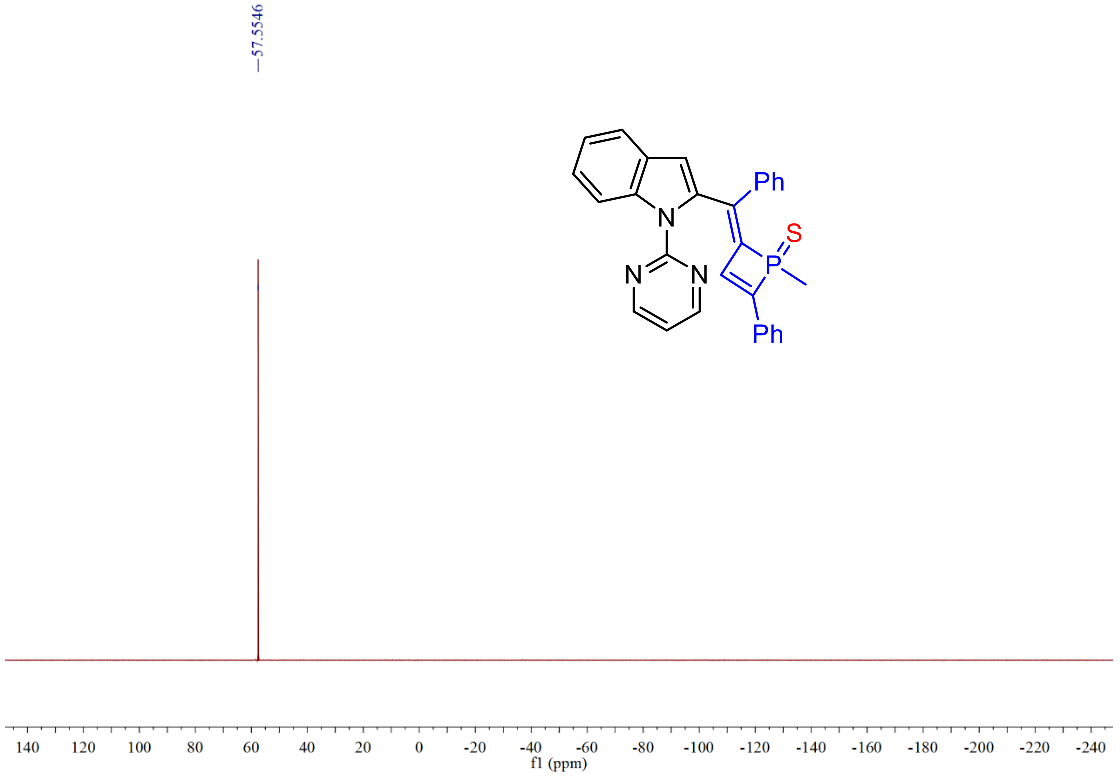

^{31}P NMR spectrum of compound **7**

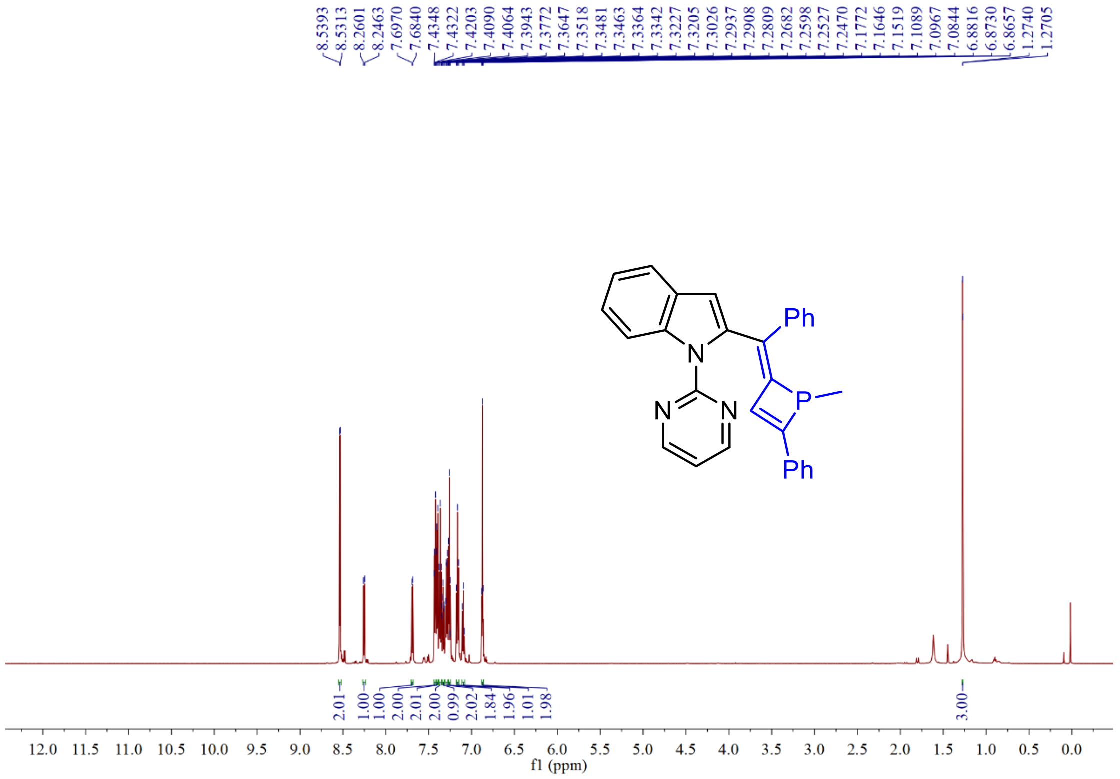

^{1}H NMR spectrum of compound **8**

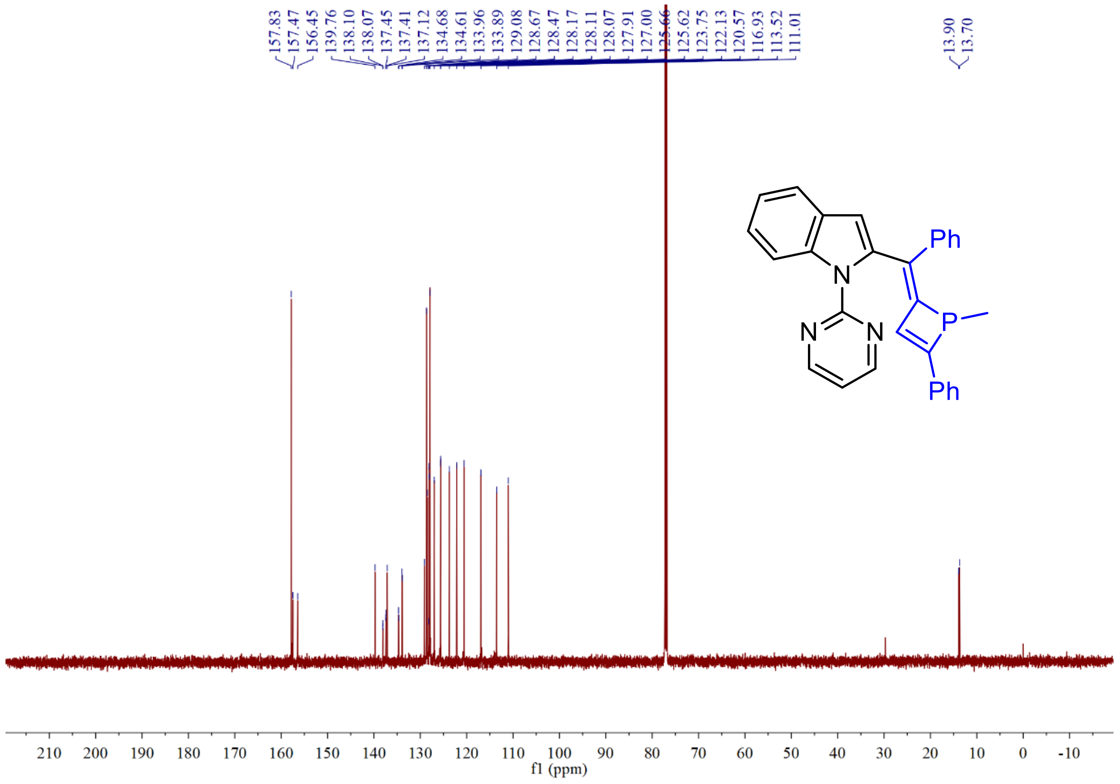

^{13}C NMR spectrum of compound **8**

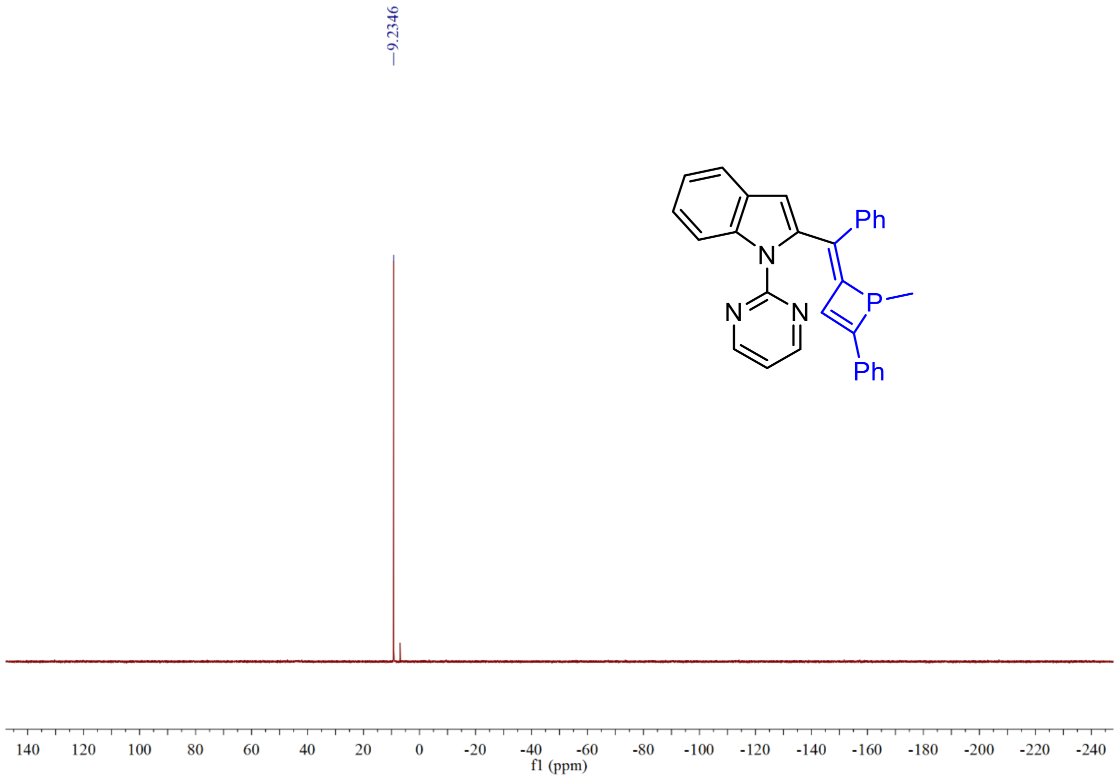

^{31}P NMR spectrum of compound **8**

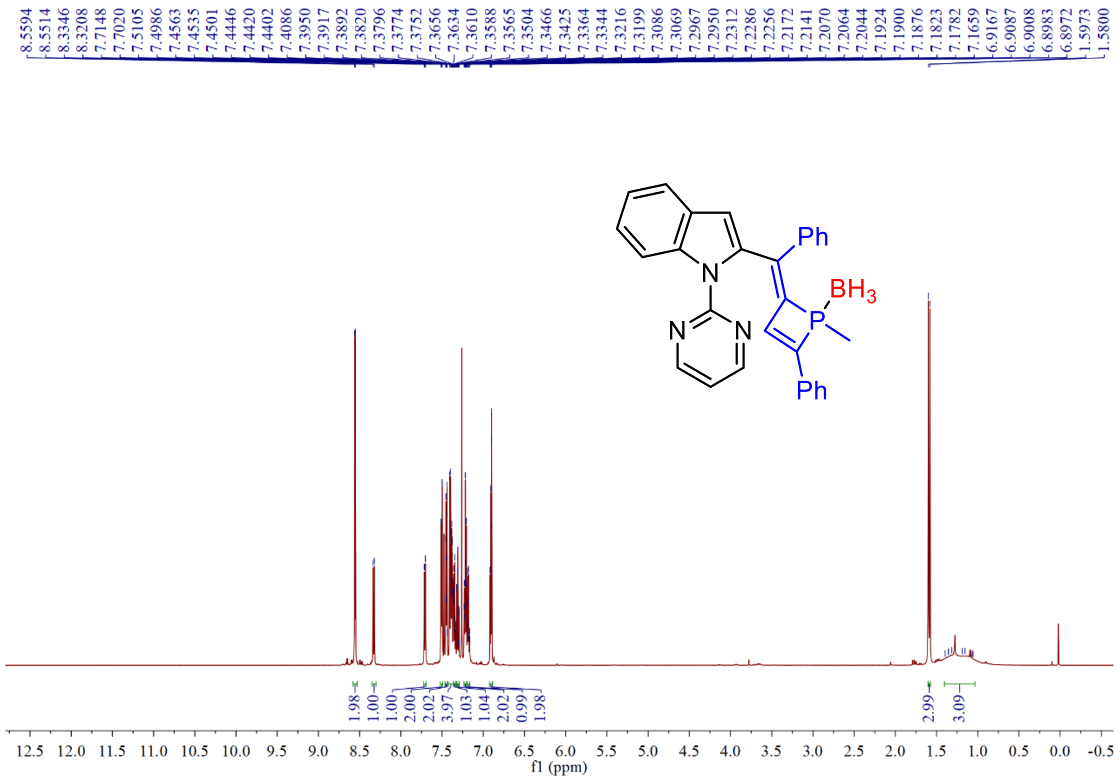

^{1}H NMR spectrum of compound **9**

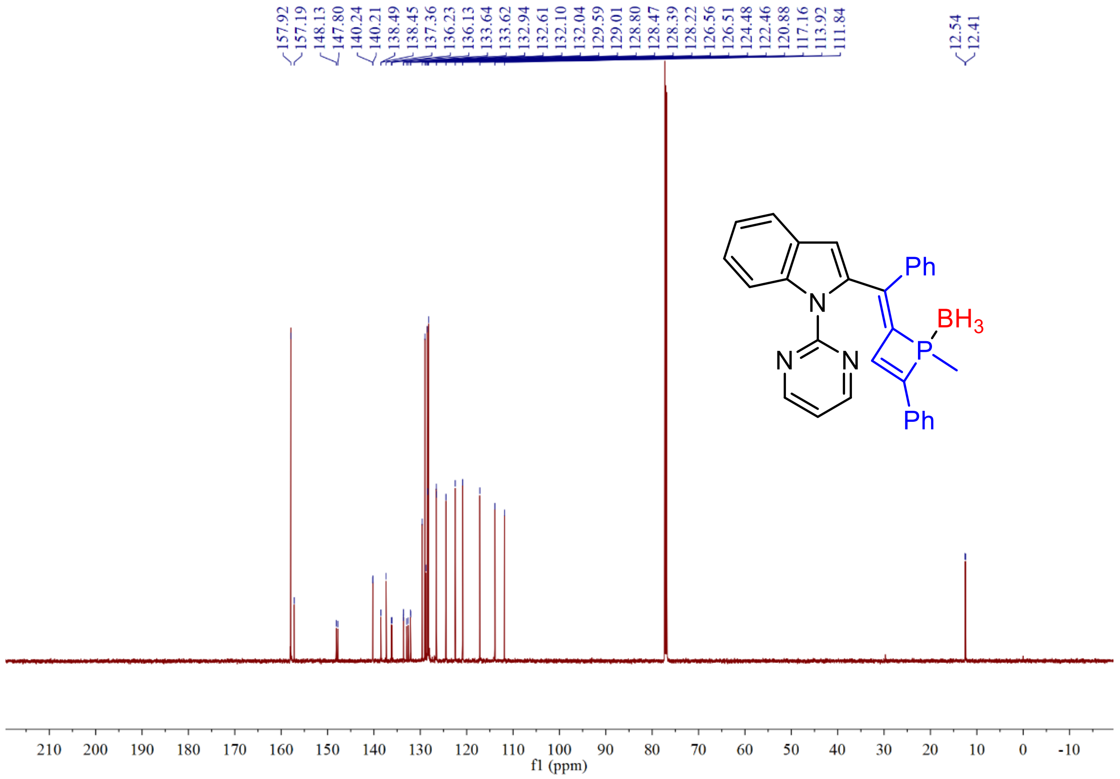

^{13}C NMR spectrum of compound **9**

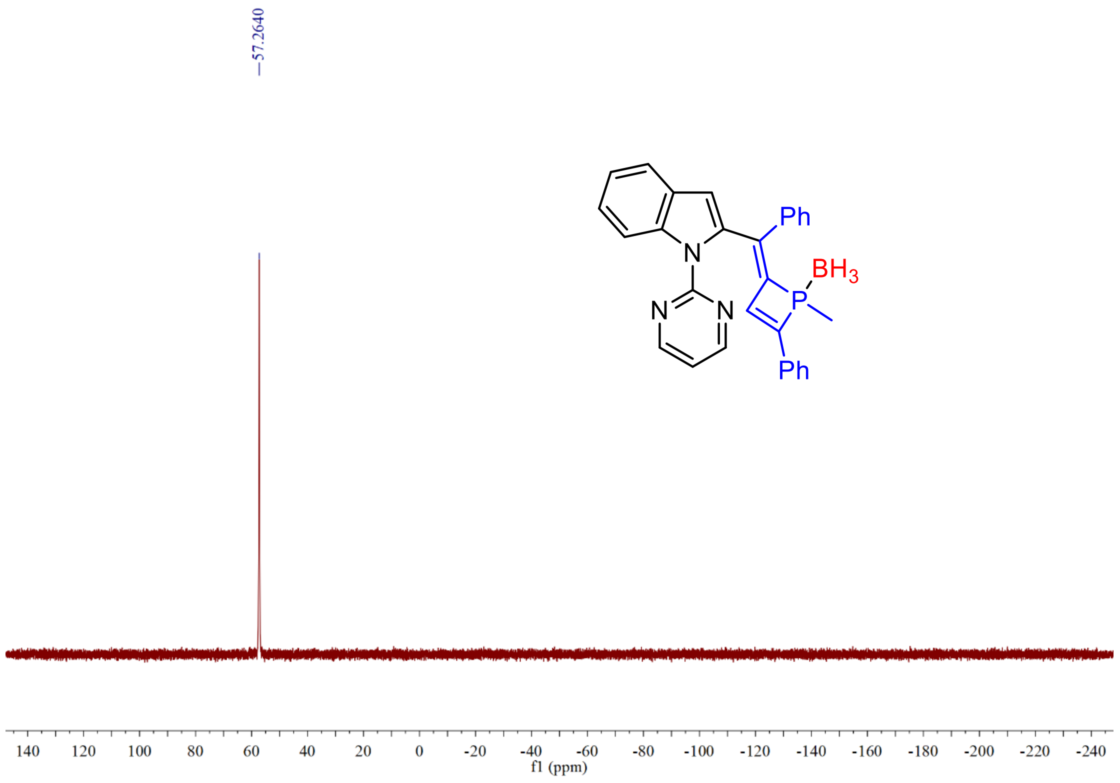

^{31}P NMR spectrum of compound **9**

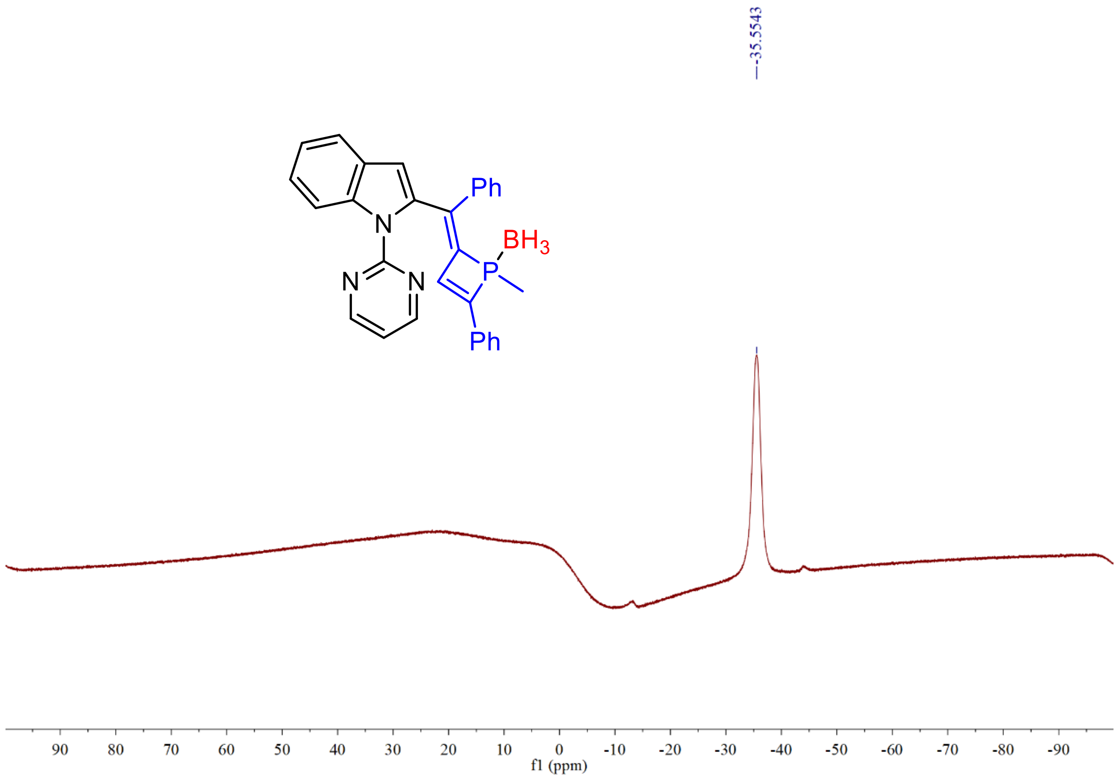

^{11}B NMR spectrum of compound **9**

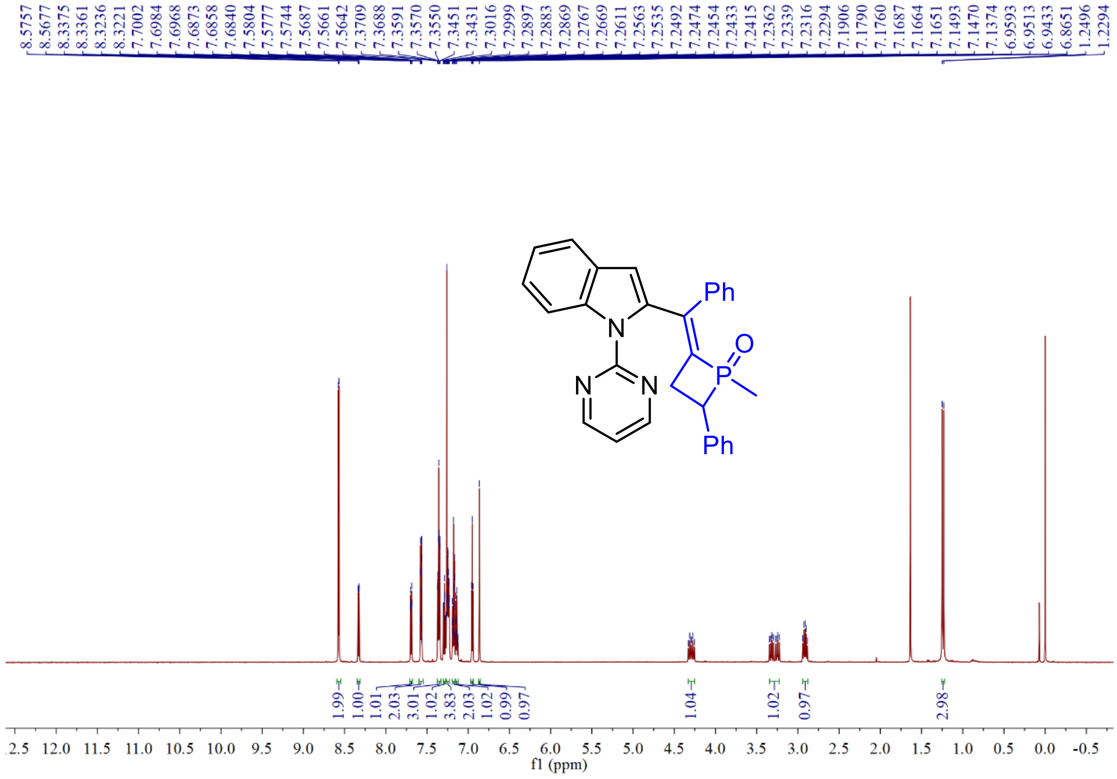

^{1}H NMR spectrum of compound **10**

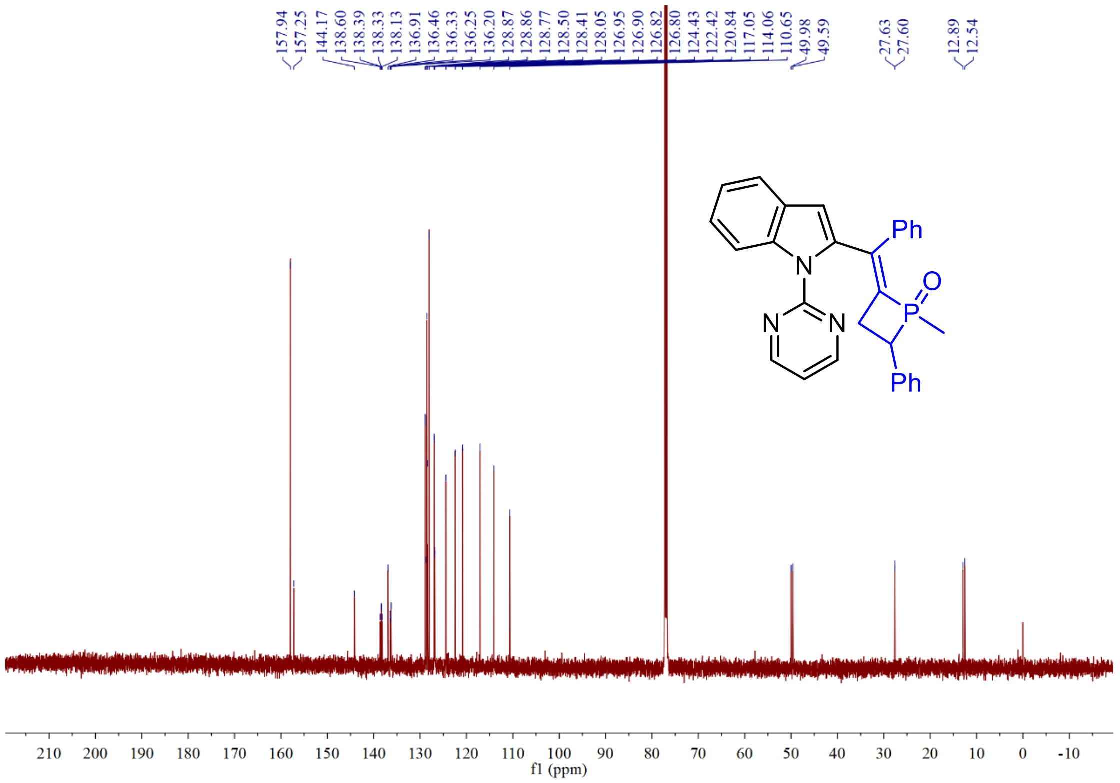

^{13}C NMR spectrum of compound **10**

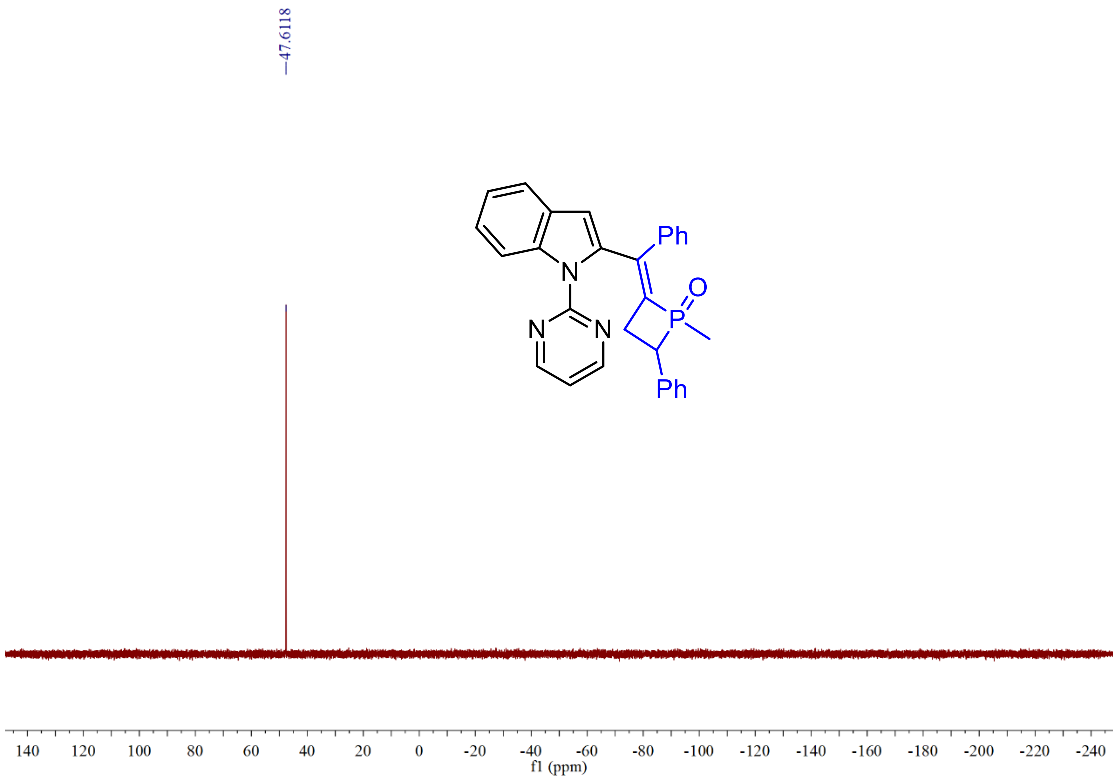

^{31}P NMR spectrum of compound **9**

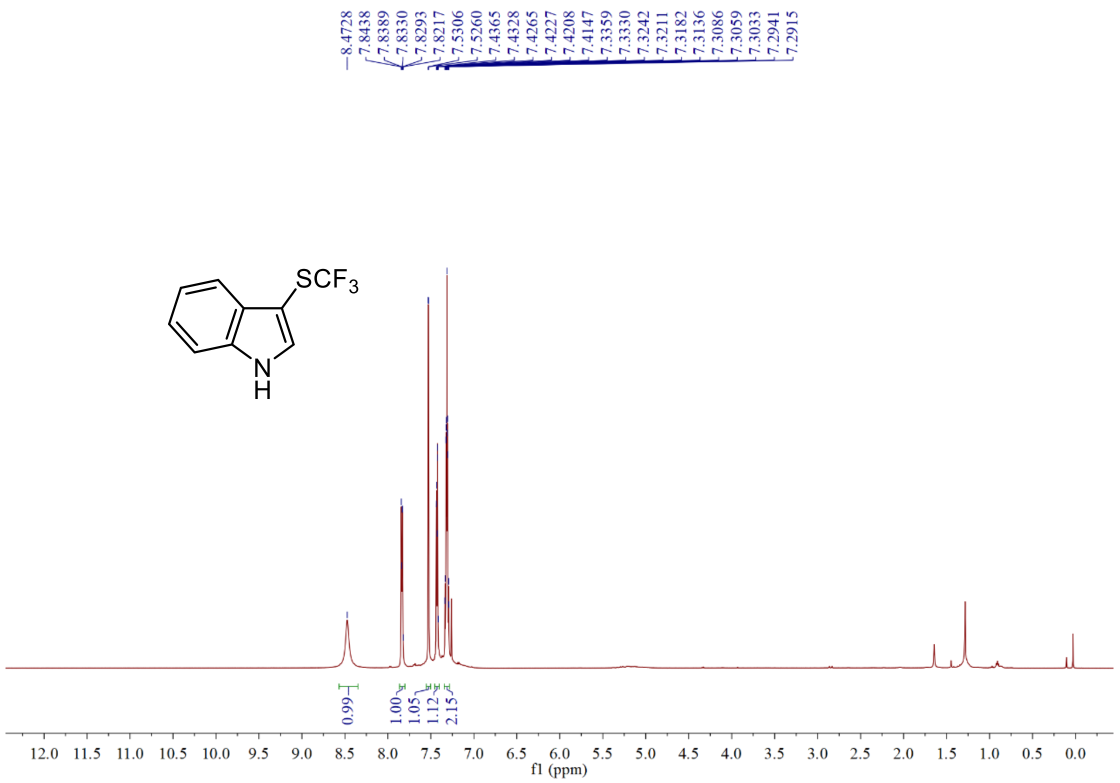

^{1}H NMR spectrum of compound **12**

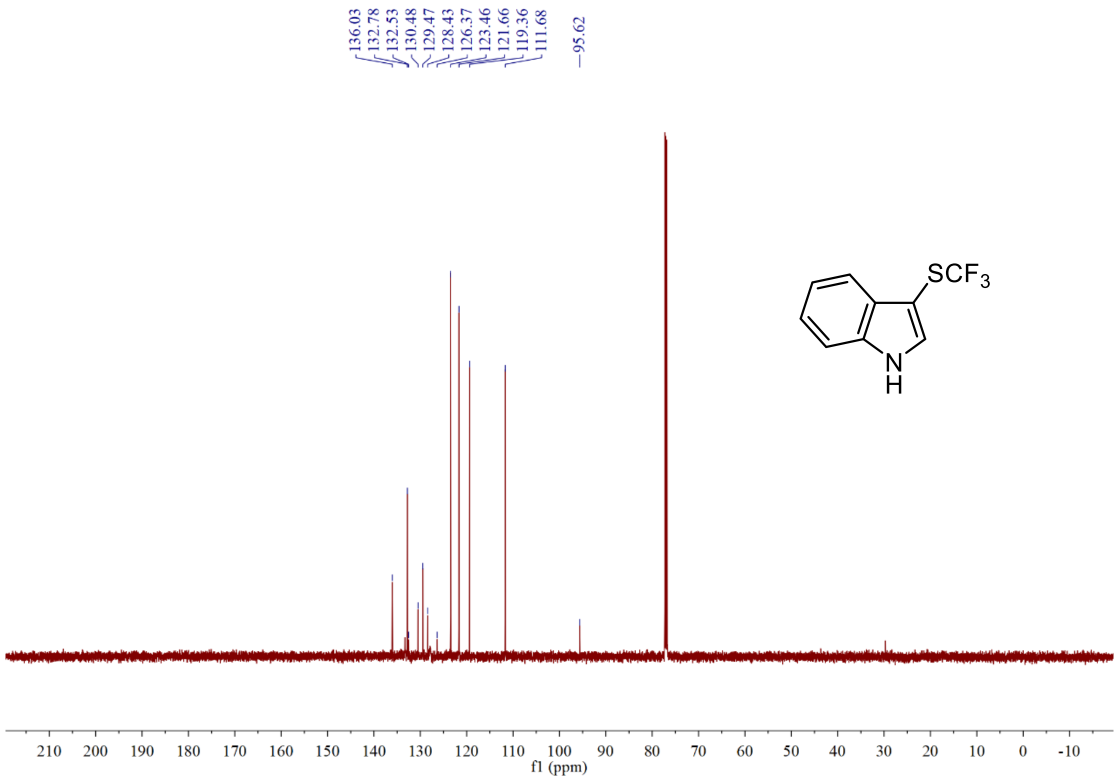

^{13}C NMR spectrum of compound **12**

9 References

[1] Ackermann, L.; Lygin, A. V. Ruthenium-Catalyzed Direct C–H Bond Arylations of Heteroarenes. *Org. Lett.* **2011**, *13*, 3332 – 3335.

[2] Mohanty, S. R.; Prusty, N.; Gupta, L.; Biswal, P.; Ravikumar, P. C. Cobalt(III)-Catalyzed C-6 Alkenylation of 2-Pyridones by Using Terminal Alkyne with High Regioselectivity. *J. Org. Chem*. **2021**, *86*, 9444 − 9454.

[3] Zhang, S.; Cheng, H.; Mo, S.; Yin, S.; Zhang, Z.; Wang, T. Gold(I)-Catalyzed Synthesis of Six-Membered P, O-Heterocycles via Hydration/Intramolecular Cyclization Cascade Reaction. *Adv. Synth. Catal*. **2019**, *361*, 4227 – 4231.

[4] Ghosh, A.; Lecomte, M.; Kim-Lee, S-H.; Radosevich, A. Organophosphorus-Catalyzed Deoxygenation of Sulfonyl Chlorides: Electrophilic (Fluoroalkyl)sulfenylation by P^{III}/P^{V}=O Redox Cycling. *Angew. Chem. Int. Ed*. **2019**, *58*, 2864 – 2869.
